# Improved Accuracy in Semi-Experimental Structure Determination by Resolving Problems Associated with Rotation of Principal Inertial Axes of Isotopologues: Structures of 1,3-Oxazole (*c*‑C_3_H_3_NO)

**DOI:** 10.1021/acs.jpca.6c01535

**Published:** 2026-04-24

**Authors:** Brian J. Esselman, Maria A. Zdanovskaia, Madeleine G. Atwood, Taylor K. Adkins, Manamu Kobayashi, Shozo Tsunekawa, Kaori Kobayashi, Nitai P. Sahoo, John F. Stanton, R. Claude Woods, Robert J. McMahon

**Affiliations:** † Department of Chemistry, 5228 University of Wisconsin-Madison, 1101 University Avenue, Madison, Wisconsin 53706−1322, United States; ‡ Department of Physics, 34823 University of Toyama, 3190 Gofuku, Toyama 930-8555, Japan; § Quantum Theory Project, Departments of Physics and Chemistry, 3463 University of Florida, Gainesville, Florida 32611, United States

## Abstract

The rotational spectrum of the normal isotopologue of 1,3-oxazole (*c*-C_3_H_3_NO) was observed from 43 to 750 GHz. Over 3900 transitions for the ground vibrational state are measured, assigned, and least-squares fit to sextic centrifugally distorted-rotor Hamiltonians. The measured frequencies and resulting spectroscopic constants from this extended spectral range, combined with previous measurements of the nuclear quadrupole coupling constants, will facilitate astronomical searches for oxazole across the majority of the range of modern radiotelescopes. Spectra for a set of 30 oxazole isotopologues, which include multiple isotopic substitutions of each atom, are used to determine the first semi-experimental equilibrium (*r*_*e*_^SE^) structure and semi-experimental substitution structure (*r*_*s*_^SE^), each using CCSD­(T) computed values for the vibration–rotation interaction and electron-mass corrections. The large number of isotopologues, including 21 isotopologues observed for the first time, and the redundant substitutions of each atom provide sufficient spectroscopic information to determine the *r*_*e*_^SE^ structure with the expected high level of accuracy and precision (0.0001 or 0.0002 Å in bond distances and 0.013 to 0.025° in bond angles). In the course of this study, we analyzed a known issue for some *r*_*e*_^SE^ structure determinations of near-oblate asymmetric tops in which inclusion of individual isotopologues degrades the structure determination. We demonstrate that this problem primarily arises from the difference in the values of the computed vibration–rotation interaction corrections as evaluated at the computed *r*_*e*_ geometry vs the *r*_*e*_^SE^ geometry of the “real” molecule. Our solution to this problem substantially improves the *r*_*e*_^SE^ structure of oxazole and likely can be generalized to many other molecules.

## Graphical abstract

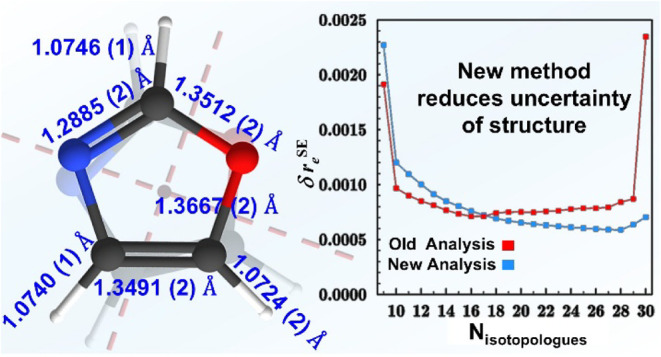

## Introduction

Oxazole (1,3-oxazole, *c*-C_3_H_3_NO, *C*_*s*_, Figure 1) is an aromatic heterocycle with a moderate dipole moment (μ_*a*_ = 1.34 D, μ_*b*_ = 0.66 D),^1^ whose derivatives are important species in organic chemistry as natural products^2^ and pharmaceuticals.^3^ The rotational spectrum of oxazole was first measured in 1966 by Mackrodt et al.,^1^ providing its rotational and nuclear quadrupole-coupling constants. In 1974, Flygare and coworkers measured the Zeeman effect and magnetic susceptibility of oxazole.^4^ In 1978, Stiefvater and coworkers reinvestigated the rotational spectrum of oxazole in a series of works.^5–7^ They remeasured the microwave spectrum of the normal isotopologue of oxazole, improving the precision of its rotational constants and providing the first determination of some quartic centrifugal distortion constants.^7^ They analyzed the spectra of all singly substituted heavy-atom isotopologues at natural abundance using double-resonance microwave (DRM) spectroscopy.^6,7^ Additionally, the spectra of all singly substituted deuterated species using isotopically enriched mixtures were observed with DRM spectroscopy.^7^ The dipole moment was determined, along with the nuclear quadrupole-coupling parameters for oxazole and its three monodeuterium-substituted isotopologues.^5^ With the observed rotational constants (*B*_0_) of nine isotopologues, Stiefvater and coworkers determined a substitution structure (*r*_s_) and a structure determined by least-squares fitting the *B*_0_ constants (*r*_0_) that were in good agreement.^7^ The *r*_s_ structure determined the bond distances and bond angles to precisions (2σ) of ≤0.006 Å and ≤1.2°, respectively, while the *r*_0_ structure determined the bond distances and bond angles to precisions (2σ) of <0.002 Å and 0.2°. A high-resolution infrared study from 600 to 1400 cm^–1^ of oxazole was completed in 2007 by Hegelund et al.,^8^ providing updated rotational constants and a complete set of quartic centrifugal distortion constants for the vibrational ground state, as well as for 10 excited vibrational fundamental states. The previously reported spectroscopic constants of oxazole and its isotopologues provided the foundation for the current spectroscopic investigation.^1,5–8^

**Figure 1:**
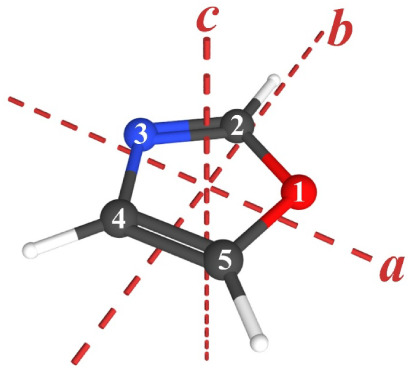
1,3-Oxazole (*c*-C_3_H_3_NO, *C_s_*, μ*_a_* = 1.34 D, μ*_b_* = 0.66 D^1^) with principal inertial axes and atom numbering.

Recent advances in structure determination have resulted in semi-experimental equilibrium (*r*_*e*_^SE^) structures for aromatic species with bond distances and angles more than an order of magnitude more precise than those previously reported for oxazole.^9–19^ For example, the *r*_*e*_^SE^ structural parameters of thiophene were determined with 2σ statistical uncertainties of ≤0.0002 Å for bond distances and ≤0.02° for bond angles.^18^ In these works,^9–18^ computed equilibrium (*r*_*e*_) structures (CCSD­(T)/cc-pCV5Z or CCSD­(T)/cc-pCV6Z) are improved by estimates of the residual untreated electron correlation, an extrapolation to the infinite basis-set limit, estimates of relativistic effects, and the diagonal Born–Oppenheimer correction. For pyrimidine,^9^ pyridazine,^12^ 1*H*-1,2,4-triazole,^13^ hydrazoic acid,^14^ 2*H*-1,2,3-triazole,^15^ and ketene,^17^ there is agreement between the theoretical *r*_*e*_ and semi-experimental *r*_*e*_^SE^ structure within the 2σ statistical uncertainty of the *r*_*e*_^SE^ structure. For thiophene,^11,18^ thiazole,^10^ 1*H*-1,2,3-triazole,^15^ benzene,^16^ and pyridine,^19^ however, one or more of the *r*_*e*_ structural parameters fall outside of the 2σ limits of the *r*_*e*_^SE^ structural parameters. For benzene, this can be attributed to the remarkably small (<0.000 02 Å) uncertainty of its σ_C–C_ distance. For the two sulfur-containing aromatic heterocycles, thiophene^11,18^ and thiazole,^10^ the discrepancy has been attributed to the inability of theory to adequately model the larger sulfur atom.

Achieving a high-quality structure of oxazole necessitated the investigation of a known problem in structure determination associated with the isotope-induced rotation of the principal inertial axes in oblate asymmetric-top molecules.^20,21^ The analysis presented herein affords new insight into this problem and develops a new method for treating the data.

Along with other aromatic heterocycles, oxazole and its derivatives are of astrochemical interest as potential prebiotic organic compounds.^22^ Among the approximately 340 molecules detected in the interstellar medium (ISM) or circumstellar shells, a number of aromatic organic compounds have recently been detected.^23–37^ The likelihood that other aromatic species are present in these environments has inspired searches, as yet unsuccessful, for aromatic heterocycles.^38–41^ The possibility of detecting these species, including oxazole, depends upon the existence of sufficiently precise microwave or millimeter-wave laboratory rotational spectra and a suitable intensity, which is governed by a combination of the dipole moment and molecular abundance, in the source. While oxazole is not predicted to be the most likely C_3_H_3_NO isomer for interstellar detection,^42^ it is nevertheless a likely candidate for detection with continued improvements in telescopes. The spectroscopic constants and transition frequencies of oxazole presented in this work will provide the foundation for future radioastronomical searches, particularly at frequencies above 40 GHz.^42^

## Methods

### Spectroscopic Methods

Rotational spectra of the normal isotopologue and the singly substituted heavy-atom isotopologues (at natural abundance) of oxazole were collected at room temperature using two spectrometers at the University of Toyama and a spectrometer at the University of Wisconsin–Madison from commercially available samples without additional purification. At the University of Toyama, the spectrum of oxazole was collected from 40–330 GHz at sample pressures of 15 to 50 mTorr using instrumentation described previously.^43,44^ The microwave signal generated by the synthesizer with a 50 kHz frequency modulation was multiplied, passed through the waveguide/free-space cell, and subsequently detected using a liquid-He-cooled InSb detector. Frequencies below 20 GHz were collected using a chirped-pulse Fourier-transform microwave spectrometer (CP-FTMW) at the University of Toyama. The CP-FTMW spectrometer design is based on those of McJunkins and Brown^45^ and Shipman and coauthors.^46^ Spectra were measured at room temperature using a frequency-appropriate waveguide and sample pressures of 15–50 mTorr. A total of 10,000 molecular signal acquisitions were achieved using a 100 MHz chirp span. Line widths are approximately 400 kHz with a nominal measurement uncertainty of 30 kHz. At the University of Wisconsin–Madison, continuous broadband spectra of oxazole were obtained in the 78–130 GHz, 130–230 GHz, 235–360 GHz, 350–500 GHz, and 500–750 GHz frequency regions, using an instrument described previously.^17,47–49^ Spectra were obtained automatically at a rate of approximately 55 GHz per day, given the following experimental parameters: 0.6 MHz/s sweep rate, 10 ms time constant, and 50 kHz AM and 500 kHz FM modulation in a tone-burst design.^50^ Since it was not possible to collect the full 78–750 GHz frequency region for most synthesized samples, variable frequency regions among those listed above were collected for different samples with the goal of collecting as much data as possible for the observed isotopologues. Spectral segments were combined into a single broadband spectrum using Kisiel’s Assignment and Analysis of Broadband Spectra (AABS) software package.^51,52^ SPFIT/SPCAT^53^ were used for least-squares fits and spectral predictions, along with PLANM and AC programs for analysis.^54,55^ Measurements from Wisconsin and Toyama assume experimental uncertainty of 50 and 30 kHz, respectively, while all previously reported measurements use the uncertainty that was provided in the original publication. All least-squares fitting files are available in the Supporting Information.

### Synthesis of Oxazole-*d_x_* Isotopologues

Deuterium-containing samples of oxazole were prepared from commercial samples of oxazole via exchange reactions with D_2_O (Scheme 1). Base-catalyzed H/D exchange mediated by carbonate in deuterium oxide at room temperature (Scheme 1a), in reflux (Scheme 1b), and heated in a sealed vessel (Scheme 1c) resulted in product mixtures that, respectively, were rich in [2-^2^H]-oxazole, [2,5-^2^H]-oxazole, and [2,5-^2^H]- and [2,4,5-^2^H]-oxazole. The reaction conditions in Scheme 1a and b were based upon previous syntheses of [2-^2^H]- and [2,5-^2^H]-thiazole.^10,56,57^ As expected from previous experience with thiazole, base-catalyzed H/D exchange at the 2-position is by far the most rapid for oxazole.^10,58^ The slowest H/D exchange occurs at the 4-position.^10^ As a result, the conditions in Scheme 1a and b did not yield detectable amounts of [2,4-^2^H]- or [4,5-^2^H]-oxazole. The pressurized reaction, based on the work of Heinrich et al.,^59^ generated predominantly [2,5-^2^H]-oxazole even after two cycles of deuterium enrichment. The second-most-abundant product, however, was [2,4,5-^2^H]-oxazole, and both [2,4-^2^H]- and [4,5-^2^H]-oxazoles were observable in this sample. Fortunately, the variable mixtures of oxazole-*d*_*x*_ isotopologues contained sufficient quantities of various deuterium-containing species that the corresponding heavy-atom isotopologues ([2, 4, or 5-^13^C]-, [3-^15^N]-, and [1-^18^O]-) were able to be observed at natural abundance. Synthetic details are provided in the Supporting Information.

**Scheme 1:**
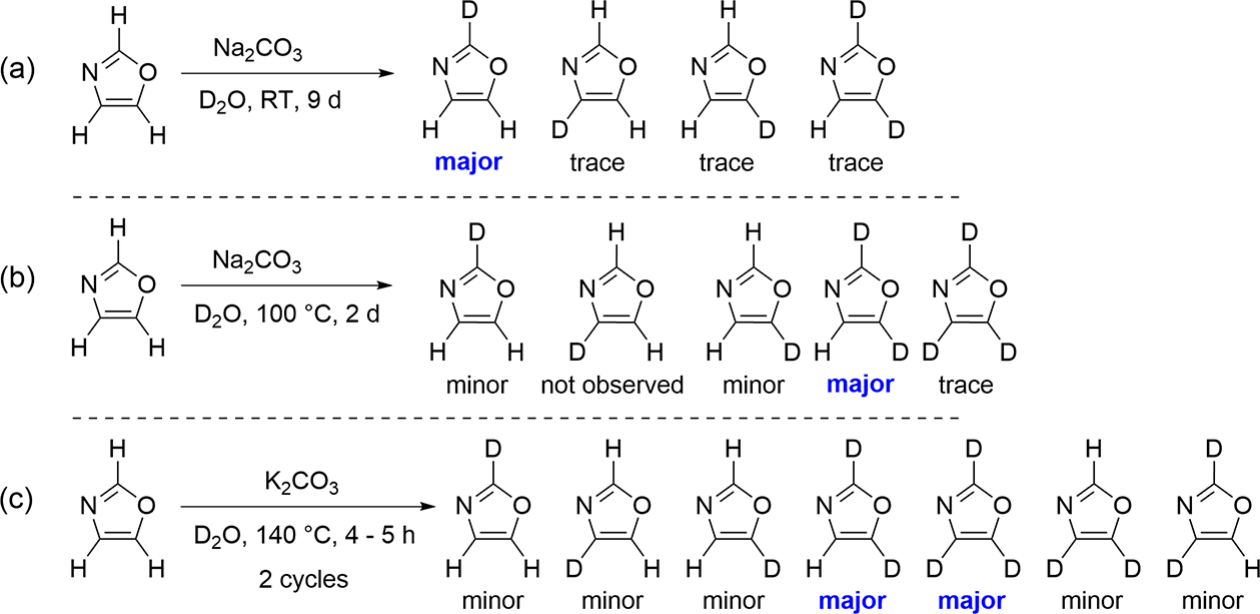
Deuterium Incorporation into Oxazole: (a) by Weak Base at Ambient Conditions, (b) by Weak Base at Reflux, and (c) by Heating with a Weak Base in a Sealed Vessel

### Computational and Structure Determination Methods

Calculations were carried out using a development version of CFOUR.^60^ Computational details are similar to previous works.^9–13,15,16,18,19^ Calculations using cc-pVDZ or cc-pVTZ basis sets^61,62^ were performed with the frozen-core (fc) approximation. Calculations using cc-pCVTZ, cc-pCVQZ, and cc-pCV5Z basis sets^63^ included correlation of all electrons (ae). The oxazole structure optimized at the (ae) CCSD­(T)/cc-pCVTZ level of theory was used for magnetic properties calculations and an anharmonic, second-order vibrational perturbation theory (VPT2) calculation at the same level of theory. The anharmonic frequency calculation evaluates cubic force constants using analytical second derivatives at displaced points.^64–66^ A CCSD­(T)/cc-pCV5Z geometry optimization was used as the basis of the “best theoretical estimate” (BTE). Details of other calculations, including those involved in producing the best theoretical estimate, are described in the Supporting Information.

The *xrefit* module of CFOUR was used to determine the semi-experimental equilibrium structure via least-squares fitting of corrected moments of inertia. *Xrefit* calculates the corrected moments of inertia from input data including experimental averaged determinable rotational constants, computational electron-mass distribution corrections, and computational vibration–rotation interaction corrections per eq 1:^67,68^

1 $$B_{e}^{\beta }=B_{0}^{\prime \prime \beta }+\frac{1}{2}\underset{i}{\sum }\alpha_{i}^{\beta }-\frac{m_{e}}{M_{p}}g^{\beta \beta }B^{\beta }$$

where β refers to the rotational axis, *B*_0_^″^ is the determinable rotational constant,^69^ *B*_*e*_ is the equilibrium constant, the half-sum is the vibration–rotation interaction correction, $\frac{m_{e}}{M_{p}}$ is the electron-to-proton mass ratio, and *g* ^ββ^ is the corresponding magnetic *g*-tensor component.

Because the *Z*-matrix whose parameters are determined in this routine must comprise the minimal parameters necessary to geometrically define the entire molecular structure and standard error propagation assumes independent uncertainties, an additional *Z*-matrix was employed in order to obtain all parameters of interest and their proper uncertainty values. The *xrefiteration* routine^14^ was used to analyze the impact of each isotopologue on the *r*_*e*_^SE^ structure determination.

Semi-experimental substitution (*r*_*s*_^SE^) structures were obtained by calculating the equilibrium rotational constants using eq 1. These rotational constants were used to calculate the equilibrium moments of inertia, which were subsequently used to determine the atomic coordinates in a plane using Kraitchman analysis.^70,71^ Typically, this analysis is carried out using the normal isotopologue as the reference system and the singly substituted isotopologues to determine the coordinates of the various atomic positions. For oxazole, this corresponds to a structure determined using nine isotopologues. As a benefit of the numerous isotopologues analyzed in this study, it was possible to carry out this analysis using multiple reference structures: normal 1,3-oxazole, [2-^2^H]-oxazole, [5-^2^H]-oxazole, [2,5-^2^H]-oxazole, and [2,4,5-^2^H]-oxazole. Twenty-seven isotopologues are used across the five reference structures. For those atomic coordinates that were determined multiple times across these reference structures, we calculated the mean coordinate value and its standard deviation. The uncertainty is reported as twice the standard deviation.

## Results and Discussion

### Analysis of Rotational Spectra

The rotational spectrum of oxazole (κ = 0.842) features clear bands of intense *a*- and *b*-type, R-branch transitions and weaker Q-branch transitions, typical of oblate asymmetric tops. As shown in Figure 2, the most intense *a*-type transitions occur near 430 GHz when the spectrum is collected at room temperature. The weaker *b*-type transitions of oxazole have their peak over 100 GHz higher in energy. Both the *a*- and *b*-type transitions maintain strong intensity well beyond 750 GHz (the frequency limit of the current analysis). Figure 3 shows two bands of the normal isotopologue and the heavy-atom substituted oxazole bands at natural abundance. For the normal isotopologue, the R-branch bands appear as sets of four quadruply degenerate transitions: two *a*-type ^*a*^R_0,1_ (*J* = *K*_*a*_ + *K*_*c*_ and *J* + 1 = *K*_*a*_ + *K*_*c*_) and two *b*-type transitions (^*b*^R_1,1_, and ^*b*^R_–1,1_) separated by approximately 2*C* (∼9.8 GHz). Across nearly the entire frequency range, the transitions within the band progress to higher frequency as *K*_*a*_ increases, eventually losing degeneracy and forming a quartet of *R*-branch transitions. Each R-branch band is accompanied by a lower-intensity Q-branch band whose peaks, near the R-branch bandhead, consist of four degenerate transitions (^*a*^Q_0,–1_, ^*a*^Q_2,–1_, ^*b*^Q_–1,1_, and ^*b*^Q_1,–1_). Due to the large value of 2*C* and the relatively high energy of the lowest-energy fundamental state, ν_18_ (609 cm^–1^),^72^ the rotational spectrum is rather sparse. The sparse nature of the spectrum makes assigning the transitions of the heavy-atom isotopologues relatively straightforward, even for [1-^18^O]-oxazole at its natural abundance (0.187%). The majority of the remaining transitions observed in the spectrum of the normal isotopologue have already been assigned to vibrationally excited states of oxazole, but a detailed description of these assignments is beyond the scope of the present work.

**Figure 2:**
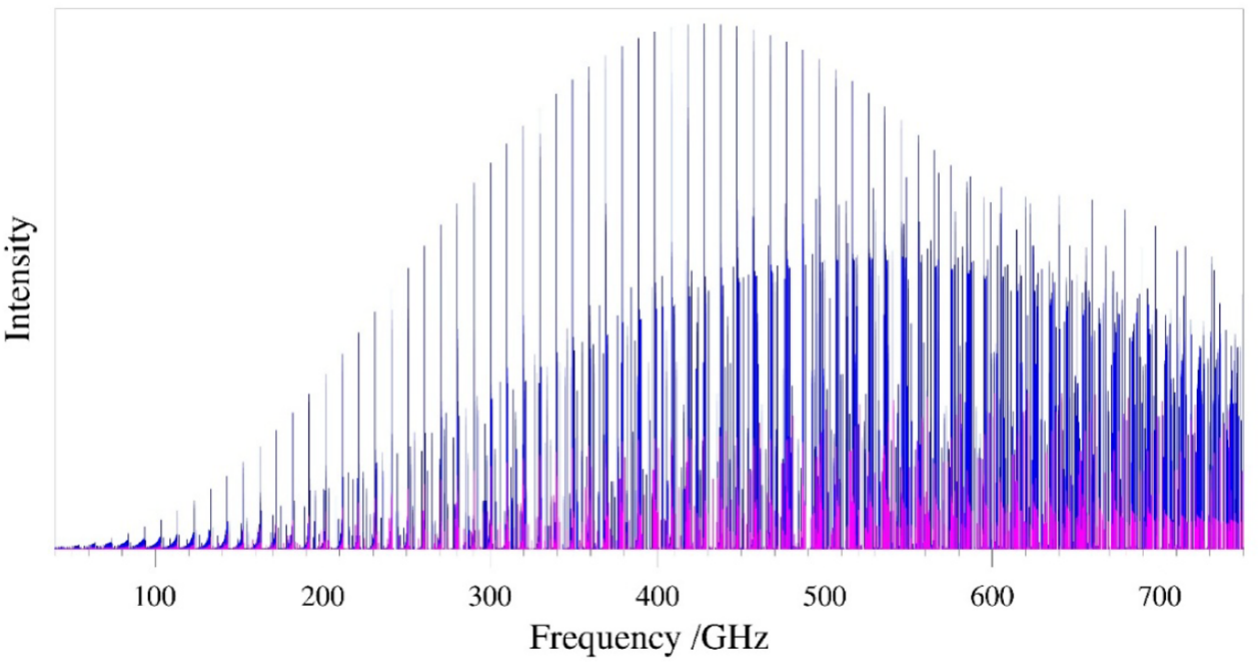
Predicted rotational spectrum of 1,3-oxazole using the experimentally determined spectroscopic constants of the ground vibrational state at 300 K. *a*-Type transitions in blue and *b*-type transitions in magenta.

**Figure 3:**
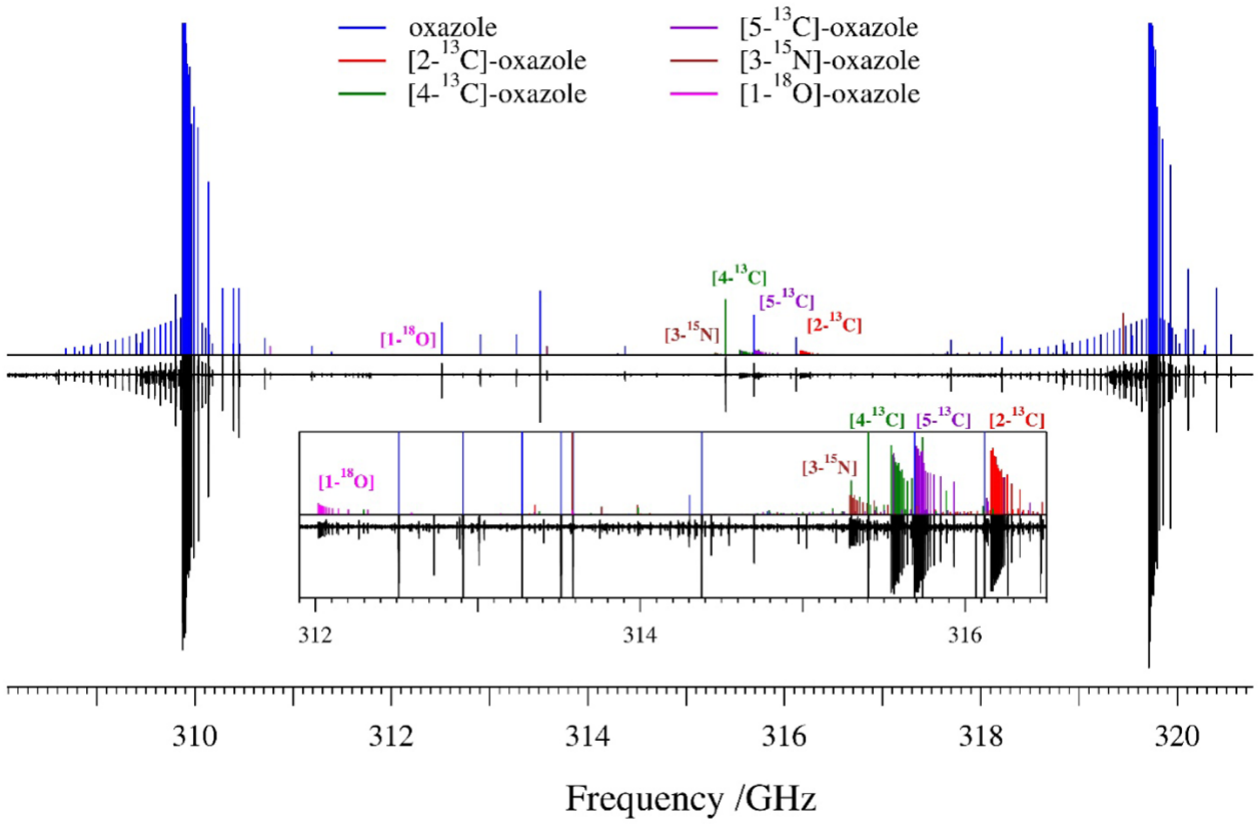
Predicted stick spectra from 308 to 321 GHz (top) and experimental rotational spectrum (bottom) of the normal isotopologue of 1,3-oxazole and its heavy-atom isotopologues. The inset spectrum expands the region from 311.9 to 316.5 GHz to highlight the R-branch bands of the heavy-atom isotopologues. The unassigned spectral features belong to vibrationally excited states of oxazole that will be analyzed in a future work.

Predicted from previous spectroscopic constants,^8^ the rotational transitions for ground-state oxazole were measured, assigned, and least-squares fit to a sextic centrifugally distorted-rotor Hamiltonian. Least-squares fits were completed in the A and S reductions using both the I and III representations in SPFIT. In this work, we generated two least-squares fits for the ground vibrational state of the normal isotopologue: one including nuclear quadrupole coupling and one without. Hyperfine splitting is not resolvable for most of the transitions in the majority of the frequency range observed, so the least-squares fit was initially completed without quadrupole coupling by excluding transitions exhibiting splitting. For thoroughness, a least-squares fit with quadrupole coupling was subsequently carried out including the hyperfine-resolved transitions. The final data set includes nearly 4000 hyperfine-unresolved transitions and just over 5400 total transitions. As shown in the data distribution plot in Figure 4, *J*″ ranges from 0 to 80 and *K*_*a*_^″^ from 0 to 56. The hyperfine-unresolved transitions are least-squares fit equally well in the A reduction I representation, S reduction I representation, and S reduction III representation (complete set of well-determined constants through the sextic level, σ_fit_ = 0.033 MHz; see Supporting Information). In contrast, the A-reduction, III-representation least-squares fit struggles to model the high *K*_*a*_/low *K*_*c*_ transitions and has a substantially higher σ_fit_ value (0.047 MHz) for the same transitions. These observations, taken together with the consistent and effective fitting by the other three models, indicate that the behavior of the A-reduction, III-representation least-squares fit results from a breakdown in the Hamiltonian model rather than an issue with the transition assignments or untreated quadrupole coupling. Although the III representation might seem to be the natural choice for a near-oblate asymmetric top like oxazole, and despite the previous analysis having been conducted using the A reduction, III representation,^8^ our analyses do not employ this methodology. The breakdown of the A reduction using the I representation for near-prolate asymmetric tops or the III representation for near-oblate asymmetric tops is well-known.^13,17,73–78^

**Figure 4:**
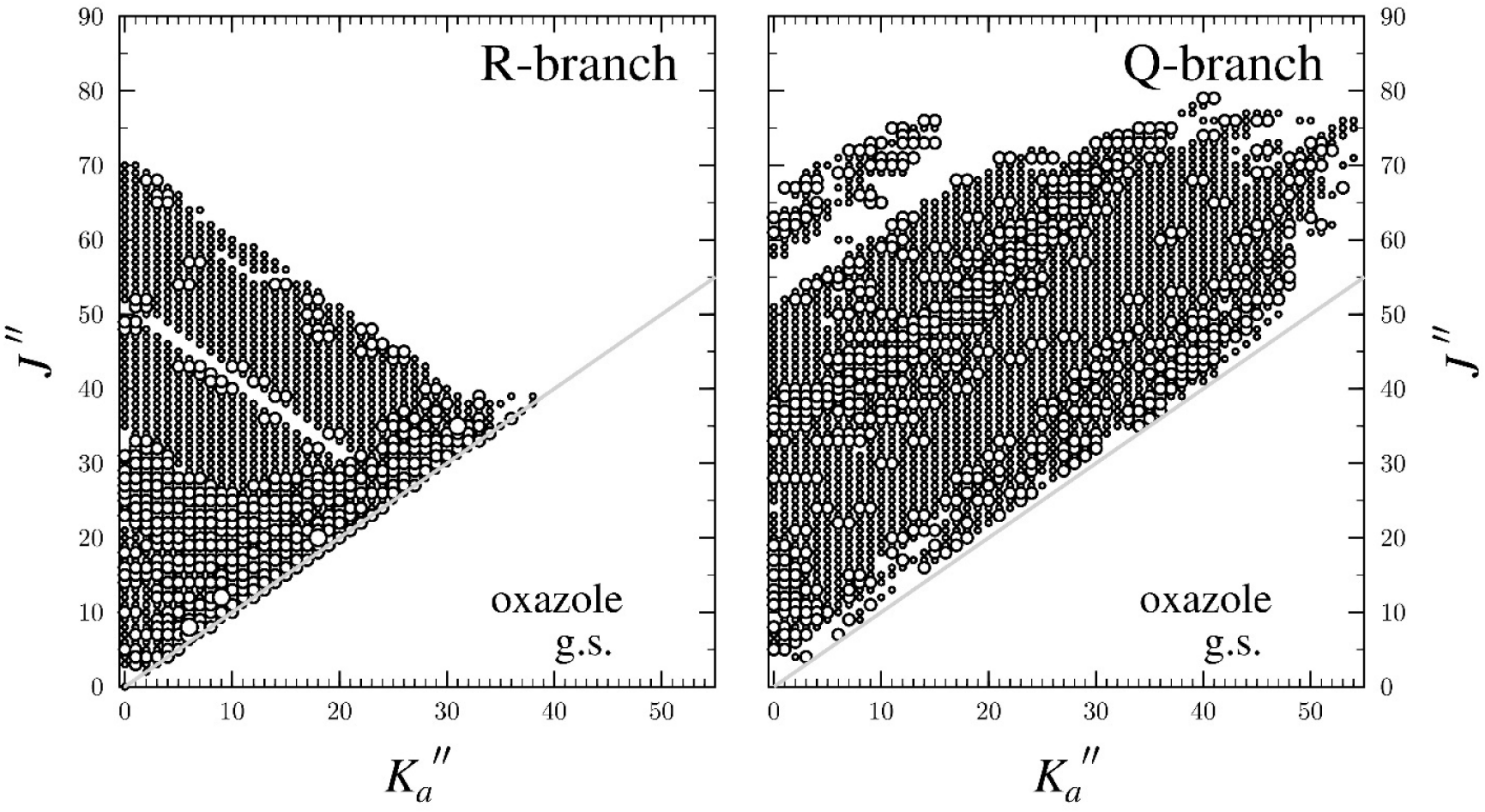
Data distribution plot for the least-squares fit of spectroscopic data for the vibrational ground state of oxazole, normal isotopologue, including hyperfine-resolved transitions. The size of the outlined circle is proportional to the value of |(*f*_obs_ – *f*_calc_)/δ*f*|, where δ*f* is the frequency measurement uncertainty, and all values represented are smaller than 3. Gaps in the plots are due to hardware limitations in measuring the corresponding frequencies.

Spectroscopic constants in the A reduction, I^*r*^ representation and the S reduction, III^*r*^ representation are presented in Table 1. Spectroscopic constants using additional models and comparison to previous determinations are available in Table S1. Agreement between the spectroscopic constants determined by Hegelund et al.^8^ using the A reduction, III^*r*^ representation and those determined in the current work is generally excellent, except for the off-diagonal terms δ_*J*_ and δ_*K*_, where agreement is quite poor (Table S1). This disagreement could be due to the aforementioned breakdown of the Hamiltonian or due to the differences in the amount of spectroscopic information and spectroscopic constants fitted. The computed spectroscopic constants are generally in excellent agreement with their corresponding experimental values. In either reduction, the computed rotational constants are within 0.7% of their experimental values (Table 1). All of the computed A-reduction centrifugal distortion constants are within 8% of their experimental values, with the exception of Δ_*JK*_ (16.2%). The computed S-reduction centrifugal distortion constants are in similar agreement with the experimental values, except for the off-diagonal terms, *d*_1_ (−50%), *h*_1_ (15%), *h*_2_ (−16%), and *h*_3_ (21%). The origin of the comparatively large deviations of Δ_*JK*_ and *d*_1_ is not clear, given the excellent agreement of the other quartic centrifugal distortion constants. Nevertheless, the computed constants at the CCSD­(T)/cc-pCVTZ level of theory would be sufficient to make useful initial predictions of oxazole if experimental spectroscopic constants were not available.

**Table 1:** Spectroscopic Constants for the Ground Vibrational State of Oxazole, Normal Isotopologue (S Reduction, III^*r*^ Representation and A Reduction, I^*r*^ Representation)^a^

S reduction, III^*r*^ representation			A reduction, I^*r*^ representation		
constant	CCSD(T)^b^	experimental	constant	CCSD(T)^b^	experimental
*A*_0_^(S)^ (MHz)	10024​	10050.​995 804 (47)	*A*_0_^(A)^ (MHz)	10024​	10050.​​993 453 (47)
*B*_0_^(S)^ (MHz)	9579​	9645.​​730 520 (45)	*B*_0_^(A)^ (MHz)	9579​	9645.​​733 331 (45)
*C*_0_^(S)^ (MHz)	4895​	4919.​​403 233 (53)	*C*_0_^(A)^ (MHz)	4895​	4919.​​402 831 (53)
					
*D*_*J*_ (kHz)	3.​​68	3.​​759 257 (30)	Δ_*J*_ (kHz)	1.​​97	2.​​​029 928 (23)
*D*_*JK*_ (kHz)	–5.​​76	–5.​​891 699 (48)	Δ_*JK*_ (kHz)	–0.​​435	–0.​​517 793 (41)
*D*_*K*_ (kHz)	2.​​48	2.​​541 538 (31)	Δ_*K*_ (kHz)	2.​​14	2.​​231 667 (45)
*d*_1_ (kHz)	–0.​​034 4	–0.​​023 259 4 (77)	δ_*J*_ (kHz)	0.​​786	0.​​810 418 5 (89)
*d*_2_ (kHz)	0.​​033 3	0.​​030 986 0 (34)	δ_*K*_ (kHz)	1.​​45	1.​​470 053 (20)
					
*H*_*J*_ (Hz)	0.​​001 51	0.​​001 433 4 (51)	Φ_*J*_ (Hz)	0.​​000 685	0.​​000 606 9 (36)
*H*_*JK*_ (Hz)	–0.​​006 51	–0.​​006 560 (10)	Φ_*JK*_ (Hz)	0.​​​001 76	0.​​​001 986 (11)
*H*_*KJ*_ (Hz)	0.​​​008 45	0.​​​008 624 (12)	Φ_*KJ*_ (Hz)	–0.​​​009 70	–0.​​​010 251 (21)
*H*_*K*_ (Hz)	–0.​​​003 46	–0.​​​003 523 0 (63)	Φ_*K*_ (Hz)	0.​​​008 41	0.​​​008 746 (21)
*h*_1_ (Hz)	–0.​​​000 100	–0.​​​000 090 1 (27)	ϕ_*J*_ (Hz)	0.​​​000 345	0.​​​000 316 2 (15)
*h*_2_ (Hz)	–0.​​​000 121	–0.​​​000 136 1 (20)	ϕ_*JK*_ (Hz)	0.​​​001 35	0.​​​001 424 7 (60)
*h*_3_ (Hz)	0.​​​000 045	0.​​​000 050 82 (45)	ϕ_*K*_ (Hz)	0.​​​002 62	0.​​​002 593 6 (65)
χ_*aa*_ (MHz)	–3.​​​934	–5.​​​072 (47)	χ_*aa*_ (MHz)	–3.​​​934	–4.​​​071 (14)
χ_*bb*_ – χ_*cc*_ (MHz)	–0.​​​828	–2.​​​020 (76)	χ_*bb*_ – χ_*cc*_ (MHz)	–0.​​828	–1.​​​027 (40)
χ_*ab*_ (MHz)	1.​​​009	0​^c^	χ_*ab*_ (MHz)	1.​​​009	0​^c^
χ_*aa,J*_ (MHz)		–0.​​​000 093 (13)	χ_*aa,J*_ (MHz)		0.​​​000 880 (87)
χ_*aa,K*_ (MHz)		0.​​​002 08 (15)	χ_*aa,K*_ (MHz)		–0.​​​000 87 (12)
[χ_*bb*_ – χ_*cc*_]_*J*_ (MHz)		[0]	[χ_*bb*_ – χ_*cc*_]_*J*_ (MHz)		0.​​​001 140 (92)
[χ_*bb*_ – χ_*cc*_]_*K*_ (MHz)		[0]	[χ_*bb*_ – χ_*cc*_]_*K*_ (MHz)		–0.​​​002 72 (40)
*N*_lines_ ^d^		5402​	*N*_lines_ ^d^		5402
σ_fit_ (MHz)		0.​​​033	σ_fit_ (MHz)		0.​​​039
κ^e^		0.​​​842 051	κ^e^		0.​​​842 0543
Δ_*i*_ (uÅ^2^)^f^		0.​056 222 (1)	Δ_*i*_ (uÅ^2^)^f^		0.​056 234 (1)

a Left-handed output from SPFIT was converted to right-handed for presentation.

b Evaluated with the cc-pCVTZ basis set. Rotational constants (*B*_0_) are determined from their equilibrium (*B*_e_) values adjusted for vibration–rotation interaction.

c Value was allowed to be fitted, but remained at zero.

d Number of independent transitions.

e κ = (2*B* – *A* – *C*)/(*A* – *C*) calculated from *B*_0_ values using PLANM.

f Inertial defect (Δ_*i*_ *= I*_*c*_ – *I*_*a*_ – *I*_*b*_) calculated from *B*_0_ values using PLANM.

For brevity, only the spectroscopic constants for the singly substituted oxazole isotopologues in the S reduction, III^*r*^ representation are provided in Table 2. All spectroscopic constants are provided in the Supporting Information, alongside data distribution plots. All of the spectroscopic constants are substantially improved compared to those available from the previous works, which included only transitions below 40 GHz.^4–7^ Complete sets of quartic and some sextic centrifugal distortion constants for the heavy-atom and deuterium-substituted isotopologues are determined for the first time. The improvement in both the number and precision of constants determined is due to the greatly increased line counts and broader range of *J* and *K*_*a*_ values of the observed transitions. In the case of the deuterium-substituted isotopologues, the larger number of observed transitions is due to the intentional H/D exchange that led to a much larger partial pressure of the individual species. The H/D exchange reactions also allowed the observation of polydeuterio isotopologues, as well as some of the heavy-atom isotopologues of [2-^2^H]-, [5-^2^H]-, [2,5-^2^H]-, and [2,4,5-^2^H]-oxazoles. In total, the spectroscopic constants were determined for 30 isotopologues, which include multiple isotopic substitutions of each atom with respect to the normal isotopologue. Of the oxazole-*d*_*x*_ isotopologues, only [2,5-^2^H]-oxazole had a sufficient number of observed transitions to allow a complete determination of the centrifugal distortion constants through the sextic level. In all cases where a centrifugal distortion constant could not be satisfactorily determined by least-squares fitting, it was held constant at its CCSD­(T)/cc-pCVTZ value. Rotational constants from all 30 of the isotopologues were used in the subsequent *r*_*e*_^SE^ structure determination.

**Table 2:** Spectroscopic Constants for the Singly Substituted Isotopologues of Oxazole (S Reduction, III*^r^* Representation)^a^ ^,^ ^b^

constant	[2-^13^C]	[4-^13^C]	[5-^13^C]	[3-^15^N]	[1-^18^O]	[2-^2^H]	[4-^2^H]	[5-^2^H]
*A*_0_^(S)^ (MHz)	9835.​​057 79 (27)	9899.​​115 64 (23)	9938.​​119 97 (28)	10042.​​273 02 (40)	10014.​​349 40 (58)	9649.​​659 500 (87)	9801.​​517 2 (10)	9884.​​523 81 (12)
*B*_0_^(S)^ (MHz)	9636.​​714 74 (23)	9539.​​316 17 (19)	9512.​​562 17 (23)	9395.​358 11 (25)	9232.​046 62 (47)	9207.​276 481 (76)	9005.​691 63 (94)	8959.​632 117 (97)
*C*_0_^(S)^ (MHz)	4864.​781 108 (94)	4855.​289 258 (91)	4857.​691 245 (97)	4851.​377 16 (21)	4801.​031 25 (22)	4709.​223 406 (89)	4690.​937 16 (14)	4697.​285 37 (10)
*D*_*J*_ (kHz)	3.​672 93 (17)	3.​672 64 (15)	3.​666 59 (17)	3.​662 12 (25)	3.​616 17 (34)	3.​359 930 (67)	3.​383 49 (26)	3.​356 338 (87)
*D*_*JK*_ (kHz)	–5.​762 00 (29)	–5.​756 57 (25)	–5.​747 34 (27)	–5.​730 95 (66)	–5.​644 28 (54)	–5.​264 449 (79)	–5.​290 25 (39)	–5.​243 43 (12)
*D*_*K*_ (kHz)	2.​488 22 (16)	2.​482 55 (14)	2.​479 83 (15)	2.​468 70 (48)	2.​424 26 (30)	2.​267 241 (61)	2.​268 68 (20)	2.​250 706 (82)
*d*_1_ (kHz)	0.​032 23 (25)	–0.​070 09 (20)	–0.​003 70 (26)	–0.​053 66 (47)	–0.​105 55 (71)	–0.​143 790 (44)	–0.​240 75 (72)	–0.​141 109 (61)
*d*_2_ (kHz)	–0.​002 877 (78)	0.​050 35 (13)	–0.​023 74 (17)	0.​007 13 (20)	0.​040 46 (41)	0.​032 709 (17)	–0.​033 04 (32)	–0.​045 572 (13)
*H*_*J*_ (Hz)	0.​001 261 (52)	0.​001 477 (39)	0.​001 527 (39)	[0.​001 441 4]	0.​001 406 (54)	0.​001 278 (17)	[0.​001 323 7]	0.​001 335 (19)
*H*_*JK*_ (Hz)	–0.​005 94 (13)	–0.​006 55 (10)	–0.​006 32 (11)	–0.​006 08 (31)	[−0.​005 984 2]	–0.​005 517 (15)	[−0.​005 579 5]	–0.​005 834 (26)
*H*_*KJ*_ (Hz)	0.​008 20 (17)	0.​009 06 (16)	0.​008 27 (20)	0.​007 47 (47)	[0.​007 832 3]	0.​007 026 (24)	[0.​007 19]	0.​007 576 (43)
*H*_*K*_ (Hz)	–0.​003 481 (83)	–0.​003 971 (88)	–0.​003 49 (11)	–0.​002 65 (27)	[−0.​003 216 4]	–0.​002 886 (19)	[−0.​002 938 5]	–0.​003 103 (30)
*h*_1_ (Hz)	[−0.​000 053 3]	[−0.​000 103 5]	[−0.​000 071 4]	[−0.​000 083 2]	[−0.​000 09]	[0.​000 222 2]	[0.​000 062 8]	[0.​000 096 6]
*h*_2_ (Hz)	[−0.​000 114 1]	[−0.​000 054]	[−0.​000 046 6]	[−0.​000 076 1]	[−0.​000 050 1]	[−0.​000 032 4]	[0.​000 079 5]	[0.​000 062 7]
*h*_3_ (Hz)	[0.​000 073 1]	[−0.​000 011 5]	[0.​000 038]	[0.​000 047 3]	[−0.​000 004 2]	[−0.​000 015 9]	[0.​000 021]	[−0.​000 012 8]
								
*N*_lines_ ^c^	1057	1157	1077	544	436	2244	295	1645
σ_fit_ (MHz)	0.​036	0.​036	0.​036	0.​039	0.​046	0.​033	0.​041	0.​034
κ^d^	0.​920 188	0.​857 331	0.​832 472	0.​750 750	0.​699 883	0.​820 913	0.​688 558	0.​643 397
Δ_*i*_ (uÅ^2^)^e^	0.​056 707 (3)	0.​056 860 (3)	0.​056 758 (3)	0.​056 830 (5)	0.​057 375 (6)	0.​055 030 (2)	0.​056 152 (9)	0.​055 056 (2)

a Left-handed output from SPFIT was converted to right-handed for presentation.

b Values in square brackets held fixed at the computed value [CCSD­(T)/cc-pCVTZ] in the least-squares fit.

c Number of independent transitions.

d κ = (2*B* – *A* – *C*)/(*A* – *C*) calculated from *B*_0_ values using PLANM.

e Inertial defect (Δ_*i*_ *= I*_*c*_ – *I*_*a*_ – *I*_*b*_) calculated from *B*_0_ values using PLANM.

### Initial Analysis of Semi-Experimental Equilibrium Structure (*r*_*e*_^SE^)

As a consequence of planarity, oxazole possesses two independent moments of inertia. Using 30 isotopologues (60 independent moments of inertia), the 13 unique structural parameters are substantially overdetermined. Rotational constants determined in both A and S reductions (*B*_0_) were converted to determinable constants (*B*_0_^″^) using eqs S1–S6 in the Supporting Information. The agreement between these two sets of parameters (typically within 0.02 MHz) indicates that the rotational and quartic centrifugal distortion constants are likely determined with sufficient accuracy for use in the *r*_*e*_^SE^ structure. The only concerning discrepancies between the *B*_0_^″(S)^ and *B*_0_^″(A)^ rotational constants were for [2,5-^2^H, 1-^18^O]- and [5-^2^H, 3-^15^N]-oxazole, where the *A*_0_^″^ and *B*_0_^″^ values differed by absolute values greater than 0.038 MHz. Such differences indicate that the 251 and 22 transitions available for [2,5-^2^H, 1-^18^O]- and [5-^2^H, 3-^15^N]-oxazole, respectively, were not sufficient to adequately remove the correlations between *A*_0_ and *B*_0_ or enable a satisfactory determination of the centrifugal distortion constants.

For a planar molecule, such as oxazole, the true equilibrium inertial defect (Δ_*ie*_) must be exactly zero. Any deviation from zero may be used as a metric to judge the quality of the experimental *B*_0_^″^ values for each isotopologue, as well as the quality of the computed vibration–rotation interaction and electron-mass corrections. The inertial defects for each isotopologue are presented in Table 3, calculated using four different sets of rotational constants. The Δ_*i* 0_ values are determined from the uncorrected *B*_0_^″^ values and show the small, positive deviations from zero expected for the isotopologues of an aromatic heterocycle.^9–13,15,18,19^ When the *B*_0_^″^ values are corrected for only the vibration–rotation interaction, the resulting Δ_*ie*_ values (second column of values from the left) are reduced by an order of magnitude, but are overcorrected and become negative. Applying both the vibration–rotation interaction and electron-mass corrections to the *B*_0_^″^ values results in very small, slightly positive Δ_*ie*_ values (third column of values from the left). This pattern of decreasing magnitudes of inertial defects with successive corrections is typical. The mean of the fully corrected oxazole inertial defects is 0.00053 uÅ^2^, which is approximately half that observed for many other molecules (∼0.001 uÅ^2^).^9–13,16,18,19^ Although 2*H*-1,2,3-triazole also had an unusually small mean of the fully corrected inertial defect values,^15^ the individual values varied in sign and magnitude. The values for oxazole isotopologues are all positive and fairly similar in magnitude. The [5-^2^H, 3-^15^N]-oxazole isotopologue, however, has a fully corrected Δ_*ie*_ value (0.00088) that falls outside two standard deviations (2σ) from the mean Δ_*ie*_ value of all 30 isotopologues (Table 3). Given the large data set of 30 isotopologues, this outlier may reflect an issue in the semi-experimental equilibrium rotational constants for this isotopologue. The Δ_*ie*_ values, combined with the *B*_0_^″(S)^ and *B*_0_^″(A)^ analysis, indicate that the spectroscopic data and computational corrections are very high quality for the oxazole isotopologues excepting [2,5-^2^H, 1-^18^O]- and [5-^2^H, 3-^15^N]-oxazole (vide supra).

**Table 3:** Inertial Defects (Δ_i_) of Oxazole Isotopologues from Various Determinations of the Moments of Inertia

isotopologue	Δ_*i*0_ (uÅ^2^)	Δ_*i*e_ (uÅ^2^)^a^	Δ_*i*e_ (uÅ^2^)^b^	Δ_*i*e_ (uÅ^2^)^c^
C_3_H_3_NO	0.​05622	–0.​00753	0.​00070	0.​00130
[2-^13^C]	0.​05671	–0.​00759	0.​00065	0.​00130
[4-^13^C]	0.​05686	–0.​00746	0.​00077	0.​00134
[5-^13^C]	0.​05677	–0.​00767	0.​00057	0.​00130
[3-^15^N]	0.​05683	–0.​00755	0.​00069	0.​00131
[1-^18^O]	0.​05725	–0.​00756	0.​00067	0.​00122
				
[2-^2^H]	0.​05503	–0.​00763	0.​00061	0.​00126
[2-^2^H, 2-^13^C]	0.​05538	–0.​00764	0.​00060	0.​00125
[2-^2^H, 4-^13^C]	0.​05561	–0.​00776	0.​00048	0.​00124
[2-^2^H, 5-^13^C]	0.​05552	–0.​00760	0.​00064	0.​00128
[2-^2^H, 3-^15^N]	0.​05567	–0.​00757	0.​00067	0.​00132
[2-^2^H, 1-^18^O]	0.​05608	–0.​00791	0.​00033	0.​00118
				
[4-^2^H]	0.​05615	–0.​00769	0.​00055	0.​00134
				
[5-^2^H]	0.​05506	–0.​00777	0.​00047	0.​00130
[5-^2^H, 2-^13^C]	0.​05535	–0.​00792	0.​00032	0.​00115
[5-^2^H, 4-^13^C]	0.​05572	–0.​00775	0.​00049	0.​00132
[5-^2^H, 5-^13^C]	0.​05526	–0.​00794	0.​00030	0.​00113
**[5-^2^H, 3-^15^N]^d^**	**0.​05600**	**–0.​00736**	**0.​00088**	**0.​00169**
				
[2,4-^2^H]	0.​05478	–0.​00793	0.​00031	0.​00125
[2,5-^2^H]	0.​05373	–0.​00777	0.​00047	0.​00127
[2,5-^2^H, 2-^13^C]	0.​05408	–0.​00773	0.​00051	0.​00129
[2,5-^2^H, 4-^13^C]	0.​05434	–0.​00774	0.​00050	0.​00127
[2,5-^2^H, 5-^13^C]	0.​05409	–0.​00776	0.​00048	0.​00128
[2,5-^2^H, 3-^15^N]	0.​05432	–0.​00778	0.​00046	0.​00130
**[2,5-^2^H, 1-^18^O]^d^**	**0.​05478**	**–0.​00784**	**0.​00040**	**0.​00123**
**[4,5-^2^H]^*d*^**	**0.​05515**	**–0.​00776**	**0.​00048**	**0.​00133**
				
[2,4,5-^2^H]	0.​05365	–0.​00776	0.​00048	0.​00128
[2,4,5-^2^H, 2-^13^C]	0.​05380	–0.​00794	0.​00030	0.​00110
[2,4,5-^2^H, 4-^13^C]	0.​05414	–0.​00779	0.​00045	0.​00130
[2,4,5-^2^H, 5-^13^C]	0.​05407	–0.​00768	0.​00056	0.​00134
Average (*x̅*)	0.​05528	–0.​00771	0.​00053	0.​00128
Std.​ Dev.​ (*s*)	0.​00105	0.​00014	0.​00014	0.​00010

a Vibration–rotation interaction corrections only. Corrections computed at the CCSD­(T)/cc-pCVTZ geometry.

b Vibration–rotation interaction and electron-mass corrections. Corrections computed at the CCSD­(T)/cc-pCVTZ geometry.

c Vibration–rotation interaction corrections calculated at the *r*_*e*_^SE^(initial) geometry; electron-mass corrections computed at the CCSD­(T)/cc-pCVTZ geometry.

d Isotopologue with *A*_0_^″^ and *B*_0_^″^ constants excluded from *r*_*e*_^SE^ structure determination, also highlighted in bold font.

Similar to the recent work on thiophene,^18^ an *xrefiteration* analysis was performed in the process of determining the *r*_*e*_^SE^ structure. This routine, described in greater detail elsewhere,^12^ determines an *r*_*e*_^SE^ structure from the core set of the normal and the single-substitution isotopologues. It then sequentially incorporates the isotopologue that most decreases the overall uncertainty of the structural parameters into the *r*_*e*_^SE^ structure. Consequently, isotopologues that increase the uncertainty of the structural parameters are added at the end of the analysis and are identified for further scrutiny. The *xrefiteration* plot of total uncertainty in the oxazole structure using all rotational constants of all 30 isotopologues is provided in Figure 5 (black plot). A plot showing the total uncertainty, the bond-distance uncertainty, and the uncertainty in the angles is included in the Supporting Information, but the latter quantities largely follow the pattern of the total uncertainty plot. The impact of including each isotopologue beyond the core set on the individual structural parameters is illustrated in Figure 6 (left-hand side; bond distances) and Figure 7 (left-hand-side; angles). Consistent with the plot in Figure 5, the individual plots in Figures 6 and 7 show that the structure undergoes significant changes throughout the entire process of incorporating isotopologues into the least-squares fit. The extent of these changes—to all the parameters and with many isotopologues in the data set—differs notably from the behavior observed in previous structure determinations^9–19^ and is cause for concern regarding the precision and accuracy of this initially determined *r*_*e*_^SE^ structure.

**Figure 5:**
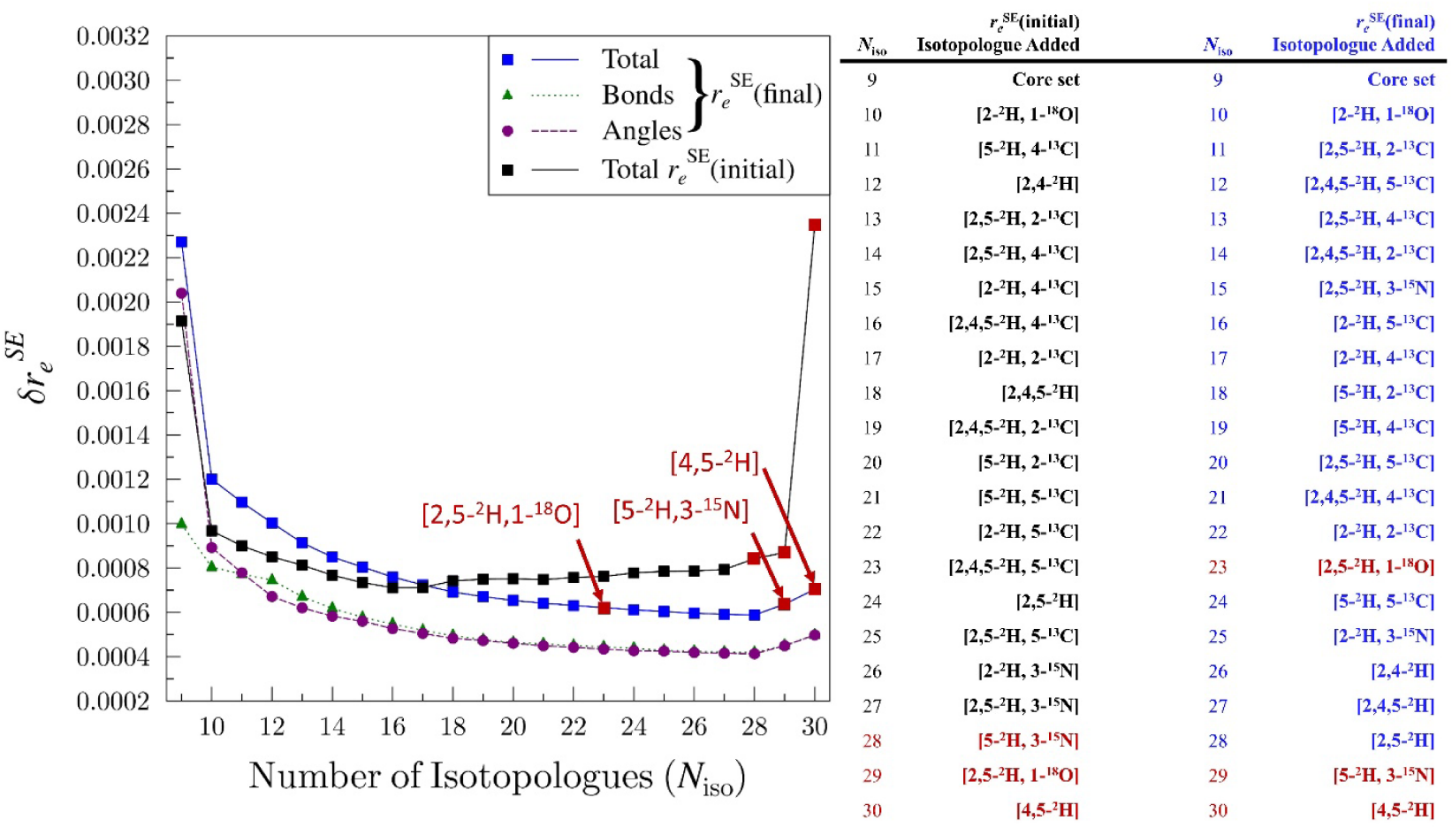
Plot of structural 2σ parameter uncertainty values (δ*r*_*e*_^SE^) as a function of the number of isotopologues (*N*_iso_) incorporated in the structure determination. The total uncertainty (δ*r*_*e*_^SE^ Total) for the *r*_*e*_^SE^ (final) structure (blue squares) and *r*_*e*_^SE^ (initial) structure (black squares) is derived using all three rotational constants for all 30 isotopologues. The bond distance uncertainty (δ*r*_*e*_^SE^ Bonds, green triangles) and the uncertainty in the angles (δ*r*_*e*_^SE^ Angles, purple circles) are also presented for *r*_*e*_^SE^ (final). Maroon squares and labels correspond to isotopologues for which only *C*_0_^″^ was included in the isotopologue data set for the recommended *r*_*e*_^SE^ structure determination.

**Figure 6:**
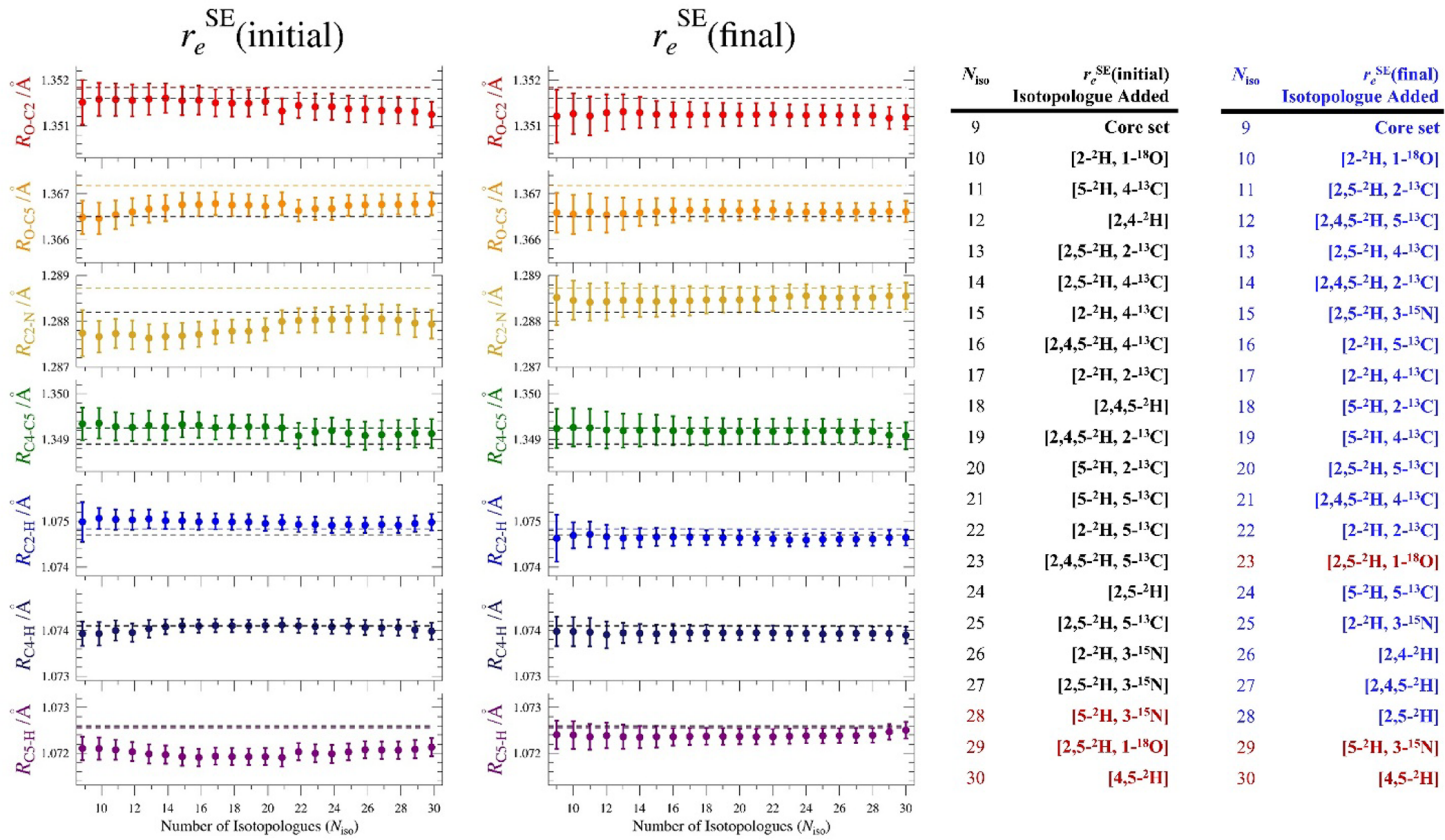
Plots of *r*_*e*_^SE^(initial) (left) and *r*_*e*_^SE^(final) (right) bond distances as a function of the number of isotopologues (*N*_iso_) and their 2σ uncertainties using all three rotational constants for all 30 isotopologues. Consistent scales are used for each distance (0.0020 Å). Colored dashed line—BTE value calculated for that parameter. Black dashed line—*r*_*s*_^SE^ value (using 27 isotopologues) for that parameter. Isotopologue ordering along the *x*-axis is provided in the tables on the right.

**Figure 7:**
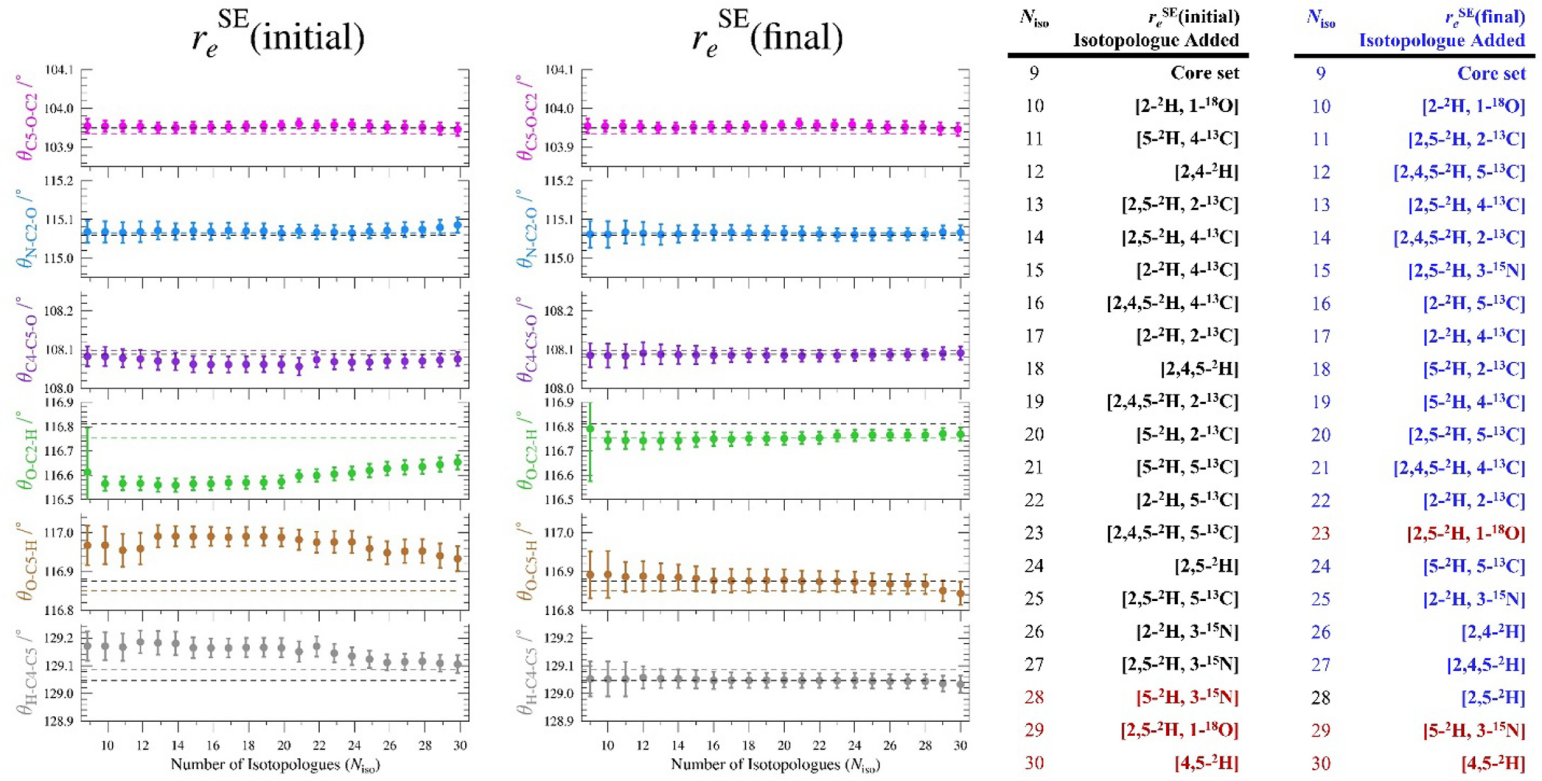
Plots of *r*_*e*_^SE^(initial) (left) and *r*_*e*_^SE^(final) (right) bond angles as a function of the number of isotopologues (*N*_iso_) and their 2σ uncertainties using all three rotational constants for all 30 isotopologues. Scales for θ_C5–O–C2_, θ_N–C2–O_, and θ_C4–C5–O_ are consistent (0.25°); those for θ_O–C2–H_, θ_O–C5–H_, and θ_H–C4–C5_ are adjusted to show both initial and final values within the plots. Colored dashed line—BTE value calculated for that parameter. Black dashed line—*r_s_* ^SE^ value (using 27 isotopologues) for that parameter. Isotopologue ordering along the *x*-axis is provided in the tables on the right.

Inspection of the *xrefiteration* plot and the *xrefiteration* parameter convergence plots (Figures 5 (black plot), 6—left-hand side, and 7—left-hand side) validates the aforementioned concerns regarding [2,5-^2^H, 1-^18^O]- and [5-^2^H, 3-^15^N]-oxazole and reveals an obvious problem with the inclusion of [4,5-^2^H]-oxazole, which substantially increases the uncertainty of the structural parameters. This behavior of [4,5-^2^H]-oxazole was initially puzzling, in light of the unremarkable Δ_*ie*_ value (Table 3) and good agreement between the *B*_0_^″(S)^ and *B*_0_^″(A)^ values (Table S31). Nevertheless, given the detrimental effect of [4,5-^2^H]-oxazole on the structure, and the aforementioned concerns regarding [2,5-^2^H, 1-^18^O]- and [5-^2^H, 3-^15^N]-oxazole, we determined a semi-experimental structure excluding *A*_0_^″^ and *B*_0_^″^ for these three isotopologues.

In addition to consideration of inertial defects and *xrefiteration* plots, the isotopologue data was assessed by consideration of rotational constants and their residuals. In this analysis, we compare the equilibrium rotational constant for each isotopologue at the *r*_*e*_^SE^ structure (*B*_*e*_^*xrefit*^) with the corresponding experimental rotational constant (*B*_0_^″^) that has been adjusted by application of computed corrections for vibration–rotation interaction and electron mass to afford a semi-experimental equilibrium rotational constant (*B*_*e*_^SE^). (It is important to note that the designation “SE” does not imply that these constants are derived from the *r*_*e*_^SE^ structure.) In general, there is excellent agreement between the *xrefit*-predicted and semi-experimental values (most residuals within ±0.3 MHz; Table 4). For [4,5-^2^H]-oxazole, however, the residuals of *A*_*e*_ and *B*_*e*_ are an order-of-magnitude larger than those for any other isotopologue (−2.25 MHz for *A*_*e*_ and 2.18 MHz for *B*_*e*_; Table 4). The magnitudes of the residuals are much larger than the electron-mass corrections (all are <0.65 MHz), which excludes these corrections as the source of the discrepancy. Considering that the rotational constants, themselves, appear to be good quality, it seems likely that the vibration–rotation interaction constants for this isotopologue are the source of the error. (For reference, the magnitudes of the vibration–rotation interaction constants for *A*_*e*_ and *B*_*e*_ fall in the range 60–85 MHz.)

**Table 4:** Rotational Constant Residuals (*B_e_^xrefit^* – *B*_*e*_^SE^) from the *r*_*e*_^SE^ Structure of Oxazole^a^ and Principal Axis Rotations Relative to the Normal Isotopologue (θ)^b^ ^,^ ^c^

isotopologue	*A*_*e*,residual_ (MHz)	*B*_*e*,residual_ (MHz)	*C*_*e*,residual_ (MHz)	θ*r*_*e*_ (deg)	θ*r*_*e*_^SE^ (deg)	θ*r*_*e*_ – θ*r*_*e*_^SE^ (deg)
C_3_H_3_NO​	–0.​​02	–0.​02	0.​02	0.​00	0.​00	0.​00
[2-^13^C]​	–0.​22	0.​25	0.​04	–6.​82	–8.​40	1.​58
[4-^13^C]​	–0.​10	0.​02	0.​02	17.​10	15.​51	1.​59
[5-^13^C]​	–0.​22	0.​09	0.​00	–15.​72	–17.​09	1.​38
[3-^15^N]​	–0.​03	–0.​03	0.​02	–5.​29	–5.​52	0.​24
[1-^18^O]​	–0.​10	–0.​05	–0.​01	11.​69	12.​35	–0.​66
[2-^2^H]​	–0.​01	–0.​04	0.​02	–62.​26	–61.​77	–0.​50
[2-^2^H, 2-^13^C]​	–0.​03	0.​04	0.​03	–62.​63	–62.​26	0.​16
[2-^2^H, 4-^13^C]​	–0.​02	–0.​03	0.​01	–48.​26	–48.​87	0.​61
[2-^2^H, 5-^13^C]​	–0.​12	–0.​02	–0.​01	–71.​87	–72.​63	–0.​96
[2-^2^H, 3-^15^N]​	–0.​19	0.​11	0.​01	–64.​29	–67.​26	2.​98
[2-^2^H, 1-^18^O]​	–0.​40	0.​32	0.​00	–18.​68	–23.​84	5.​15
[4-^2^H]​	–0.​13	0.​08	0.​02	–8.​07	–9.​35	1.​28
[5-^2^H]​	–0.​08	0.​04	0.​01	–32.​95	–33.​88	0.​93
[5-^2^H, 2-^13^C]​	0.​11	–0.​05	0.​03	–39.​46	–40.​46	1.​00
[5-^2^H, 4-^13^C]​	–0.​13	0.​06	0.​01	–32.​65	–33.​97	1.​32
[5-^2^H, 5-^13^C]​	0.​06	–0.​14	–0.​01	–36.​25	–37.​03	0.​78
**[5-^2^H, 3-^15^N]​^c^**	**–0.​47**	**0.​27**	**0.​01**	**–29.​04**	**–29.​73**	**0.​68**
[2,4-^2^H]​	–0.​06	0.​03	0.​01	–33.​44	–34.​16	0.​72
[2,5-^2^H]​	–0.​10	0.​03	0.​01	–57.​12	–57.​89	0.​77
[2,5-^2^H, 2-^13^C]​	0.​03	–0.​03	0.​02	–60.​70	–61.​30	0.​60
[2,5-^2^H, 4-^13^C]​	0.​05	–0.​11	0.​00	–62.​94	–63.​71	0.​77
[2,5-^2^H, 5-^13^C]​	–0.​14	–0.​01	–0.​01	–56.​22	–56.​84	0.​62
[2,5-^2^H, 3-^15^N]​	–0.​25	0.​14	0.​00	–50.​24	–51.​09	0.​86
**[2,5-^2^H, 1-^18^O]​^c^**	**–0.​44**	**0.​28**	**–0.​01**	**–50.​58**	**–52.​07**	**1.​50**
**[4,5-^2^H]​^c^**	**–2.​25**	**2.​18**	**0.​01**	**–15.​89**	**–28.​42**	**12.​54**
[2,4,5-^2^H]​	–0.​03	–0.​04	0.​00	–60.​58	–60.​37	–0.​21
[2,4,5-^2^H, 2-^13^C]​	–0.​05	0.​05	0.​01	–61.​14	–60.​96	–0.​19
[2,4,5-^2^H, 4-^13^C]​	0.​06	–0.​12	0.​00	–53.​08	–53.​21	0.​13
[2,4,5-^2^H, 5-^13^C]​	–0.​14	–0.​02	–0.​02	–67.​36	–66.​89	–0.​47

a Calculated at *r*_*e*_^SE^(initial) geometry.

b Calculation of the isotopologue rotation angles θ*r*_*e*_ and θ*r*_*e*_^SE^ is explained in the Supporting Information and by Demaison et al.^20^

c Isotopologue with *A*_0_^″^ and *B*_0_^″^ constants excluded from *r*_*e*_^SE^ structure determination, also highlighted in bold font.

Demaison et al.^20^ previously demonstrated that evaluating the vibration–rotation interaction terms at the computed geometry (*r*_*e*_) rather than the actual *r*_*e*_^SE^ geometry can limit the accuracy and precision of the *r*_*e*_^SE^ structure determination.^20^ Specifically, they suggested that the vibration–rotation interaction corrections for other isotopologues may be expressed in terms of the vibration–rotation interaction values of the normal isotopologue corrected for the angle of rotation relative to the normal isotopologue, i.e., the angle between the principal axes of the normal and other isotopologue. A difference in this rotation between the semi-experimental (θ*r*_*e*_^SE^) and computed structures (θ*r*_*e*_) can cause inaccurate vibration–rotation interaction constant values, and the authors describe that the error is exacerbated if the relative angle of rotation for an isotopologue is near 45°, approaching the symmetric top limit where *A*_*e*_ = *B*_*e*_. Isotopologues suffering this discrepancy may exhibit large differences between the semi-experimental and structure-based rotational constants (residuals), and these *A*_*e*_ and *B*_*e*_ residuals are likely to be opposite in sign and similar in magnitude. Although there is not a definitive limit where the rotation of principal axes is sufficiently close to 45° (or 135°) to negatively impact the semi-experimental structure determination, the authors indicated individual isotopologues of several small molecules in their respective structure determinations for which this rotation was problematic: the five-membered aromatic heterocycles furazan ([3,4-^2^H, 3-^13^C], θ*r*_*e*_^SE^ = 20.1°),^20^ furan ([2-^13^C], θ*r*_*e*_^SE^ = 39.9),^20^ and 1,3,4-oxadiazole ([^13^C], θ*r*_*e*_^SE^ = 122.3).^20,21^

To explore this issue for 1,3-oxazole, the angle of rotation for each isotopologue relative to the normal was calculated for the CCSD­(T)/cc-pCVTZ computed equilibrium (θ*r*_*e*_) and semi-experimental equilibrium (θ*r*_*e*_^SE^) geometries (Table 4). Figure 8 depicts the compilation of rotation angles for the *r*_*e*_^SE^ geometry. Expanding upon the observations and conclusions of Demaison et al.,^20,21^ we note that the rotation of an isotopologue’s principal axes near 45° relative to the normal isotopologue is not the primary cause of issues in the *r*_*e*_^SE^ structure determination of oxazole. The most problematic isotopologue—[4,5-^2^H]-oxazole—has a smaller value of θ*r*_*e*_^SE^ than the majority of other isotopologues and its value (θ*r*_*e*_^SE^ = −28.42°) is not especially close to −45°. Of the several isotopologues with θ*r*_*e*_^SE^ values closer to 45° rotation than [4,5-^2^H]-oxazole, none of these isotopologues suffers the error in residual values (Table 4) or negatively impacts the structure in the same way (Figures 5–7). Although [4,5-^2^H]-oxazole has relatively modest rotations of the principal axes (θ*r*_*e*_ = −15.89° and θ*r*_*e*_^SE^ = −28.42°), the *discrepancy* between these angles (12.54°; Table 4) is uniquely large among the 30 isotopologues in the data set. That is, there is a large differential rotation of the principal axes of [4,5-^2^H]-oxazole in the *r*_*e*_ and *r*_*e*_^SE^ structures. The implication of this result is that the vibration–rotation interaction corrections for this isotopologue have been evaluated at a geometry (*r*_*e*_) that is nontrivially different than the structure determined by the least-squares fit (*r*_*e*_^SE^).

**Figure 8:**
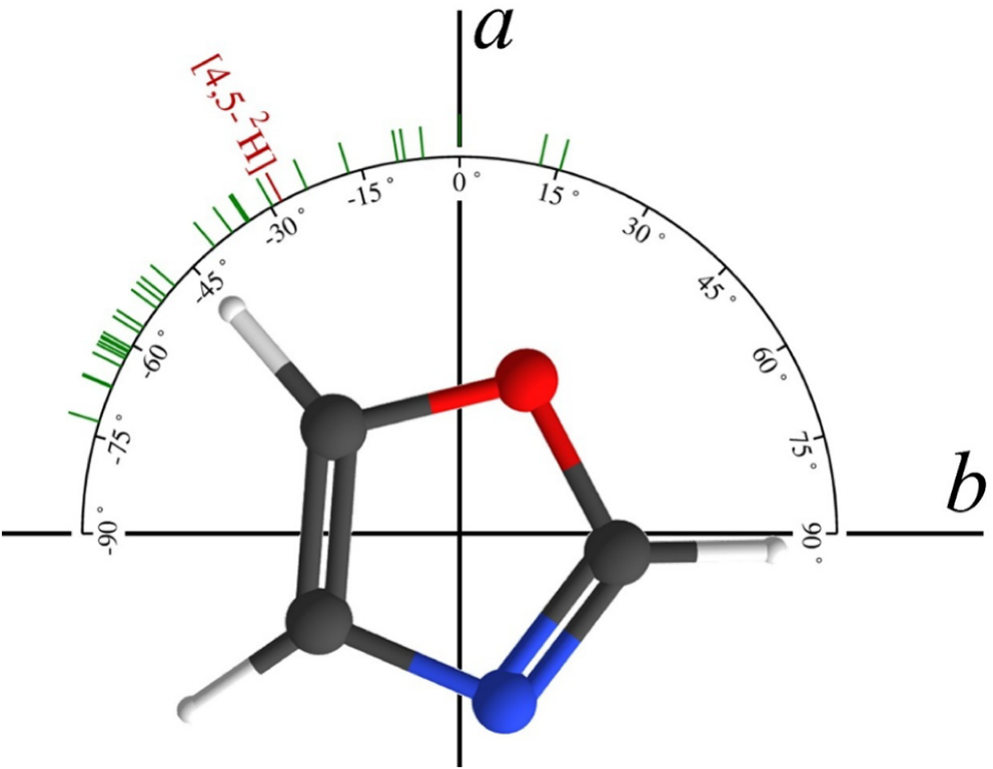
Normal isotopologue of 1,3-oxazole with principal inertial axes. The green dashes indicate the direction of the *a*-axis for each of the 30 isotopologues in the *r*_*e*_^SE^ geometry. The value of angular rotation corresponds to θ*r*_*e*_^SE^ (deg). [4,5-^2^H]-Oxazole is highlighted in maroon for convenience. The center of mass for each set of axes is shifted slightly (not displayed), but this shift does not affect the relative angles.

As noted previously, the *a*- and *b*-axis vibration–rotation interaction corrections in a planar molecule are not entirely independent of, and have a somewhat compensatory effect toward, one another.^20^ As a result, poorly determined corrections can be masked in the calculation of the inertial defect value (Δ_*i*_ *= I*_*c*_ – *I*_*a*_ – *I*_*b*_). In the subsequent analysis, a generalized solution to the problem of differential rotation is proposed. In brief, consideration of the θ value for each isotopologue and an *xrefiteration* analysis (if possible) of the data set are warranted in structure determinations where isotopologues have unexpectedly large residuals. Both of these analyses, combined with analysis of the inertial defects or second moments, can help identify poor computed corrections and problematic experimental constants for isotopologues included in the structure determination. A more complete description of the θ analysis, the equations employed, and an example calculation are available in the Supporting Information.

### Addressing the Differential Isotopologue Rotation Effect on Structure Determination

The vibration–rotation interaction constants are heavily dependent on the cubic force constants and the atomic coordinates of the molecule.^79,80^ Despite the fact that the new anharmonic calculation is not evaluated at a stationary point on the computed potential energy surface, we computed a new set of vibration–rotation interaction constants at the geometry of the initial semi-experimental structure (*r*_*e*_^SE^(initial)) to investigate the possibility that using a more accurate molecular geometry could improve the vibration–rotation interaction corrections. Specifically, we used the *r*_*e*_^SE^(initial) structure excluding *A*_0_^″^ and *B*_0_^″^ for the [2,5-^2^H, 1-^18^O]-, [5-^2^H, 3-^15^N]-, and [4,5-^2^H]- isotopologues as input for the new vibration–rotation interaction constants. The resultant vibration–rotation interaction corrections (provided in the Table S35) were used to determine a new *r*_*e*_^SE^(final) geometry. The magnitudes of the [4,5-^2^H]-oxazole residuals for *A*_*e*_ and *B*_*e*_ were reduced from −2.25 and 2.18 MHz (*B*_*e*_^*xrefit*^ – *B*_*e*_^SE^(initial); Table 4) to −0.42 and 0.29 MHz (*B_e_^xrefit^* – *B*_*e*_^SE^(final); Table S36), respectively. Since the vibration–rotation interaction corrections are the only changes to the *xrefit* input, the changes to the residual values are a direct reflection of the improvement in the vibration–rotation interaction corrections.

The fully corrected Δ_*ie*_ values for the isotopologues with the updated corrections are provided in Table 3 (rightmost column). The average Δ_*ie*_ value increases by a factor of ∼2.4 after the new corrections are applied, but the absolute magnitude is still quite small. At the same time, the standard deviation of the Δ_*ie*_ values decreases. This opposite behavior of the average and standard deviation of the inertial defects may indicate that the original vibration–rotation interaction corrections computed at the *r*_*e*_ geometry were too large in magnitude and produced fortuitously low Δ_*ie*_ values. The smaller standard deviation of the residual Δ_*ie*_ values in the *r*_*e*_^SE^(final) data set may suggest that the larger values of Δ_*ie*_(final) may be more accurate, thereby implying that further improvement toward the ideal value of zero is possible, e.g., by the inclusion of higher-order γ corrections to the vibration–rotation interaction.^81–82^

The *xrefiteration* plot for *r*_*e*_^SE^(final) (Figure 5; blue plot) depicts a single implementation of the strategy to improve the vibration–rotation interaction corrections. (The data set for this plot includes all three rotational constants for all 30 isotopologues for direct comparability to the *r*_*e*_^SE^(initial) plot). The dramatic improvement in the behavior of [4,5-^2^H]-oxazole is the most notable change, relative to the *r*_*e*_^SE^(initial) plot (Figure 5; black plot). (Separate *r*_*e*_^SE^(initial) and *r*_*e*_^SE^(final) plots are provided in a stacked format in the Supporting Information.) The sequence in which the isotopologues appear in the *xrefiteration* analysis for *r*_*e*_^SE^(final) is rather different than that for *r*_*e*_^SE^(initial) (Figure 5). Of note, [2,5-^2^H, 1-^18^O]-oxazole was incorporated second-to-last (29th) in the initial *xrefiteration* analysis and caused a modest increase in the total uncertainty of the structure; using the vibration–rotation interaction corrections computed at the *r*_*e*_^SE^(initial) structure, it is incorporated 23rd and causes a slight decrease in the total uncertainty. Another notable change in the *xrefiteration* plot for *r*_*e*_^SE^(final) is the smooth, continuous change in the center region of the plot (*N*_iso_ = 16–27), which contrasts with the behavior of the *r*_*e*_^SE^(initial) plot. This behavior is mirrored in the individual parameter plots in Figures 6 and 7 (right-hand side). Each of the isotopologues displays much smoother incorporation into the *r*_*e*_^SE^(final) structure than those in the analogous plot of the *r*_*e*_^SE^(initial) structure determination. Importantly, none of those isotopologues had notably large *r*_*e*_^SE^(initial) residuals, inertial defects, poorly determined rotational constants, or other concerning characteristics. The improved continuity and convergence of the plots reflect a superior quality of the *r*_*e*_^SE^(final) data set. The lowest overall uncertainty achieved in the *r*_*e*_^SE^(initial) and *r*_*e*_^SE^(final) *xrefiteration* analyses differs by only ∼0.0001. In the *r*_*e*_^SE^(final) analysis, the core set of isotopologues has a slightly greater uncertainty than in the *r*_*e*_^SE^(initial) analysis (0.0023 vs 0.0019; Figure 5), indicating that this strategy may not have universally improved the corrections for all isotopologues.

Despite the substantial improvement in the ultimate total statistical uncertainty (δ*r*_*e*_^SE^(initial) = 0.0023; δ*r*_*e*_^SE^(final) = 0.0007; Figure 5) and the values of many parameters moving toward their BTE values, the *r*_*e*_ BTE values of five of the 13 parameters of oxazole still fall slightly outside the 2σ limit of their corresponding *r*_*e*_^SE^(final) parameters (Figures 6 and 7; Table 5). This behavior contrasts sharply with that of other five-membered rings, such as 1*H*-1,2,3-, 2*H*-1,2,3-, and 1*H*-1,2,4-triazole, whose *r*_*e*_ BTE and *r*_*e*_^SE^ structures were obtained in the conventional manner, without using *r*_*e*_^SE^-based vibration–rotation interaction corrections.^13,15^ The *r*_*e*_^SE^ structure of oxazole is better determined than the structures of all of the triazoles, i.e., the largest bond-distance uncertainty in oxazole is 0.0002 Å, while the smallest uncertainty in the triazole bond distances is 0.0003 Å, with some as large as 0.0010 Å. Thus, the apparent larger discrepancy between theory and experiment for oxazole relative to previous works is likely due to the relatively small 2σ values of the oxazole *r*_*e*_^SE^(final) parameters and is not indicative of more poorly predicted *r*_*e*_ BTE parameters. Importantly, it appears that each of the oxazole *r*_*e*_^SE^(final) structural parameters is well determined and nearly converged using 30 isotopologues (Figures 6 and 7), approaching a limit in which additional isotopologues are unlikely to improve the structure. Similar observations have been made in other recent *r*_*e*_^SE^ structure determinations, most notably in the case of thiophene, which incorporated 46 isotopologues in the analysis.^18^ This limit is consistent with a breakdown in the assumption that the *r*_*e*_ or *r*_*e*_^SE^ geometry of all isotopologues is the same or with the idea that higher-order corrections, such as the aforementioned γ corrections,^81–82^ are needed to the vibration–rotation interactions. The isotopologues of concern that contribute only *C*_0_^″^ values to the recommended structure determination ([2,5-^2^H, 1-^18^O]-, [5-^2^H, 3-^15^N]-, and [4,5-^2^H]-oxazole) do not seem to have a dramatic impact on the *r*_*e*_^SE^ parameters even when all three constants are included.

**Table 5:** Experimental and Computational Structural Parameters of Oxazole

	*r*_*e*_^SE^(initial) CCSD(T)/cc-pCVTZ	*r*_*e*_^SE^(final) CCSD(T)/cc-pCVTZ (recommended)	CCSD(T) BTE	CCSD(T)/cc-pCV5Z
*N*_isotopologues_	30​	30​		
*N*_*I*_ ^a^	57​	57​		
*R*_O–C2_ (Å)	1.​3512 (3)	1.​3512 (2)	1.​3518	1.​3502
*R*_O–C5_ (Å)	1.​3668 (2)	1.​3667 (2)	1.​3672	1.​3661
*R*_C2–N_ (Å)	1.​2879 (3)	1.​2885 (2)	1.​2887	1.​2884
*R*_C4–C5_ (Å)	1.​3491 (3)	1.​3491 (2)	1.​3493	1.​3491
*R*_N–C4_ (Å)	1.​3925 (3)^b^	1.​3924 (2)^b^	1.​3926^c^	1.​3921^c^
*R*_C2–H_ (Å)	1.​0750 (2)	1.​0746 (1)	1.​0748	1.​0748
*R*_C4–H_ (Å)	1.​0740 (2)	1.​0740 (1)	1.​0741	1.​0740
*R*_C5–H_ (Å)	1.​0721 (2)	1.​0724 (2)	1.​0726	1.​0725
				
θ_C5–O–C2_ (deg)	103.​946 (17)	103.​955 (13)	103.​935	103.​998
θ_N–C2–O_ (deg)	115.​085 (20)	115.​067 (15)	115.​065	115.​062
θ_C4–C5–O_ (deg)	108.​076 (18)	108.​088 (14)	108.​097	108.​065
θ_C2–N–C4_ (deg)	103.​868 (21)^b^	103.​865 (16)^b^	103.​873^c^	103.​858^c^
θ_N–C4–C5_ (deg)	109.​024 (19)^b^	109.​025 (15)^b^	109.​031^c^	109.​017^c^
θ_O–C2–H_ (deg)	116.​654 (30)	116.​758 (23)	116.​752	116.​786
θ_O–C5–H_ (deg)	116.​933 (32)	116.​864 (25)	116.​850	116.​902
θ_C5–C4–H_ (deg)	129.​105 (32)	129.​045 (25)	129.​086	129.​020

a Number of independent moments of inertia.

b Values in italics are dependent parameters determined in an alternate *Z*-matrix.

c Values are determined from other parameters using mathematical relationships.

A second iteration of this *r*_*e*_^SE^-based vibration–rotation interaction constant approach resulted in a further, slight reduction in the remaining residuals (see the Supporting Information). Overall, however, the impact of the second iteration was not nearly as meaningful as the first iteration and did not noticeably improve the structure. This outcome is attributed to the very small differences between θ*r*_*e*_^SE^(final) and θ*r*_*e*_^SE^(final, second iteration) values for each isotopologue (all <0.03°).

The *r*_*e*_^SE^(initial) and *r*_*e*_^SE^(final) parameters of oxazole using all moments of inertia from the 30 isotopologues (except those derived from *A*_0_^″^ and *B*_0_^″^ for [2,5-^2^H, 1-^18^O]-, [5-^2^H, 3-^15^N]-oxazole, and [4,5-^2^H]-oxazole) are presented in Figure 9 and Table 5. In this analysis, the 13 independent structural parameters are determined from 57 independent moments of inertia. The highly accurate and precise *r*_*e*_^SE^(final) structure determined from these data is comparable to those for other molecules determined using the same methodology, with the exception that the *r*_*e*_^SE^-based vibration–rotation interaction corrections were not included in those works.^9–19^ All of the bond distances are determined with 2σ uncertainties of 0.0001 Å or 0.0002 Å, which is the limit of precision for bond lengths determined by *r*_*e*_^SE^ methodology in prototypical organic molecules.^16,17^ The bond angles are also well determined, with the largest 2σ uncertainties equal to 0.032° and also similar to those other works. Given the reliability of the *r*_*e*_ BTE structures in previous works,^9–18^ it appears that the new method for determining the *r*_*e*_^SE^(final) is validated by having more parameters in closer agreement with the *r*_*e*_ BTE structure than did the *r*_*e*_^SE^(initial) structure. Some structural parameters appear unaffected by the use of *r*_*e*_^SE^-based vibration–rotation interaction corrections (*R*_O–C2_), while others change by more than the original 2σ uncertainty (*R*_C2–N_). The parameters with the largest relative change between the initial and final structures involve C2. The exact contribution of each isotopologue to the observed effects, however, is not readily discernible, so no conclusions can be drawn.

**Figure 9:**
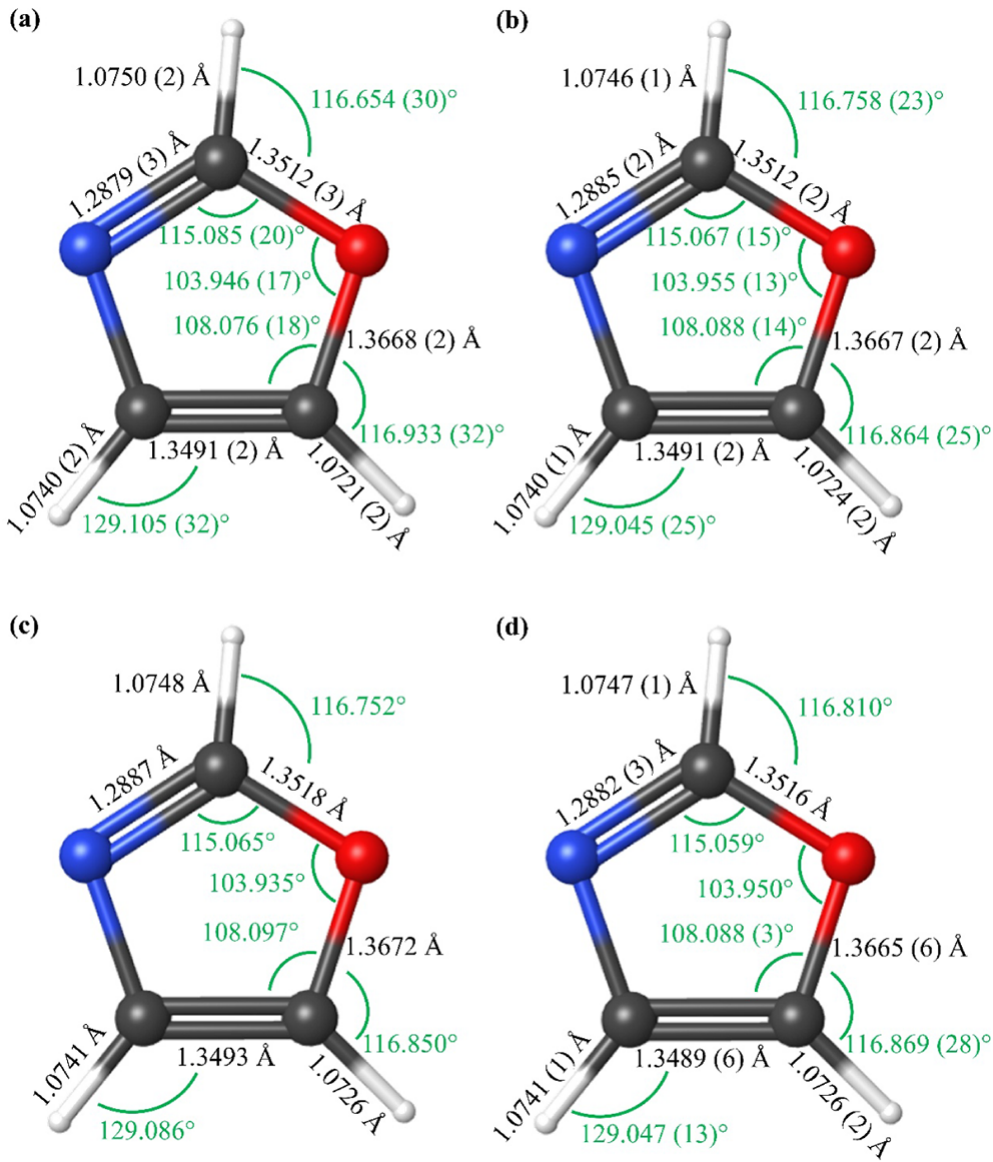
Semi-experimental equilibrium (*r*_*e*_^SE^), theoretical equilibrium (*r_e_* BTE), and semi-experimental substitution (*r_s_* ^SE^) structures of oxazole. Uncertainties shown are 2σ and all corrections are calculated at the CCSD­(T)/cc-pCVTZ level. (a) *r*_*e*_^SE^(initial) structure with vibration–rotation interaction corrections at the CCSD­(T)/cc-pCVTZ geometry. (b) *r*_*e*_^SE^(final) structure with vibration–rotation interaction corrections at the *r*_*e*_^SE^(initial) geometry. (c) *r_e_* BTE structure. (d) Semi-experimental substitution structure (*r_s_* ^SE^) using 27 isotopologues with vibration–rotation interaction corrections at the *r*_*e*_^SE^(initial) geometry. Estimation of *r_s_* ^SE^ uncertainties is discussed in the Supporting Information.

### Comparing Theoretical and Semi-Experimental Equilibrium Structures

Figure 10 provides a direct comparison of the *r*_*e*_ CCSD­(T)/cc-pCV*X*Z (*X* = 3, 4, or 5) and *r*_*e*_ BTE theoretical structural parameters of oxazole to their *r*_*e*_^SE^(initial) and *r*_*e*_^SE^(final) values. These plots make it clear that the CCSD­(T)/cc-pCVTZ *r*_*e*_ structural parameters—reflecting the geometry at which the vibration–rotation interaction constants were initially evaluated—are distinctly different from the structural parameters of *r*_*e*_^SE^(initial) and *r*_*e*_^SE^(final). At the level of precision that is now achievable with *r*_*e*_^SE^ structure determinations, these small structural differences matter, in terms of the appropriate evaluation of the vibration–rotation interaction terms. Increasing the size of the basis set from cc-pCVTZ to cc-pCVQZ and cc-pCV5Z establishes that the computed values are converging. The three *R*_C–H_ parameters and the θ_O–C5–H_ parameter have converged. The other structural parameters appear to be nearly converged at the CCSD­(T)/cc-pCV5Z level and further increasing the basis set to the CCSD­(T)/cc-pCV6Z level is expected to move the resulting *r*_*e*_ BTE parameter toward the *r*_*e*_^SE^(final) values for all parameters except θ_C5–C4–H_. The same is not true for the *r*_*e*_^SE^(initial) parameter values. This observation provides further support for the assertion that the improvement of the vibration–rotation interaction corrections used in the *r*_*e*_^SE^(final) structure ultimately affords more accurate structural parameters.

**Figure 10:**
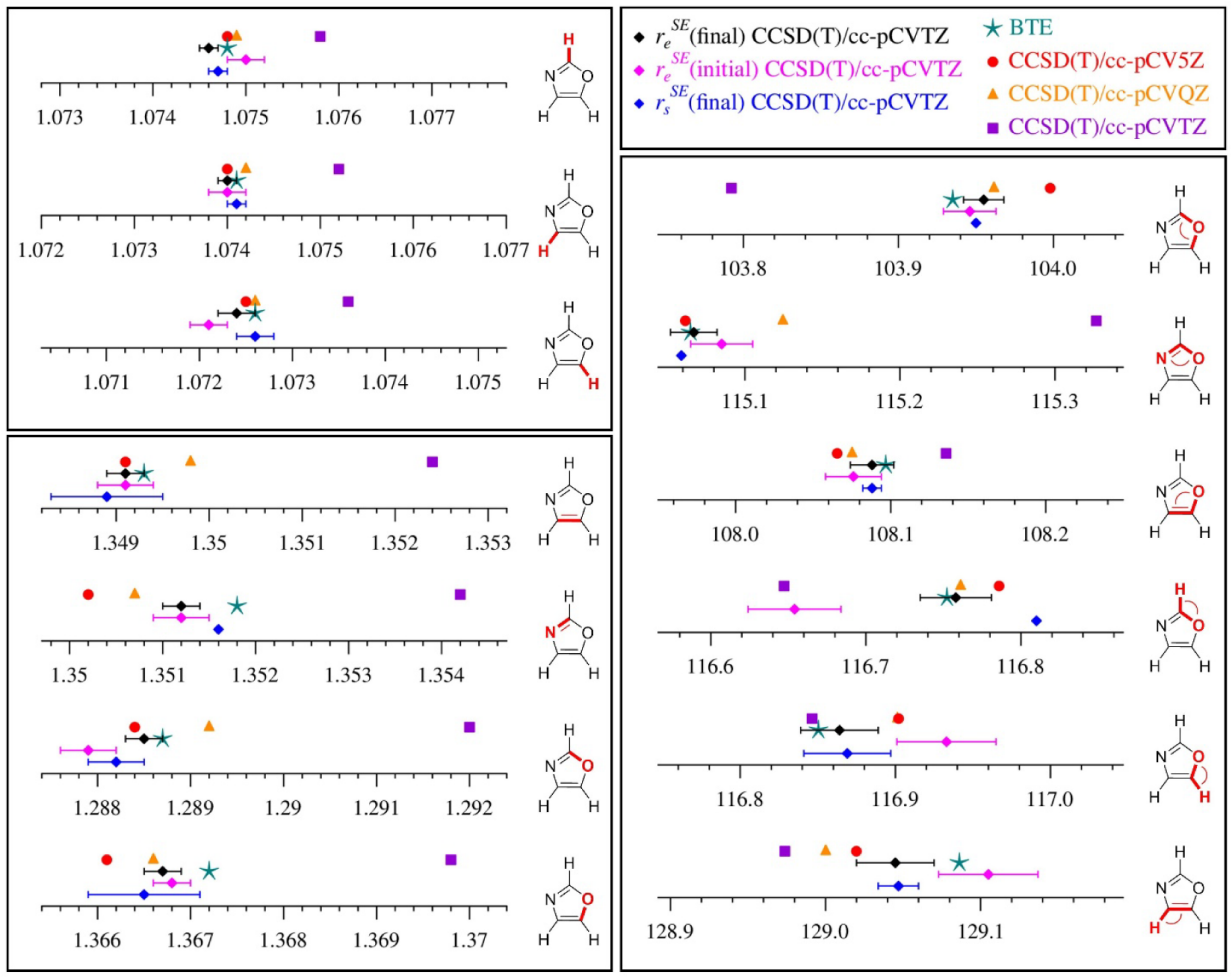
Graphical comparison of oxazole structural parameters with bond distances in Ångstrom (Å) and angles in deg (°). Uncertainties shown are 2σ. CCSD­(T)/cc-pCVQZ marker for θ_O–C5–H_ is not observable due to overlap with CCSD­(T)/cc-pCV5Z marker. Semi-experimental substitution (*r*_*s*_^SE^) structure is derived from 27 isotopologues and uses *r*_*e*_^SE^(initial)-based vibration–rotation interaction corrections.

### Assessing the Semi-Experimental Substitution (*r_s_* ^SE^) Structure of Oxazole

It is instructive to compare alternate methods of analyzing and interpreting structural data determined by rotational spectroscopy. Substitution structures (*r*_s_) have been a mainstay in structure determination for decades.^70,71,83^ Rather than determining a semi-experimental equilibrium structure (*r*_*e*_^SE^) by least-squares fitting the moments of inertia, substitution structures (*r*_*s*_) use Kraitchman’s equations to determine the Cartesian coordinates of a substituted atom in the principal-axis system of the reference isotopologue. Stiefvater and coworkers determined the substitution structure of oxazole using the observable *B*_0_ constants,^7^ affording a structure that is not directly comparable to *r*_*e*_ or *r*_*e*_^SE^ structures because the observable constants include effects of vibration–rotation coupling. Semi-experimental substitution structures (*r*_*s*_^SE^), however, where the rotational constants are corrected for the vibration–rotation interactions (and sometimes for the electron-mass distribution), are directly comparable to *r*_*e*_ and *r*_*e*_^SE^ structures. There have been a few reports of substitution structures from semi-experimental equilibrium constants corrected for the vibration–rotation interaction constants, e.g., isocyanic acid,^84^ ketene,^85^ and hydridotrioxygen (HO_3_).^86^ A complete *r*_*s*_ or *r*_*s*_^SE^ structure of a molecule requires a complete set of single-substitution isotopologues, which often represents more than the minimum number of inertial moments that could determine a complete structure. The data from these same isotopologues also could be used to determine an *r*_*e*_^SE^ structure. The primary advantage of *r*_*s*_^SE^ structures is that they can be used to determine individual molecular parameters without the need to hold unfit parameters at a constant value, in contrast to the least-squares fits used to determine *r*_*e*_^SE^ structures. Holding a parameter at a constant value inevitably impacts the values of the other parameters that are able to be determined, limiting their accuracy and precision in an *r*_*e*_^SE^ structure determination. Such a partial-*r*_*s*_^SE^ structure strategy has been employed for several molecules where no hydrogen atoms were isotopically substituted, e.g., 2-ethylfuran^87^ and 2,5-difluorophenol.^88^

Table 6 displays the structural parameters of several oxazole *r*_*s*_^SE^ structures, using 9 or 27 isotopologues and using the initial or final vibration–rotation interaction corrections. As with the *r*_*e*_^SE^ structure determinations, nine isotopologues constitute the “core set” of single-substitution isotopologues: the minimum data set for a complete substitution structure determination. The 27 oxazole isotopologues from this work (excluding [2,5-^2^H, 1-^18^O]-, [5-^2^H, 3-^15^N]-, and [4,5-^2^H]-oxazole), meanwhile, provide four additional reference structures. Between the benefit of multiple reference structures and the limitation of the well-established near-axis problem in substitution structure determination, the parameters for the 27-isotopologue structures could be calculated using one to five reference structures. Details of the *r*_*s*_^SE^ structure determination and the estimates of the statistical uncertainties are provided in the Supporting Information. Of the four semi-experimental substitution structures (*r*_*s*_^SE^) that we determined (Table 6), the *r*_*s*_^SE^(initial) structure using nine isotopologues (using the normal isotopologue as the reference structure) shows the poorest agreement with the *r*_*e*_^SE^(final) structure. Even in this case, however, most of the structural parameters are quite close to their *r*_*e*_^SE^(final) values, and some even fall within the very small uncertainties of those parameters. This observation suggests that when limited data sets are available, determination of the parameters by Kraitchman’s analysis using *B*_*e*_ constants is a viable alternative. When all available reference structures utilizing 27 isotopologues are used in the *r*_*s*_^SE^(initial) structure determination, the *r*_*s*_^SE^ structural parameters show good agreement with the *r*_*e*_^SE^(final) parameters. Likewise, the *r*_*s*_^SE^(final) structures (from either 9 or 27 isotopologues), with vibration–rotation interaction corrections based on the *r*_*e*_^SE^(initial) geometry, show good agreement with the *r*_*e*_^SE^(final) structure. For visual comparison, the *r*_*s*_^SE^ structural parameters from 27 isotopologues are displayed in Figures 6, 7, and 10. The observation that the *r*_*s*_^SE^(final) structures with *r*_*e*_^SE^-based vibration–rotation interaction corrections more closely resemble the *r*_*e*_^SE^ and *r*_*e*_ BTE structures serves as further evidence that the *r*_*e*_^SE^-based vibration–rotation corrections are physically meaningful, and that the improved agreement between *r*_*e*_^SE^(final) and *r*_*e*_ BTE is not simply a case of confirmation bias.

**Table 6:** Semi-Experimental Substitution, Semi-Experimental Equilibrium, and BTE Structural Parameters of Oxazole

	*r*_*s*_^SE^(initial)^a^	*r*_*s*_^SE^(initial)^a^	*r*_*s*_^SE^(final)^b^	*r*_*s*_^SE^(final)^b^	*r*_*e*_^SE^(final) (recommended)	CCSD(T) BTE
*N*_isotopologues_	9​^c^	27​^c^	9​^c^	27​^c^	30​	
*N*_*I*_ ^d^	18​	54​	18​	54​	57	
*R*_O–C2_ (Å)	1.​3536	1.​3536	1.​3516	1.​3516	1.​3512 (2)	1.​3518
*R*_O–C5_ (Å)	1.​3656	1.​3668 (34)	1.​3663	1.​3665 (6)	1.​3667 (2)	1.​3672
*R*_C2–N_ (Å)	1.​2859	1.​2871 (34)	1.​2881	1.​2882 (3)	1.​2885 (2)	1.​2887
*R*_C4–C5_ (Å)	1.​3499	1.​3477 (59)	1.​3491	1.​3489 (6)	1.​3491 (2)	1.​3493
*R*_N–C4_ (Å)	1.​3921	1.​3925 (11)	1.​3922	1.​3923 (4)	1.​3924 (2)^e^	1.​3931^f^
*R*_C2–H_ (Å)	1.​0752	1.​0748 (6)	1.​0746	1.​0747 (1)	1.​0746 (1)	1.​0748
*R*_C4–H_ (Å)	1.​0741	1.​0740 (1)	1.​0741	1.​0741 (1)	1.​0740 (1)	1.​0741
*R*_C5–H_ (Å)	1.​0722	1.​0724 (3)	1.​0725	1.​0726 (2)	1.​0724 (2)	1.​0726
θ_C5–O–C2_ (deg)	103.​918	103.​918	103.​950	103.​950	103.​955 (13)	103.​935
θ_N–C2–O_ (deg)	115.​059	115.​059	115.​059	115.​059	115.​067 (15)	115.​065
θ_C4–C5–O_ (deg)	108.​083	108.​075 (20)	108.​090	108.​088 (6)	108.​088 (14)	108.​097
θ_C2–N–C4_ (deg)	103.​936	103.​904 (93)	103.​878	103.​875 (8)	103.​865 (16)^e^	103.​873^f^
θ_N–C4–C5_ (deg)	109.​004	109.​016 (27)	109.​024	109.​028 (27)	109.​025 (15)^e^	109.​031^f^
θ_O–C2–H_ (deg)	116.​468	116.​468	116.​810	116.​810	116.​758 (23)	116.​752
θ_O–C5–H_ (deg)	116.​969	116.​950 (56)	116.​879	116.​869 (28)	116.​864 (25)	116.​850
θ_C5–C4–H_ (deg)	129.​137	129.​097 (71)	129.​052	129.​047 (13)	129.​045 (25)	129.​086

a Vibration–rotation interaction and electron-mass corrections computed at the CCSD­(T)/cc-pCVTZ geometry.

b Vibration–rotation interaction corrections calculated at the *r*_*e*_^SE^(initial) geometry, electron-mass corrections at the CCSD­(T)/cc-pCVTZ geometry. Statistical uncertainties are twice the standard deviation of the values determined from each reference structure. Where only a single reference structure is available, no statistical uncertainty is provided.

c Determined from either the normal isotopologue and the single isotopic substitution isotopologues (9) or from all reliable isotopologues (27). See Supporting Information for details.

d Number of independent moments of inertia.

e Value in italics is a dependent parameter determined in an alternate *Z*-matrix.

f Value determined from other parameters using mathematical relationships.

### Comparing Semi-Experimental Equilibrium Structures of Oxazole and Thiazole

The improved agreement observed between the *r*_*e*_ BTE and *r*_*e*_^SE^(final) structures of oxazole, relative to the corresponding structures of the sulfur-containing analogue, thiazole,^10^ is achieved using only five more isotopologues for oxazole than for thiazole and with smaller uncertainties in the parameters of oxazole than those of thiazole. The *xrefiteration* plots of oxazole *r*_*e*_^SE^(final) and thiazole *r*_*e*_^SE^(initial) are qualitatively similar. Additionally, the heavy sulfur atom of thiazole prevents a substantial rotation of principal axes of its isotopologues, in contrast to those of oxazole. It therefore seems unlikely that differential rotation is the culprit in the poorer *r*_*e*_^SE^-BTE agreement of thiazole, supporting the notion suggested in that work that improvements to the BTE or to the vibration–rotation interaction constants (through higher-order terms) are necessary to bring the semi-experimental and computed equilibrium structures into agreement.

## Conclusion

We provide the first highly precise and accurate *r*_*e*_^SE^ structure of 1,3-oxazole (*c*-C_3_H_3_NO) with CCSD­(T) corrections for the vibration–rotation interaction and electron-mass distribution corrections to the rotational constants of 30 isotopologues. Though the structural parameters were well-determined after least-squares fitting the core set of 9 isotopologues, the statistical uncertainty of the parameters continued to decrease with the inclusion of each additional isotopologue until nearly the last isotopologue. The statistical uncertainty of the bond distances approached a lower limit of 0.0001 to 0.0002 Å, providing further support for the identified limit of interatomic distance determination for molecules including C–H bonds.

To achieve the most accurate and precise structure, we find it useful to ensure that the least-squares fits for available isotopologues are good quality, that there is good agreement between the determinable constants computed from the A and S reductions, and that the inertial defects or planar second moments are sensible and do not provide cause for concern. In this work, we present a case where it is also valuable to examine the rotation of principal axes of each isotopologue relative to the normal isotopologue in the computed *r*_*e*_ geometry and the semi-experimental equilibrium (*r*_*e*_^SE^) geometry. The analysis of the rotational spectra from many isotopologues of oxazole revealed that one of the oxazole-*d*_2_ isotopologues suffers a complication, of a type previously identified, involving the substantial differential rotation of the principal axis system between the *r*_*e*_ and *r*_*e*_^SE^ structures. Previous remedies for this issue have been limited to excluding the rotational constants of the impacted isotopologues or using only isotopologues that have the same principal axis systems as the normal isotopologue by symmetry. With the benefit of 30 isotopologues in the current study of oxazole, we were able to observe for the first time cases where principal axis rotations close to 45° did not manifest the issue. This observation provides strong evidence that the underlying problem is caused by the difference between the *r*_*e*_ and *r*_*e*_^SE^ geometries and how that discrepancy impacts the calculated corrections for vibration–rotation coupling. Because it is not always practical or desirable to exclude data in a structure determination, we attempted to improve the *r*_*e*_^SE^ structure and address the differential isotopologue rotation problem. The structural parameters of an initial semi-experimental structure determination, *r*_*e*_^SE^(initial), which excluded problematic isotopologues, were used to complete a second CCSD­(T)/cc-pCVTZ anharmonic frequency calculation, providing vibration–rotation interaction corrections using a geometry that is closer to the semi-experimentally determined one.

The physical basis for the seemingly aberrant behavior of some isotopologues derives from the sensitivity of the computed vibration–rotation interaction corrections to atomic coordinates and to cubic force constants. Small changes in the geometry at which the vibration–rotation constants are evaluated (CCSD­(T)/cc-pCVTZ geometry vs *r*_*e*_^SE^(initial) geometry) afforded a change in the α_*A*_ vibration–rotation constant of ca. 2 MHz and a subsequent change in the *A*_*e*_^SE^ rotational constant of 2.58 MHz for the [4,5-^2^H]-isotopologue of oxazole. Smaller, but still consequential, changes are noted for most other isotopologues. The updated vibration–rotation constants introduce an uncertainty associated with the fact that they are not evaluated at a stationary point on the computed potential energy surface. For any particular isotopologue, the updated constants might either improve or degrade the total uncertainty (δ*r*_*e*_^SE^) of the *r*_*e*_^SE^ structure. What is clear is that updated constants, applied to all 30 isotopologues in the current data set for 1,3-oxazole, dramatically decrease the total uncertainty (δ*r*_*e*_^SE^) of the *r*_*e*_^SE^ structure. Although additional studies will be needed to determine whether use of an *r*_*e*_^SE^(initial) input geometry for the calculation of the vibration–rotation interaction constants is a generally applicable technique, it seems reasonable to expect that there is a balance between the benefit of more accurate structural coordinates vs the uncertainty of vibration–rotation interaction constants computed at a nonstationary point.

In a complementary analysis, we obtained a similarly precise and accurate semi-experimental substitution structure (*r*_*s*_^SE^) for 1,3-oxazole from the same rotational constants, allowing a direct comparison of two types of highly precise semi-experimental structures for the first time. Both methods require the best possible values for vibration–rotation interaction constants. Although both methods initially utilize computed values of these constants, the *r*_*e*_^SE^ methodology affords a pathway to improve the values of these corrections. The *r*_*s*_^SE^ methodology does not. Without the *r*_*e*_^SE^ structure to evaluate updated vibration–rotation interaction corrections (a best-case scenario), it would be difficult for the *r*_*s*_^SE^ structure to approach the accuracy and precision of the *r*_*e*_^SE^(final) structure. Without access to an *r*_*e*_^SE^ structure, the *r*_*s*_^SE^ methodology would require improvements in computational methods in order to provide more accurate values for the vibration–rotation interaction constants. CCSD­(T) optimizations with very large basis sets would be required for the molecule of interest in order to provide vibration–rotation interaction corrections to an *r*_*s*_^SE^ structure. This analysis does suggest, however, that high-quality *r*_*s*_^SE^ structures or *r*_*s*_^SE^ partial structures may be accessible if computational advances allow it. At this time, however, the best structure determinations remain from the *r*_*e*_^SE^ method using as many isotopologues as are available and the best possible vibration–rotation interaction and electron-mass corrections.

## Supplementary Material





## Notes

Equations used for calculating determinable constants and BTE corrections; tables of spectroscopic constants; data distribution plots for all isotopologues; additional plots and figures mentioned in the text; experimental details for isotopologue synthesis and characterization (PDF)
Least-squares fitting files; computational output files; *xrefit* files; *xrefiteration* files; output files used to compute the BTE (ZIP)

## #

Deceased March 21, 2025
