# Supplementary material for: Improved Accuracy in Semi-Experimental Structure Determination by Resolving Problems Associated with Rotation of Principal Inertial Axes of Isotopologues: Structures of 1,3-Oxazole (c‑C3H3NO)
Source: J Phys Chem A. 2026 Apr 24;130(18):3623–41. doi: 10.1021/acs.jpca.6c01535 (PMC13158917; doi:10.1021/acs.jpca.6c01535)
Supplement: Supplementary file 1 [file jp6c01535_si_001.pdf]

Supporting Information

Improved Accuracy in Semi-Experimental Structure Determination by Resolving Problems Associated with Rotation of Principal Inertial Axes of Isotopologues: Structures of 1,3-Oxazole (*c*-C_3H_3NO)

Brian J. Esselman,[1] Maria A. Zdanovskaia,[1] Madeleine G. Atwood,[1] Taylor K. Adkins,[1]

Manamu Kobayashi,[2] Shozo Tsunekawa,[2] Kaori Kobayashi,[2]

Nitai P. Sahoo,[3] John F. Stanton,[3,†] R. Claude Woods,[1,*] Robert J. McMahon[1,*]

[1] *Department of Chemistry, University of Wisconsin–Madison, Madison, Wisconsin 53706, United States*

[2] *Department of Physics, University of Toyama, 3190 Gofuku, Toyama 930-8555, Japan*

[3] *Quantum Theory Project, Departments of Physics and Chemistry, University of Florida, Gainesville, Florida 32611, United States*

† Deceased March 21, 2025

* corresponding authors
E-mail address: rcwoods@wisc.edu (R.C. Woods).
E-mail address: robert.mcmahon@wisc.edu (R.J. McMahon).

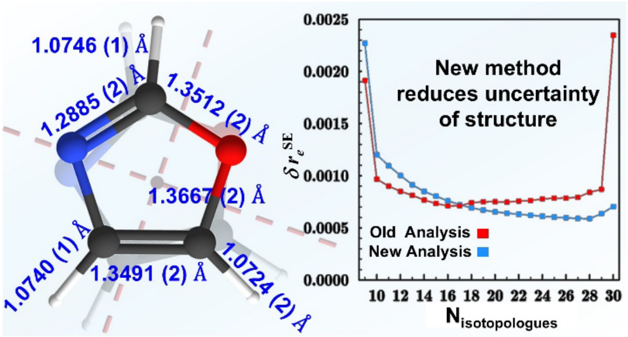

Table of Contents		Page
**Eq. S1 – S6**.	Equations for calculating determinable constants	S7
**Eq. S7 – S13.**	Equations for Calculating the Best Theoretical Estimate (BTE)	S7 - S8
**Table S0.**	Corrections (ΔR) to Applied to Computed Structural Parameters (CCSD(T)/cc-pCV5Z) to Afford the Best Theoretical Estimate (BTE) for Oxazole	S9
Isotopologue numbering scheme for convenience in examining output files		S10
**Overview: tables of spectroscopic constants / figures of data distribution plots**		S11
**Table S1**.	Experimental and computed spectroscopic constants for the normal isotopologue of oxazole	S11 – S13
**Figure S1**.	Data distribution plot for the least-squares fit of millimeter-wave spectroscopic data for the normal isotopologue of oxazole	S14
**Table S2**.	Experimental and computed spectroscopic constants for [2-^{13}C]-oxazole	S15
**Figure S2**.	Data distribution plot for the least-squares fit of millimeter-wave spectroscopic data for [2-^{13}C]-oxazole	S16
**Table S3**.	Experimental and computed spectroscopic constants for [4-^{13}C]-oxazole	S17
**Figure S3**.	Data distribution plot for the least-squares fit of millimeter-wave spectroscopic data for [4-^{13}C]-oxazole	S18
**Table S4**.	Experimental and computed spectroscopic constants for [5-^{13}C]-oxazole	S19
**Figure S4**.	Data distribution plot for the least-squares fit of millimeter-wave spectroscopic data for [5-^{13}C]-oxazole	S20
**Table S5**.	Experimental and computed spectroscopic constants for [3-^{15}N]-oxazole	S21
**Figure S5**.	Data distribution plot for the least-squares fit of millimeter-wave spectroscopic data for [3-^{15}N]-oxazole	S22
**Table S6**.	Experimental and computed spectroscopic constants for [1-^{18}O]-oxazole	S23
**Figure S6**.	Data distribution plot for the least-squares fit of millimeter-wave spectroscopic data for [1-^{18}O]-oxazole	S24
**Table S7**.	Experimental and computed spectroscopic constants for [2-^{2}H]-oxazole	S25
**Figure S7**.	Data distribution plot for the least-squares fit of millimeter-wave spectroscopic data for [2-^{2}H]-oxazole	S26
**Table S8**.	Experimental and computed spectroscopic constants for [4-^{2}H]-oxazole	S27

Table of Contents		Page
**Figure S8**.	Data distribution plot for the least-squares fit of millimeter-wave spectroscopic data for [4-^{2}H]-oxazole	S28
**Table S9**.	Experimental and computed spectroscopic constants for [5-^{2}H]-oxazole	S29
**Figure S9**.	Data distribution plot for the least-squares fit of millimeter-wave spectroscopic data for [5-^{2}H]-oxazole	S30
**Table S10**.	Experimental and computed spectroscopic constants for [2-^{2}H, 2-^{13}C]-oxazole	S31
**Figure S10**.	Data distribution plot for the least-squares fit of millimeter-wave spectroscopic data for [2-^{2}H, 2-^{13}C]-oxazole	S32
**Table S11**.	Experimental and computed spectroscopic constants for [2-^{2}H, 4-^{13}C]-oxazole	S33
**Figure S11**.	Data distribution plot for the least-squares fit of millimeter-wave spectroscopic data for [2-^{2}H, 4-^{13}C]-oxazole	S34
**Table S12**.	Experimental and computed spectroscopic constants for [2-^{2}H, 5-^{13}C]-oxazole	S35
**Figure S12**.	Data distribution plot for the least-squares fit of millimeter-wave spectroscopic data for [2-^{2}H, 5-^{13}C]-oxazole	S36
**Table S13**.	Experimental and computed spectroscopic constants for [2-^{2}H, 3-^{15}N]-oxazole	S37
**Figure S13**.	Data distribution plot for the least-squares fit of millimeter-wave spectroscopic data for [2-^{2}H, 3-^{15}N]-oxazole	S38
**Table S14**.	Experimental and computed spectroscopic constants for [2-^{2}H, 1-^{18}O]-oxazole	S39
**Figure S14**.	Data distribution plot for the least-squares fit of millimeter-wave spectroscopic data for [2-^{2}H, 1-^{18}O]-oxazole	S40
**Table S15**.	Experimental and computed spectroscopic constants for [2,5-^{2}H]-oxazole	S41
**Figure S15**.	Data distribution plot for the least-squares fit of millimeter-wave spectroscopic data for [2,5-^{2}H]-oxazole	S42
**Table S16**.	Experimental and computed spectroscopic constants for [2,5-^{2}H, 2-^{13}C]-oxazole	S43
**Figure S16**.	Data distribution plot for the least-squares fit of millimeter-wave spectroscopic data for [2,5-^{2}H, 2-^{13}C]-oxazole	S44
**Table S17**.	Experimental and computed spectroscopic constants for [2,5-^{2}H, 4-^{13}C]-oxazole	S45
**Figure S17**.	Data distribution plot for the least-squares fit of millimeter-wave spectroscopic data for [2,5-^{2}H, 4-^{13}C]-oxazole	S46
**Table S18**.	Experimental and computed spectroscopic constants for [2,5-^{2}H, 5-^{13}C]-oxazole	S47

	Table of Contents	Page
**Figure S18**.	Data distribution plot for the least-squares fit of millimeter-wave spectroscopic data for [2,5-^{2}H, 5-^{13}C]-oxazole	S48
**Table S19**.	Experimental and computed spectroscopic constants for [2,5-^{2}H, 3-^{15}N]-oxazole	S49
**Figure S19**.	Data distribution plot for the least-squares fit of millimeter-wave spectroscopic data for [2,5-^{2}H, 3-^{15}N]-oxazole	S50
**Table S20**.	Experimental and computed spectroscopic constants for [2,5-^{2}H, 1-^{18}O]-oxazole	S51
**Figure S20**.	Data distribution plot for the least-squares fit of millimeter-wave spectroscopic data for [2,5-^{2}H, 1-^{18}O]-oxazole	S52
**Table S21**.	Experimental and computed spectroscopic constants for [2,4-^{2}H]-oxazole	S53
**Figure S21**.	Data distribution plot for the least-squares fit of millimeter-wave spectroscopic data for [2,4-^{2}H]-oxazole	S54
**Table S22**.	Experimental and computed spectroscopic constants for [4,5-^{2}H]-oxazole	S55
**Figure S22**.	Data distribution plot for the least-squares fit of millimeter-wave spectroscopic data for [4,5-^{2}H]-oxazole	S56
**Table S23**.	Experimental and computed spectroscopic constants for [2,4,5-^{2}H]-oxazole	S57
**Figure S23**.	Data distribution plot for the least-squares fit of millimeter-wave spectroscopic data for [2,4,5-^{2}H]-oxazole	S58
**Table S24**.	Experimental and computed spectroscopic constants for [5-^{2}H, 2-^{13}C]-oxazole	S59
**Figure S24**.	Data distribution plot for the least-squares fit of millimeter-wave spectroscopic data for [5-^{2}H, 2-^{13}C]-oxazole	S60
**Table S25**.	Experimental and computed spectroscopic constants for [5-^{2}H, 4-^{13}C]-oxazole	S61
**Figure S25**.	Data distribution plot for the least-squares fit of millimeter-wave spectroscopic data for [5-^{2}H, 4-^{13}C]-oxazole	S62
**Table S26**.	Experimental and computed spectroscopic constants for [5-^{2}H, 5-^{13}C]-oxazole	S63
**Figure S26**.	Data distribution plot for the least-squares fit of millimeter-wave spectroscopic data for [5-^{2}H, 5-^{13}C]-oxazole	S64
**Table S27**.	Experimental and computed spectroscopic constants for [5-^{2}H, 3-^{15}N]-oxazole	S65
**Figure S27**.	Data distribution plot for the least-squares fit of millimeter-wave spectroscopic data for [5-^{2}H, 3-^{15}N]-oxazole	S66
**Table S28**.	Experimental and computed spectroscopic constants for [2,4,5-^{2}H, 2-^{13}C]-oxazole	S67

Table of Contents		Page
**Figure S28**.	Data distribution plot for the least-squares fit of millimeter-wave spectroscopic data for [2,4,5-^{2}H, 2-^{13}C]-oxazole	S68
**Table S29**.	Experimental and computed spectroscopic constants for [2,4,5-^{2}H, 4-^{13}C]-oxazole	S69
**Figure S29**.	Data distribution plot for the least-squares fit of millimeter-wave spectroscopic data for [2,4,5-^{2}H, 4-^{13}C]-oxazole	S70
**Table S30**.	Experimental and computed spectroscopic constants for [2,4,5-^{2}H, 5-^{13}C]-oxazole	S71
**Figure S30**.	Data distribution plot for the least-squares fit of millimeter-wave spectroscopic data for [2,4,5-^{2}H, 5-^{13}C]-oxazole	S72
**Figure S31**.	Plots of the structural parameters as a function of the number of isotopologues for r_e^{SE}(final) structure	S76
**r_e vs. r_e^{SE} Rotation Analysis**		S77
**Table S32.**	Center-of-Mass Coordinates, exact masses, and products for the r_e and r_e^{SE} structures of the normal isotopologue of oxazole	S78
**Table S33.**	Center-of-Mass Coordinates, exact masses, and products for the r_e and r_e^{SE} structures of [4,5-^{2}H]- oxazole	S78
**Table S34.**	Angles of rotation of principal axes relative to the normal isotopologue using different oxazole structures	S79
**Eq. S19.**	General relation between the observable and equilibrium rotational constants and the α and γ contributions to the vibration-rotation interaction correction	S79
**Table S35.**	Vibration-Rotation Interaction Corrections (α) before and after application of r_e^{SE}-based vibration-rotation interaction corrections	S80
**Table S36.**	Residuals (difference between measured rotational constants and rotational constants predicted from semi-experimental structure) before and after application of r_e^{SE}-based vibration-rotation interaction corrections	S81
**Table S37.**	Fully corrected inertial defects ($\Delta_{i\,e}$) of oxazole isotopologues from various r_e^{SE} structures	S82
**Table S38.**	Experimental and computational structural parameters of oxazole.	S83
**Table S39** through **Table S43** –	Coordinates and uncertainties of semi-experimental substitution (r_s^{SE}) structure using various isotopologue reference systems	S84 - S85
**Table S44.**	Semi-experimental substitution (r_s^{SE}) structure parameters that could be determined from various isotopologue reference systems	S85
**Figure S32.**	Plot of δr_e^{SE} values as a function of the number of isotopologues (N_{iso}) incorporated in the structure determination for initial, final, and round 2 r_e^{SE} structures.	S86

Table of Contents		Page
**Figure S33.**	Plots of the structural parameters as a function of the number of isotopologues (N_{iso}) and their 2σ uncertainties for initial, final, and round 2 r_e^{SE} structures.	S87
**Synthetic Details**		S88
**References**		S96

Additional Files Provided (zipped file)

Computational outputs	Separate files
Initial r_e^{SE} structure output (*oxazole_reSE.out*)	Separate file
Final r_e^{SE} structure output (*oxazole_reSE_redone.out*)	Separate file
2nd Iteration r_e^{SE} structure output (*oxazole_reSE_redone_2.out*)	Separate file
Xrefit output without computational corrections (provides $\Delta_{i\,0}$) (*oxazole_r0.out*)	Separate file
Xrefit output without electron-mass distribution correction (provides $\Delta_{i\,e}$ using vibration-rotation interaction correction only) (*oxazole_reSE_noE.out*)	Separate file
Files containing calculation of center-of-mass coordinates (*oxazole_pac_*_pac.out*)	Separate files
xrefiteration results (results for multiple structures included as separate sheets)	Separate file

Equations for Calculating the Determinable Constants

The ground-state rotational constants for both the S- and A-reduced Hamiltonians were converted to the "determinable constants" (A_0'', B_0'', C_0'') to account for the influence of centrifugal distortion using eq. (S1) – (S6).[1] The resultant constants were then averaged to obtain the values input into *xrefit.*

$$A_0'' = A_0^{(A)} + 2\Delta_\mathrm{J} \tag{S1}$$
$$B_0'' = B_0^{(A)} + 2\Delta_\mathrm{J} + \Delta_\mathrm{JK} - 2\delta_J - 2\delta_K \tag{S2}$$
$$C_0'' = C_0^{(A)} + 2\Delta_\mathrm{J} + \Delta_\mathrm{JK} + 2\delta_J + 2\delta_K \tag{S3}$$

$$A_0'' = A_0^{(S)} + 2D_\mathrm{J} + 6d_2 \tag{S4}$$
$$B_0'' = B_0^{(S)} + 2D_J + D_{JK} + 2d_1 + 4d_2 \tag{S5}$$
$$C_0'' = C_0^{(S)} + 2D_J + D_{JK} - 2d_1 + 4d_2 \tag{S6}$$

Ideally, the B_0'' values determined from the A and S reductions are equal. The magnitude of the difference between these values is informative regarding the quality of the determined constants. Small magnitudes in these differences provide confidence that both models employed in the least-squares fits are adequately treating the datasets and that the values obtained are suitable for use in the r_e^{SE} structure determination.

Equations for Calculating the Best Theoretical Estimate (BTE)

A "best theoretical estimate" (BTE) for the structure of 1,3-oxazole uses the CCSD(T)/cc-pCV5Z structure as a basis, with four corrections that are evaluated using conventional methodology:[2-3]

1. Residual basis set effects beyond cc-pCV5Z: To estimate the correction needed to approach the infinite basis set limit, equilibrium structural parameters obtained with the cc-pCVXZ (X = T, Q, and 5) basis sets were extrapolated using the empirical exponential expression in Eq. (S7).[2-4]

$$R(x) = R(\infty) + Ae^{-Bx} \tag{S7}$$

R(x) are the values of the parameters obtained using the various basis sets (x = 3, 4, and 5), and R(∞) is the desired basis set limit estimate. Using three basis sets (x = 3, 4, and 5), the system of equations using Eq. (S7) can be solved, yielding Eq. (S8).[2]

$$R(\infty) = -\frac{R(4)^2 - R(3)R(5)}{R(3) + R(5) - 2R(4)} \tag{S8}$$

The correction to the structure due to a finite basis set is estimated by Eq. (S9).

$$\Delta R(\text{basis}) = R(\infty) - R(\text{CCSD(T)/cc-pCV5Z}) \tag{S9}$$

2. Residual electron correlation effects beyond the CCSD(T) treatment: Residual correlation effects are assessed by doing geometry optimizations at the CCSDT(Q) level[5] and then estimating the correlation correction in Eq. (S10).

$$\Delta R(\text{cor}) = R(\text{CCSDT(Q)}) - R(\text{CCSD(T)}) \tag{S10}$$

As calculations at the CCSDT(Q) level of theory are computationally demanding, these two calculations are obtained with the cc-pVDZ basis, in the frozen-core approximation.

3. Effects of scalar (mass-velocity and Darwin) relativistic effects: The relativistic corrections are obtained by subtraction of the equilibrium parameters obtained with a standard non-relativistic calculation from those obtained with the X2C-1e variant of coupled-cluster theory,[6-8] as shown in Eq. (S11).

$$\Delta R(\text{rel}) = R(\text{CCSD(T)/cc-pCVTZ})_{\text{SFX2C-1e}} - R(\text{CCSD(T)/cc-pCVTZ})_{NR} \tag{S11}$$

The relativistic and non-relativistic calculations were performed with the cc-pVTZ basis set. All electrons were correlated in both calculations. A re-contracted basis set was not used for the relativistic calculation. (Keyword: RELATIVISTIC=X2C1E)

4. The diagonal Born-Oppenheimer correction (DBOC): The diagonal Born-Oppenheimer correction (DBOC)[9-10] is obtained from Eq. (S12).

$$\Delta R(\text{DBOC}) = R(\text{SCF/cc-pVTZ})_{DBOC} - R(\text{SCF/cc-pVTZ})_{NR} \tag{S12}$$

Here, the first value is obtained by minimizing the DBOC-corrected SCF energy with respect to nuclear positions, and the latter is again the traditional calculation. (Keyword: DBOC=ON)

The sum of the above-described corrections is used to obtain the best equilibrium structural parameters, given by Eq. (S13).

$$\Delta R(\text{total}) = \Delta R(\text{basis}) + \Delta R(\text{cor}) + \Delta R(\text{rel}) + \Delta R(\text{DBOC}) \qquad \text{(S13)}$$

The sum of the corrections, ΔR(total), is applied to the CCSD(T)/cc-pCV5Z structural parameters.

Table S0. Corrections (Δ*R*) to Applied to Computed Structural Parameters (CCSD(T)/cc-pCV5Z) to Afford the Best Theoretical Estimate (BTE) for Oxazole [a,b]

Parameter	Δ*R*(basis)	Δ*R*(rel)	Δ*R*(corr)	Δ*R*(DBOC)	Δ*R*(Total)
$R_{O–C2}$ (Å)	−0.00009	0.00003	0.00166	0.000010	0.00161
$R_{O–C5}$ (Å)	−0.00009	0.00006	0.00113	0.000017	0.00112
$R_{C2–N}$ (Å)	−0.00026	−0.00019	0.00076	0.000004	0.00031
$R_{C4–C5}$ (Å)	−0.00026	−0.00032	0.00073	−0.000001	0.00015
$R_{C2–H}$ (Å)	−0.00001	−0.00012	0.00004	0.000142	0.00005
$R_{C5–H}$ (Å)	−0.00003	−0.00012	0.00010	0.000137	0.00009
$R_{C4–H}$ (Å)	−0.00003	−0.00013	0.00011	0.000133	0.00008
$\theta_{C5–O–C2}$ (°)	0.0099	−0.0243	−0.0507	0.00211	−0.0629
$\theta_{N–C2–O}$ (°)	−0.0283	0.0210	0.0129	−0.00239	0.0032
$\theta_{C4–C5–O}$ (°)	−0.0018	0.0090	0.0241	0.00004	0.0313
$\theta_{O–C2–H}$ (°)	0.0069	−0.0173	−0.0263	0.00311	−0.0336
$\theta_{O–C5–H}$ (°)	0.00001	−0.0204	−0.0328	0.00151	−0.0517
$\theta_{H–C4–C5}$ (°)	0.0599	0.0035	0.0020	0.00096	0.0664

[a] Exact values were used for all mathematical functions, but values in table are rounded to one decimal more than provided in structural determination for legibility, resulting in apparent discrepancies between sum of rounded corrections shown and rounded Δ*R*(Total).

[b] Rows highlighted in grey indicate parameters for which the value of the BTE falls outside 2σ of the r_e^{SE} value determined using the r_e^{SE}-based VPT2 calculation.

Isotopologue numbering scheme for convenience in examining anharmonic computational, magnetic computational, and spectroscopic least-squares fitting output files

ISOMASS Number	Isotopologue
001	C_3H_3NO
002	[2-^{13}C]-
003	[4-^{13}C]-
004	[5-^{13}C]-
005	[3-^{15}N]-
006	[1-^{18}O]-
007	[2-^{2}H]-
008	[4-^{2}H]-
009	[5-^{2}H]-
010	[2-^{2}H, 2-^{13}C]-
011	[2-^{2}H, 4-^{13}C]-
012	[2-^{2}H, 5-^{13}C]-
013	[2-^{2}H, 3-^{15}N]-
014	[2-^{2}H, 1-^{18}O]-
015	[2,5-^{2}H]-
016	[2,5-^{2}H, 2-^{13}C]-
017	[2,5-^{2}H, 4-^{13}C]-
018	[2,5-^{2}H, 5-^{13}C]-
019	[2,5-^{2}H, 3-^{15}N]-
020	[2,5-^{2}H, 1-^{18}O]-
021	[2,4-^{2}H]-
022	[4,5-^{2}H]-
023	[2,4,5-^{2}H]-
024	[5-^{2}H, 2-^{13}C]-
025	[5-^{2}H, 4-^{13}C]-
026	[5-^{2}H, 5-^{13}C]-
027	[5-^{2}H, 3-^{15}N]-
028	[5-^{2}H, 1-^{18}O]- [a]
029	[2,4,5-^{2}H, 2-^{13}C]-
030	[2,4,5-^{2}H, 4-^{13}C]-
031	[2,4,5-^{2}H, 5-^{13}C]-
032	[2,4,5-^{2}H, 3-^{15}N]- [a]
033	[2,4,5-^{2}H, 1-^{18}O]- [a]

[a] ISOMASS 028, 032, and 033 were predicted and searched for in the spectra, but not identified.

Overview of tables of spectroscopic constants and figures of data distribution plots. For archival purposes, data for all isotopologues are included in the Supporting Information, even if small portions of these data have been included in the manuscript. For all of the tables, it should be noted: Values in square brackets have been held constant at the computed value [CCSD(T)/cc-pCVTZ] in the least-squares fit, and III^r values were converted from the III^l representation output by SPFIT.

Table S1. Experimental and computed spectroscopic constants for the normal isotopologue of oxazole

	S Reduction, I^r representation			S Reduction, III^r representation [a]		
	Experimental (Hyperfine Excluded)	Experimental (Hyperfine Included)	CCSD(T) [b]	Experimental (Hyperfine Excluded)	Experimental (Hyperfine Included)	CCSD(T) [b]
$A_0^{(S)}$ (MHz)	10050.994 628 (60)	10050.996 587 (47)	10024	10050.993 839 (60)	10050.995 804 (47)	10024
$B_0^{(S)}$ (MHz)	9645.729 398 (52)	9645.729 140 (45)	9579	9645.730 777 (52)	9645.730 520 (45)	9579
$C_0^{(S)}$ (MHz)	4919.404 307 (48)	4919.404 517 (53)	4895	4919.403 024 (48)	4919.403 233 (53)	4895
D_J (kHz)	1.403 322 (30)	1.402 508 (24)	1.36	3.759 606 (28)	3.759 257 (30)	3.68
D_{JK} (kHz)	3.245 03 (12)	3.246 727 (50)	3.22	−5.891 440 (47)	−5.891 699 (48)	−5.76
D_K (kHz)	−0.904 69 (12)	−0.905 429 (56)	−0.902	2.541 322 (29)	2.541 538 (31)	2.48
d_1 (kHz)	−0.810 577 (12)	−0.810 417 5 (89)	−0.786	−0.022 961 (22)	−0.023 259 4 (77)	−0.034 4
d_2 (kHz)	−0.313 656 9 (55)	−0.313 709 0 (43)	−0.305	0.030 943 4 (71)	0.030 986 0 (34)	0.033 2
H_J (Hz)	−0.000 377 3 (75)	−0.000 544 0 (42)	−0.000 404	0.001 511 2 (50)	0.001 433 4 (51)	0.001 51
H_{JK} (Hz)	0.006 010 (41)	0.006 419 (17)	0.005 78	−0.006 570 (11)	−0.006 560 (10)	−0.006 51
H_{KJ} (Hz)	−0.007 779 (49)	−0.007 754 (27)	−0.006 75	0.008 706 (12)	0.008 624 (12)	0.008 45
H_K (Hz)	0.003 334 (53)	0.002 963 (27)	0.002 53	−0.003 615 5 (58)	−0.003 523 0 (63)	−0.003 46
h_1 (Hz)	0.000 216 8 (27)	0.000 184 6 (15)	0.000 214	−0.000 117 7 (68)	−0.000 090 1 (27)	−0.000 100
h_2 (Hz)	0.000 552 9 (22)	0.000 575 4 (13)	0.000 545	−0.000 103 6 (41)	−0.000 136 1 (20)	−0.000 121
h_3 (Hz)	0.000 131 62 (47)	0.000 131 93 (27)	0.000 132	0.000 056 77 (86)	0.000 050 82 (45)	0.000 045
χ_{aa} (MHz)		−4.071 (14)	−3.934		−5.072 (47)	−3.934
$\chi_{bb} - \chi_{cc}$ (MHz)		−1.026 (40)	−0.828		−2.020 (76)	−0.828
χ_{ab} (MHz)		0 [c]	1.009		0 [c]	1.009
$\chi_{aa,J}$ (MHz)		0.000893 (87)			−0.000 093 (13)	
$\chi_{aa,K}$ (MHz)		−0.00089 (12)			0.002 08 (15)	
$[\chi_{bb} - \chi_{cc}]_J$ (MHz)		0.001144 (92)			[0]	
$[\chi_{bb} - \chi_{cc}]_K$ (MHz)		−0.00280 (40)			[0]	
N_{lines} [d]	3962	5402		3962	5402	
σ_{fit} (MHz)	0.033	0.039		0.033	0.039	

[a] Converted from the III^l representation output of SPFIT. [b] Evaluated using the cc-pCVTZ basis set. [c] Value was allowed to fit, but remained at zero. [d] Number of independent transitions.

Table S1 Continued. Experimental and computed spectroscopic constants for the normal isotopologue of oxazole

	A Reduction, I^r representation		
	Experimental (Hyperfine Excluded)	Experimental (Hyperfine Included)	CCSD(T) [b]
$A_0^{(A)}$ (MHz)	10050.991 493 (60)	10050.993 453 (47)	10024
$B_0^{(A)}$ (MHz)	9645.733 580 (52)	9645.733 331 (45)	9579
$C_0^{(A)}$ (MHz)	4919.402 621 (48)	4919.402 831 (53)	4895
Δ_J (kHz)	2.030 633 (28)	2.029 928 (23)	1.97
Δ_{JK} (kHz)	−0.518 863 (83)	−0.517 793 (41)	−0.435
Δ_K (kHz)	2.231 883 (77)	2.231 667 (45)	2.14
δ_J (kHz)	0.810 576 (12)	0.810 418 5 (89)	0.786
δ_K (kHz)	1.469 806 (26)	1.470 053 (20)	1.45
Φ_J (Hz)	0.000 727 7 (56)	0.000 606 9 (36)	0.000 685
Φ_{JK} (Hz)	0.001 842 (24)	0.001 986 (11)	0.001 76
Φ_{KJ} (Hz)	−0.010 484 (31)	−0.010 251 (21)	−0.009 70
Φ_K (Hz)	0.009 108 (35)	0.008 746 (21)	0.008 41
ϕ_J (Hz)	0.000 347 6 (26)	0.000 316 2 (15)	0.000 345
ϕ_{JK} (Hz)	0.001 321 7 (87)	0.001 424 7 (60)	0.001 35
ϕ_K (Hz)	0.002 586 (11)	0.002 593 6 (65)	0.002 62
χ_{aa} (MHz)		−4.071 (14)	−3.934
$\chi_{bb} - \chi_{cc}$ (MHz)		−1.027 (40)	−0.828
χ_{ab} (MHz)		0 [c]	1.009
$\chi_{aa,J}$ (MHz)		0.000 880 (87)	
$\chi_{aa,K}$ (MHz)		−0.000 87 (12)	
$[\chi_{bb} - \chi_{cc}]_J$ (MHz)		0.001 140 (92)	
$[\chi_{bb} - \chi_{cc}]_K$ (MHz)		−0.002 72 (40)	
N_{lines} [d]	3962	5402	
σ_{fit} (MHz)	0.033	0.039	

[a] Converted from the III^l representation output of SPFIT. [b] Evaluated using the cc-pCVTZ basis set. [c] Value was allowed to fit, but remained at zero. [d] Number of independent transitions.

Table S1 Continued. Experimental and computed spectroscopic constants for the normal isotopologue of oxazole

	A Reduction, III^r representation [a]			
	Experimental Hegelund *et al.*[11]	Experimental (Current Work, Hyperfine Excluded)	Experimental (Current Work, Hyperfine Included)	CCSD(T) [b]
$A_0^{(A)}$ (MHz)	10050.993 79 (678)	10050.999 932 (85)	10051.001 718 (75)	10024
$B_0^{(A)}$ (MHz)	9645.759 53 (678)	9645.724 828 (74)	9645.724 584 (71)	9579
$C_0^{(A)}$ (MHz)	4919.399 58 (438)	4919.403 212 (68)	4919.403 385 (84)	4895
Δ_J (kHz)	3.711 6 (24)	3.697 364 (43)	3.697 002 (49)	3.61
Δ_{JK} (kHz)	−5.592 (12)	−5.517 41 (11)	−5.517 703 (94)	−5.36
Δ_K (kHz)	2.287 (10)	2.229 397 (86)	2.229 592 (67)	2.14
δ_J (kHz)	0.189 (14)	0.022 771 (32)	0.023 058 (12)	0.034 4
δ_K (kHz)	2.34 (10)	3.034 92 (74)	3.032 63 (44)	2.88
Φ_J (Hz)		0.000 949 (11)	0.000 853 4 (95)	0.001 51
Φ_{JK} (Hz)		−0.006 106 (73)	−0.007 398 (69)	−0.006 509
Φ_{KJ} (Hz)		0.015 63 (19)	0.020 10 (21)	0.008 45
Φ_K (Hz)		−0.010 47 (13)	−0.013 63 (15)	−0.003 457
ϕ_J (Hz)		−0.000 246 6 (89)	−0.000 214 9 (37)	−0.000 100
ϕ_{JK} (Hz)		0.029 87 (34)	0.029 39 (20)	−0.000 121
ϕ_K (Hz)		0.003 93 (98)	0.028 7 (11)	0.000 044 6
χ_{aa} (MHz)			−5.60 (73)	−3.934
$\chi_{bb} - \chi_{cc}$ (MHz)			−1.93 (12)	−0.828
χ_{ab} (MHz)			0 [c]	1.009
$\chi_{aa,J}$ (MHz)				
$\chi_{aa,K}$ (MHz)				
$[\chi_{bb} - \chi_{cc}]_J$ (MHz)			0.147 (14)	
$[\chi_{bb} - \chi_{cc}]_K$ (MHz)				
N_{lines} [d]		3962	5402	
σ_{fit} (MHz)		0.046	0.062	

[a] Converted from the III^l representation output of SPFIT. [b] Evaluated using the cc-pCVTZ basis set. [c] Value was allowed to fit, but remained at zero. [d] Number of independent transitions.

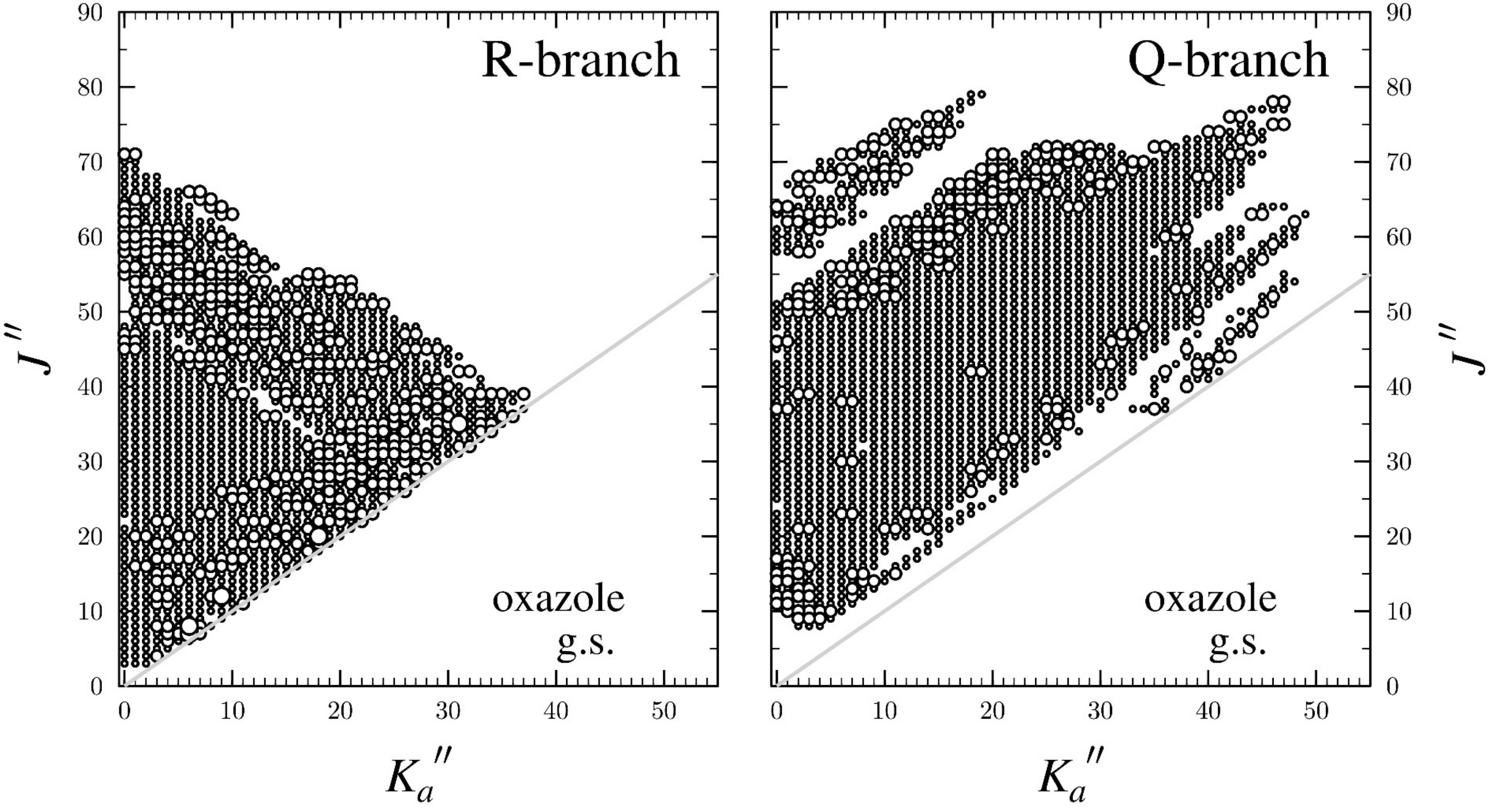

Figure S1. Data distribution plot for the least-squares fit of millimeter-wave spectroscopic data for the normal isotopologue of oxazole, ground vibrational state, excluding hyperfine resolved transitions. The size of the outlined circle is proportional to the value of $|(f_{obs.} - f_{calc.})/\delta f|$, where δf is the frequency measurement uncertainty (50 kHz), and no quotient values are larger than three.

Table S2. Experimental and computed spectroscopic constants for [2-^{13}C]-oxazole [a]

	S Reduction, III^r representation			A Reduction, I^r representation	
	Experimental	CCSD(T) [b]		Experimental	CCSD(T) [b]
A_0 (MHz)	9835.057 79 (27)	9807	A_0 (MHz)	9835.055 10 (28)	9807
B_0 (MHz)	9636.714 74 (23)	9572	B_0 (MHz)	9636.717 45 (22)	9572
C_0 (MHz)	4864.781 108 (94)	4841	C_0 (MHz)	4864.781 026 (94)	4841
D_J (kHz)	3.672 93 (17)	3.60	Δ_J (kHz)	2.071 38 (24)	2.00
D_{JK} (kHz)	−5.762 00 (29)	−5.63	Δ_{JK} (kHz)	−0.974 51 (95)	−0.788
D_K (kHz)	2.488 22 (16)	2.43	Δ_K (kHz)	2.516 62 (81)	2.34
d_1 (kHz)	0.032 23 (25)	0.014 4	δ_J (kHz)	0.836 12 (12)	0.804
d_2 (kHz)	−0.002 877 (78)	0.008 70	δ_K (kHz)	1.287 36 (24)	1.30
H_J (Hz)	0.001 261 (52)	0.001 47	Φ_J (Hz)	0.000 639 (10)	0.000 598
H_{JK} (Hz)	−0.005 94 (13)	−0.006 33	Φ_{JK} (Hz)	0.002 36 (13)	0.002 22
H_{KJ} (Hz)	0.008 20 (17)	0.008 20	Φ_{KJ} (Hz)	−0.011 76 (33)	−0.010 4
H_K (Hz)	−0.003 481 (83)	−0.003 35	Φ_K (Hz)	0.009 30 (29)	0.008 89
h_1 (Hz)	[−0.000 053 3]	−0.000 053 3	ϕ_J (Hz)	[0.000 302]	0.000 302
h_2 (Hz)	[−0.000 114]	−0.000 114	ϕ_{JK} (Hz)	[0.001 40]	0.001 40
h_3 (Hz)	[0.000 073 1]	0.000 073 1	ϕ_K (Hz)	[0.002 10]	0.002 10
N_{lines} [c]	1057		N_{lines} [c]	1057	
σ_{fit} (MHz)	0.036		σ_{fit} (MHz)	0.036	

[a] Values in brackets are held constant at the computed values. [b] Evaluated using the cc-pCVTZ basis set. [c] Number of independent transitions.

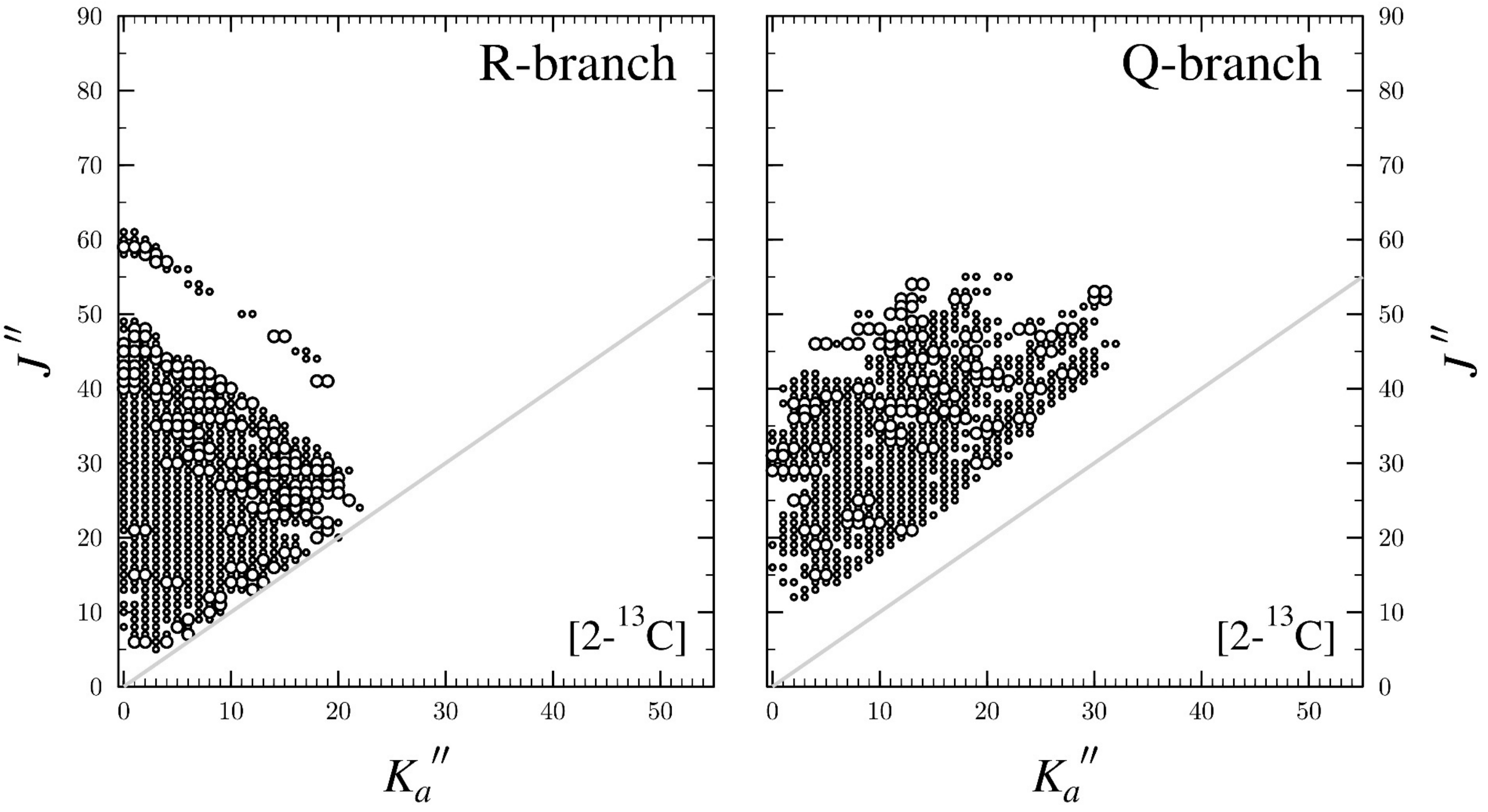

Figure S2. Data distribution plot for the least-squares fit of millimeter-wave spectroscopic data for [2-^{13}C]-oxazole, ground vibrational state. The size of the outlined circle is proportional to the value of $|(f_{obs.} - f_{calc.})/\delta f|$, where δf is the frequency measurement uncertainty (50 kHz), and no quotient values are larger than three.

Table S3. Experimental and computed spectroscopic constants for [4-^{13}C]-oxazole [a]

	S Reduction, III^r representation			A Reduction, I^r representation	
	Experimental	CCSD(T) [b]		Experimental	CCSD(T) [b]
A_0 (MHz)	9899.115 64 (23)	9869	A_0 (MHz)	9899.113 44 (24)	9869
B_0 (MHz)	9539.316 17 (19)	9477	B_0 (MHz)	9539.318 94 (18)	9477
C_0 (MHz)	4855.289 258 (91)	4831	C_0 (MHz)	4855.288 772 (91)	4831
D_J (kHz)	3.672 64 (15)	3.59	Δ_J (kHz)	1.915 21 (22)	1.87
D_{JK} (kHz)	−5.756 57 (25)	−5.62	Δ_{JK} (kHz)	−0.182 2 (13)	−0.148
D_K (kHz)	2.482 55 (14)	2.42	Δ_K (kHz)	1.979 0 (13)	1.92
d_1 (kHz)	−0.070 09 (20)	−0.071 9	δ_J (kHz)	0.758 30 (11)	0.740
d_2 (kHz)	0.050 35 (13)	0.050 4	δ_K (kHz)	1.483 82 (38)	1.46
H_J (Hz)	0.001 477 (39)	0.001 45	Φ_J (Hz)	0.000 806 5 (100)	0.000 785
H_{JK} (Hz)	−0.006 55 (10)	−0.006 29	Φ_{JK} (Hz)	0.000 94 (14)	0.000 123
H_{KJ} (Hz)	0.009 06 (16)	0.008 18	Φ_{KJ} (Hz)	−0.008 92 (35)	−0.005 97
H_K (Hz)	−0.003 971 (88)	−0.003 35	Φ_K (Hz)	0.008 41 (32)	0.006 18
h_1 (Hz)	[−0.000 104]	−0.000 104	ϕ_J (Hz)	[0.000 396]	0.000 396
h_2 (Hz)	[−0.000 054]	−0.000 054 0	ϕ_{JK} (Hz)	[0.000 766]	0.000 766
h_3 (Hz)	[−0.000 011 5]	−0.000 011 5	ϕ_K (Hz)	[0.003 14]	0.003 14
N_{lines} [c]	1157		N_{lines} [c]	1157	
σ_{fit} (MHz)	0.036		σ_{fit} (MHz)	0.036	

[a] Values in brackets are held constant at the computed values. [b] Evaluated using the cc-pCVTZ basis set. [c] Number of independent transitions.

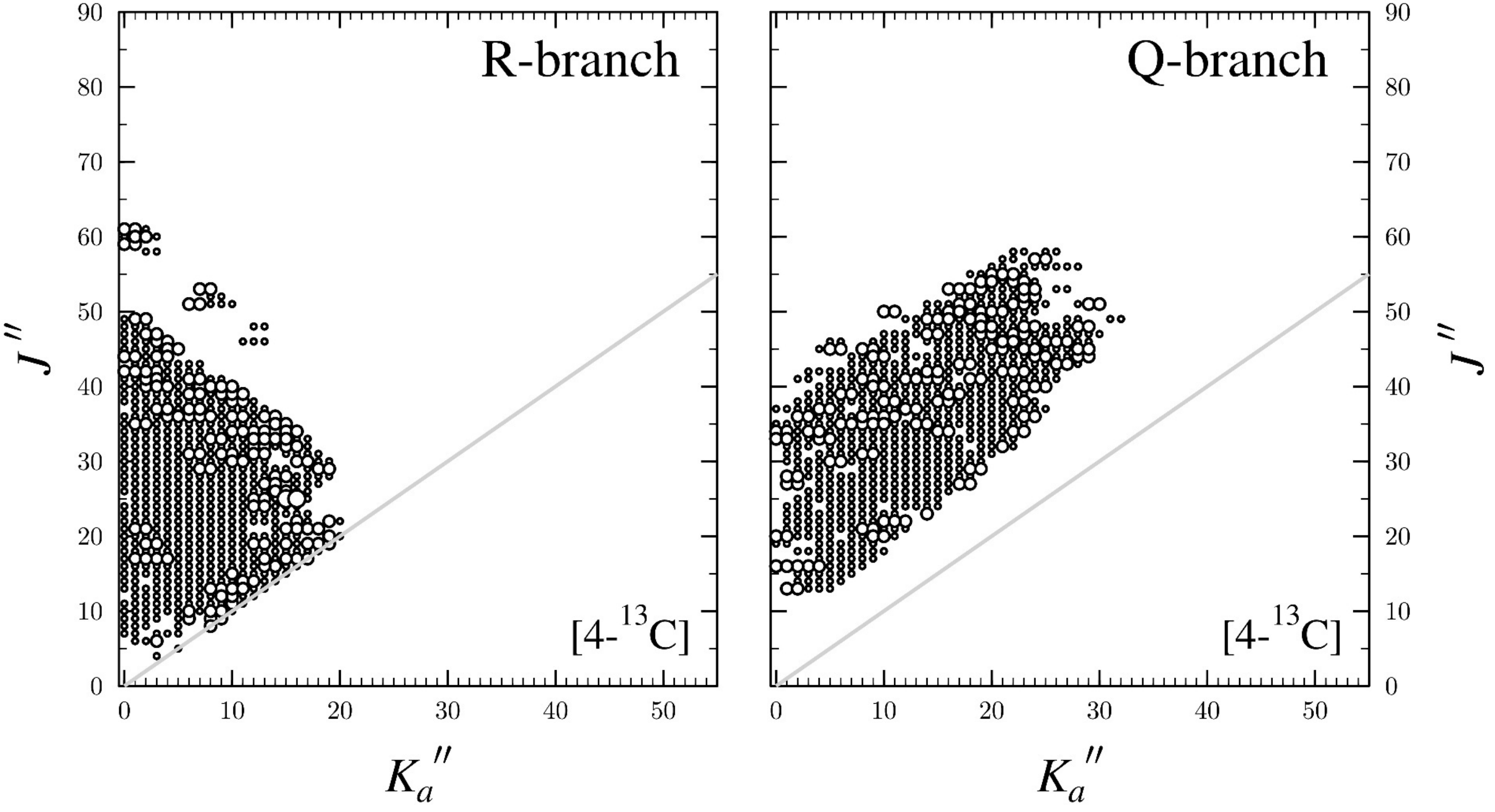

Figure S3. Data distribution plot for the least-squares fit of millimeter-wave spectroscopic data for [4-^{13}C]-oxazole, ground vibrational state. The size of the outlined circle is proportional to the value of $|(f_{obs.} - f_{calc.})/\delta f|$, where δf is the frequency measurement uncertainty (50 kHz), and no quotient values are larger than three.

Table S4. Experimental and computed spectroscopic constants for [5-^{13}C]-oxazole [a]

	S Reduction, III^r representation			A Reduction, I^r representation	
	Experimental	CCSD(T) [b]		Experimental	CCSD(T) [b]
A_0 (MHz)	9938.119 97 (28)	9907	A_0 (MHz)	9938.117 36 (29)	9907
B_0 (MHz)	9512.562 17 (23)	9451	B_0 (MHz)	9512.564 83 (22)	9451
C_0 (MHz)	4857.691 245 (97)	4834	C_0 (MHz)	4857.691 154 (97)	4834
D_J (kHz)	3.666 59 (17)	3.59	Δ_J (kHz)	2.052 85 (21)	1.99
D_{JK} (kHz)	−5.747 34 (27)	−5.62	Δ_{JK} (kHz)	−1.048 4 (14)	−0.903
D_K (kHz)	2.479 83 (15)	2.42	Δ_K (kHz)	2.717 0 (17)	2.57
d_1 (kHz)	−0.003 70 (26)	−0.016 2	δ_J (kHz)	0.826 887 (100)	0.799
d_2 (kHz)	−0.023 74 (17)	−0.014 9	δ_K (kHz)	1.284 31 (47)	1.29
H_J (Hz)	0.001 527 (39)	0.001 46	Φ_J (Hz)	0.000 710 (12)	0.000 716
H_{JK} (Hz)	−0.006 32 (11)	−0.006 31	Φ_{JK} (Hz)	0.001 58 (13)	0.000 910
H_{KJ} (Hz)	0.008 27 (20)	0.008 18	Φ_{KJ} (Hz)	−0.009 20 (44)	−0.007 93
H_K (Hz)	−0.003 49 (11)	−0.003 34	Φ_K (Hz)	0.008 27 (40)	0.007 61
h_1 (Hz)	[−0.000 071 4]	−0.000 071 4	ϕ_J (Hz)	[0.000 361]	0.000 361
h_2 (Hz)	[−0.000 046 6]	−0.000 046 6	ϕ_{JK} (Hz)	[0.000 918]	0.000 918
h_3 (Hz)	[0.000 038 0]	0.000 038 0	ϕ_K (Hz)	[0.002 81]	0.002 81
N_{lines} [c]	1077		N_{lines} [c]	1077	
σ_{fit} (MHz)	0.036		σ_{fit} (MHz)	0.036	

[a] Values in brackets are held constant at the computed values. [b] Evaluated using the cc-pCVTZ basis set. [c] Number of independent transitions.

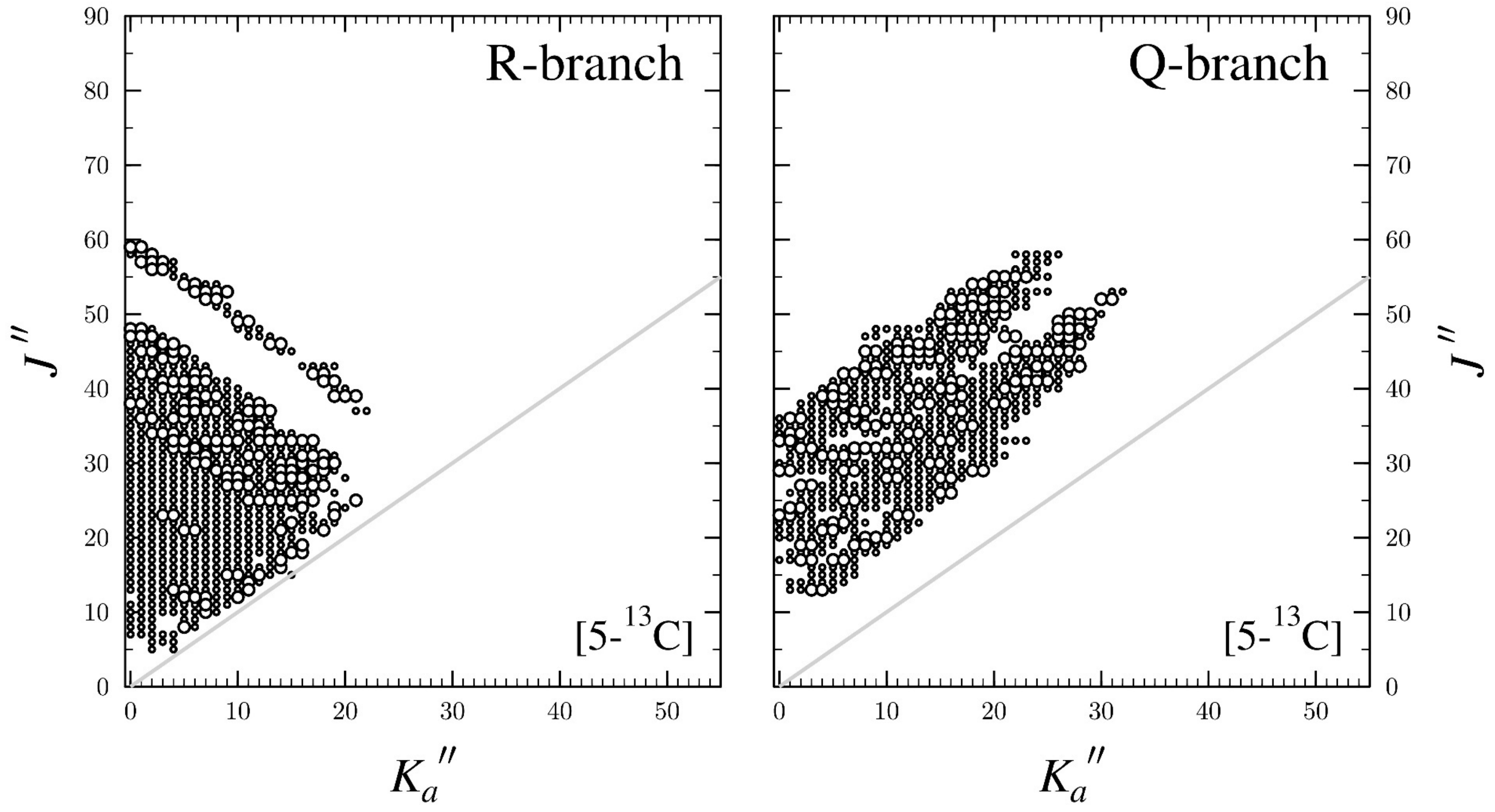

Figure S4. Data distribution plot for the least-squares fit of millimeter-wave spectroscopic data for [5-^{13}C]-oxazole, ground vibrational state. The size of the outlined circle is proportional to the value of $|(f_{obs.} - f_{calc.})/\delta f|$, where δf is the frequency measurement uncertainty (50 kHz), and no quotient values are larger than three.

Table S5. Experimental and computed spectroscopic constants for [3-^{15}N]-oxazole [a]

	S Reduction, III^r representation			A Reduction, I^r representation	
	Experimental	CCSD(T) [b]		Experimental	CCSD(T) [b]
A_0 (MHz)	10042.273 02 (40)	10014	A_0 (MHz)	10042.270 51 (48)	10014
B_0 (MHz)	9395.358 11 (25)	9331	B_0 (MHz)	9395.361 08 (27)	9331
C_0 (MHz)	4851.377 16 (21)	4827	C_0 (MHz)	4851.376 56 (11)	4827
D_J (kHz)	3.662 12 (25)	3.58	Δ_J (kHz)	1.970 14 (29)	1.91
D_{JK} (kHz)	−5.730 95 (66)	−5.60	Δ_{JK} (kHz)	−0.611 5 (16)	−0.523
D_K (kHz)	2.468 70 (48)	2.40	Δ_K (kHz)	2.395 6 (21)	2.30
d_1 (kHz)	−0.053 66 (47)	−0.062 6	δ_J (kHz)	0.785 34 (14)	0.761
d_2 (kHz)	0.007 13 (20)	0.010 7	δ_K (kHz)	1.430 16 (56)	1.42
H_J (Hz)	[0.001 44]	0.001 44	Φ_J (Hz)	[0.000 678]	0.000 678
H_{JK} (Hz)	−0.006 08 (31)	−0.006 24	Φ_{JK} (Hz)	[0.001 50]	0.001 50
H_{KJ} (Hz)	0.007 47 (47)	0.008 11	Φ_{KJ} (Hz)	−0.008 33 (81)	−0.009 29
H_K (Hz)	−0.002 65 (27)	−0.003 32	Φ_K (Hz)	0.006 92 (98)	0.008 33
h_1 (Hz)	[−0.000 083 2]	−0.000 083 2	ϕ_J (Hz)	[0.000 342]	0.000 342
h_2 (Hz)	[−0.000 076 1]	−0.000 076 1	ϕ_{JK} (Hz)	[0.001 22]	0.001 22
h_3 (Hz)	[0.000 047 3]	0.000 047 3	ϕ_K (Hz)	[0.002 74]	0.002 74
N_{lines} [c]	544		N_{lines} [c]	544	
σ_{fit} (MHz)	0.039		σ_{fit} (MHz)	0.039	

[a] Values in brackets are held constant at the computed values. [b] Evaluated using the cc-pCVTZ basis set. [c] Number of independent transitions.

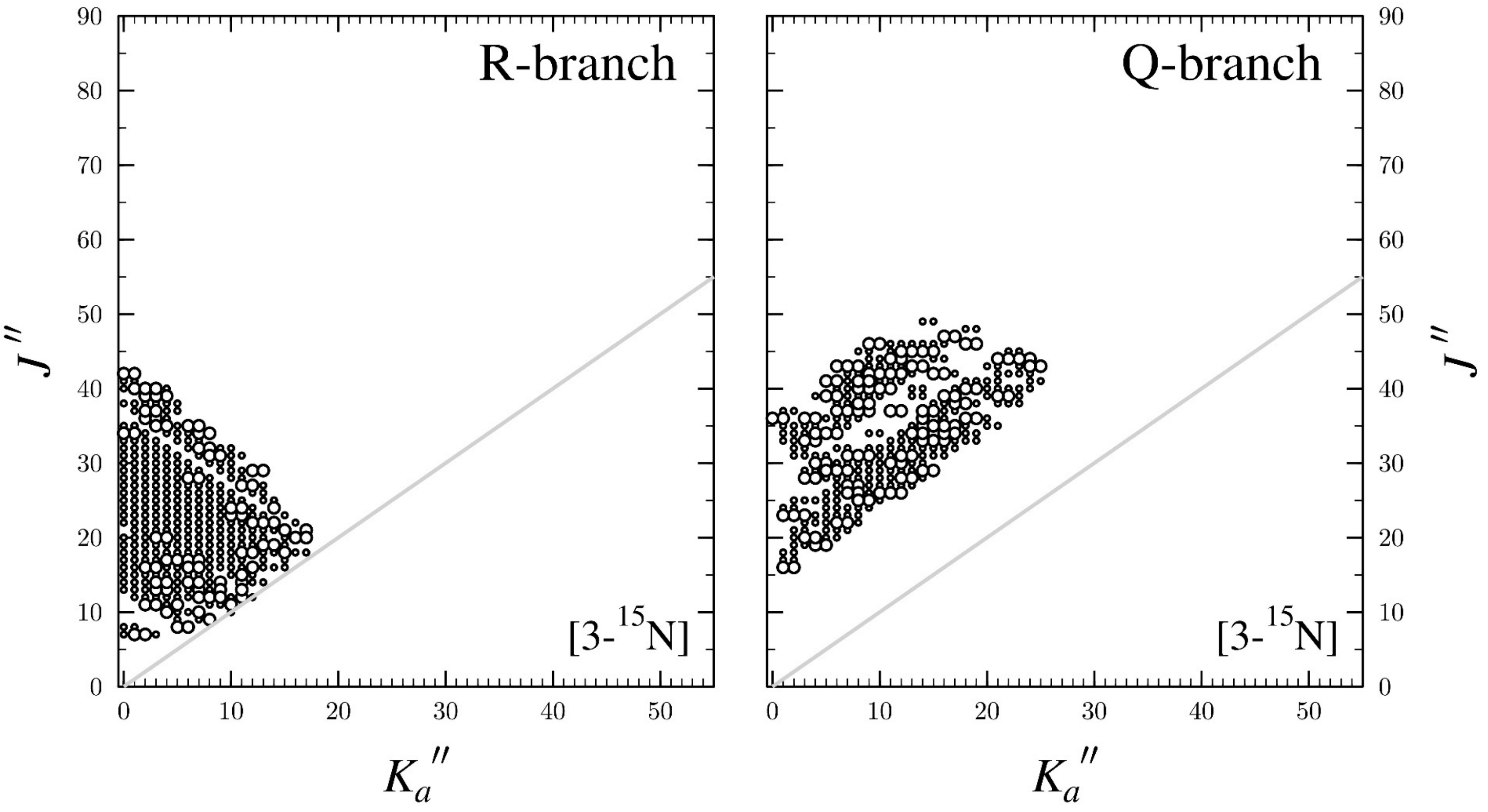

Figure S5. Data distribution plot for the least-squares fit of millimeter-wave spectroscopic data for [3-^{15}N]-oxazole, ground vibrational state. The size of the outlined circle is proportional to the value of $|(f_{obs.} - f_{calc.})/\delta f|$, where δf is the frequency measurement uncertainty (50 kHz), and no quotient values are larger than three.

Table S6. Experimental and computed spectroscopic constants for [1-^{18}O]-oxazole [a]

	S Reduction, III^r representation			A Reduction, I^r representation	
	Experimental	CCSD(T) [b]		Experimental	CCSD(T) [b]
A_0 (MHz)	10014.349 40 (58)	9986	A_0 (MHz)	10014.347 22 (58)	9986
B_0 (MHz)	9232.046 62 (47)	9169	B_0 (MHz)	9232.049 53 (47)	9169
C_0 (MHz)	4801.031 25 (22)	4777	C_0 (MHz)	4801.030 58 (22)	4777
D_J (kHz)	3.616 17 (34)	3.54	Δ_J (kHz)	1.860 15 (57)	1.81
D_{JK} (kHz)	−5.644 28 (54)	−5.51	Δ_{JK} (kHz)	−0.133 5 (33)	−0.110
D_K (kHz)	2.424 26 (30)	2.36	Δ_K (kHz)	2.019 7 (42)	1.98
d_1 (kHz)	−0.105 55 (71)	−0.110	δ_J (kHz)	0.732 00 (28)	0.714
d_2 (kHz)	0.040 46 (41)	0.038 4	δ_K (kHz)	1.540 4 (11)	1.51
H_J (Hz)	0.001 406 (54)	0.001 36	Φ_J (Hz)	0.000 767 (54)	0.000 722
H_{JK} (Hz)	[−0.005 98]	−0.005 98	Φ_{JK} (Hz)	[0.000 439]	0.000 439
H_{KJ} (Hz)	[0.007 83]	0.007 83	Φ_{KJ} (Hz)	[−0.006 67]	−0.006 67
H_K (Hz)	[−0.003 22]	−0.003 22	Φ_K (Hz)	[0.006 59]	0.006 59
h_1 (Hz)	[−0.000 090 0]	−0.000 090 0	ϕ_J (Hz)	[0.000 364]	0.000 364
h_2 (Hz)	[−0.000 050 1]	−0.000 050 1	ϕ_{JK} (Hz)	[0.000 851]	0.000 85
h_3 (Hz)	[−0.000 004 20]	−0.000 004 20	ϕ_K (Hz)	[0.003 30]	0.003 30
N_{lines} [c]	436		N_{lines} [c]	436	
σ_{fit} (MHz)	0.046		σ_{fit} (MHz)	0.046	

[a] Values in brackets are held constant at the computed values. [b] Evaluated using the cc-pCVTZ basis set. [c] Number of independent transitions.

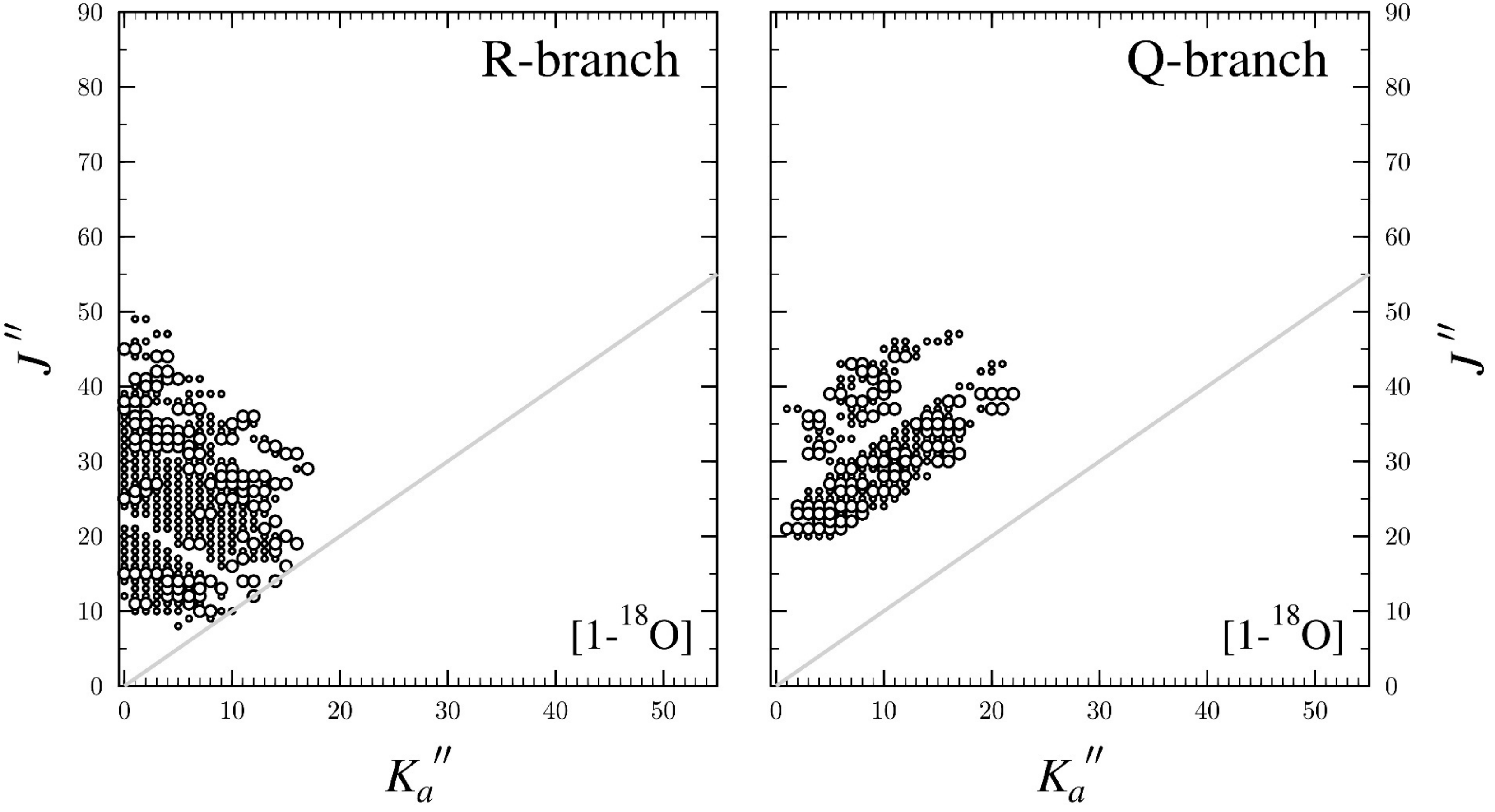

Figure S6. Data distribution plot for the least-squares fit of millimeter-wave spectroscopic data for [1-^{18}O]-oxazole, ground vibrational state. The size of the outlined circle is proportional to the value of $|(f_{obs.} - f_{calc.})/\delta f|$, where δf is the frequency measurement uncertainty (50 kHz), and no quotient values are larger than three.

Table S7. Experimental and computed spectroscopic constants for [2-^{2}H]-oxazole [a]

	S Reduction, III^r representation			A Reduction, I^r representation	
	Experimental	CCSD(T) [b]		Experimental	CCSD(T) [b]
A_0 (MHz)	9649.659 500 (87)	9584	A_0 (MHz)	9649.657 485 (89)	9584
B_0 (MHz)	9207.276 481 (76)	9183	B_0 (MHz)	9207.279 049 (74)	9183
C_0 (MHz)	4709.223 406 (89)	4687	C_0 (MHz)	4709.222 927 (88)	4687
D_J (kHz)	3.359 930 (67)	3.29	Δ_J (kHz)	1.684 784 (73)	1.66
D_{JK} (kHz)	−5.264 449 (79)	−5.14	Δ_{JK} (kHz)	−0.042 70 (17)	−0.058 1
D_K (kHz)	2.267 241 (61)	2.21	Δ_K (kHz)	1.940 08 (20)	1.89
d_1 (kHz)	−0.143 790 (44)	−0.133	δ_J (kHz)	0.661 065 (19)	0.651
d_2 (kHz)	0.032 709 (17)	0.032 2	δ_K (kHz)	1.340 042 (52)	1.30
H_J (Hz)	0.001 278 (17)	0.001 34	Φ_J (Hz)	0.000 323 (19)	0.000 431
H_{JK} (Hz)	−0.005 517 (15)	−0.005 59	Φ_{JK} (Hz)	0.001 079 (69)	0.000 751
H_{KJ} (Hz)	0.007 026 (24)	0.007 14	Φ_{KJ} (Hz)	−0.005 04 (12)	−0.004 53
H_K (Hz)	−0.002 886 (19)	−0.002 90	Φ_K (Hz)	0.005 325 (72)	0.005 04
h_1 (Hz)	[0.000 222]	0.000 222	ϕ_J (Hz)	[0.000 218]	0.000 218
h_2 (Hz)	[−0.000 0324]	−0.000 032 4	ϕ_{JK} (Hz)	[0.000 640]	0.000 640
h_3 (Hz)	[−0.000 0159]	−0.000 015 9	ϕ_K (Hz)	[0.002 85]	0.002 85
N_{lines} [c]	2244		N_{lines} [c]	2244	
σ_{fit} (MHz)	0.033		σ_{fit} (MHz)	0.033	

[a] Values in brackets are held constant at the computed values. [b] Evaluated using the cc-pCVTZ basis set. [c] Number of independent transitions.

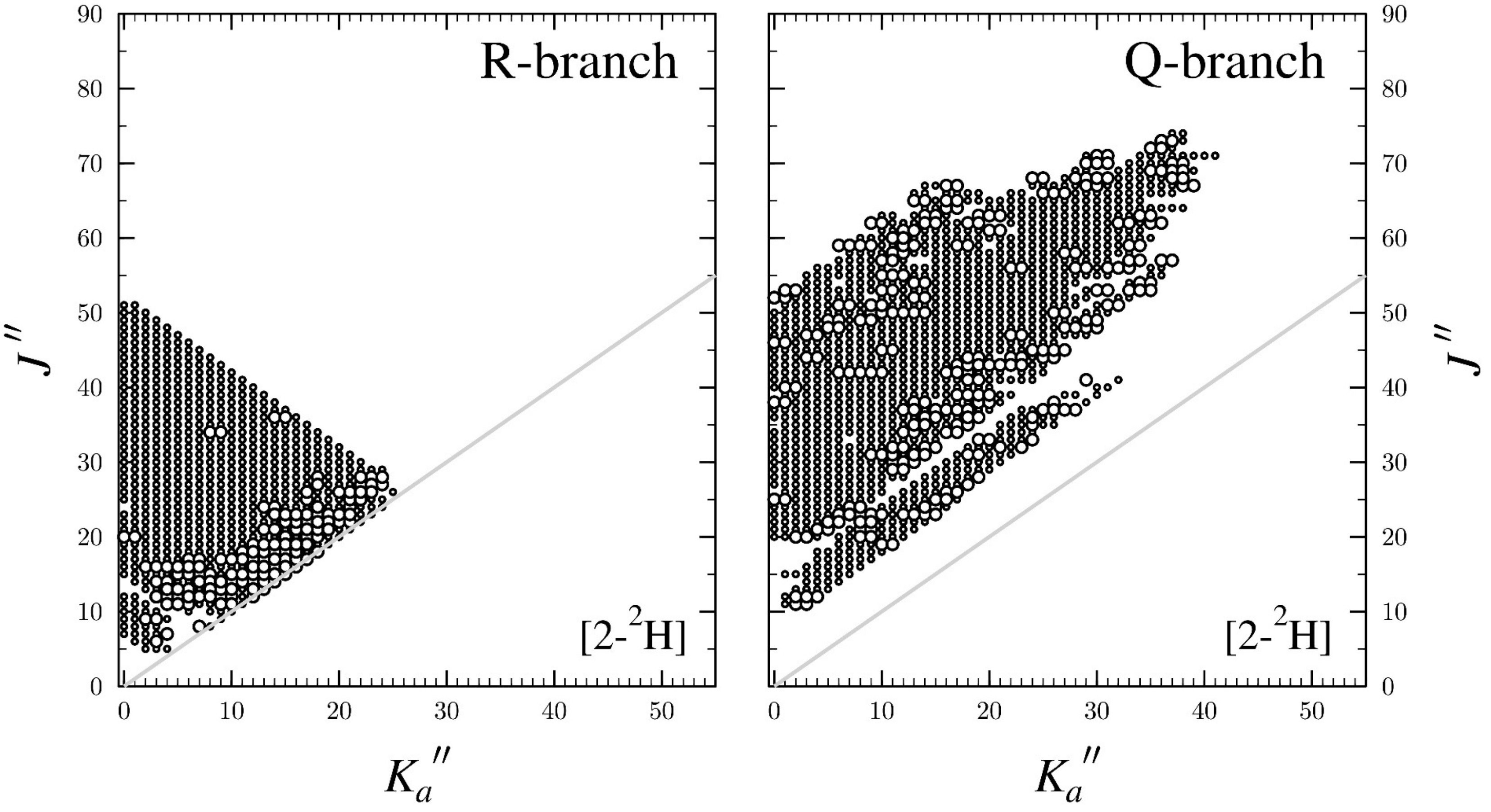

Figure S7. Data distribution plot for the least-squares fit of millimeter-wave spectroscopic data for [2-^{2}H]-oxazole, ground vibrational state. The size of the outlined circle is proportional to the value of $|(f_{obs.} - f_{calc.})/\delta f|$, where δf is the frequency measurement uncertainty (50 kHz), and no quotient values are larger than three.

Table S8. Experimental and computed spectroscopic constants for [4-^{2}H]-oxazole [a]

	S Reduction, III^r representation			A Reduction, I^r representation	
	Experimental	CCSD(T) [b]		Experimental	CCSD(T) [b]
A_0 (MHz)	9801.517 2 (10)	9756	A_0 (MHz)	9801.514 7 (10)	9756
B_0 (MHz)	9005.691 63 (94)	8962	B_0 (MHz)	9005.694 26 (94)	8962
C_0 (MHz)	4690.937 16 (14)	4668	C_0 (MHz)	4690.936 92 (14)	4668
D_J (kHz)	3.383 49 (26)	3.31	Δ_J (kHz)	1.665 01 (82)	1.63
D_{JK} (kHz)	−5.290 25 (39)	−5.17	Δ_{JK} (kHz)	−0.333 0 (43)	−0.323
D_K (kHz)	2.268 68 (20)	2.21	Δ_K (kHz)	2.599 0 (32)	2.52
d_1 (kHz)	−0.240 75 (72)	−0.230	δ_J (kHz)	0.651 55 (41)	0.640
d_2 (kHz)	−0.033 04 (32)	−0.031 2	δ_K (kHz)	1.250 58 (97)	1.23
H_J (Hz)	[0.001 32]	0.001 32	Φ_J (Hz)	[0.000 655]	0.000 655
H_{JK} (Hz)	[−0.005 58]	−0.005 58	Φ_{JK} (Hz)	[−0.000 036 6]	−0.000 036 6
H_{KJ} (Hz)	[0.007 19]	0.007 19	Φ_{KJ} (Hz)	[−0.005 40]	−0.005 40
H_K (Hz)	[−0.002 94]	−0.002 94	Φ_K (Hz)	[0.006 43]	0.006 43
h_1 (Hz)	[0.000 062 8]	0.000 062 8	ϕ_J (Hz)	[0.000 330]	0.000 330
h_2 (Hz)	[0.000 079 5]	0.000 079 5	ϕ_{JK} (Hz)	[0.000 608]	0.000 608
h_3 (Hz)	[0.000 021 0]	0.000 021 0	ϕ_K (Hz)	[0.002 97]	0.002 97
N_{lines} [c]	295		N_{lines} [c]	295	
σ_{fit} (MHz)	0.041		σ_{fit} (MHz)	0.041	

[a] Values in brackets are held constant at the computed values. [b] Evaluated using the cc-pCVTZ basis set. [c] Number of independent transitions.

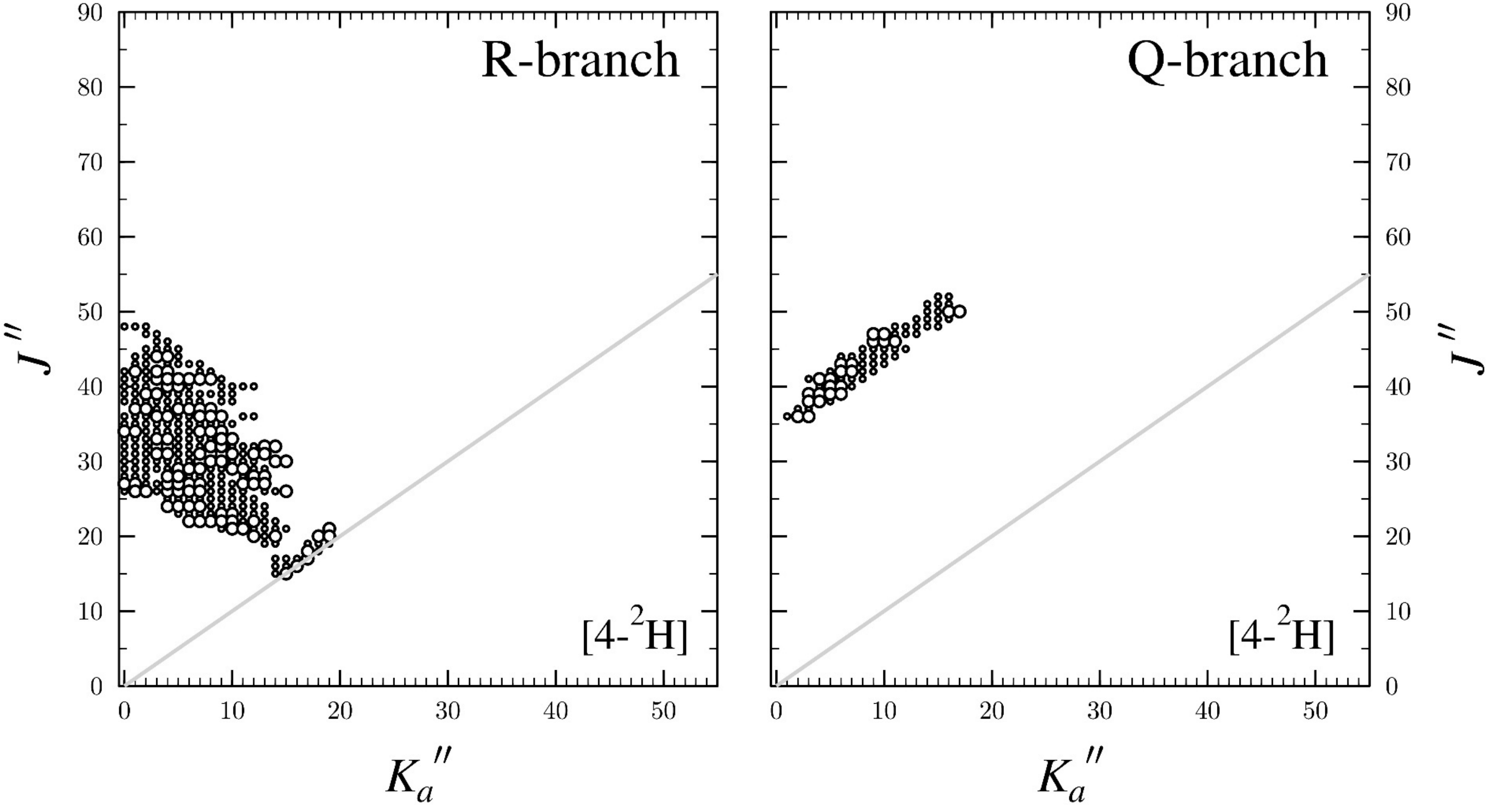

Figure S8. Data distribution plot for the least-squares fit of millimeter-wave spectroscopic data for [4-^{2}H]-oxazole, ground vibrational state. The size of the outlined circle is proportional to the value of $|(f_{obs.} - f_{calc.})/\delta f|$, where δf is the frequency measurement uncertainty (50 kHz), and no quotient values are larger than three.

Table S9. Experimental and computed spectroscopic constants for [5-^{2}H]-oxazole [a]

	S Reduction, III^r representation			A Reduction, I^r representation	
	Experimental	CCSD(T) [b]		Experimental	CCSD(T) [b]
A_0 (MHz)	9884.523 81 (12)	9843	A_0 (MHz)	9884.521 28 (13)	9843
B_0 (MHz)	8959.632 117 (97)	8912	B_0 (MHz)	8959.634 771 (97)	8912
C_0 (MHz)	4697.285 37 (10)	4674	C_0 (MHz)	4697.285 15 (10)	4674
D_J (kHz)	3.356 338 (87)	3.29	Δ_J (kHz)	1.764 439 (84)	1.72
D_{JK} (kHz)	−5.243 43 (12)	−5.13	Δ_{JK} (kHz)	−0.741 17 (22)	−0.705
D_K (kHz)	2.250 706 (82)	2.19	Δ_K (kHz)	2.706 44 (33)	2.65
d_1 (kHz)	−0.141 109 (61)	−0.145	δ_J (kHz)	0.700 404 (23)	0.683
d_2 (kHz)	−0.045 572 (13)	−0.045 6	δ_K (kHz)	1.242 736 (82)	1.21
H_J (Hz)	0.001 335 (19)	0.001 35	Φ_J (Hz)	0.000 633 (21)	0.000 650
H_{JK} (Hz)	−0.005 834 (26)	−0.005 60	Φ_{JK} (Hz)	−0.000 442 (82)	−0.000 445
H_{KJ} (Hz)	0.007 576 (43)	0.007 15	Φ_{KJ} (Hz)	−0.004 03 (28)	−0.003 68
H_K (Hz)	−0.003 103 (30)	−0.002 91	Φ_K (Hz)	0.005 50 (39)	0.005 12
h_1 (Hz)	[0.000 096 6]	0.000 096 6	ϕ_J (Hz)	[0.000 328]	0.000 328
h_2 (Hz)	[0.000 062 7]	0.000 062 7	ϕ_{JK} (Hz)	[0.000 296]	0.000 296
h_3 (Hz)	[−0.000 012 8]	−0.000 012 8	ϕ_K (Hz)	0.003 72 (13)	0.003 52
N_{lines} [c]	1645		N_{lines} [c]	1645	
σ_{fit} (MHz)	0.034		σ_{fit} (MHz)	0.034	

[a] Values in brackets are held constant at the computed values. [b] Evaluated using the cc-pCVTZ basis set. [c] Number of independent transitions.

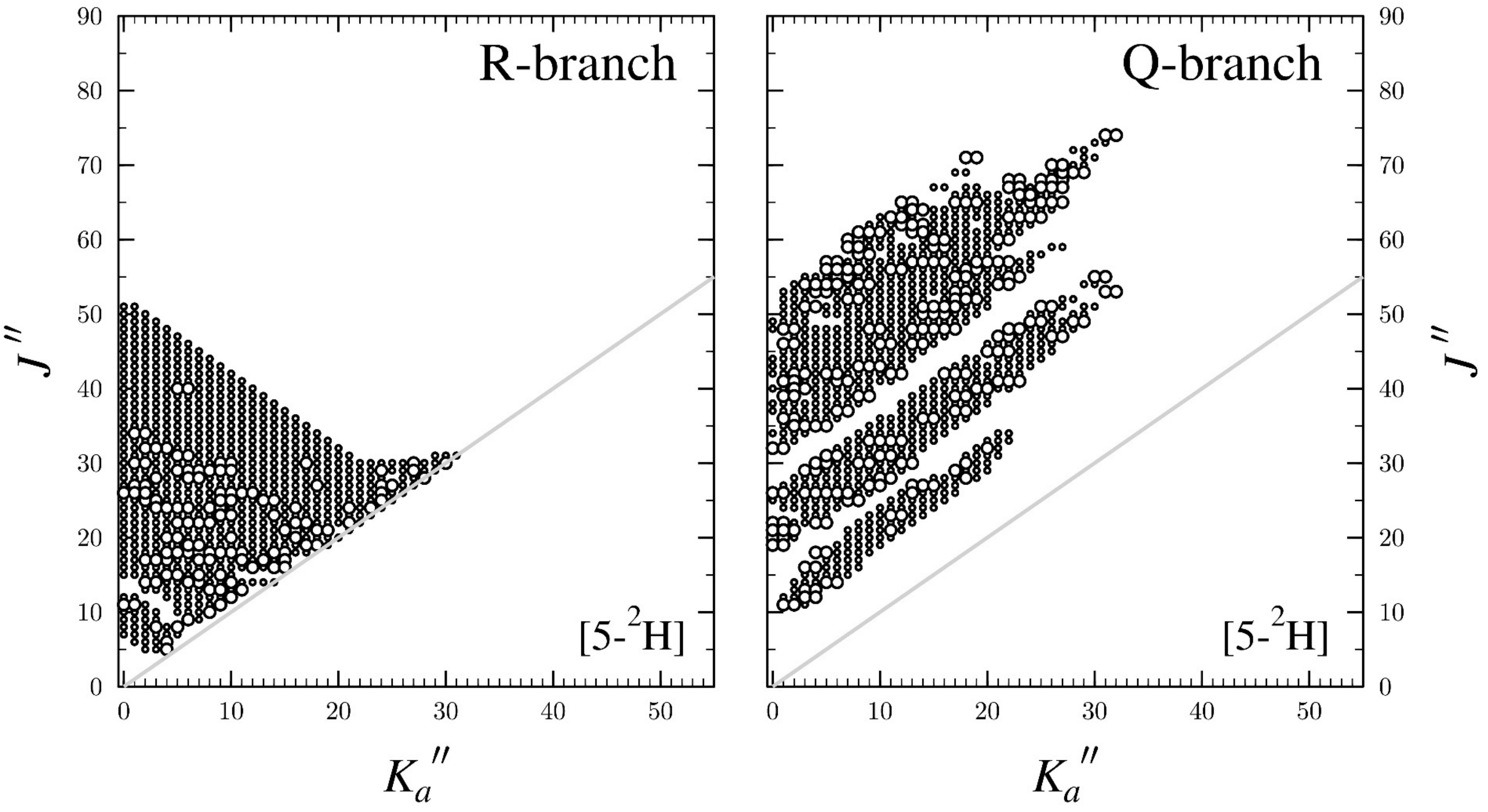

Figure S9. Data distribution plot for the least-squares fit of millimeter-wave spectroscopic data for [5-^{2}H]-oxazole, ground vibrational state. The size of the outlined circle is proportional to the value of $|(f_{obs.} - f_{calc.})/\delta f|$, where δf is the frequency measurement uncertainty (50 kHz), and no quotient values are larger than three.

Table S10. Experimental and computed spectroscopic constants for [2-^{2}H, 2-^{13}C]-oxazole [a]

	S Reduction, III^{r} representation			A Reduction, I^{r} representation	
	Experimental	CCSD(T) [b]		Experimental	CCSD(T) [b]
A_0 (MHz)	9650.277 51 (62)	9584	A_0 (MHz)	9650.275 41 (62)	9584
B_0 (MHz)	9027.932 94 (71)	9004	B_0 (MHz)	9027.935 04 (84)	9004
C_0 (MHz)	4661.985 74 (26)	4640	C_0 (MHz)	4661.984 60 (11)	4640
D_J (kHz)	3.296 02 (15)	3.23	Δ_J (kHz)	1.634 46 (71)	1.61
D_{JK} (kHz)	−5.157 63 (48)	−5.04	Δ_{JK} (kHz)	−0.017 7 (34)	−0.033 6
D_K (kHz)	2.217 76 (40)	2.16	Δ_K (kHz)	1.956 6 (26)	1.91
d_1 (kHz)	−0.164 91 (45)	−0.155	δ_J (kHz)	0.639 47 (35)	0.629
d_2 (kHz)	0.026 04 (24)	0.025 1	δ_K (kHz)	1.341 59 (75)	1.30
H_J (Hz)	[0.001 30]	0.001 30	Φ_J (Hz)	[0.000 441]	0.000 441
H_{JK} (Hz)	−0.005 03 (19)	−0.005 41	Φ_{JK} (Hz)	[0.000 657]	0.000 657
H_{KJ} (Hz)	0.006 67 (19)	0.006 91	Φ_{KJ} (Hz)	−0.005 01 (42)	−0.004 52
H_K (Hz)	[−0.002 81]	−0.002 81	Φ_K (Hz)	[0.005 08]	0.005 08
h_1 (Hz)	[0.000 206]	0.000 206	ϕ_J (Hz)	[0.000 223]	0.000 223
h_2 (Hz)	[−0.000 016 1]	−0.000 016 1	ϕ_{JK} (Hz)	[0.000 626]	0.000 626
h_3 (Hz)	[−0.000 013 7]	−0.000 013 7	ϕ_K (Hz)	[0.002 88]	0.002 88
N_{lines} [c]	403		N_{lines} [c]	403	
σ_{fit} (MHz)	0.039		σ_{fit} (MHz)	0.040	

[a] Values in brackets are held constant at the computed values. [b] Evaluated using the cc-pCVTZ basis set. [c] Number of independent transitions.

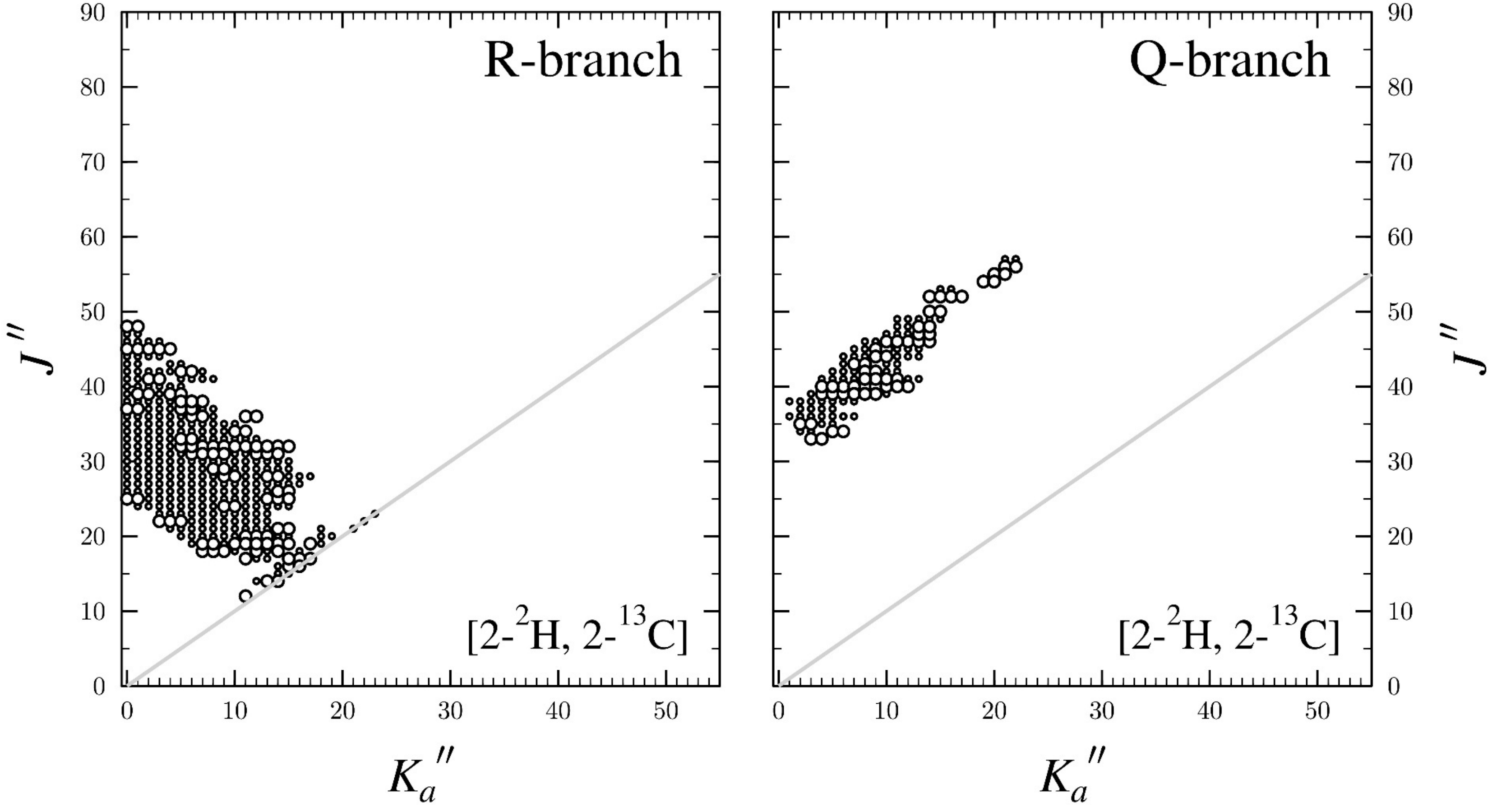

Figure S10. Data distribution plot for the least-squares fit of millimeter-wave spectroscopic data for [2-^{2}H, 2-^{13}C]-oxazole, ground vibrational state. The size of the outlined circle is proportional to the value of $|(f_{obs.} - f_{calc.})/\delta f|$, where δf is the frequency measurement uncertainty (50 kHz), and no quotient values are larger than three.

Table S11. Experimental and computed spectroscopic constants for [2-^{2}H, 4-^{13}C]-oxazole [a]

	S Reduction, III^r representation			A Reduction, I^r representation	
	Experimental	CCSD(T) [b]		Experimental	CCSD(T) [b]
A_0 (MHz)	9580.090 9 (15)	9515	A_0 (MHz)	9580.089 3 (15)	9515
B_0 (MHz)	9036.856 1 (13)	9012	B_0 (MHz)	9036.857 7 (13)	9012
C_0 (MHz)	4647.895 80 (30)	4626	C_0 (MHz)	4647.894 89 (11)	4626
D_J (kHz)	3.283 60 (63)	3.21	Δ_J (kHz)	1.623 2 (19)	1.60
D_{JK} (kHz)	−5.141 16 (85)	−5.02	Δ_{JK} (kHz)	−0.115 3 (88)	−0.169
D_K (kHz)	2.211 82 (26)	2.16	Δ_K (kHz)	2.135 6 (60)	2.11
d_1 (kHz)	−0.186 8 (16)	−0.172	δ_J (kHz)	0.634 73 (96)	0.628
d_2 (kHz)	0.007 43 (58)	0.004 45	δ_K (kHz)	1.264 4 (17)	1.21
H_J (Hz)	0.001 36 (15)	0.001 29	Φ_J (Hz)	[0.000 466]	0.000 466
H_{JK} (Hz)	−0.005 30 (15)	−0.005 38	Φ_{JK} (Hz)	[0.000 612]	0.000 612
H_{KJ} (Hz)	[0.006 88]	0.006 88	Φ_{KJ} (Hz)	−0.005 25 (53)	−0.004 76
H_K (Hz)	[−0.002 80]	−0.002 80	Φ_K (Hz)	[0.005 31]	0.005 31
h_1 (Hz)	[0.000 175]	0.000 175	ϕ_J (Hz)	[0.000 235]	0.000 235
h_2 (Hz)	[−0.000 005 40]	−0.000 005 40	ϕ_{JK} (Hz)	[0.000 645]	0.000 645
h_3 (Hz)	[−0.000 001 30]	−0.000 001 30	ϕ_K (Hz)	[0.002 75]	0.002 75
N_{lines} [c]	370		N_{lines} [c]	370	
σ_{fit} (MHz)	0.040		σ_{fit} (MHz)	0.040	

[a] Values in brackets are held constant at the computed values. [b] Evaluated using the cc-pCVTZ basis set. [c] Number of independent transitions.

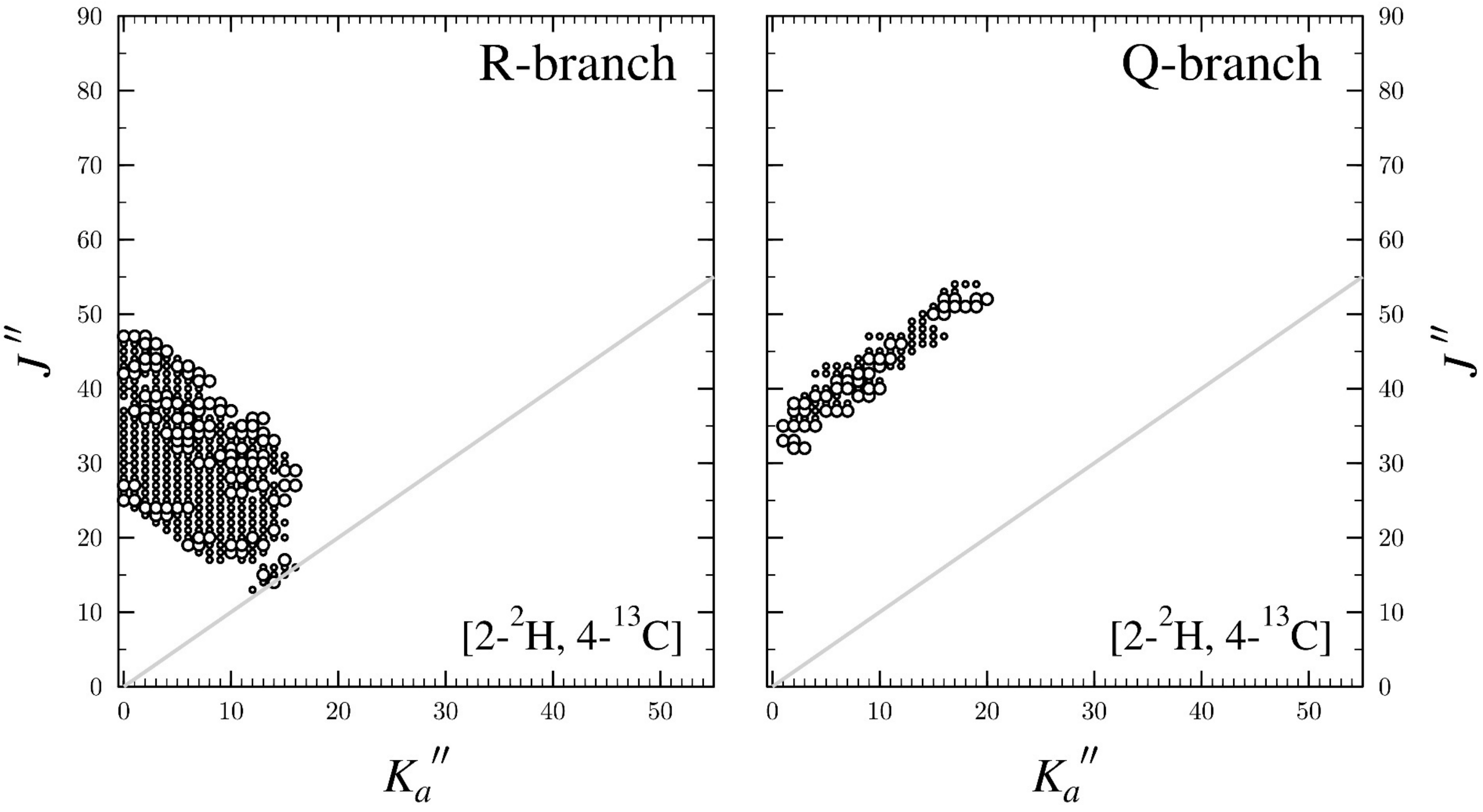

Figure S11. Data distribution plot for the least-squares fit of millimeter-wave spectroscopic data for [2-^{2}H, 4-^{13}C]-oxazole, ground vibrational state. The size of the outlined circle is proportional to the value of $|(f_{obs.} - f_{calc.})/\delta f|$, where δf is the frequency measurement uncertainty (50 kHz), and no quotient values are larger than three.

Table S12. Experimental and computed spectroscopic constants for [2-^{2}H, 5-^{13}C]-oxazole [a]

	S Reduction, III^r representation			A Reduction, I^r representation	
	Experimental	CCSD(T) [b]		Experimental	CCSD(T) [b]
A_0 (MHz)	9602.779 28 (70)	9540	A_0 (MHz)	9602.777 43 (72)	9540
B_0 (MHz)	9025.216 36 (65)	8999	B_0 (MHz)	9025.219 27 (76)	8999
C_0 (MHz)	4650.145 24 (30)	4628	C_0 (MHz)	4650.145 36 (12)	4628
D_J (kHz)	3.275 13 (21)	3.21	Δ_J (kHz)	1.664 12 (71)	1.64
D_{JK} (kHz)	−5.130 14 (58)	−5.01	Δ_{JK} (kHz)	−0.101 5 (27)	−0.124
D_K (kHz)	2.208 81 (44)	2.15	Δ_K (kHz)	1.885 1 (22)	1.85
d_1 (kHz)	−0.118 47 (72)	−0.110	δ_J (kHz)	0.654 89 (35)	0.645
d_2 (kHz)	0.032 39 (21)	0.030 1	δ_K (kHz)	1.342 89 (62)	1.30
H_J (Hz)	[0.001 31]	0.001 31	Φ_J (Hz)	[0.000 366]	0.000 366
H_{JK} (Hz)	−0.005 81 (24)	−0.005 40	Φ_{JK} (Hz)	[0.001 55]	0.001 55
H_{KJ} (Hz)	0.007 16 (23)	0.006 88	Φ_{KJ} (Hz)	−0.005 93 (53)	−0.006 32
H_K (Hz)	[−0.002 79]	−0.002 79	Φ_K (Hz)	[0.006 04]	0.006 04
h_1 (Hz)	[0.000 214]	0.000 214	ϕ_J (Hz)	[0.000 185]	0.000 185
h_2 (Hz)	[−0.000 057 9]	−0.000 057 9	ϕ_{JK} (Hz)	[0.000 921]	0.000 921
h_3 (Hz)	[0.000 013 3]	0.000 013 3	ϕ_K (Hz)	[0.002 47]	0.002 47
N_{lines} [c]	379		N_{lines} [c]	379	
σ_{fit} (MHz)	0.040		σ_{fit} (MHz)	0.041	

[a] Values in brackets are held constant at the computed values. [b] Evaluated using the cc-pCVTZ basis set. [c] Number of independent transitions.

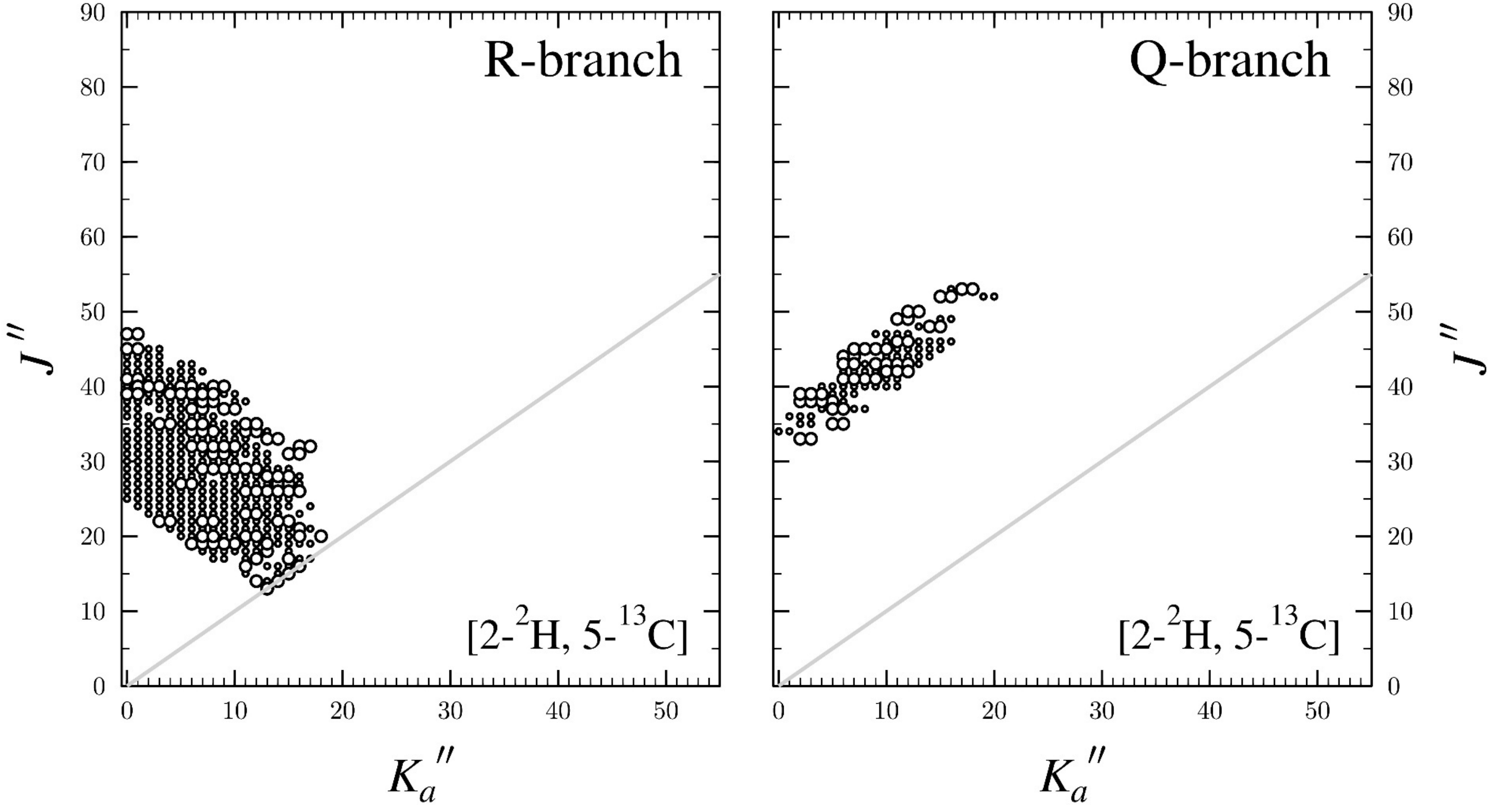

Figure S12. Data distribution plot for the least-squares fit of millimeter-wave spectroscopic data for [2-^{2}H, 5-^{13}C]-oxazole, ground vibrational state. The size of the outlined circle is proportional to the value of $|(f_{obs.} - f_{calc.})/\delta f|$, where δf is the frequency measurement uncertainty (50 kHz), and no quotient values are larger than three.

Table S13. Experimental and computed spectroscopic constants for [2-^{2}H, 3-^{15}N]-oxazole [a]

	S Reduction, III^r representation			A Reduction, I^r representation	
	Experimental	CCSD(T) [b]		Experimental	CCSD(T) [b]
A_0 (MHz)	9444.531 (23)	9385	A_0 (MHz)	9444.545 (23)	9385
B_0 (MHz)	9160.262 (23)	9130	B_0 (MHz)	9160.249 (23)	9130
C_0 (MHz)	4647.731 76 (41)	4625	C_0 (MHz)	4647.731 57 (40)	4625
D_J (kHz)	3.274 3 (19)	3.20	Δ_J (kHz)	1.716 74 (14)	1.71
D_{JK} (kHz)	−5.139 0 (42)	−5.01	Δ_{JK} (kHz)	[−0.385]	−0.385
D_K (kHz)	2.219 0 (23)	2.16	Δ_K (kHz)	2.095 4 (56)	1.90
d_1 (kHz)	−[0.038 764 1]	−0.039	δ_J (kHz)	[0.681]	0.681
d_2 (kHz)	[0.025 659 5]	0.025 7	δ_K (kHz)	1.216 58 (75)	1.21
H_J (Hz)	[0.001 294 9]	0.001 29	Φ_J (Hz)	[0.000 241]	0.000 241
H_{JK} (Hz)	[−0.005 397 2]	−0.005 40	Φ_{JK} (Hz)	[0.002 57]	0.002 57
H_{KJ} (Hz)	[0.006 893 8]	0.006 89	Φ_{KJ} (Hz)	[−0.007 96]	−0.007 96
H_K (Hz)	[−0.002 796 4]	−0.002 80	Φ_K (Hz)	[0.006 79]	0.006 79
h_1 (Hz)	[0.000 247 1]	0.000 247	ϕ_J (Hz)	[0.000 123]	0.000 123
h_2 (Hz)	[−0.000 115 7]	−0.000 116	ϕ_{JK} (Hz)	[0.001 19]	0.001 19
h_3 (Hz)	[0.000 040 8]	0.000 040 8	ϕ_K (Hz)	[0.001 95]	0.001 95
N_{lines} [c]	128		N_{lines} [c]	128	
σ_{fit} (MHz)	0.040		σ_{fit} (MHz)	0.040	

[a] Values in brackets are held constant at the computed values. [b] Evaluated using the cc-pCVTZ basis set. [c] Number of independent transitions.

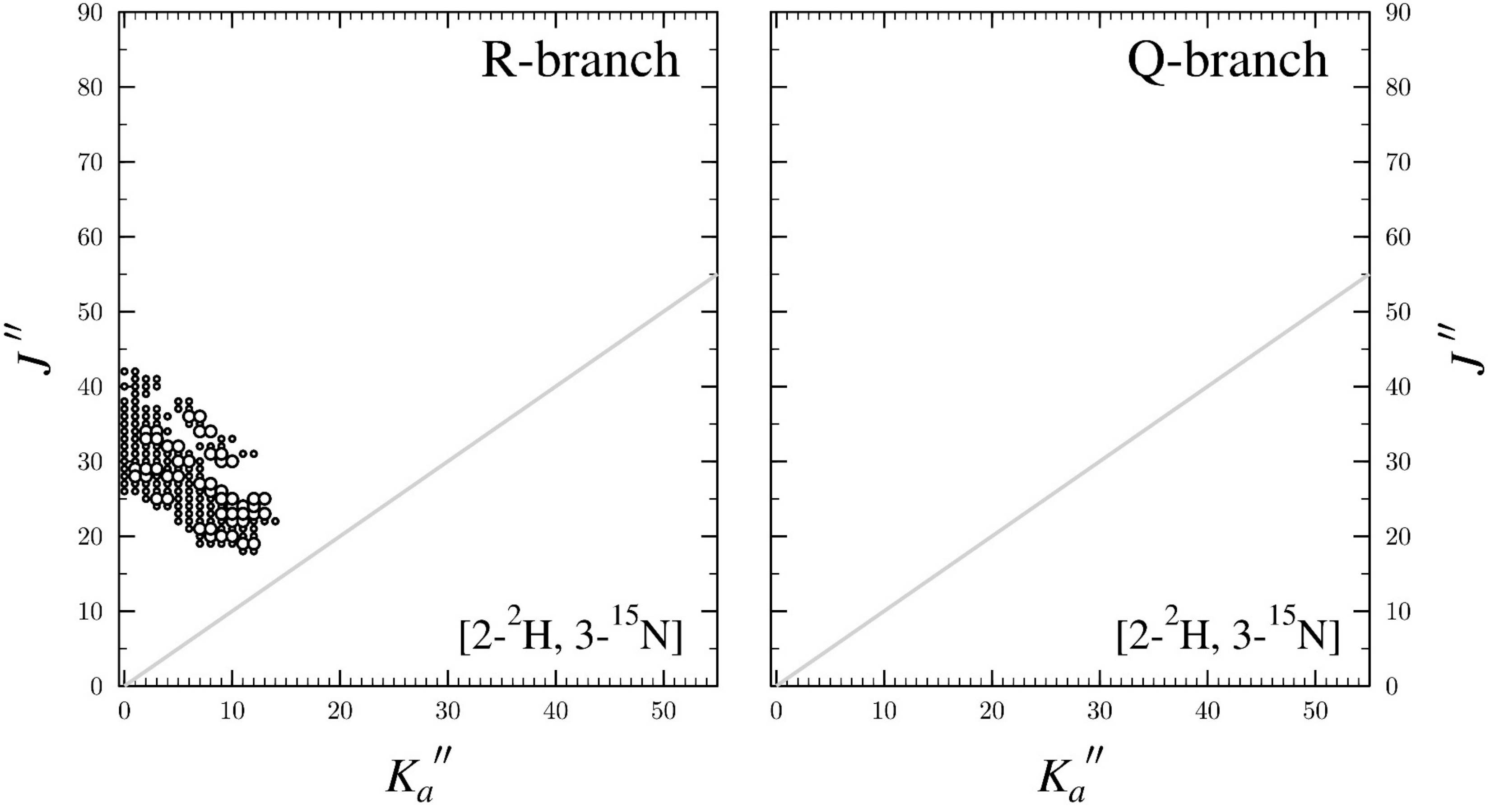

Figure S13. Data distribution plot for the least-squares fit of millimeter-wave spectroscopic data for [2-^{2}H, 3-^{15}N]-oxazole, ground vibrational state. The size of the outlined circle is proportional to the value of $|(f_{obs.} - f_{calc.})/\delta f|$, where δf is the frequency measurement uncertainty (50 kHz), and no quotient values are larger than three.

Table S14. Experimental and computed spectroscopic constants for [2-^{2}H, 1-^{18}O]-oxazole [a]

	S Reduction, III^r representation			A Reduction, I^r representation	
	Experimental	CCSD(T) [b]		Experimental	CCSD(T) [b]
A_0 (MHz)	9315.305 (23)	9264	A_0 (MHz)	9315.321 (23)	9264
B_0 (MHz)	9106.207 (23)	9069	B_0 (MHz)	9106.192 (23)	9069
C_0 (MHz)	4602.433 39 (72)	4580	C_0 (MHz)	4602.433 59 (71)	4580
D_J (kHz)	3.228 3 (26)	3.16	Δ_J (kHz)	1.684 37 (29)	1.68
D_{JK} (kHz)	−5.053 6 (55)	−4.95	Δ_{JK} (kHz)	[−0.671]	−0.671
D_K (kHz)	2.177 8 (29)	2.13	Δ_K (kHz)	2.600 3 (76)	2.44
d_1 (kHz)	[−0.109]	−0.109	δ_J (kHz)	[0.666]	0.666
d_2 (kHz)	[−0.031 2]	−0.031 2	δ_K (kHz)	1.015 11 (99)	1.02
H_J (Hz)	[0.001 23]	0.001 23	Φ_J (Hz)	[0.000 583]	0.000 583
H_{JK} (Hz)	[−0.005 19]	−0.005 19	Φ_{JK} (Hz)	[−0.000 050 6]	−0.000 050 6
H_{KJ} (Hz)	[0.006 68]	0.006 68	Φ_{KJ} (Hz)	[−0.004 65]	−0.004 65
H_K (Hz)	[−0.002 72]	−0.002 72	Φ_K (Hz)	[0.005 65]	0.005 65
h_1 (Hz)	[0.000 065 2]	0.000 065 2	ϕ_J (Hz)	[0.000 294]	0.000 294
h_2 (Hz)	[0.000 062 6]	0.000 062 6	ϕ_{JK} (Hz)	[0.000 442]	0.000 442
h_3 (Hz)	[0.000 024 9]	0.000 024 9	ϕ_K (Hz)	[0.002 30]	0.002 30
N_{lines} [c]	100		N_{lines} [c]	100	
σ_{fit} (MHz)	0.038		σ_{fit} (MHz)	0.038	

[a] Values in brackets are held constant at the computed values. [b] Evaluated using the cc-pCVTZ basis set. [c] Number of independent transitions.

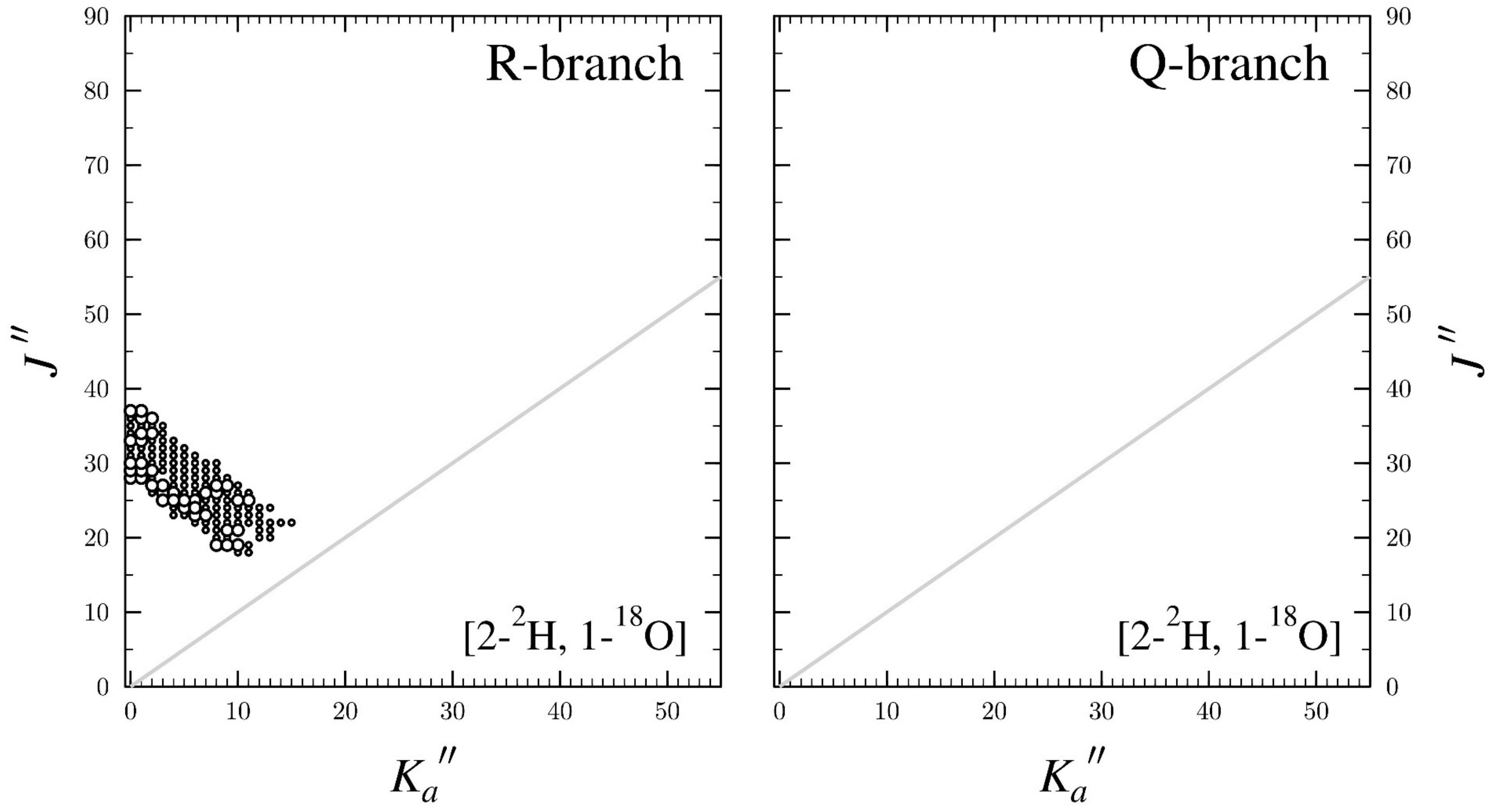

Figure S14. Data distribution plot for the least-squares fit of millimeter-wave spectroscopic data for [2-^{2}H, 1-^{18}O]-oxazole, ground vibrational state. The size of the outlined circle is proportional to the value of $|(f_{obs.} - f_{calc.})/\delta f|$, where δf is the frequency measurement uncertainty (50 kHz), and no quotient values are larger than three.

Table S15. Experimental and computed spectroscopic constants for [2,5-^{2}H]-oxazole [a]

	S Reduction, III^r representation			A Reduction, I^r representation	
	Experimental	CCSD(T) [b]		Experimental	CCSD(T) [b]
A_0 (MHz)	9530.681 737 (48)	9476	A_0 (MHz)	9530.679 712 (48)	9476
B_0 (MHz)	8536.920 327 (46)	8506	B_0 (MHz)	8536.922 824 (46)	8506
C_0 (MHz)	4501.080 919 (58)	4480	C_0 (MHz)	4501.080 442 (58)	4480
D_J (kHz)	3.002 709 (39)	2.94	Δ_J (kHz)	1.501 224 (41)	1.47
D_{JK} (kHz)	−4.686 166 (37)	−4.58	Δ_{JK} (kHz)	−0.206 601 (50)	−0.222
D_K (kHz)	2.007 219 (29)	1.96	Δ_K (kHz)	2.048 706 (48)	2.02
d_1 (kHz)	−0.166 160 (10)	−0.160	δ_J (kHz)	0.588 734 5 (77)	0.579
d_2 (kHz)	−0.004 150 6 (38)	−0.006 2	δ_K (kHz)	1.239 323 (21)	1.20
H_J (Hz)	0.001 166 4 (96)	0.001 19	Φ_J (Hz)	0.000 376 8 (98)	0.000 406
H_{JK} (Hz)	−0.004 874 4 (87)	−0.004 79	Φ_{JK} (Hz)	0.000 899 (21)	0.000 886
H_{KJ} (Hz)	0.006 199 (11)	0.006 02	Φ_{KJ} (Hz)	−0.005 459 (36)	−0.005 24
H_K (Hz)	−0.002 516 3 (76)	−0.002 43	Φ_K (Hz)	0.005 861 (22)	0.005 62
h_1 (Hz)	0.000 206 1 (27)	0.000 196 2	ϕ_J (Hz)	0.000 201 5 (16)	0.000 205
h_2 (Hz)	0.000 030 6 (16)	0.000 023 9	ϕ_{JK} (Hz)	0.000 700 1 (73)	0.000 702
h_3 (Hz)	0.000 017 75 (44)	0.000 017 5	ϕ_K (Hz)	0.002 611 6 (81)	0.002 50
N_{lines} [c]	3958		N_{lines} [c]	3958	
σ_{fit} (MHz)	0.029		σ_{fit} (MHz)	0.029	

[a] Values in brackets are held constant at the computed values. [b] Evaluated using the cc-pCVTZ basis set. [c] Number of independent transitions.

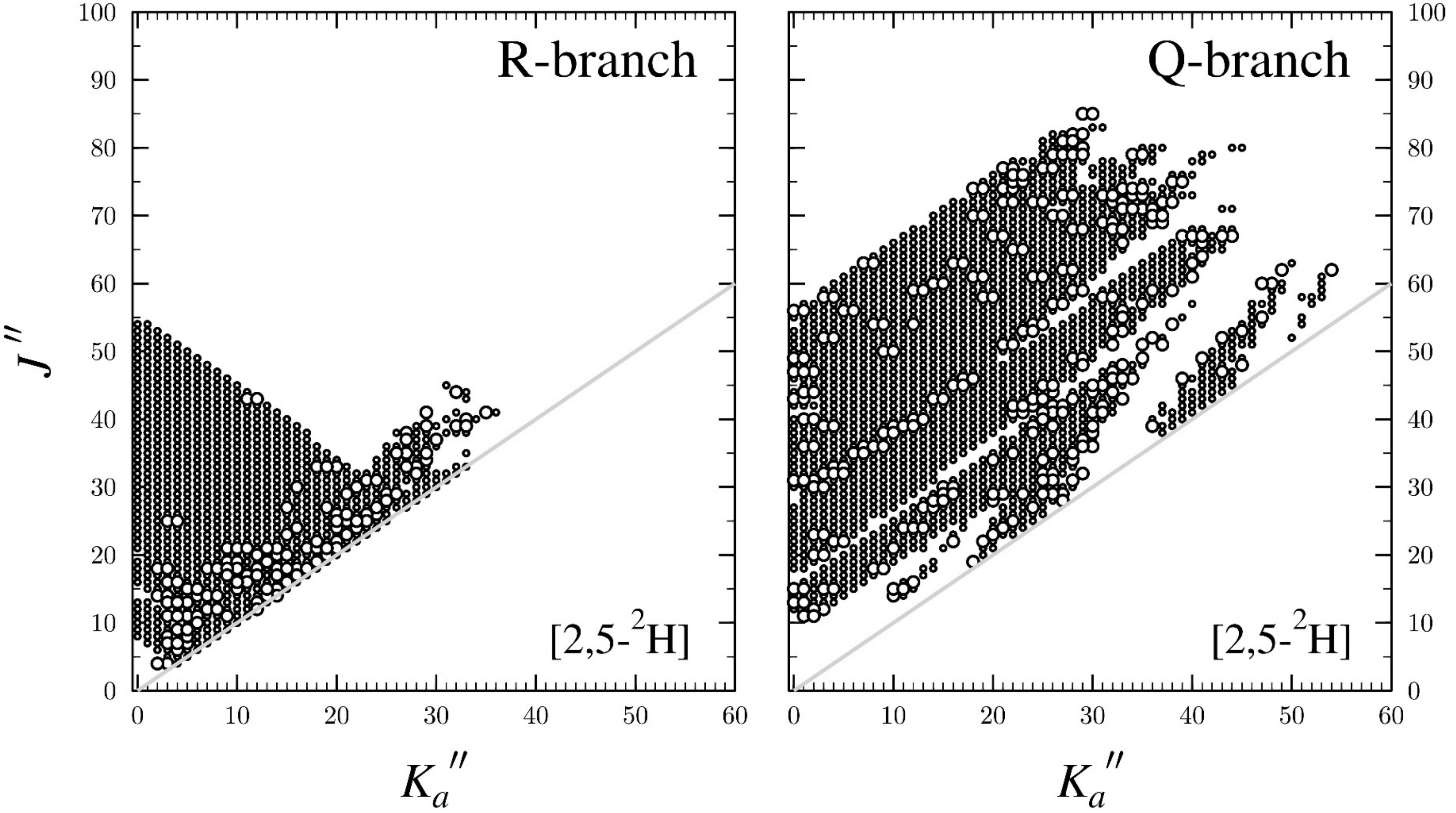

Figure S15. Data distribution plot for the least-squares fit of millimeter-wave spectroscopic data for [2,5-^{2}H]-oxazole, ground vibrational state. The size of the outlined circle is proportional to the value of $|(f_{obs.} - f_{calc.})/\delta f|$, where δf is the frequency measurement uncertainty (50 kHz), and no quotient values are larger than three.

Table S16. Experimental and computed spectroscopic constants for [2,5-^{2}H, 2-^{13}C]-oxazole [a]

	S Reduction, III^r representation			A Reduction, I^r representation	
	Experimental	CCSD(T) [b]		Experimental	CCSD(T) [b]
A_0 (MHz)	9504.021 61 (40)	9448	A_0 (MHz)	9504.020 20 (44)	9448
B_0 (MHz)	8396.067 62 (30)	8367	B_0 (MHz)	8396.069 82 (25)	8367
C_0 (MHz)	4455.751 99 (17)	4435	C_0 (MHz)	4455.751 679 (81)	4435
D_J (kHz)	2.945 69 (31)	2.88	Δ_J (kHz)	1.446 01 (12)	1.42
D_{JK} (kHz)	−4.590 10 (66)	−4.49	Δ_{JK} (kHz)	−0.091 93 (55)	−0.103
D_K (kHz)	1.961 49 (38)	1.91	Δ_K (kHz)	1.964 42 (90)	1.93
d_1 (kHz)	−0.185 21 (16)	−0.179	δ_J (kHz)	0.564 358 (60)	0.554
d_2 (kHz)	−0.000 092 (52) [c]	−0.001 24	δ_K (kHz)	1.255 90 (19)	1.22
H_J (Hz)	0.001 59 (11)	0.001 16	Φ_J (Hz)	[0.000 375]	0.000 375
H_{JK} (Hz)	−0.006 06 (37)	−0.004 63	Φ_{JK} (Hz)	[0.001 01]	0.001 01
H_{KJ} (Hz)	0.007 25 (43)	0.005 81	Φ_{KJ} (Hz)	−0.005 95 (36)	−0.005 31
H_K (Hz)	−0.002 83 (17)	−0.002 35	Φ_K (Hz)	0.009 06 (88)	0.005 57
h_1 (Hz)	[0.000 204]	0.000 204	ϕ_J (Hz)	[0.000 190]	0.000 190
h_2 (Hz)	[0.000 018 7]	0.000 018 7	ϕ_{JK} (Hz)	[0.000 741]	0.000 741
h_3 (Hz)	[0.000 017 0]	0.000 017 0	ϕ_K (Hz)	[0.002 49]	0.002 49
N_{lines} [d]	668		N_{lines} [d]	668	
σ_{fit} (MHz)	0.039		σ_{fit} (MHz)	0.039	

[a] Values in brackets are held constant at the computed values. [b] Evaluated using the cc-pCVTZ basis set. [c] Although the uncertainty in d_2 is over half the magnitude of the term itself, holding this value constant at its predicted value resulted in an increase σ_{fit} and tens of transitions being unable to be fitted, therefore, this term was allowed to vary and is expected to be physically meaningful despite the large uncertainty. [d] Number of independent transitions.

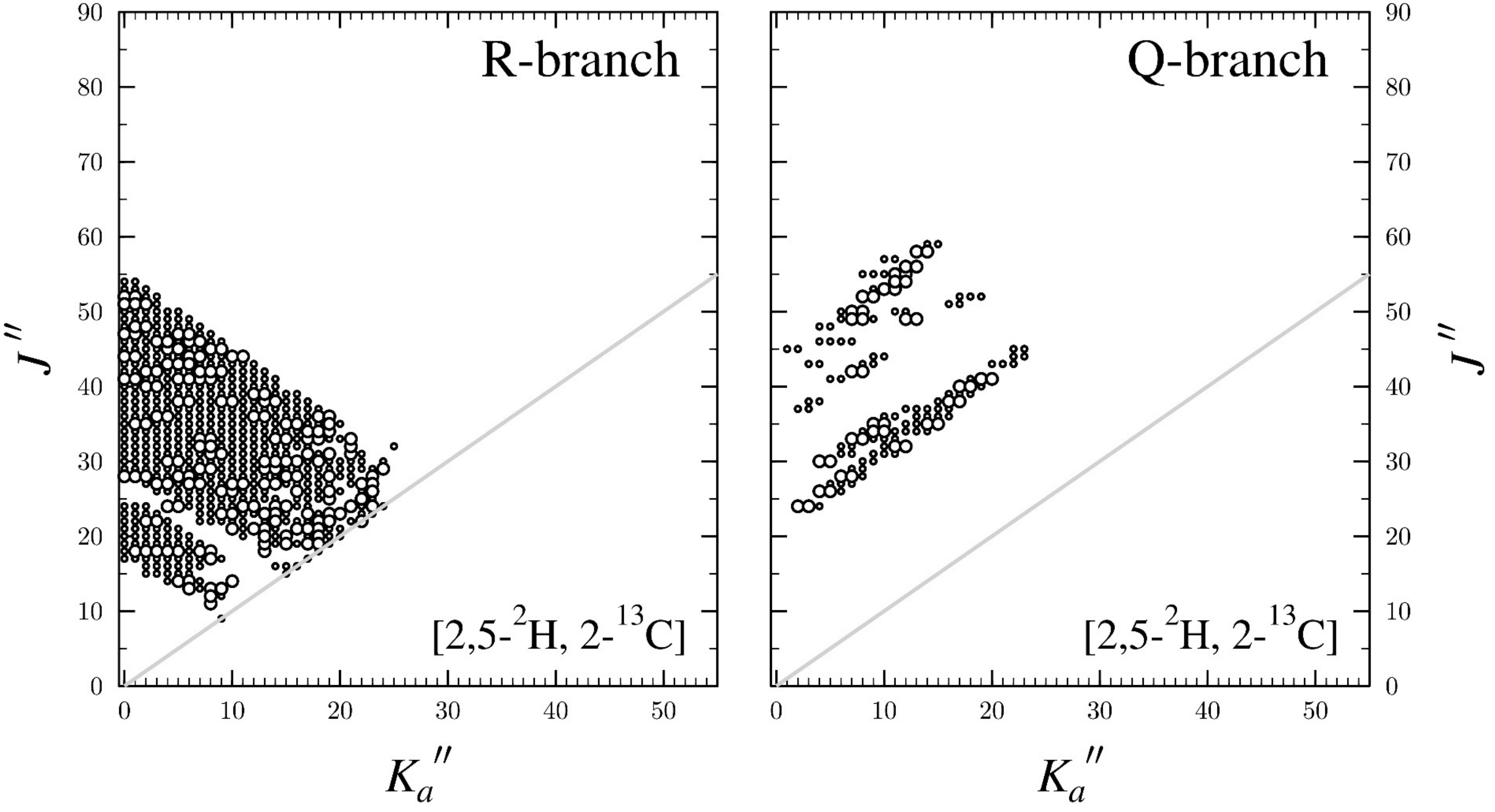

Figure S16. Data distribution plot for the least-squares fit of millimeter-wave spectroscopic data for [2,5-^{2}H, 2-^{13}C]-oxazole, ground vibrational state. The size of the outlined circle is proportional to the value of $|(f_{obs.} - f_{calc.})/\delta f|$, where δf is the frequency measurement uncertainty (50 kHz), and no quotient values are larger than three.

Table S17. Experimental and computed spectroscopic constants for [2,5-^{2}H, 4-^{13}C]-oxazole [a]

	S Reduction, III^r representation			A Reduction, I^r representation	
	Experimental	CCSD(T) [b]		Experimental	CCSD(T) [b]
A_0 (MHz)	9338.627 63 (27)	9282	A_0 (MHz)	9338.625 18 (30)	9282
B_0 (MHz)	8492.944 78 (21)	8464	B_0 (MHz)	8492.947 37 (19)	8464
C_0 (MHz)	4445.739 96 (14)	4425	C_0 (MHz)	4445.739 593 (85)	4425
D_J (kHz)	2.931 94 (20)	2.87	Δ_J (kHz)	1.461 726 (94)	1.44
D_{JK} (kHz)	−4.583 86 (39)	−4.48	Δ_{JK} (kHz)	−0.116 84 (43)	−0.137
D_K (kHz)	1.967 34 (23)	1.92	Δ_K (kHz)	1.872 20 (60)	1.85
d_1 (kHz)	−0.152 932 (99)	−0.146	δ_J (kHz)	0.573 115 (45)	0.564
d_2 (kHz)	0.009 329 (36)	0.007 25	δ_K (kHz)	1.215 42 (14)	1.17
H_J (Hz)	0.000 817 (57)	0.001 16	Φ_J (Hz)	[0.000 356]	0.000 356
H_{JK} (Hz)	−0.003 84 (15)	−0.004 64	Φ_{JK} (Hz)	0.001 40 (17)	0.001 17
H_{KJ} (Hz)	0.005 36 (10)	0.005 84	Φ_{KJ} (Hz)	−0.006 58 (38)	−0.005 52
H_K (Hz)	[−0.002 36]	−0.002 36	Φ_K (Hz)	0.004 13 (47)	0.005 58
h_1 (Hz)	[0.000 197]	0.000 197	ϕ_J (Hz)	[0.000 180]	0.000 180
h_2 (Hz)	[−0.000 002 10]	−0.000 002 10	ϕ_{JK} (Hz)	[0.000 786]	0.000 786
h_3 (Hz)	[0.000 021 2]	0.000 021 2	ϕ_K (Hz)	[0.002 23]	0.002 23
N_{lines} [c]	779		N_{lines} [c]	779	
σ_{fit} (MHz)	0.036		σ_{fit} (MHz)	0.036	

[a] Values in brackets are held constant at the computed values. [b] Evaluated using the cc-pCVTZ basis set. [c] Number of independent transitions.

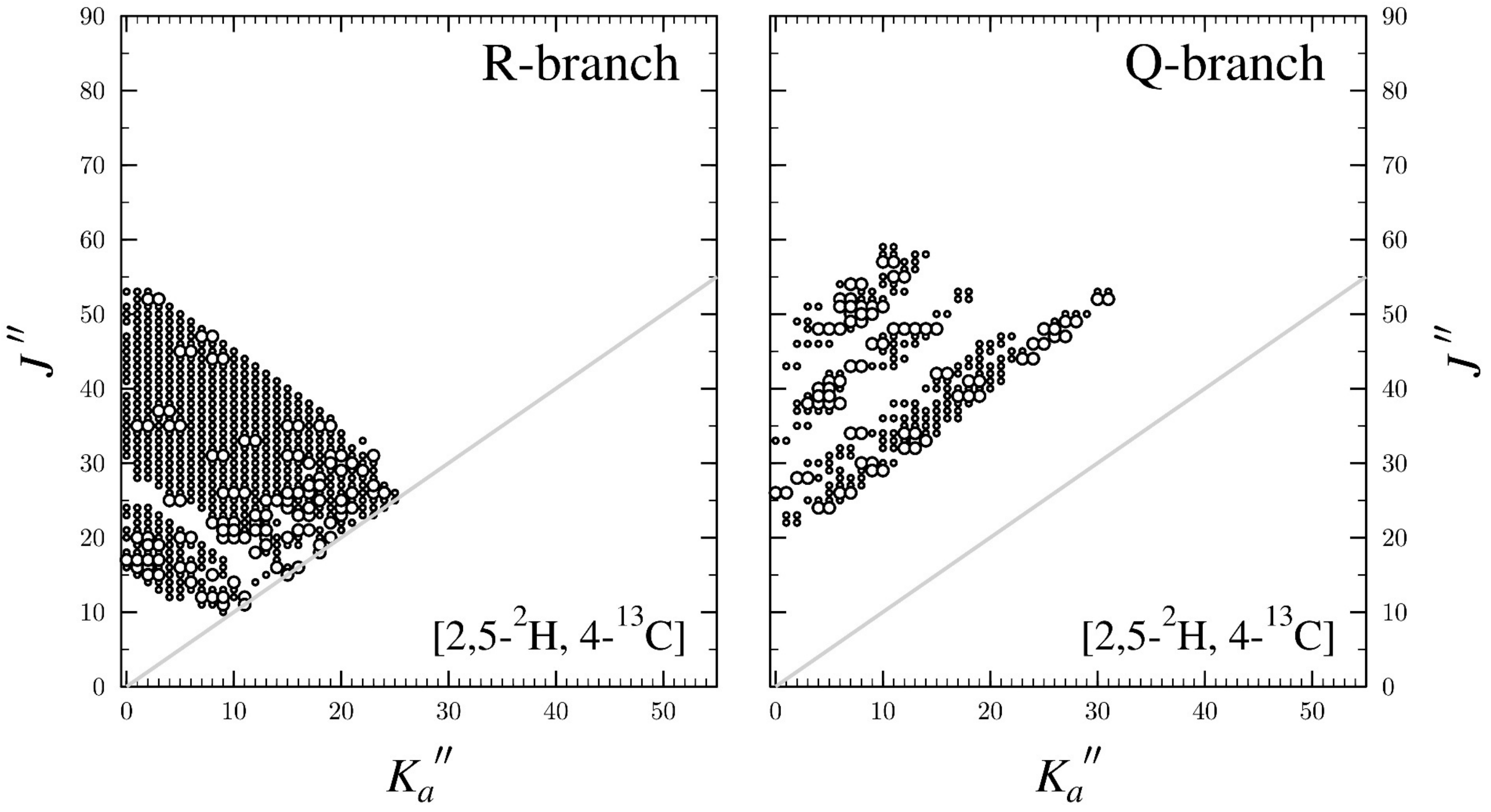

Figure S17. Data distribution plot for the least-squares fit of millimeter-wave spectroscopic data for [2,5-^{2}H, 4-^{13}C]-oxazole, ground vibrational state. The size of the outlined circle is proportional to the value of $|(f_{obs.} - f_{calc.})/\delta f|$, where δf is the frequency measurement uncertainty (50 kHz), and no quotient values are larger than three.

Table S18. Experimental and computed spectroscopic constants for [2,5-^{2}H, 5-^{13}C]-oxazole [a]

	S Reduction, III^r representation			A Reduction, I^r representation	
	Experimental	CCSD(T) [b]		Experimental	CCSD(T) [b]
A_0 (MHz)	9529.028 18 (31)	9475	A_0 (MHz)	9529.025 87 (34)	9475
B_0 (MHz)	8356.212 36 (24)	8325	B_0 (MHz)	8356.214 95 (20)	8325
C_0 (MHz)	4449.962 86 (15)	4429	C_0 (MHz)	4449.962 512 (74)	4429
D_J (kHz)	2.939 97 (25)	2.88	Δ_J (kHz)	1.454 582 (89)	1.43
D_{JK} (kHz)	−4.574 69 (48)	−4.47	Δ_{JK} (kHz)	−0.192 13 (39)	−0.207
D_K (kHz)	1.951 94 (29)	1.91	Δ_K (kHz)	2.074 34 (65)	2.05
d_1 (kHz)	−0.186 56 (12)	−0.181	δ_J (kHz)	0.568 588 (44)	0.559
d_2 (kHz)	−0.012 330 (32)	−0.014 3	δ_K (kHz)	1.236 08 (11)	1.19
H_J (Hz)	0.001 013 (84)	0.001 15	Φ_J (Hz)	[0.000 407]	0.000 407
H_{JK} (Hz)	−0.003 94 (27)	−0.004 61	Φ_{JK} (Hz)	[0.000 768]	0.000 768
H_{KJ} (Hz)	0.004 86 (30)	0.005 79	Φ_{KJ} (Hz)	−0.004 29 (29)	−0.005 08
H_K (Hz)	−0.001 98 (12)	−0.002 34	Φ_K (Hz)	0.003 65 (65)	0.005 57
h_1 (Hz)	[0.000 193]	0.000 193	ϕ_J (Hz)	[0.000 205]	0.000 205
h_2 (Hz)	[0.000 043 0]	0.000 043 0	ϕ_{JK} (Hz)	[0.000 662]	0.000 662
h_3 (Hz)	[0.000 018 3]	0.000 018 3	ϕ_K (Hz)	[0.002 56]	0.002 56
N_{lines} [c]	802		N_{lines} [c]	802	
σ_{fit} (MHz)	0.041		σ_{fit} (MHz)	0.041	

[a] Values in brackets are held constant at the computed values. [b] Evaluated using the cc-pCVTZ basis set. [c] Number of independent transitions.

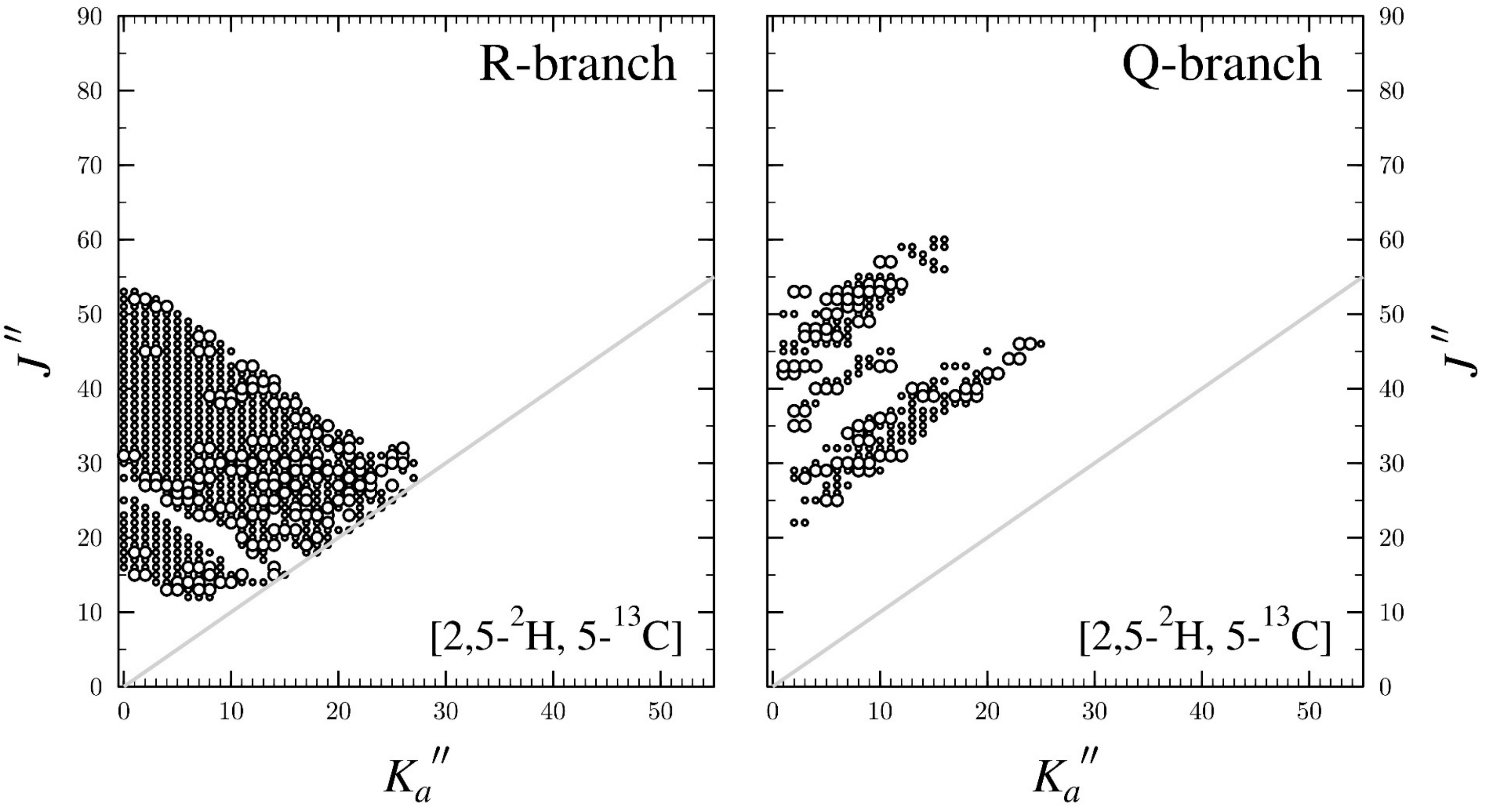

Figure S18. Data distribution plot for the least-squares fit of millimeter-wave spectroscopic data for [2,5-^{2}H, 5-^{13}C]-oxazole, ground vibrational state. The size of the outlined circle is proportional to the value of $|(f_{obs.} - f_{calc.})/\delta f|$, where δf is the frequency measurement uncertainty (50 kHz), and no quotient values are larger than three.

Table S19. Experimental and computed spectroscopic constants for [2,5-^{2}H, 3-^{15}N]-oxazole [a]

	S Reduction, III^r representation			A Reduction, I^r representation	
	Experimental	CCSD(T) [b]		Experimental	CCSD(T) [b]
A_0 (MHz)	9416.101 0 (15)	9366	A_0 (MHz)	9416.096 6 (24)	9366
B_0 (MHz)	8417.864 6 (11)	8383	B_0 (MHz)	8417.865 8 (17)	8383
C_0 (MHz)	4442.400 44 (23)	4421	C_0 (MHz)	4442.400 26 (13)	4421
D_J (kHz)	2.925 71 (45)	2.86	Δ_J (kHz)	1.484 27 (98)	1.46
D_{JK} (kHz)	−4.564 74 (90)	−4.46	Δ_{JK} (kHz)	−0.332 7 (34)	−0.331
D_K (kHz)	1.954 98 (45)	1.91	Δ_K (kHz)	2.103 (10)	2.05
d_1 (kHz)	−0.150 24 (68)	−0.142	δ_J (kHz)	0.584 05 (49)	0.574
d_2 (kHz)	[−0.014 1]	−0.014 1	δ_K (kHz)	1.181 0 (26)	1.15
H_J (Hz)	[0.001 15]	0.001 15	Φ_J (Hz)	[0.000 423]	0.000 423
H_{JK} (Hz)	−0.004 654 (31)	−0.004 60	Φ_{JK} (Hz)	[0.000 576]	0.000 576
H_{KJ} (Hz)	[0.005 79]	0.005 79	Φ_{KJ} (Hz)	−0.007 6 (13)	−0.004 50
H_K (Hz)	[−0.002 34]	−0.002 34	Φ_K (Hz)	[0.005 05]	0.005 05
h_1 (Hz)	[0.000 166]	0.000 166	ϕ_J (Hz)	[0.000 213]	0.000 213
h_2 (Hz)	[0.000 025 6]	0.000 025 6	ϕ_{JK} (Hz)	[0.000 570]	0.000 570
h_3 (Hz)	[0.000 008 00]	0.000 008 00	ϕ_K (Hz)	[0.002 56]	0.002 56
N_{lines} [c]	381		N_{lines} [c]	381	
σ_{fit} (MHz)	0.041		σ_{fit} (MHz)	0.041	

[a] Values in brackets are held constant at the computed values. [b] Evaluated using the cc-pCVTZ basis set. [c] Number of independent transitions.

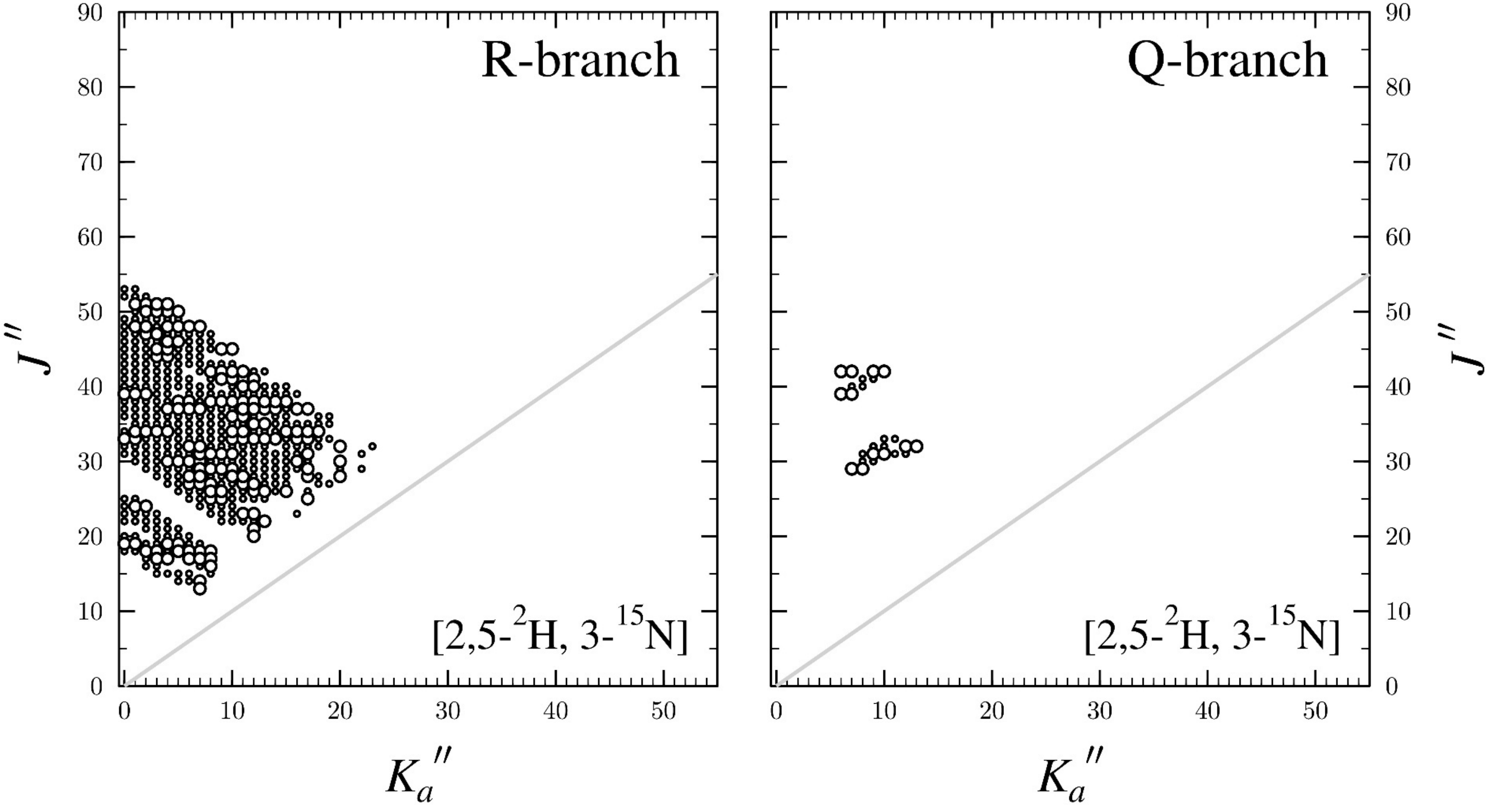

Figure S19. Data distribution plot for the least-squares fit of millimeter-wave spectroscopic data for [2,5-^{2}H, 3-^{15}N]-oxazole, ground vibrational state. The size of the outlined circle is proportional to the value of $|(f_{obs.} - f_{calc.})/\delta f|$, where δf is the frequency measurement uncertainty (50 kHz), and no quotient values are larger than three.

Table S20. Experimental and computed spectroscopic constants for [2,5-^{2}H, 1-^{18}O]-oxazole [a]

	S Reduction, III^r representation			A Reduction, I^r representation	
	Experimental	CCSD(T) [b]		Experimental	CCSD(T) [b]
A_0 (MHz)	9128.162 (25)	9079	A_0 (MHz)	9128.116 (12)	9079
B_0 (MHz)	8522.499 (24)	8488	B_0 (MHz)	8522.542 (11)	8488
C_0 (MHz)	4405.364 83 (22)	4384	C_0 (MHz)	4405.364 83 (25)	4384
D_J (kHz)	2.914 1 (58)	2.83	Δ_J (kHz)	1.491 614 (47)	1.48
D_{JK} (kHz)	−4.550 8 (75)	−4.42	Δ_{JK} (kHz)	−0.72 (10)	−0.474
D_K (kHz)	1.950 8 (18)	1.90	Δ_K (kHz)	2.65 (18)	2.05
d_1 (kHz)	−0.227 (31)	−0.100	δ_J (kHz)	[0.588 788]	0.589
d_2 (kHz)	[−0.015 2]	−0.015 2	δ_K (kHz)	0.916 (50)	1.04
H_J (Hz)	[0.001 10]	0.001 10	Φ_J (Hz)	[0.000 426]	0.000 426
H_{JK} (Hz)	[−0.004 48]	−0.004 48	Φ_{JK} (Hz)	[0.000 284]	0.000 284
H_{KJ} (Hz)	[0.005 68]	0.005 68	Φ_{KJ} (Hz)	[−0.003 86]	−0.003 86
H_K (Hz)	[−0.002 30]	−0.002 30	Φ_K (Hz)	[0.004 63]	0.004 63
h_1 (Hz)	[0.000 147]	0.000 147	ϕ_J (Hz)	[0.000 215]	0.000 215
h_2 (Hz)	[0.000 034 3]	0.000 034 3	ϕ_{JK} (Hz)	[0.000 423]	0.000 423
h_3 (Hz)	[0.000 007 10]	0.000 007 10	ϕ_K (Hz)	[0.002 29]	0.002 29
N_{lines} [c]	251		N_{lines} [c]	251	
σ_{fit} (MHz)	0.045		σ_{fit} (MHz)	0.045	

[a] Values in brackets are held constant at the computed values. [b] Evaluated using the cc-pCVTZ basis set. [c] Number of independent transitions.

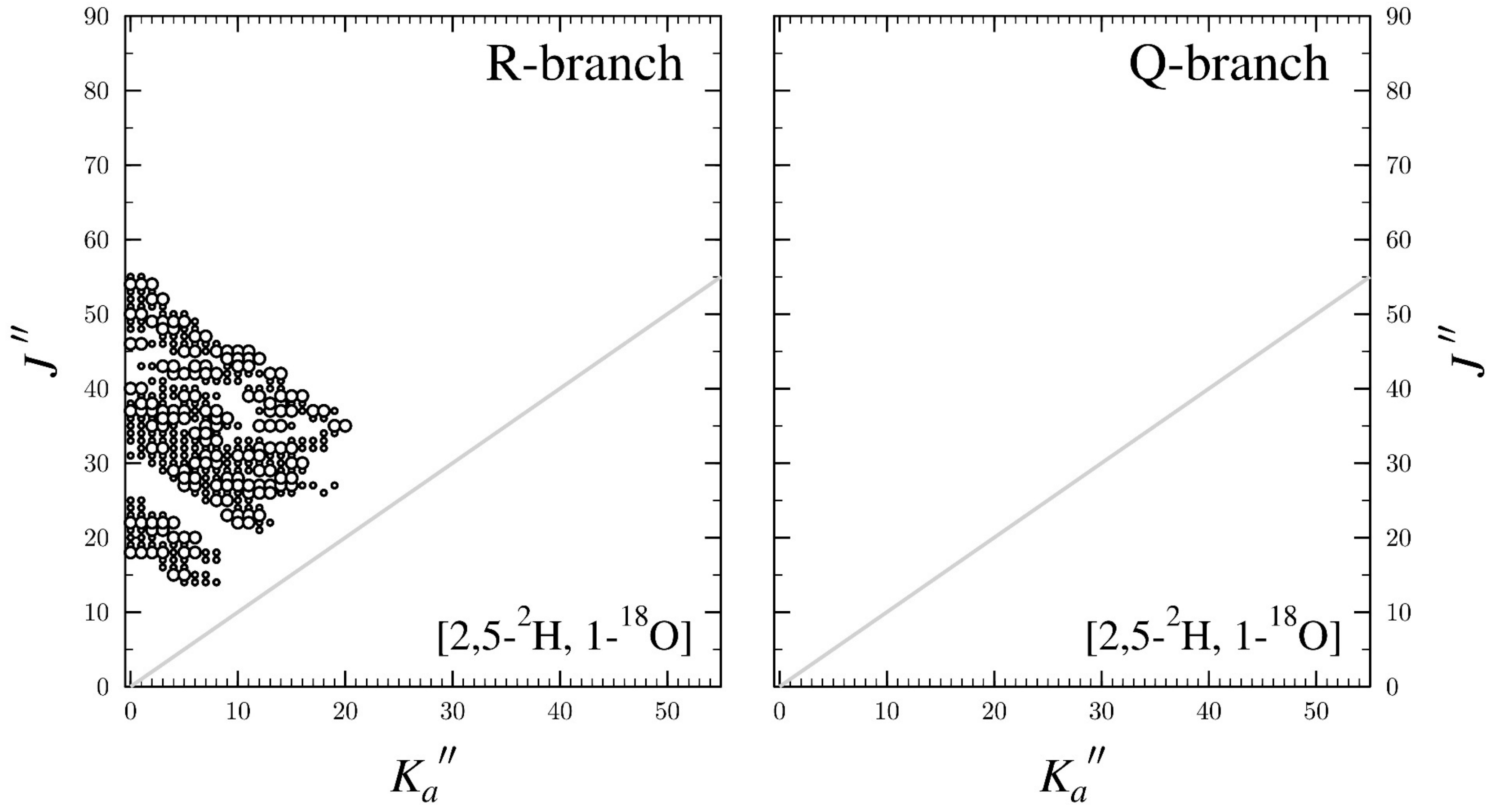

Figure S20. Data distribution plot for the least-squares fit of millimeter-wave spectroscopic data for [2,5-^{2}H, 1-^{18}O]-oxazole, ground vibrational state. The size of the outlined circle is proportional to the value of $|(f_{obs.} - f_{calc.})/\delta f|$, where δf is the frequency measurement uncertainty (50 kHz), and no quotient values are larger than three.

Table S21. Experimental and computed spectroscopic constants for [2,4-^{2}H]-oxazole [a]

	S Reduction, III^r representation			A Reduction, I^r representation	
	Experimental	CCSD(T) [b]		Experimental	CCSD(T) [b]
A_0 (MHz)	9510.525 65 (37)	9452	A_0 (MHz)	9510.523 32 (41)	9452
B_0 (MHz)	8531.252 78 (41)	8503	B_0 (MHz)	8531.255 77 (35)	8503
C_0 (MHz)	4494.965 20 (20)	4474	C_0 (MHz)	4494.964 98 (19)	4474
D_J (kHz)	3.035 45 (27)	2.97	Δ_J (kHz)	1.420 17 (22)	1.40
D_{JK} (kHz)	-4.731 08 (61)	-4.62	Δ_{JK} (kHz)	-0.092 5 (10)	-0.110
D_K (kHz)	2.017 92 (37)	1.97	Δ_K (kHz)	2.364 1 (11)	2.31
d_1 (kHz)	−0.293 69 (17)	-0.280	δ_J (kHz)	0.548 932 (93)	0.541
d_2 (kHz)	-0.034 576 (80)	-0.034 2	δ_K (kHz)	1.158 15 (24)	1.12
H_J (Hz)	0.000 966 (92)	0.001 17	Φ_J (Hz)	0.000 439 (26)	0.000 438
H_{JK} (Hz)	-0.004 40 (32)	-0.004 75	Φ_{JK} (Hz)	[0.000 481]	0.000 481
H_{KJ} (Hz)	0.005 75 (36)	0.006 02	Φ_{KJ} (Hz)	-0.005 12 (30)	-0.004 47
H_K (Hz)	-0.002 31 (14)	-0.002 45	Φ_K (Hz)	0.005 43 (74)	0.005 17
h_1 (Hz)	[0.000 177]	0.000 177	ϕ_J (Hz)	[0.000 221]	0.000 221
h_2 (Hz)	[0.000 040 0]	0.000 040 0	ϕ_{JK} (Hz)	[0.000 629]	0.000 629
h_3 (Hz)	[0.000 009 60]	0.000 009 60	ϕ_K (Hz)	[0.002 73]	0.002 73
N_{lines} [c]	596		N_{lines} [c]	596	
σ_{fit} (MHz)	0.040		σ_{fit} (MHz)	0.040	

[a] Values in brackets are held constant at the computed values. [b] Evaluated using the cc-pCVTZ basis set. [c] Number of independent transitions.

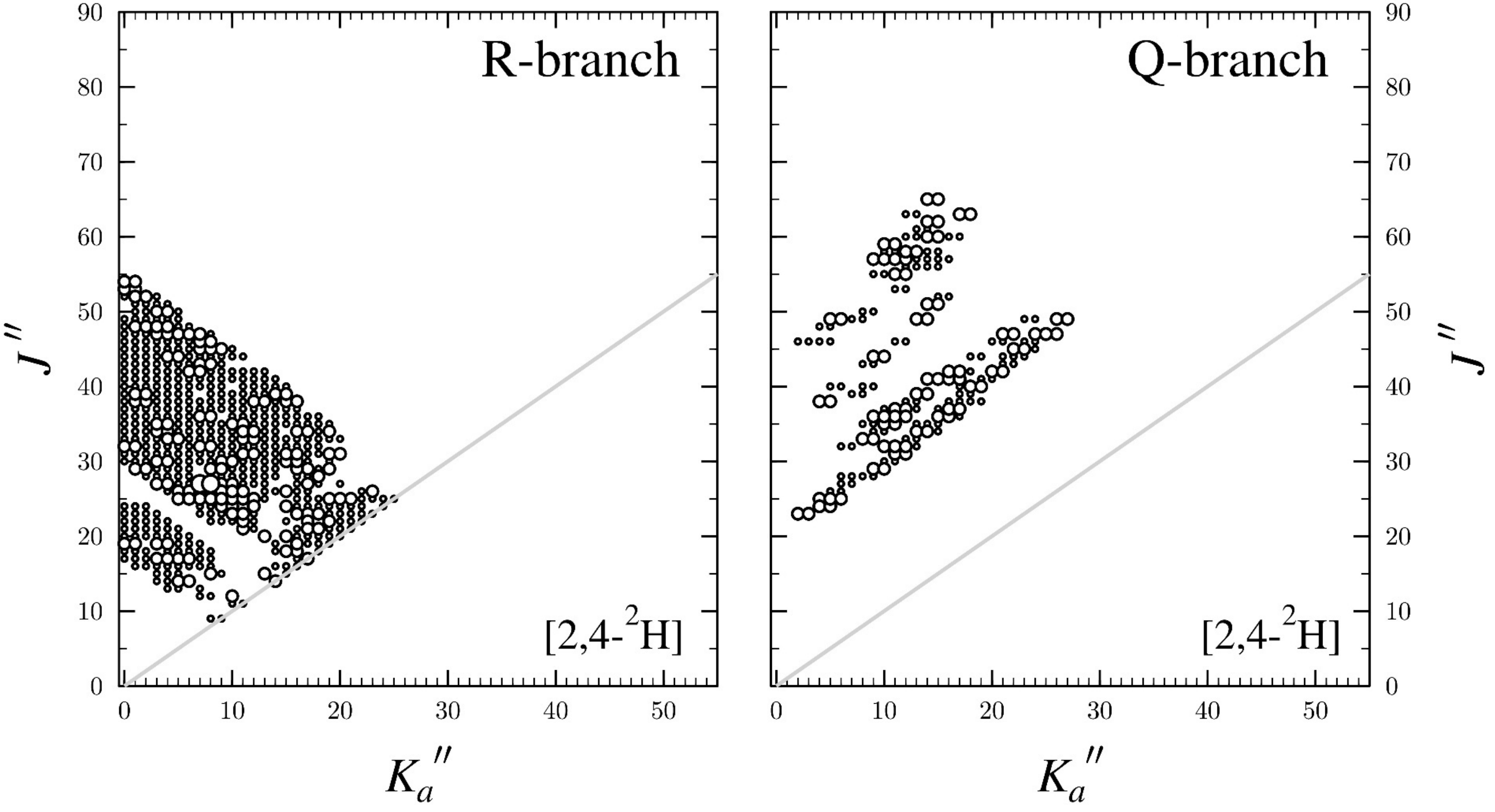

Figure S21. Data distribution plot for the least-squares fit of millimeter-wave spectroscopic data for [2,4-^{2}H]-oxazole, ground vibrational state. The size of the outlined circle is proportional to the value of $|(f_{obs.} - f_{calc.})/\delta f|$, where δf is the frequency measurement uncertainty (50 kHz), and no quotient values are larger than three.

Table S22. Experimental and computed spectroscopic constants for [4,5-^{2}H]-oxazole [a]

	S Reduction, III^r representation Experimental	CCSD(T) [b]		A Reduction, I^r representation Experimental	CCSD(T) [b]
A_0 (MHz)	9007.906 14 (55)	8975	A_0 (MHz)	9007.903 90 (56)	8975
B_0 (MHz)	8959.735 62 (58)	8908	B_0 (MHz)	8959.737 74 (58)	8908
C_0 (MHz)	4489.677 79 (16)	4468	C_0 (MHz)	4489.678 003 (100)	4468
D_J (kHz)	3.014 07 (19)	2.95	Δ_J (kHz)	1.744 29 (46)	1.68
D_{JK} (kHz)	−4.745 81 (42)	−4.64	Δ_{JK} (kHz)	−1.081 5 (16)	−0.878
D_K (kHz)	2.054 45 (28)	2.00	Δ_K (kHz)	2.296 3 (18)	2.09
d_1 (kHz)	0.051 57 (51)	0.038 5	δ_J (kHz)	0.710 76 (23)	0.682
d_2 (kHz)	−0.024 21 (18)	−0.008 45	δ_K (kHz)	0.965 99 (54)	0.992
H_J (Hz)	0.001 231 (63)	0.001 20	Φ_J (Hz)	[0.000 536]	0.000 536
H_{JK} (Hz)	−0.005 08 (23)	−0.004 95	Φ_{JK} (Hz)	0.001 78 (19)	0.001 41
H_{KJ} (Hz)	0.006 54 (28)	0.006 28	Φ_{KJ} (Hz)	−0.008 76 (37)	−0.007 73
H_K (Hz)	−0.002 71 (12)	−0.002 53	Φ_K (Hz)	0.007 66 (39)	0.006 89
h_1 (Hz)	[−0.000 056 9]	−0.000 056 9	ϕ_J (Hz)	[0.000 270]	0.000 270
h_2 (Hz)	[−0.000 054 6]	−0.000 054 6	ϕ_{JK} (Hz)	[0.001 03]	0.001 03
h_3 (Hz)	[0.000 066 4]	0.000 066 4	ϕ_K (Hz)	[0.001 40]	0.001 40
N_{lines} [c]	667		N_{lines} [c]	667	
σ_{fit} (MHz)	0.039		σ_{fit} (MHz)	0.039	

[a] Values in brackets are held constant at the computed values. [b] Evaluated using the cc-pCVTZ basis set. [c] Number of independent transitions.

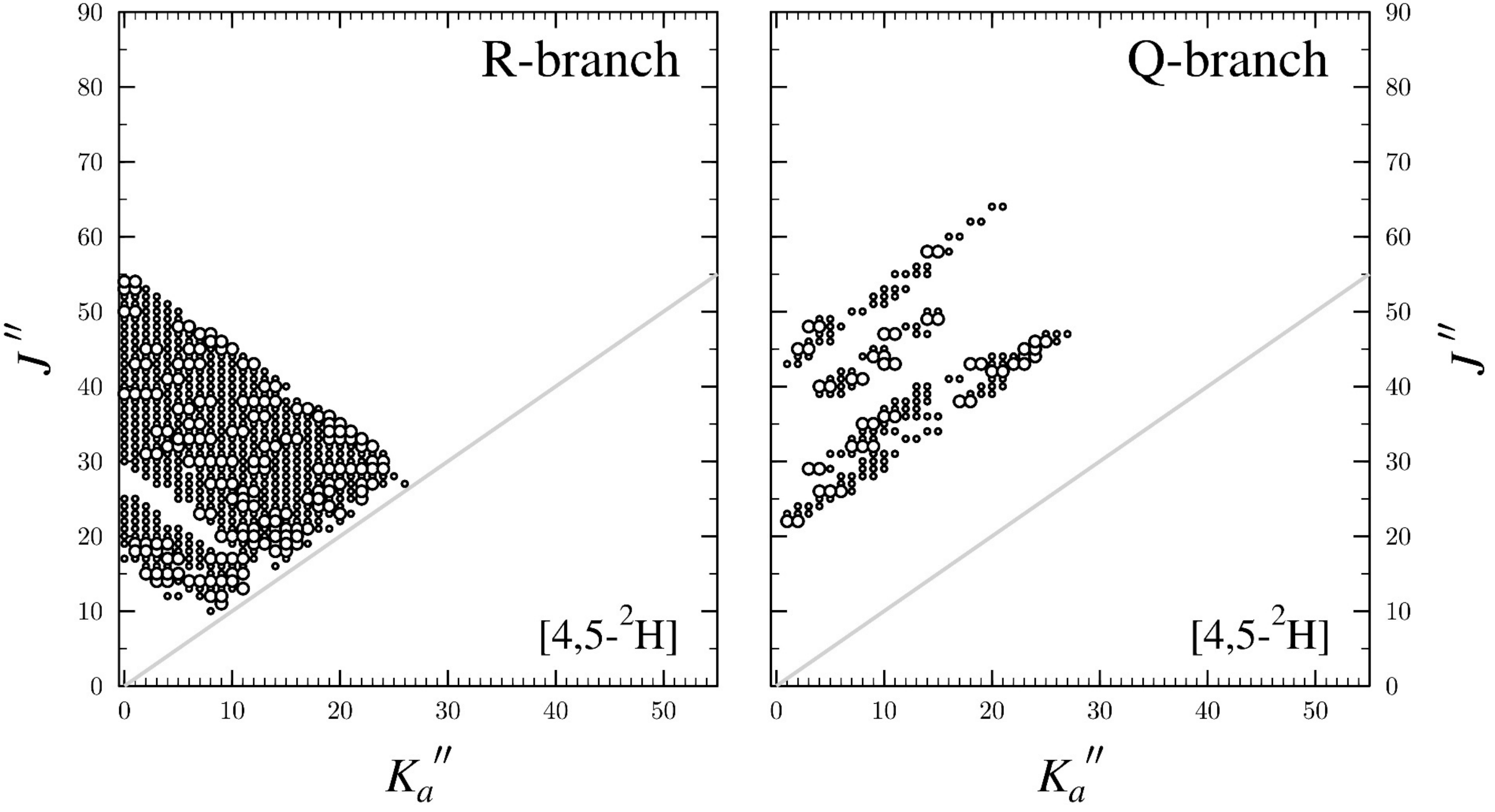

Figure S22. Data distribution plot for the least-squares fit of millimeter-wave spectroscopic data for [4,5-^{2}H]-oxazole, ground vibrational state. The size of the outlined circle is proportional to the value of $|(f_{obs.} - f_{calc.})/\delta f|$, where δf is the frequency measurement uncertainty (50 kHz), and no quotient values are larger than three.

Table S23. Experimental and computed spectroscopic constants for [2,4,5-^{2}H]-oxazole [a]

	S Reduction, III^r representation			A Reduction, I^r representation	
	Experimental	CCSD(T) [b]		Experimental	CCSD(T) [b]
A_0 (MHz)	8978.045 39 (11)	8919	A_0 (MHz)	8978.043 506 (99)	8919
B_0 (MHz)	8282.223 547 (90)	8260	B_0 (MHz)	8282.225 820 (77)	8260
C_0 (MHz)	4306.085 048 (92)	4286	C_0 (MHz)	4306.084 653 (91)	4286
D_J (kHz)	2.708 457 (62)	2.65	Δ_J (kHz)	1.325 506 (61)	1.30
D_{JK} (kHz)	−4.246 976 (80)	−4.15	Δ_{JK} (kHz)	−0.038 92 (13)	−0.042 4
D_K (kHz)	1.826 776 (56)	1.78	Δ_K (kHz)	1.728 01 (13)	1.68
d_1 (kHz)	−0.163 055 (61)	−0.153	δ_J (kHz)	0.518 633 (13)	0.510
d_2 (kHz)	0.009 893 (10)	0.010 8	δ_K (kHz)	1.101 158 (36)	1.07
H_J (Hz)	0.001 101 (15)	0.001 06	Φ_J (Hz)	0.000 344 (13)	0.000 344
H_{JK} (Hz)	−0.004 309 (27)	−0.004 20	Φ_{JK} (Hz)	0.000 660 (47)	0.000 611
H_{KJ} (Hz)	0.005 376 (33)	0.005 25	Φ_{KJ} (Hz)	−0.003 937 (91)	−0.003 67
H_K (Hz)	−0.002 169 (19)	−0.002 11	Φ_K (Hz)	0.004 524 (67)	0.004 15
h_1 (Hz)	0.000 250 (21)	0.000 188	ϕ_J (Hz)	[0.000 173]	0.000 173
h_2 (Hz)	[0.000 002 40]	0.000 002 40	ϕ_{JK} (Hz)	[0.000 555]	0.000 555
h_3 (Hz)	[−0.000 001 60]	−0.000 001 60	ϕ_K (Hz)	[0.002 15]	0.002 15
N_{lines} [c]	2037		N_{lines} [c]	2037	
σ_{fit} (MHz)	0.036		σ_{fit} (MHz)	0.036	

[a] Values in brackets are held constant at the computed values. [b] Evaluated using the cc-pCVTZ basis set. [c] Number of independent transitions.

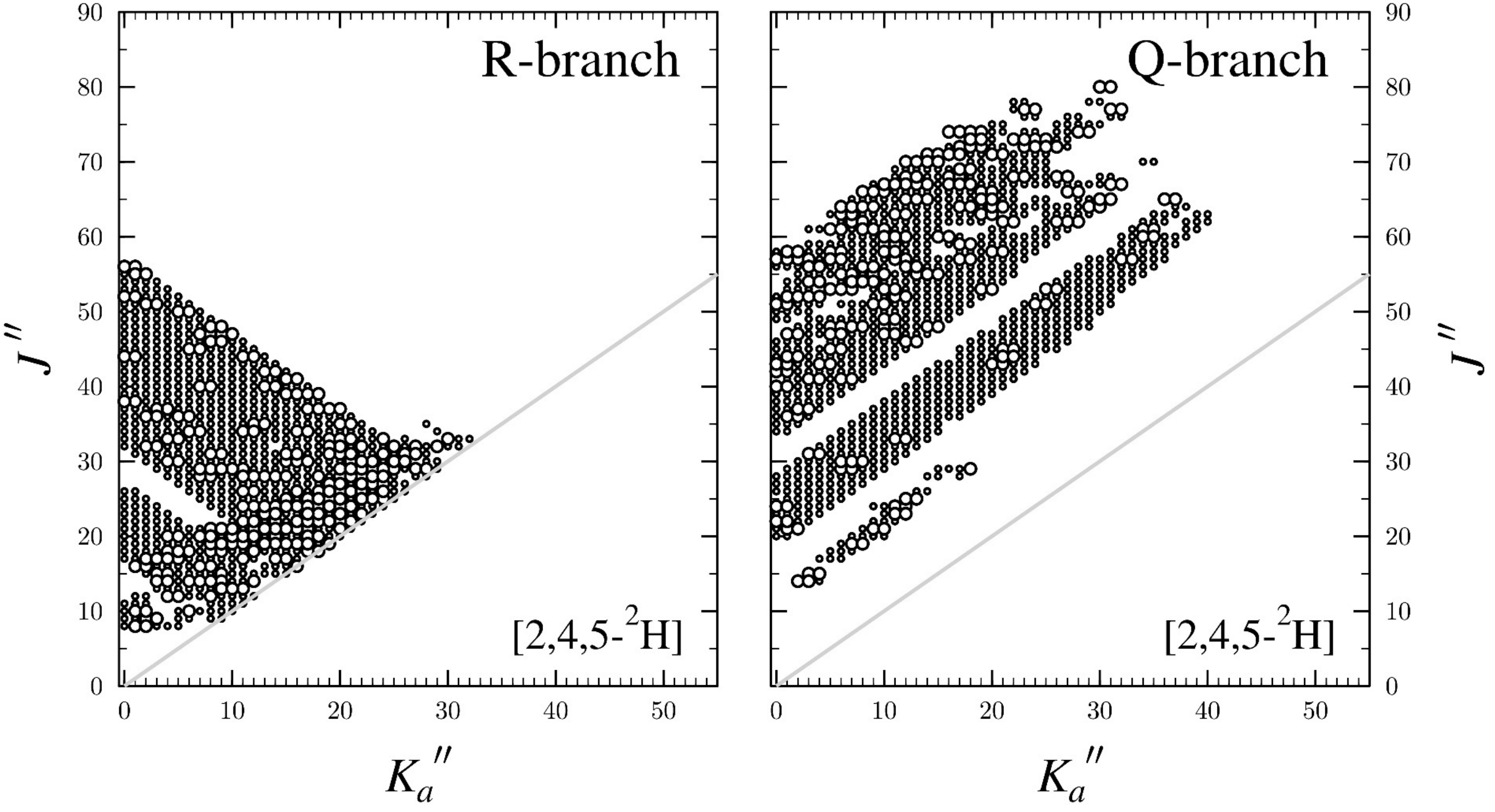

Figure S23. Data distribution plot for the least-squares fit of millimeter-wave spectroscopic data for [2,4,5-^{2}H]-oxazole, ground vibrational state. The size of the outlined circle is proportional to the value of $|(f_{obs.} - f_{calc.})/\delta f|$, where δf is the frequency measurement uncertainty (50 kHz), and no quotient values are larger than three.

Table S24. Experimental and computed spectroscopic constants for [5-^{2}H, 2-^{13}C]-oxazole [a]

	S Reduction, III^r representation			A Reduction, I^r representation	
	Experimental	CCSD(T) [b]		Experimental	CCSD(T) [b]
A_0 (MHz)	9774.000 8 (83)	9729	A_0 (MHz)	9774.019 1 (84)	9729
B_0 (MHz)	8860.486 6 (93)	8817	B_0 (MHz)	8860.470 6 (92)	8817
C_0 (MHz)	4645.061 99 (56)	4623	C_0 (MHz)	4645.061 87 (57)	4623
D_J (kHz)	3.272 5 (13)	3.21	Δ_J (kHz)	1.684 28 (14)	1.68
D_{JK} (kHz)	−5.111 2 (27)	−5.01	Δ_{JK} (kHz)	−0.610 3 (11)	−0.623
D_K (kHz)	2.194 0 (14)	2.15	Δ_K (kHz)	2.668 6 (47)	2.51
d_1 (kHz)	[−0.139]	−0.139	δ_J (kHz)	[0.664]	0.664
d_2 (kHz)	[−0.036 0]	−0.036 0	δ_K (kHz)	[1.21]	1.21
H_J (Hz)	[0.001 31]	0.001 31	Φ_J (Hz)	[0.000 583]	0.000 583
H_{JK} (Hz)	[−0.005 43]	−0.005 43	Φ_{JK} (Hz)	[−0.000 169]	−0.000 169
H_{KJ} (Hz)	[0.006 92]	0.006 92	Φ_{KJ} (Hz)	[−0.003 78]	−0.003 78
H_K (Hz)	[−0.002 81]	−0.002 81	Φ_K (Hz)	[0.005 02]	0.005 02
h_1 (Hz)	[0.000 132]	0.000 132	ϕ_J (Hz)	[0.000 294]	0.000 294
h_2 (Hz)	[0.000 047 7]	0.000 047 7	ϕ_{JK} (Hz)	[0.000 347]	0.000 347
h_3 (Hz)	[−0.000 012 0]	−0.000 012 0	ϕ_K (Hz)	[0.003 35]	0.003 35
N_{lines} [c]	122		N_{lines} [c]	122	
σ_{fit} (MHz)	0.042		σ_{fit} (MHz)	0.042	

[a] Values in brackets are held constant at the computed values. [b] Evaluated using the cc-pCVTZ basis set. [c] Number of independent transitions.

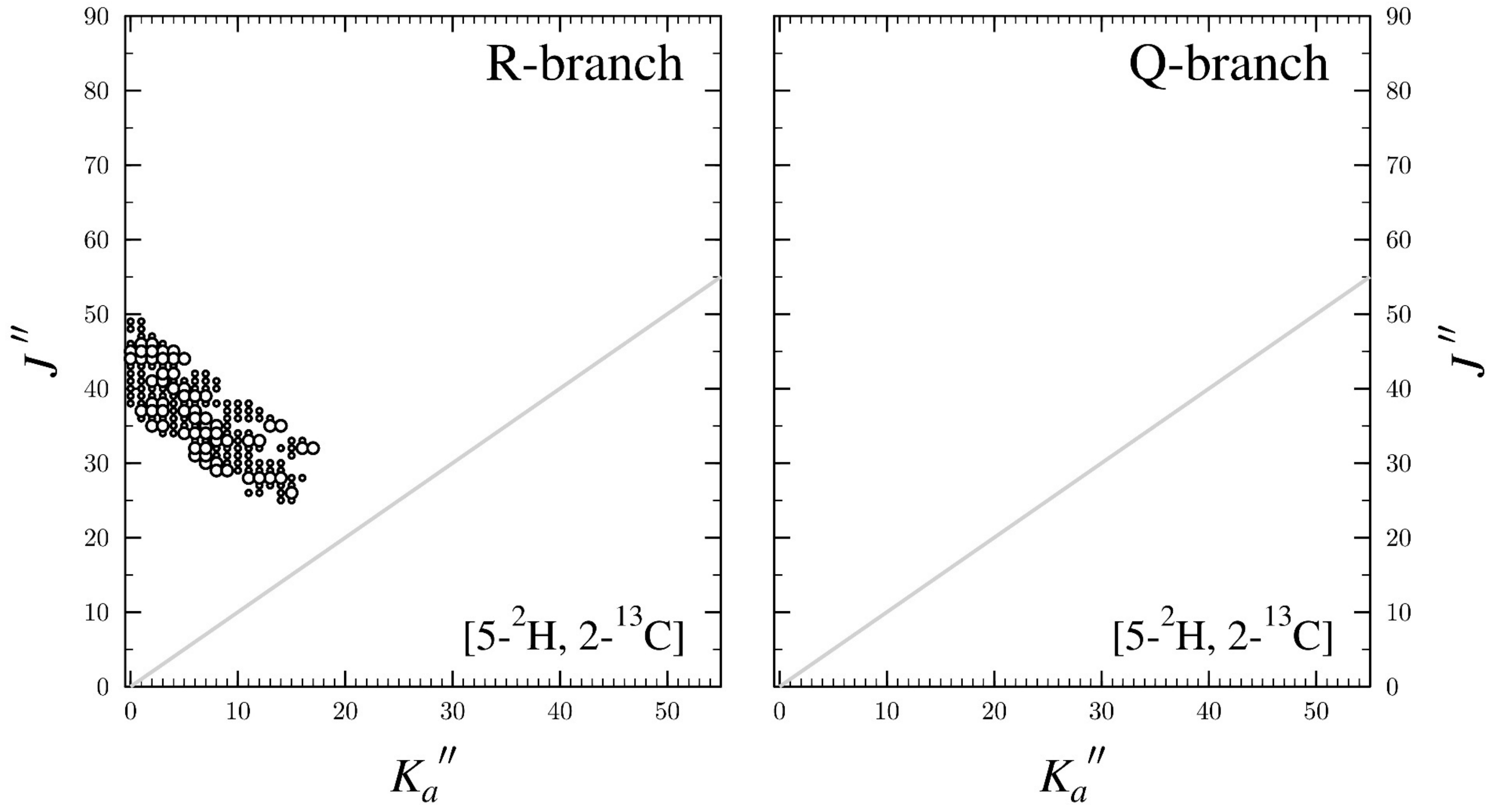

Figure S24. Data distribution plot for the least-squares fit of millimeter-wave spectroscopic data for [5-^{2}H, 2-^{13}C]-oxazole, ground vibrational state. The size of the outlined circle is proportional to the value of $|(f_{obs.} - f_{calc.})/\delta f|$, where δf is the frequency measurement uncertainty (50 kHz), and no quotient values are larger than three.

Table S25. Experimental and computed spectroscopic constants for [5-^{2}H, 4-^{13}C]-oxazole [a]

	S Reduction, III^r representation			A Reduction, I^r representation	
	Experimental	CCSD(T) [b]		Experimental	CCSD(T) [b]
A_0 (MHz)	9631.611 (13)	9592	A_0 (MHz)	9631.634 (14)	9592
B_0 (MHz)	8960.106 (13)	8912	B_0 (MHz)	8960.085 (13)	8912
C_0 (MHz)	4639.491 77 (52)	4617	C_0 (MHz)	4639.491 87 (53)	4617
D_J (kHz)	3.276 9 (13)	3.21	Δ_J (kHz)	1.724 85 (13)	1.72
D_{JK} (kHz)	−5.131 1 (27)	−5.02	Δ_{JK} (kHz)	[−0.820]	−0.820
D_K (kHz)	2.208 3 (14)	2.16	Δ_K (kHz)	2.813 1 (43)	2.62
d_1 (kHz)	[−0.107]	−0.107	δ_J (kHz)	[0.685]	0.685
d_2 (kHz)	[−0.046 3]	−0.046 3	δ_K (kHz)	1.105 86 (54)	1.12
H_J (Hz)	[0.001 31]	0.001 31	Φ_J (Hz)	[0.000 652]	0.000 652
H_{JK} (Hz)	[−0.005 45]	−0.005 45	Φ_{JK} (Hz)	[−0.000 682]	−0.000 682
H_{KJ} (Hz)	[0.006 96]	0.006 96	Φ_{KJ} (Hz)	[−0.003 08]	−0.003 08
H_K (Hz)	[−0.002 83]	−0.002 83	Φ_K (Hz)	[0.004 71]	0.004 71
h_1 (Hz)	[0.000 086 1]	0.000 086 1	ϕ_J (Hz)	[0.000 328]	0.000 328
h_2 (Hz)	[0.000 072 1]	0.000 072 1	ϕ_{JK} (Hz)	[0.000 171]	0.000 171
h_3 (Hz)	[−0.000 013 5]	−0.000 013 5	ϕ_K (Hz)	[0.003 27]	0.003 27
N_{lines} [c]	139		N_{lines} [c]	139	
σ_{fit} (MHz)	0.042		σ_{fit} (MHz)	0.043	

[a] Values in brackets are held constant at the computed values. [b] Evaluated using the cc-pCVTZ basis set. [c] Number of independent transitions.

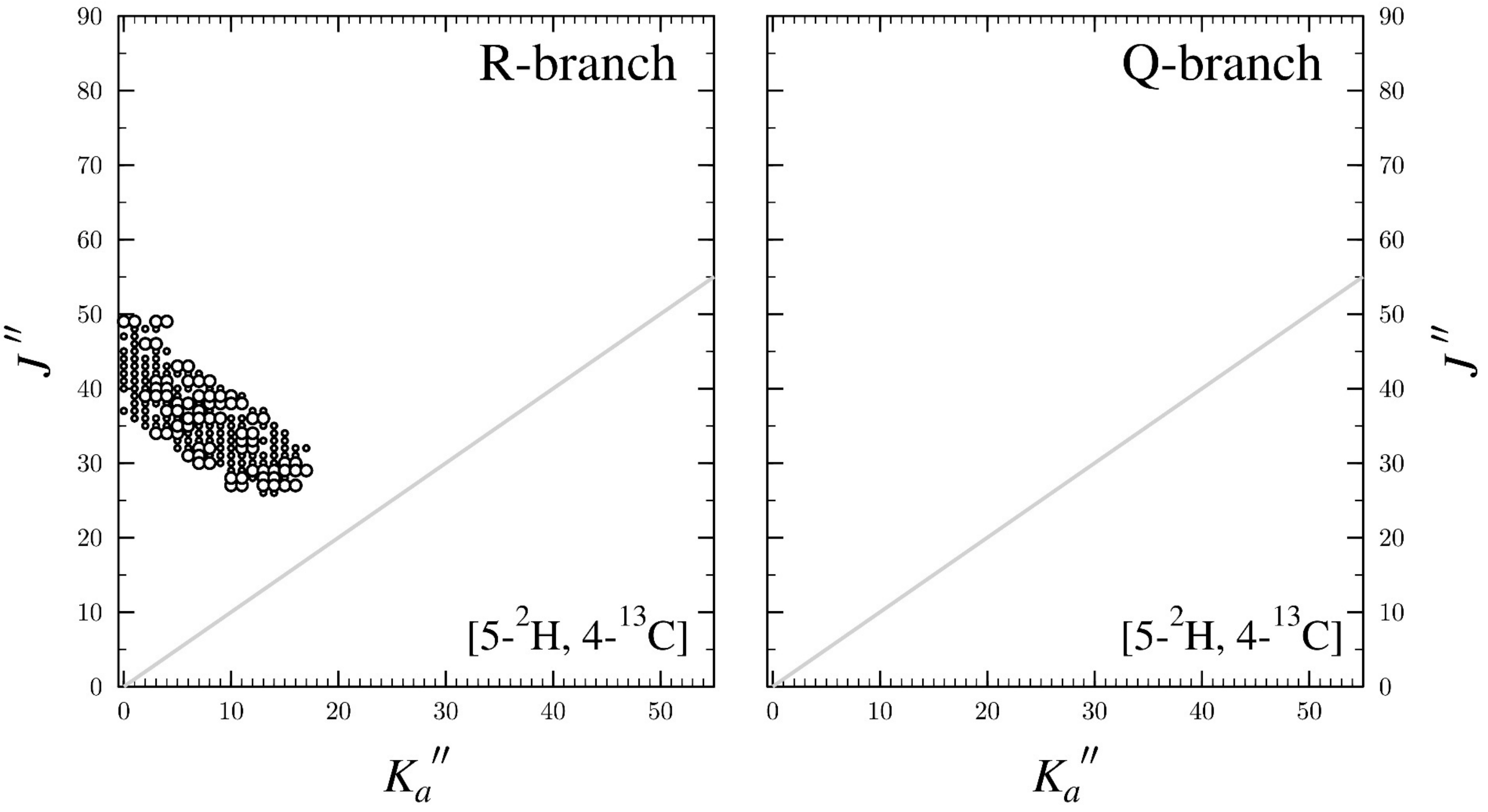

Figure S25. Data distribution plot for the least-squares fit of millimeter-wave spectroscopic data for [5-^{2}H, 4-^{13}C]-oxazole, ground vibrational state. The size of the outlined circle is proportional to the value of $|(f_{obs.} - f_{calc.})/\delta f|$, where δf is the frequency measurement uncertainty (50 kHz), and no quotient values are larger than three.

Table S26. Experimental and computed spectroscopic constants for [5-^{2}H, 5-^{13}C]-oxazole [a]

	S Reduction, III^r representation			A Reduction, I^r representation	
	Experimental	CCSD(T) [b]		Experimental	CCSD(T) [b]
A_0 (MHz)	9868.535 (13)	9825	A_0 (MHz)	9868.551 (13)	9825
B_0 (MHz)	8780.665 (13)	8736	B_0 (MHz)	8780.652 (13)	8736
C_0 (MHz)	4644.075 81 (62)	4622	C_0 (MHz)	4644.075 64 (60)	4622
D_J (kHz)	3.277 7 (16)	3.22	Δ_J (kHz)	1.672 26 (15)	1.66
D_{JK} (kHz)	−5.102 3 (33)	−5.01	Δ_{JK} (kHz)	[−0.642]	−0.642
D_K (kHz)	2.181 5 (17)	2.14	Δ_K (kHz)	2.819 6 (61)	2.64
d_1 (kHz)	−[0.170]	−0.170	δ_J (kHz)	[0.658]	0.658
d_2 (kHz)	[−0.049 8]	−0.049 8	δ_K (kHz)	1.206 14 (66)	1.22
H_J (Hz)	[0.001 30]	0.001 30	Φ_J (Hz)	[0.000 634]	0.000 634
H_{JK} (Hz)	[−0.005 40]	−0.005 40	Φ_{JK} (Hz)	[−0.000 510]	−0.000 510
H_{KJ} (Hz)	[0.006 90]	0.006 90	Φ_{KJ} (Hz)	[−0.003 52]	−0.003 52
H_K (Hz)	[−0.002 81]	−0.002 81	Φ_K (Hz)	[0.005 05]	0.005 05
h_1 (Hz)	−[−0.000 107]	0.000 107	ϕ_J (Hz)	[0.000 319]	0.000 319
h_2 (Hz)	[0.000 080 0]	0.000 080 0	ϕ_{JK} (Hz)	[0.000 269]	0.000 269
h_3 (Hz)	−[0.000 012 7]	−0.000 012 7	ϕ_K (Hz)	[0.003 56]	0.003 56
N_{lines} [c]	118		N_{lines} [c]	118	
σ_{fit} (MHz)	0.045		σ_{fit} (MHz)	0.045	

[a] Values in brackets are held constant at the computed values. [b] Evaluated using the cc-pCVTZ basis set. [c] Number of independent transitions.

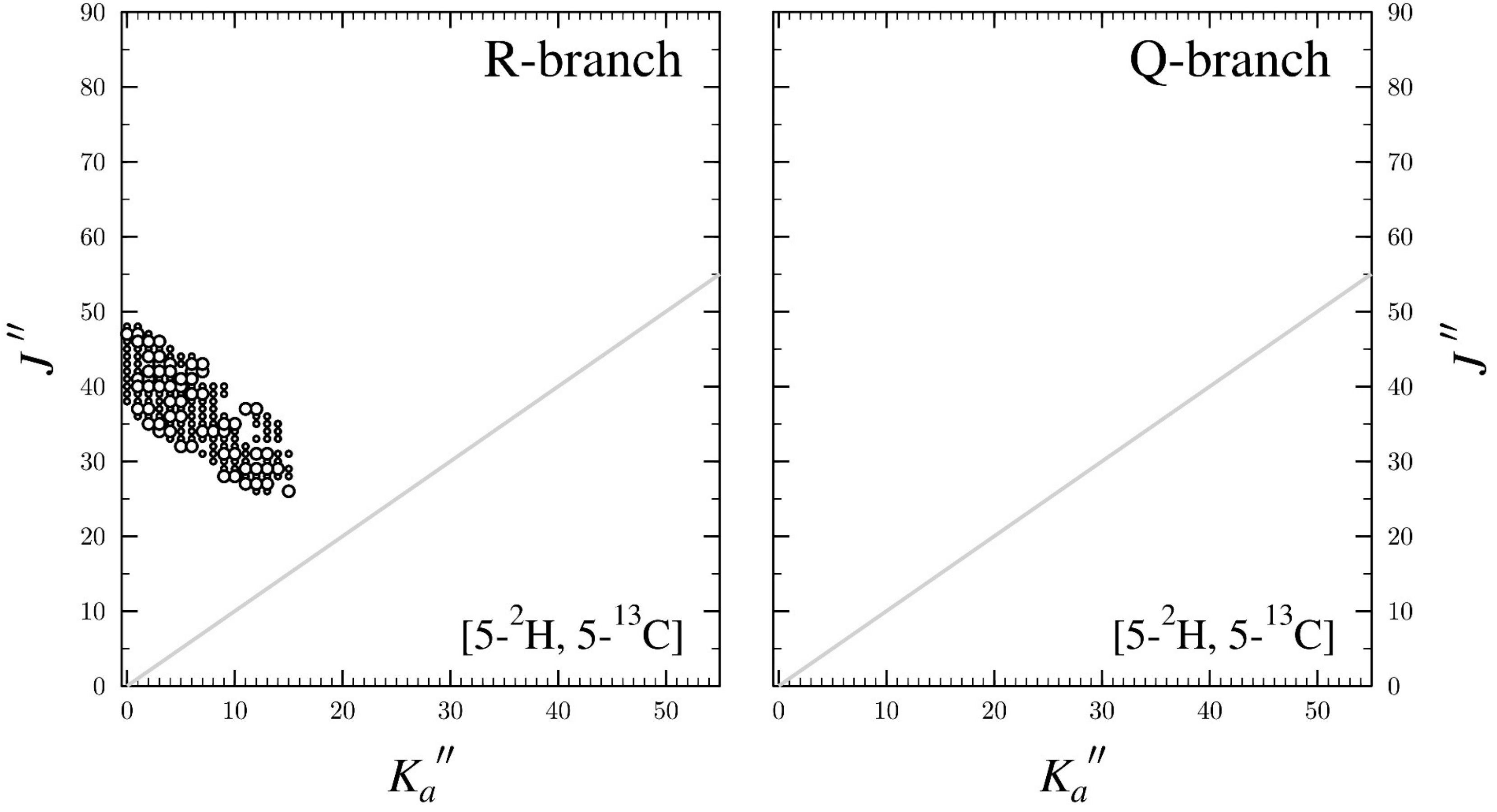

Figure S26. Data distribution plot for the least-squares fit of millimeter-wave spectroscopic data for [5-^{2}H, 5-^{13}C]-oxazole, ground vibrational state. The size of the outlined circle is proportional to the value of $|(f_{obs.} - f_{calc.})/\delta f|$, where δf is the frequency measurement uncertainty (50 kHz), and no quotient values are larger than three.

Table S27. Experimental and computed spectroscopic constants for [5-^{2}H, 3-^{15}N]-oxazole [a]

	S Reduction, III^r representation			A Reduction, I^r representation	
	Experimental	CCSD(T) [b]		Experimental	CCSD(T) [b]
A_0 (MHz)	9859.43 (98)	9821	A_0 (MHz)	9859.38 (98)	9821
B_0 (MHz)	8746.45 (79)	8697	B_0 (MHz)	8746.49 (79)	8697
C_0 (MHz)	4632.446 6 (28)	4610	C_0 (MHz)	4632.446 4 (28)	4610
D_J (kHz)	3.284 (18)	3.20	Δ_J (kHz)	1.663 48 (73)	1.66
D_{JK} (kHz)	−5.118 (35)	−4.98	Δ_{JK} (kHz)	−0.604 (13)	−0.640
D_K (kHz)	2.188 (16)	2.12	Δ_K (kHz)	2.825 (58)	2.62
d_1 (kHz)	[−0.167]	−0.167	δ_J (kHz)	[0.655]	0.655
d_2 (kHz)	[−0.049 4]	−0.049 4	δ_K (kHz)	[1.22]	1.22
H_J (Hz)	[0.001 29]	0.001 29	Φ_J (Hz)	[0.000 630]	0.000 630
H_{JK} (Hz)	[−0.005 35]	−0.005 35	Φ_{JK} (Hz)	[−0.000 336]	−0.000 336
H_{KJ} (Hz)	[0.006 83]	0.006 83	Φ_{KJ} (Hz)	[−0.003 95]	−0.003 95
H_K (Hz)	[−0.002 78]	−0.002 78	Φ_K (Hz)	[0.005 23]	0.005 23
h_1 (Hz)	[0.000 083 8]	0.000 083 8	ϕ_J (Hz)	[0.000 317]	0.000 317
h_2 (Hz)	[0.000 065 1]	0.000 065 1	ϕ_{JK} (Hz)	[0.000 351]	0.000 351
h_3 (Hz)	[−0.000 007 40]	−0.000 007 40	ϕ_K (Hz)	[0.003 49]	0.003 49
N_{lines} [c]	22		N_{lines} [c]	22	
σ_{fit} (MHz)	0.038		σ_{fit} (MHz)	0.038	

[a] Values in brackets are held constant at the computed values. [b] Evaluated using the cc-pCVTZ basis set. [c] Number of independent transitions.

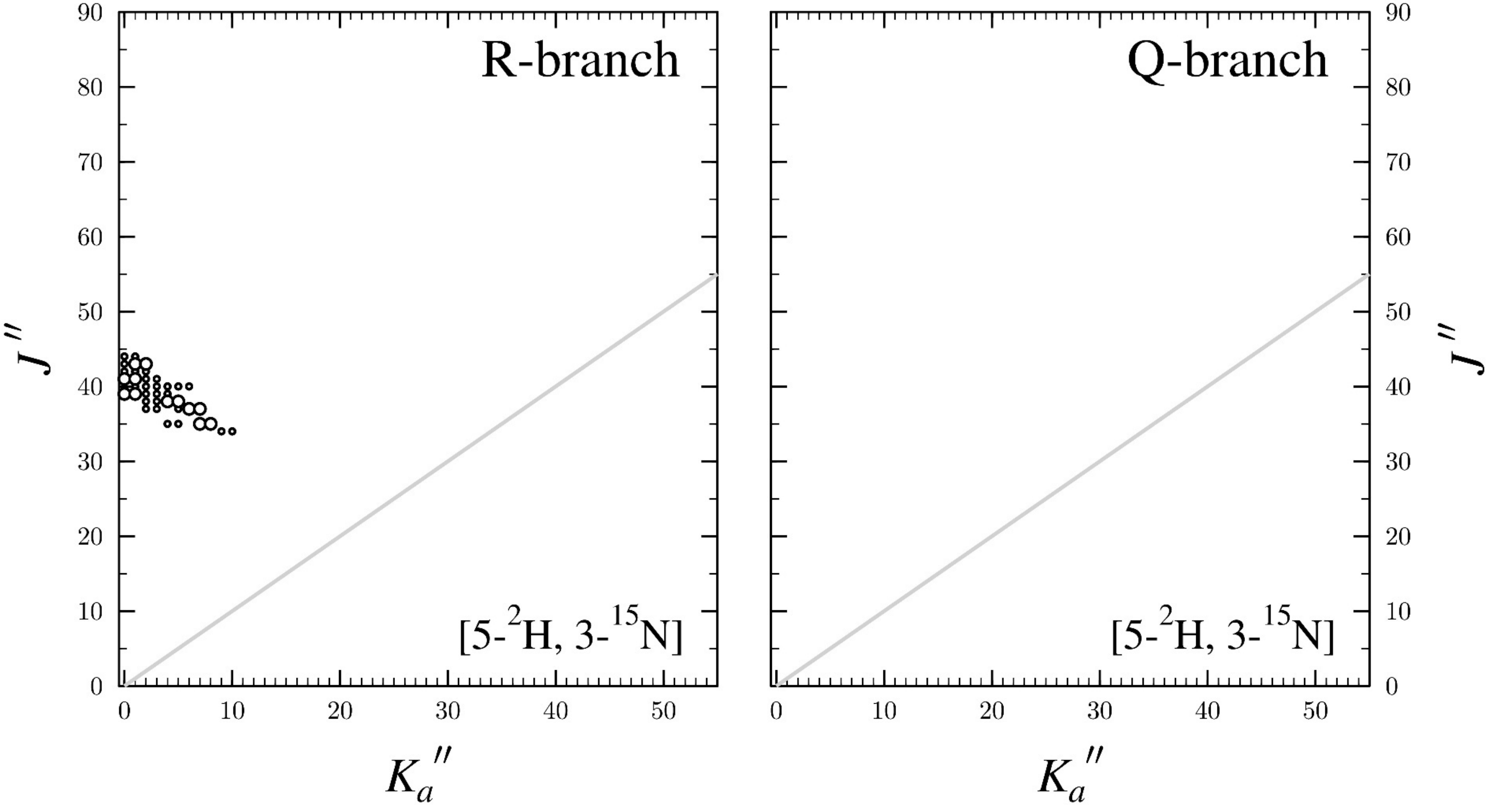

Figure S27. Data distribution plot for the least-squares fit of millimeter-wave spectroscopic data for [5-^{2}H, 3-^{15}N]-oxazole, ground vibrational state. The size of the outlined circle is proportional to the value of $|(f_{obs.} - f_{calc.})/\delta f|$, where δf is the frequency measurement uncertainty (50 kHz), and no quotient values are larger than three.

Table S28. Experimental and computed spectroscopic constants for [2,4,5-^{2}H, 2-^{13}C]-oxazole [a]

	S Reduction, III^{r} representation			A Reduction, I^{r} representation	
	Experimental	CCSD(T) [b]		Experimental	CCSD(T) [b]
A_0 (MHz)	8978.291 7 (22)	8919	A_0 (MHz)	8978.292 1 (25)	8919
B_0 (MHz)	8123.228 8 (38)	8101	B_0 (MHz)	8123.229 9 (40)	8101
C_0 (MHz)	4262.756 63 (31)	4243	C_0 (MHz)	4262.756 18 (31)	4243
D_J (kHz)	2.651 92 (97)	2.60	Δ_J (kHz)	1.281 20 (80)	1.26
D_{JK} (kHz)	−4.148 0 (21)	−4.07	Δ_{JK} (kHz)	[−0.017 1]	−0.017 1
D_K (kHz)	1.778 8 (11)	1.74	Δ_K (kHz)	1.748 2 (30)	1.69
d_1 (kHz)	−0.183 68 (98)	−0.172	δ_J (kHz)	0.499 25 (42)	0.492
d_2 (kHz)	[0.005 32]	0.005 32	δ_K (kHz)	1.095 3 (15)	1.07
H_J (Hz)	[0.001 03]	0.001 03	Φ_J (Hz)	[0.000 342]	0.000 342
H_{JK} (Hz)	[−0.004 06]	−0.004 06	Φ_{JK} (Hz)	[0.000 553]	0.000 553
H_{KJ} (Hz)	[0.005 07]	0.005 07	Φ_{KJ} (Hz)	[−0.003 63]	−0.003 63
H_K (Hz)	[−0.002 04]	−0.002 04	Φ_K (Hz)	[0.004 17]	0.004 17
h_1 (Hz)	[0.000 186]	0.000 186	ϕ_J (Hz)	[0.000 172]	0.000 172
h_2 (Hz)	[0.000 016 8]	0.000 016 8	ϕ_{JK} (Hz)	[0.000 539]	0.000 539
h_3 (Hz)	[0.000 000 200]	0.000 000 200	ϕ_K (Hz)	[0.002 17]	0.002 17
N_{lines} [c]	153		N_{lines} [c]	153	
σ_{fit} (MHz)	0.045		σ_{fit} (MHz)	0.045	

[a] Values in brackets are held constant at the computed values. [b] Evaluated using the cc-pCVTZ basis set. [c] Number of independent transitions.

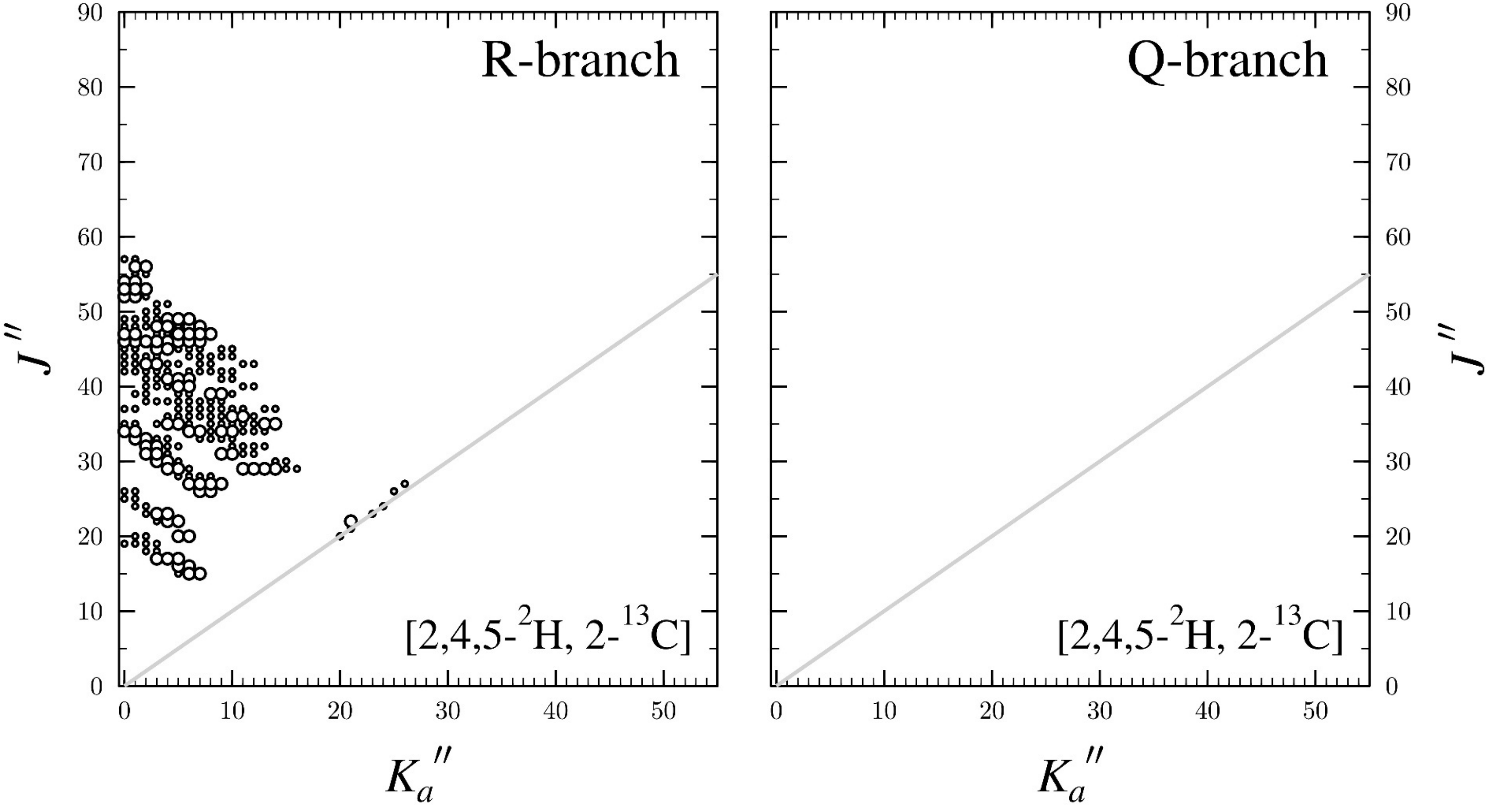

Figure S28. Data distribution plot for the least-squares fit of millimeter-wave spectroscopic data for [2,4,5-^{2}H, 2-^{13}C]-oxazole, ground vibrational state. The size of the outlined circle is proportional to the value of $|(f_{obs.} - f_{calc.})/\delta f|$, where δf is the frequency measurement uncertainty (50 kHz), and no quotient values are larger than three.

Table S29. Experimental and computed spectroscopic constants for [2,4,5-^{2}H, 4-^{13}C]-oxazole [a]

	S Reduction, III^r representation			A Reduction, I^r representation	
	Experimental	CCSD(T) [b]		Experimental	CCSD(T) [b]
A_0 (MHz)	8911.825 6 (19)	8853	A_0 (MHz)	8911.823 6 (19)	8853
B_0 (MHz)	8161.267 7 (36)	8139	B_0 (MHz)	8161.270 2 (36)	8139
C_0 (MHz)	4258.081 09 (30)	4238	C_0 (MHz)	4258.080 71 (30)	4238
D_J (kHz)	2.656 19 (87)	2.60	Δ_J (kHz)	1.287 28 (66)	1.26
D_{JK} (kHz)	−4.162 9 (18)	−4.07	Δ_{JK} (kHz)	[−0.024 8]	−0.024 8
D_K (kHz)	1.788 81 (97)	1.74	Δ_K (kHz)	1.735 9 (20)	1.68
d_1 (kHz)	−0.177 36 (81)	−0.167	δ_J (kHz)	0.502 59 (34)	0.494
d_2 (kHz)	[0.006 38]	0.006 38	δ_K (kHz)	1.081 2 (11)	1.05
H_J (Hz)	[0.001 02]	0.001 02	Φ_J (Hz)	[0.000 342]	0.000 342
H_{JK} (Hz)	[−0.004 06]	−0.004 06	Φ_{JK} (Hz)	[0.000 518]	0.000 518
H_{KJ} (Hz)	[0.005 08]	0.005 08	Φ_{KJ} (Hz)	[−0.003 39]	−0.003 39
H_K (Hz)	[−0.002 04]	−0.002 04	Φ_K (Hz)	[0.003 89]	0.003 89
h_1 (Hz)	[0.000 174]	0.000 174	ϕ_J (Hz)	[0.000 172]	0.000 172
h_2 (Hz)	[0.000 000 400]	0.000 000 400	ϕ_{JK} (Hz)	[0.000 522]	0.000 522
h_3 (Hz)	[−0.000 005 90]	−0.000 005 90	ϕ_K (Hz)	[0.002 19]	0.002 19
N_{lines} [c]	151		N_{lines} [c]	151	
σ_{fit} (MHz)	0.043		σ_{fit} (MHz)	0.043	

[a] Values in brackets are held constant at the computed values. [b] Evaluated using the cc-pCVTZ basis set. [c] Number of independent transitions.

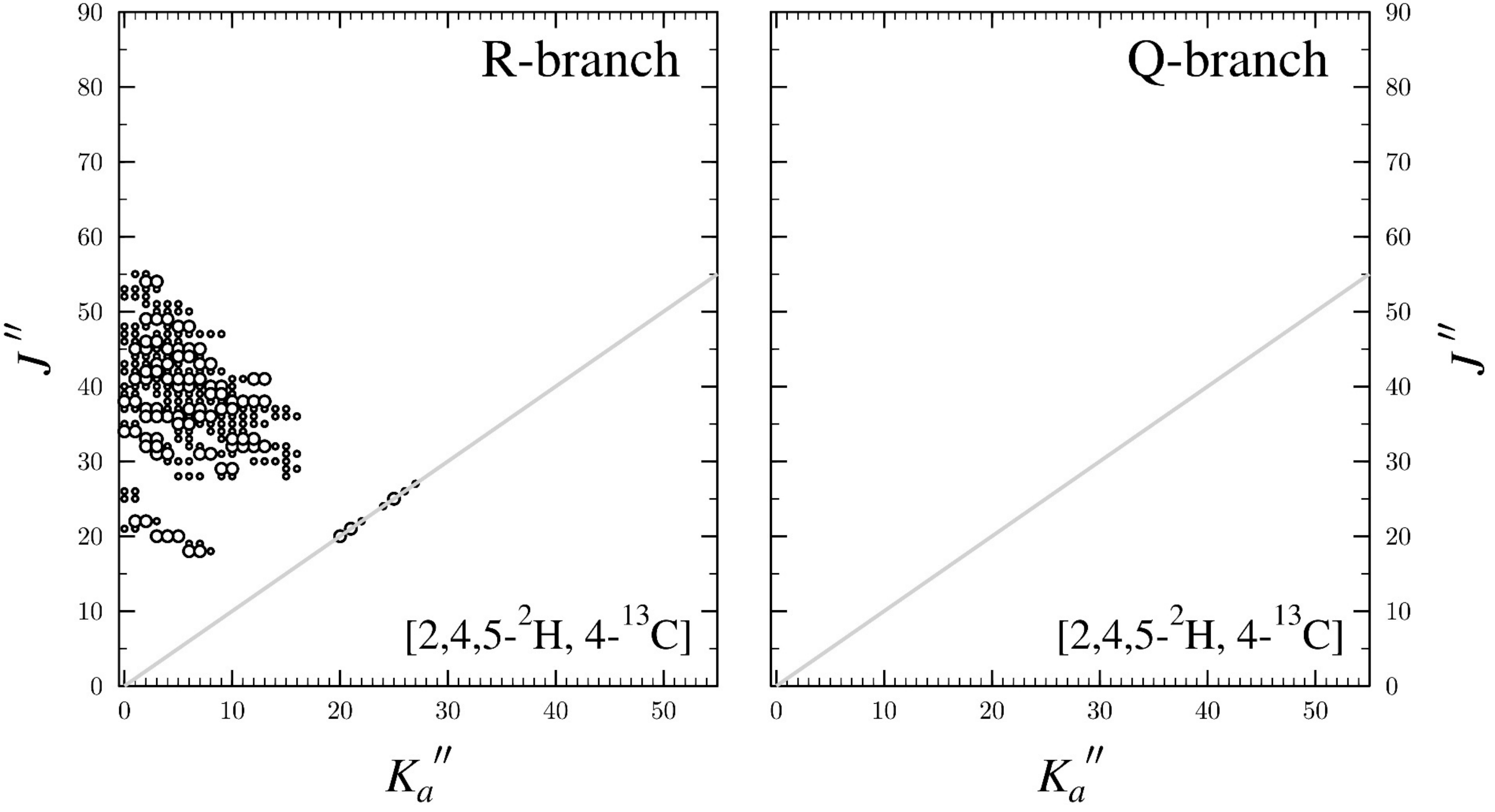

Figure S29. Data distribution plot for the least-squares fit of millimeter-wave spectroscopic data for [2,4,5-^{2}H, 4-^{13}C]-oxazole, ground vibrational state. The size of the outlined circle is proportional to the value of $|(f_{obs.} - f_{calc.})/\delta f|$, where δf is the frequency measurement uncertainty (50 kHz), and no quotient values are larger than three.

Table S30. Experimental and computed spectroscopic constants for [2,4,5-^{2}H, 5-^{13}C]-oxazole [a]

	S Reduction, III^{r} representation			A Reduction, I^{r} representation	
	Experimental	CCSD(T) [b]		Experimental	CCSD(T) [b]
A_0 (MHz)	8924.645 (14)	8867	A_0 (MHz)	8924.666 (14)	8867
B_0 (MHz)	8157.699 (13)	8134	B_0 (MHz)	8157.682 (13)	8134
C_0 (MHz)	4260.035 14 (28)	4240	C_0 (MHz)	4260.034 82 (28)	4240
D_J (kHz)	2.651 25 (75)	2.59	Δ_J (kHz)	1.283 202 (68)	1.28
D_{JK} (kHz)	−4.155 6 (16)	−4.06	Δ_{JK} (kHz)	−0.024 26 (92)	−0.022 2
D_K (kHz)	1.786 82 (94)	1.74	Δ_K (kHz)	1.773 5 (30)	1.60
d_1 (kHz)	[−0.144]	−0.144	δ_J (kHz)	[0.500]	0.500
d_2 (kHz)	[0.014 4]	0.014 4	δ_K (kHz)	[1.08]	1.08
H_J (Hz)	[0.001 03]	0.001 03	Φ_J (Hz)	[0.000 311]	0.000 311
H_{JK} (Hz)	[−0.004 07]	−0.004 07	Φ_{JK} (Hz)	[0.000 843]	0.000 843
H_{KJ} (Hz)	[0.005 08]	0.005 08	Φ_{KJ} (Hz)	[−0.004 04]	−0.004 04
H_K (Hz)	[−0.002 04]	−0.002 04	Φ_K (Hz)	[0.004 28]	0.004 28
h_1 (Hz)	[0.000 189]	0.000 189	ϕ_J (Hz)	[0.000 157]	0.000 157
h_2 (Hz)	[−0.000 009 30]	−0.000 009 30	ϕ_{JK} (Hz)	[0.000 621]	0.000 621
h_3 (Hz)	[0.000 003 20]	0.000 00 320	ϕ_K (Hz)	[0.002 06]	0.002 06
N_{lines} [c]	163		N_{lines} [c]	163	
σ_{fit} (MHz)	0.044		σ_{fit} (MHz)	0.044	

[a] Values in brackets are held constant at the computed values. [b] Evaluated using the cc-pCVTZ basis set. [c] Number of independent transitions.

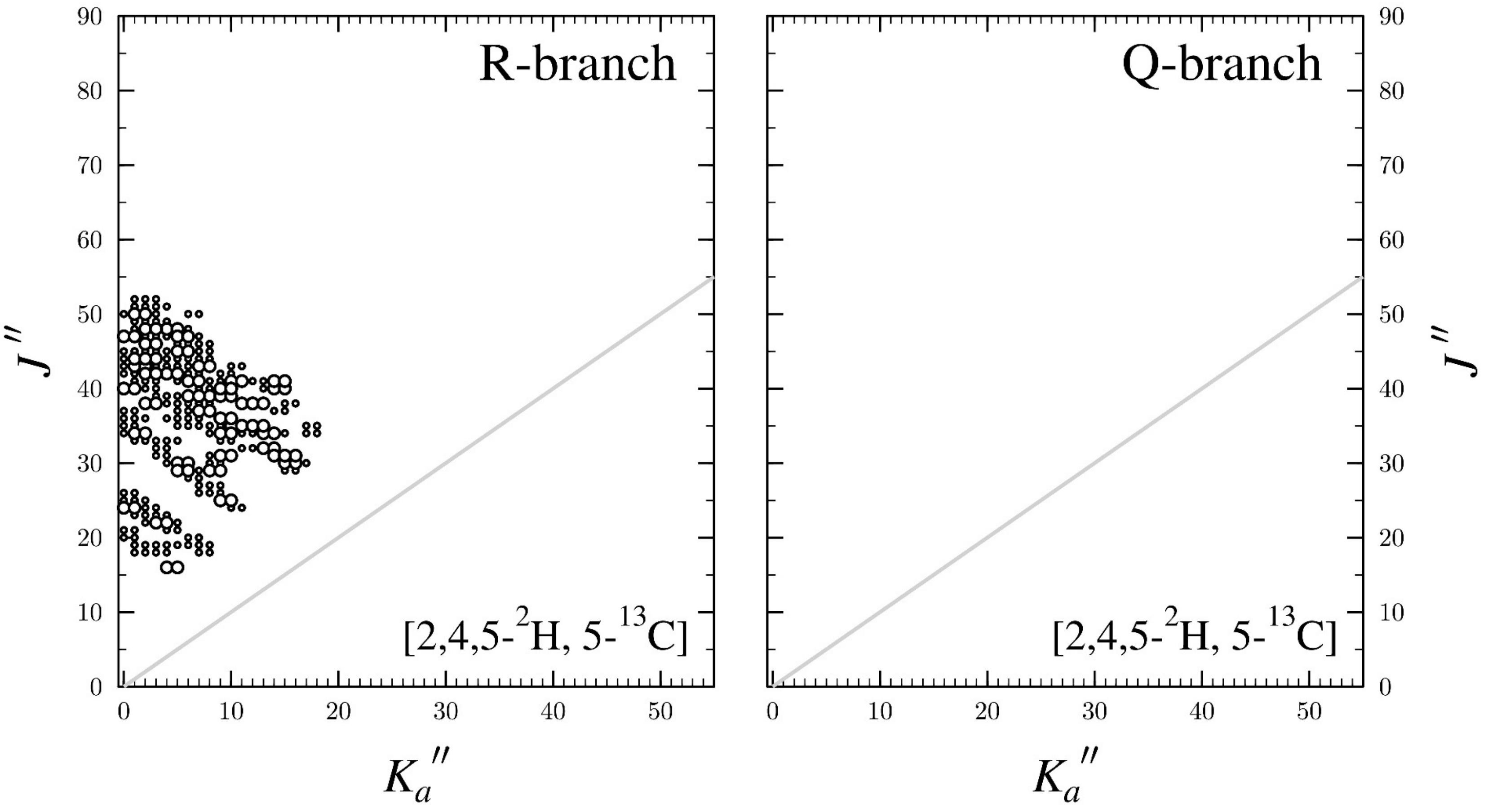

Figure S30. Data distribution plot for the least-squares fit of millimeter-wave spectroscopic data for [2,4,5-^{2}H, 5-^{13}C]-oxazole, ground vibrational state. The size of the outlined circle is proportional to the value of $|(f_{obs.} - f_{calc.})/\delta f|$, where δf is the frequency measurement uncertainty (50 kHz), and no quotient values are larger than three.

Table S31. Determinable constants calculated from A- and S-reduction rotational constants and agreement between them for oxazole isotopologues [a]

Isotopologue	C_3H_3NO	[2-^{13}C]-	[4-^{13}C]-	[5-^{13}C]-	[3-^{15}N]-	[1-^{18}O]-
$A_0''^{(A)}$ (MHz)	10050.9955543	9835.0592428	9899.1172704	9938.1214657	10042.2744503	10014.3509403
$B_0''^{(A)}$ (MHz)	9645.7325616	9636.7163713	9539.3181040	9512.5636649	9395.3599778	9232.0485720
$C_0''^{(A)}$ (MHz)	4919.4107242	4864.7884412	4855.2969045	4857.6984337	4851.3843198	4801.0387116
$A_0''^{(S)}$ (MHz)	10051.0015439	9835.0651186	9899.1232874	9938.1271607	10042.2803870	10014.3568751
$B_0''^{(S)}$ (MHz)	9645.7324826	9636.7163768	9539.3178199	9512.5636535	9395.3596245	9232.0481588
$C_0''^{(S)}$ (MHz)	4919.4048215	4864.7826159	4855.2911883	4857.6927433	4851.3788891	4801.0332110
A_0'' Mean (MHz)	10050.9977	9835.0615	9899.1195	9938.1236	10042.2767	10014.3531
B_0'' Mean (MHz)	9645.731630	9636.7155584	9539.31699	9512.562912	9395.35887	9232.04739
C_0'' Mean (MHz)	4919.4039	4864.7819	4855.2902	4857.6920	4851.3780	4801.0322
$A_0''^{(A)}-A_0''^{(S)}$ (MHz)	−0.0060	−0.0059	−0.0060	−0.0057	−0.0059	−0.0059
$B_0''^{(A)}-B_0''^{(S)}$ (MHz)	0.000079	−0.0000055	0.00028	0.000011	0.00035	0.00041
$C_0''^{(A)}-C_0''^{(S)}$ (MHz)	0.0059	0.0058	0.0057	0.0057	0.0054	0.0055

[a] Values were calculated from unrounded values; values in table are rounded for ease of reading.

Table S31 Continued. Determinable constants calculated from A- and S-reduction rotational constants and agreement between them for oxazole isotopologues [a]

Isotopologue	[2-^{2}H]-	[4-^{2}H]-	[5-^{2}H]-	[2-^{2}H, 2-^{13}C]-	[2-^{2}H, 4-^{13}C]-	[2-^{2}H, 5-^{13}C]-
$A_0''^{(A)}$ (MHz)	9649.6608546	9801.5180300	9884.5248089	9650.2786789	9580.0925464	9602.7807582
$B_0''^{(A)}$ (MHz)	9207.2783737	9005.6934528	8959.6336724	9027.9343291	9036.8570328	9025.2185012
$C_0''^{(A)}$ (MHz)	4709.2302561	4690.9437213	4697.2918240	4661.9918133	4647.9018194	4650.1525823
$A_0''^{(S)}$ (MHz)	9649.6664161	9801.5237687	9884.5302492	9650.2842583	9580.0975118	9602.7860246
$B_0''^{(S)}$ (MHz)	9207.2777797	9005.6924931	8959.6331217	9027.9341488	9036.8571822	9025.2176727
$C_0''^{(S)}$ (MHz)	4709.2252798	4690.9389861	4697.2869392	4661.9876084	4647.8976294	4650.1470266
A_0'' Mean (MHz)	9649.6630	9801.5205	9884.5270	9650.2809	9580.0942	9602.7827
B_0'' Mean (MHz)	9207.27713	9005.6921	8959.63262	9027.93354	9036.85664	9025.21702
C_0'' Mean (MHz)	4709.2243	4690.9381	4697.2862	4661.9867	4647.8967	4650.1461
$A_0''^{(A)}-A_0''^{(S)}$ (MHz)	−0.0056	−0.0057	−0.0054	−0.0056	−0.0050	−0.0053
$B_0''^{(A)}-B_0''^{(S)}$ (MHz)	0.00059	0.0010	0.00055	0.00018	−0.00015	0.00083
$C_0''^{(A)}-C_0''^{(S)}$ (MHz)	0.0050	0.0047	0.0049	0.0042	0.0042	0.0056

Table S31 Continued. Determinable constants calculated from A- and S-reduction rotational constants and agreement between them for oxazole isotopologues [a]

Isotopologue	[2-^{2}H, 3-^{15}N]-	[2-^{2}H, 1-^{18}O]-	[2,5-^{2}H]-	[2,5-^{2}H, 2-^{13}C]-	[2,5-^{2}H, 4-^{13}C]-	[2,5-^{2}H, 5-^{13}C]-
$A_0''^{(A)}$ (MHz)	9444.5484335	9315.3243687	9530.6827144	9504.0230920	9338.6281035	9529.0287792
$B_0''^{(A)}$ (MHz)	9160.2482532	9106.1913358	8536.9219637	8396.0689796	8492.9465995	8356.2140577
$C_0''^{(A)}$ (MHz)	4647.7384144	4602.4396499	4501.0868940	4455.7581196	4445.7459767	4449.9688384
$A_0''^{(S)}$ (MHz)	9444.5377026	9315.3112695	9530.6877175	9504.0275008	9338.6335499	9529.0339860
$B_0''^{(S)}$ (MHz)	9160.2634347	9106.2080604	8536.9212973	8396.0685505	8492.9457915	8356.2132428
$C_0''^{(S)}$ (MHz)	4647.7333498	4602.4348862	4501.0825540	4455.7536613	4445.7415832	4449.9644891
A_0'' Mean (MHz)	9444.534	9315.308	9530.6847	9504.0246	9338.6306	9529.0311
B_0'' Mean (MHz)	9160.263	9106.208	8536.92081	8396.06809	8492.94529	8356.21280
C_0'' Mean (MHz)	4647.7326	4602.4341	4501.0817	4455.7528	4445.7408	4449.9637
$A_0''^{(A)}-A_0''^{(S)}$ (MHz)	0.011	0.013	−0.0050	−0.0044	−0.0054	−0.0052
$B_0''^{(A)}-B_0''^{(S)}$ (MHz)	−0.015	−0.017	0.00067	0.00043	0.00081	0.00081
$C_0''^{(A)}-C_0''^{(S)}$ (MHz)	0.0051	0.0048	0.0043	0.0045	0.0044	0.0043

[a] Values were calculated from unrounded values; values in table are rounded for ease of reading.

Table S31 Continued. Determinable constants calculated from A- and S-reduction rotational constants and agreement between them for oxazole isotopologues [a]

Isotopologue	[2,5-^{2}H, 3-^{15}N]-	[2,5-^{2}H, 1-^{18}O]-	[2,4-^{2}H]-	[4,5-^{2}H]-	[2,4,5-^{2}H]-	[5-^{2}H, 2-^{13}C]-
$A_0''^{(A)}$ (MHz)	9416.0995685	9128.1189832	9510.5261603	9007.9073886	8978.0461570	9774.0224686
$B_0''^{(A)}$ (MHz)	8417.8649057	8522.5412537	8531.2551037	8959.7367936	8282.2251925	8860.4696190
$C_0''^{(A)}$ (MHz)	4442.4064259	4405.3701028	4494.9711420	4489.6837636	4306.0905047	4645.0683675
$A_0''^{(S)}$ (MHz)	9416.1067667	9128.1677371	9510.5315134	9007.9120229	8978.0508663	9774.0071288
$B_0''^{(S)}$ (MHz)	8417.8655297	8522.4997627	8531.2533941	8959.7369086	8282.2244304	8860.4876125
$C_0''^{(S)}$ (MHz)	4442.4019707	4405.3665007	4494.9669889	4489.6788724	4306.0865836	4645.0635569
A_0'' Mean (MHz)	9416.104	9128.165	9510.5286	9007.9091	8978.0481	9774.004
B_0'' Mean (MHz)	8417.865	8522.499	8531.2531	8959.7363	8282.22399	8860.487
C_0'' Mean (MHz)	4442.4012	4405.3657	4494.9661	4489.6783	4306.0858	4645.0628
$A_0''^{(A)}-A_0''^{(S)}$ (MHz)	−0.007	−0.049	−0.0054	−0.0046	−0.0047	0.015
$B_0''^{(A)}-B_0''^{(S)}$ (MHz)	−0.001	0.041	0.0017	−0.0001	0.00076	−0.018
$C_0''^{(A)}-C_0''^{(S)}$ (MHz)	0.0045	0.0036	0.0042	0.0049	0.0039	0.0048

Table S31 Continued. Determinable constants calculated from A- and S-reduction rotational constants and agreement between them for oxazole isotopologues [a]

Isotopologue	[5-^{2}H, 4-^{13}C]-	[5-^{2}H, 5-^{13}C]-	[5-^{2}H, 3-^{15}N]-	[2,4,5-^{2}H, 2-^{13}C]-	[2,4,5-^{2}H, 4-^{13}C]-	[2,4,5-^{2}H, 5-^{13}C]-
$A_0''^{(A)}$ (MHz)	9631.6374497	9868.5543445	9859.3833270	8978.2946624	8911.8261746	8924.6685664
$B_0''^{(A)}$ (MHz)	8960.0840471	8780.6509745	8746.4889745	8123.2292562	8161.2695822	8157.6813909
$C_0''^{(A)}$ (MHz)	4639.4980818	4644.0820698	4632.4528715	4262.7619144	4258.0864274	4260.0405134
$A_0''^{(S)}$ (MHz)	9631.6172763	9868.5412569	9859.4362714	8978.2970357	8911.8309506	8924.6503888
$B_0''^{(S)}$ (MHz)	8960.1070236	8780.6659148	8746.4509174	8123.2296097	8161.2685203	8157.6999158
$C_0''^{(S)}$ (MHz)	4639.4932217	4644.0774033	4632.4481870	4262.7581745	4258.0826197	4260.0366331
A_0'' Mean (MHz)	9631.614	9868.538	9859.433	8978.2944	8911.8283	8924.648
B_0'' Mean (MHz)	8960.107	8780.665	8746.450	8123.22920	8161.2681	8157.699
C_0'' Mean (MHz)	4639.4925	4644.0766	4632.4474	4262.7574	4258.0819	4260.0359
$A_0''^{(A)}-A_0''^{(S)}$ (MHz)	0.020	0.013	−0.053	−0.0024	−0.0048	0.018
$B_0''^{(A)}-B_0''^{(S)}$ (MHz)	−0.023	−0.015	0.038	−0.00035	0.0011	−0.019
$C_0''^{(A)}-C_0''^{(S)}$ (MHz)	0.0049	0.0047	0.0047	0.0037	0.0038	0.0039

[a] Values were calculated from unrounded values; values in table are rounded for ease of reading.

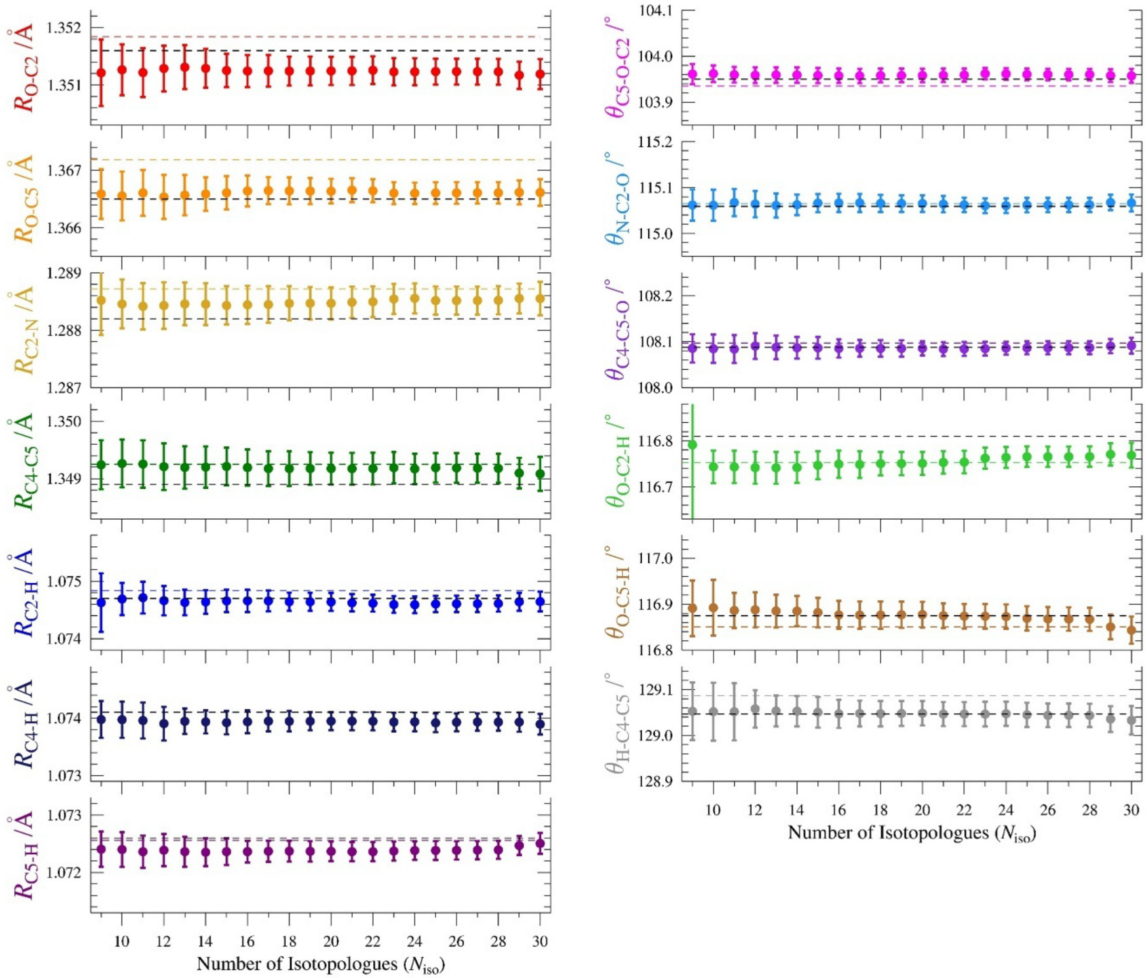

N_{iso}	Isotopologue Added
9	Core set
10	[2-^{2}H, 1-^{18}O]
11	[2,5-^{2}H, 2-^{13}C]
12	[2,4,5-^{2}H, 5-^{13}C]
13	[2,5-^{2}H, 4-^{13}C]
14	[2,4,5-^{2}H, 2-^{13}C]
15	[2,5-^{2}H, 3-^{15}N]
16	[2-^{2}H, 5-^{13}C]
17	[2-^{2}H, 4-^{13}C]
18	[5-^{2}H, 2-^{13}C]
19	[5-^{2}H, 4-^{13}C]
20	[2,5-^{2}H, 5-^{13}C]
21	[2,4,5-^{2}H, 4-^{13}C]
22	[2-^{2}H, 2-^{13}C]
23	[2,5-^{2}H, 1-^{18}O]
24	[5-^{2}H, 5-^{13}C]
25	[2-^{2}H, 3-^{15}N]
26	[2,4-^{2}H]
27	[2,4,5-^{2}H]
28	[2,5-^{2}H]
29	[5-^{2}H, 3-^{15}N]
30	[4,5-^{2}H]

Figure S31. Plots of the structural parameters as a function of the number of isotopologues (N_{iso}) and their 2σ uncertainties with consistent scales for each distance (0.0020 Å) and each angle (0.25°). The black dashed line in each plot is the r_s^{SE} (using 27 isotopologues) value for that parameter, and the dashed line matching the color of the plotted markers is the BTE value calculated for that parameter. The isotopologue included in the structural least-squares fit corresponding to the total number along the *x*-axis is provided in the table to the right. This plot corresponds to the r_e^{SE}(final), after addressing the r_e^{SE}-r_e discrepancy. The isotopologues whose labels are in maroon font are suspect due to issues discussed in the manuscript.

r_e *vs.* r_e^{SE} Principal Axis Rotation Analysis

In addition to ensuring that the least-squares fits of the spectroscopic data are good quality (no outlier transitions, constants determined with reasonable uncertainty, constants appear to be physically meaningful rather than empirical, *etc.*), that there is good agreement between the determinable constants computed from the A and S reductions, and that the inertial defects or planar second moments do not provide cause for concern, it is valuable to examine whether the principal axes of any isotopologues are differentially rotated in the computed r_e structure used to obtain vibration-rotation interaction corrections and the semiexperimental equilibrium (r_e^{SE}) structure. If there is a large difference in the extent of rotation of the isotopologue between these two structures, it is likely that the vibration-rotation interaction corrections are not sufficiently accurate for that isotopologue to generate small residuals and may be negatively impacting the precision and accuracy of the determined r_e^{SE} structure.

Herein, we provide an example of such an analysis for the convenience of future analysts.

To compare the angles of rotation, the atomic masses and their coordinates in the r_e and r_e^{SE} structures are used to calculate the center-of-mass coordinates for each isotopologue based on a reference set of input coordinates, such as those of the normal isotopologue.

For a planar molecule lying in x,y plane, the atomic coordinates (x_i, y_i, z_i) and the exact mass, m_i, of each atom, i, are used to calculate the following moments and products of inertia of the structures, per equation 2.3, page 12, from Gordy and Cook:[1]

$$I_{xx} = \sum_i m_i y_i^2 \quad \text{(S14)}$$

$$I_{yy} = \sum_i m_i x_i^2 \quad \text{(S15)}$$

$$I_{xy} = -\sum_i m_i x_i y_i \quad \text{(S16)}$$

When the I_{xx} and I_{yy} values are mapped onto I_{aa} and I_{bb}, they must follow the convention that $I_{aa} < I_{bb}$, *i.e.*, $A > B$. The a-axis and b-axis moments of inertia are used to calculate the angle, θ_i, for each isotopologue, per equation 7 from Demaison *et al*:[12]

$$\theta_i = \frac{1}{2}\tan^{-1}\frac{2I_{ab}}{I_{aa}-I_{bb}} \quad \text{(S17)}$$

This angle is the angle of rotation between the input coordinate system and the principal axis system. While this angle can vary depending on the input coordinates and does not have intrinsic meaning, it enables useful comparison. Subsequently, the relative angle (θ_r) for isotopologue i is calculated is calculated with respect to the normal isotopologue:

$$\theta_r = \theta_i - \theta_n \quad \text{(S18)}$$

where θ_n is the value of θ_i for the normal isotopologue. This relative angle is the degree of rotation between the principal axes of the selected isotopologue and the normal isotopologue. these values are compared for the r_e and r_e^{SE} structures. If there is a large difference between this rotation for the r_e and r_e^{SE} structures and a large difference between the semi-experimental and predicted (based on the semi-experimental structure) equilibrium rotational constants, then the difference in rotation between the two structures may be problematic.

An example of this analysis is provided for [4,5-^{2}H]-oxazole (C_s). The products, moments, and angles shown below are rounded for legibility, but the analysis using the masses and coordinates provided, without rounding, yields the values provided below.

Table S32. Center-of-Mass Coordinates, exact masses, and products for the r_e and r_e^{SE} structures of the normal isotopologue of oxazole

		r_e Structure					r_e^{SE} Structure				
Atom	Mass, m_i	x_i	y_i	$m_ix_iy_i$	$m_iy_i^2$	$m_ix_i^2$	x_i	y_i	$m_ix_iy_i$	$m_iy_i^2$	$m_ix_i^2$
O1	15.994914630	−0.729021	−0.878242	10.24085	12.33702	8.500843	−0.724709	−0.876506	10.16016	12.2883	8.400579
C2	12.000000000	0.625224	−0.878242	−6.58918	9.255708	4.690861	0.62654	−0.876506	−6.58999	9.219153	4.710628
C5	12.000000000	−1.055598	0.452109	−5.72694	2.452831	13.37145	−1.054121	0.449987	−5.69209	2.42986	13.33405
N3	14.003074002	1.177926	0.289605	4.776914	1.174453	19.4294	1.172587	0.289948	4.760895	1.177236	19.25367
C4	12.000000000	0.09219	1.167324	1.291387	16.35174	0.101988	0.089727	1.165365	1.254776	16.29691	0.096611
H2	1.007825035	1.107699	−1.839738	−2.05382	3.411121	1.236598	1.108771	−1.837252	−2.05303	3.401908	1.238993
H5	1.007825035	−2.101496	0.694611	−1.47114	0.48626	4.450843	−2.098832	0.690921	−1.46147	0.481107	4.439566
H4	1.007825035	0.224072	2.234373	0.504578	5.031489	0.050601	0.222158	2.231152	0.499547	5.016993	0.04974

Therefore, for the r_e structure of the normal isotopologue of oxazole, I_{xx} = 50.5006 uÅ^2, I_{yy} = 51.8326 uÅ^2, and I_{xy} = −0.972646 uÅ^2. For the r_e^{SE} structure, I_{xx} = 50.3115 uÅ^2, I_{yy} = 51.5238 uÅ^2, and I_{xy} = −0.878788 uÅ^2. The resultant angles are 27.8° for the r_e structure and 27.7° for the r_e^{SE} structure.

Table S33. Center-of-Mass Coordinates, exact masses, and products for the r_e and r_e^{SE} structures of [4,5-^{2}H]-oxazole

		r_e Structure					r_e^{SE} Structure				
Atom	Mass, m_i	x_i	y_i	$m_ix_iy_i$	$m_iy_i^2$	$m_ix_i^2$	x_i	y_i	$m_ix_iy_i$	$m_iy_i^2$	$m_ix_i^2$
O1	15.994914630	−0.702426	−0.919735	10.33345	13.5303	7.891927	−0.698124	−0.9179	10.24967	13.47636	7.795555
C2	12.000000000	0.65182	−0.919735	−7.19402	10.15095	5.098432	0.653125	−0.9179	−7.19404	10.11048	5.118867
C5	12.000000000	−1.029003	0.410616	−5.0703	2.023266	12.70617	−1.027536	0.408593	−5.03813	2.003379	12.66996
N3	14.003074002	1.204522	0.248112	4.184908	0.862023	20.31669	1.199172	0.248554	4.173742	0.865097	20.13661
C4	12.000000000	0.118786	1.125832	1.604797	15.20997	0.169321	0.116312	1.123971	1.568776	15.15973	0.162342
H2	1.007825035	1.134295	−1.88123	−2.15057	3.566719	1.296693	1.135357	−1.878646	−2.14962	3.556928	1.299122
H5	2.014101779	−2.0749	0.653118	−2.72942	0.859142	8.671131	−2.072247	0.649526	−2.71094	0.849717	8.648971
H4	2.014101779	0.250668	2.19288	1.107121	9.685257	0.126555	0.248743	2.189758	1.097055	9.657699	0.124619

Therefore, for the r_e structure of [4,5-^{2}H]-oxazole, I_{xx} = 55.8876 uÅ^2, I_{yy} = 56.2769 uÅ^2, and I_{xy} = −0.085965 uÅ^2. For the r_e^{SE} structure, I_{xx} = 55.6794 uÅ^2, I_{yy} = 55.9560 uÅ^2, and I_{xy} = 0.003488 uÅ^2. The resultant angles are 11.9° for the r_e structure and −0.7° for the r_e^{SE} structure.

The analysis demonstrates that while there is a relative rotation of 15.9° from the normal to [4,5-^{2}H]-oxazole using the r_e structure, there is a relative rotation of 28.4° using the r_e^{SE} structure. $\theta r_e - \theta r_e^{SE}$ = 12.5° Thus, small changes between r_e and r_e^{SE} structures result in a meaningful difference in the orientation of principal axes of the isotopologues. As a result, a portion of the computed vibration-rotation interaction constants will incorrectly transfer between the A_e−A_0 and B_e−B_0 values for the affected isotopologues.

Table S34. Angles of rotation of principal axes relative to the normal isotopologue using different oxazole structures

Isotopologue	θr_e (°)	θr_e^{SE} (initial) (°)	θr_e^{SE} (final) (°)	θr_e^{SE} (2nd iteration) (°)
C_3H_3NO	0.00	0.00	0.00	0.00
[2-^{13}C]	−6.82	−8.40	−8.34	−8.34
[4-^{13}C]	17.10	15.51	15.43	15.44
[5-^{13}C]	−15.72	−17.09	−17.08	−17.08
[3-^{15}N]	−5.29	−5.52	−5.53	−5.53
[1-^{18}O]	11.69	12.35	12.32	12.32
[2-^{2}H]	−62.26	−61.77	−61.89	−61.89
[2-^{2}H,2-^{13}C]	−62.63	−62.26	−62.37	−62.36
[2-^{2}H,4-^{13}C]	−48.26	−48.87	−48.96	−48.96
[2-^{2}H,5-^{13}C]	−71.87	−72.63	−72.75	−72.74
[2-^{2}H,3-^{15}N]	−64.29	−67.26	−67.20	−67.20
[2-^{2}H,1-^{18}O]	−18.68	−23.84	−23.88	−23.88
[4-^{2}H]	−8.07	−9.35	−9.43	−9.43
[5-^{2}H]	−32.95	−33.88	−33.86	−33.86
[5-^{2}H,2-^{13}C]	−39.46	−40.46	−40.45	−40.44
[5-^{2}H,4-^{13}C]	−32.65	−33.97	−33.93	−33.93
[5-^{2}H,5-^{13}C]	−36.25	−37.03	−37.02	−37.02
[5-^{2}H,3-^{15}N] [c]	−29.04	−29.73	−29.72	−29.71
[2,4-^{2}H]	−33.44	−34.16	−34.26	−34.26
[2,5-^{2}H]	−57.12	−57.89	−57.87	−57.87
[2,5-^{2}H,2-^{13}C]	−60.70	−61.30	−61.29	−61.29
[2,5-^{2}H,4-^{13}C]	−62.94	−63.71	−63.69	−63.68
[2,5-^{2}H,5-^{13}C]	−56.22	−56.84	−56.83	−56.83
[2,5-^{2}H,3-^{15}N]	−50.24	−51.09	−51.08	−51.07
[2,5-^{2}H,1-^{18}O]	−50.58	−52.07	−52.03	−52.02
[4,5-^{2}H] [a]	−15.89	−28.42	−28.17	−28.20
[2,4,5-^{2}H]	−60.58	−60.37	−60.49	−60.49
[2,4,5-^{2}H,2-^{13}C]	−61.14	−60.96	−61.06	−61.06
[2,4,5-^{2}H,4-^{13}C]	−53.08	−53.21	−53.32	−53.31
[2,4,5-^{2}H,5-^{13}C]	−67.36	−66.89	−67.01	−67.00

[a] The isotopologue with a large difference in the relative angles of rotation between r_e and r_e^{SE} structures is highlighted in light blue.

General relation of the observable ground vibrational state ($B_{v1v2v3...}$) and equilibrium (B_e) rotational constants for an asymmetric-top molecule.[13-14]

The vibration-rotation interaction correction that relates these values involves a first-order α term, which is calculated by modern software and used to reasonably approximate the vibration-rotation interaction constant, and a second-order γ term that has not been implemented in any published computational package to date.[14] The vibration-rotation interaction correction involves higher-order terms – the ε term being the next higher-order term – but these are not shown here as the primary purpose of this equation is to give the reader a sense of the γ contribution to the vibration-rotation interaction correction and because the higher-order terms are expected to have successively less impact on the value of the vibration-rotation interaction correction.

$$B_{v1v2v3\cdots} = B_e - \sum_i^{3N-6} \alpha_i^B \left(v_i + \tfrac{1}{2}\right) + \sum_{ij}^{3N-6} \gamma_{ij}^B \left(v_i + \tfrac{1}{2}\right)\left(v_j + \tfrac{1}{2}\right) \quad \text{(S19)}$$

Table S35. Vibration-Rotation Interaction Constants (α) Evaluated at Different Geometries

Geometry	r_e CCSD(T)/cc-pCTZ			r_e^{SE} (initial)			r_e^{SE} (final)		
Isotopologue	α_A (MHz)	α_B (MHz)	α_C (MHz)	α_A (MHz)	α_B (MHz)	α_C (MHz)	α_A (MHz)	α_B (MHz)	α_C (MHz)
Normal	86.48915909	75.25663162	43.39806335	85.87623551	74.10630224	42.92228594	85.86387825	74.12965270	42.92509802
[2-^{13}C]-	83.06525890	75.70244566	42.67701557	82.14904608	74.88507382	42.21295186	82.15825747	74.89099170	42.21645977
[4-^{13}C]-	86.39215871	72.19629637	42.53588392	85.83413044	71.02634684	42.07110415	85.81568736	71.05698853	42.07419968
[5-^{13}C]-	81.99385317	76.56753931	42.61612423	81.05593134	75.77895253	42.15109478	81.05779255	75.78546010	42.15306140
[3-^{15}N]-	85.14339149	73.49517729	42.51526086	84.48798721	72.42042196	42.04552465	84.47650936	72.44100431	42.04807470
[1-^{18}O]-	86.87681858	70.28308675	41.98006773	86.31600474	69.14807794	41.51804833	86.29633987	69.17662995	41.52088896
[2-^{2}H]-	73.95746083	77.02319096	40.55803809	72.89325668	76.41037019	40.11451680	72.91397944	76.38846511	40.11298820
[4-^{2}H]-	84.51795796	66.43534906	40.20822084	83.58779243	65.69974857	39.76046706	83.58635502	65.71276017	39.76349218
[5-^{2}H]-	78.27282866	71.94406730	40.23915810	77.37543484	71.15853640	39.78340339	77.38418448	71.17131264	39.78909539
[2-^{2}H,2-^{13}C]-	73.25376031	75.16327797	39.89376561	72.19081594	74.57733249	39.46035099	72.21290040	74.55703869	39.45934792
[2-^{2}H,4-^{13}C]-	74.96425908	73.19030723	39.75964640	73.76607509	72.74376517	39.32615493	73.79087297	72.71967392	39.32491887
[2-^{2}H,5-^{13}C]-	72.05253365	76.07950650	39.83755295	71.06142964	75.42746226	39.40385122	71.07903994	75.40614282	39.40165916
[2-^{2}H,3-^{15}N]-	70.49547412	77.54682479	39.78172429	69.55696216	76.83991519	39.34387606	69.57087055	76.82279512	39.34219525
[2-^{2}H,1-^{18}O]-	77.11490838	69.51319352	39.30685169	75.56494684	69.44972492	38.87576569	75.58441538	69.42831395	38.87452881
[2,5-^{2}H]-	71.31561878	69.32695902	37.68344552	70.34825756	68.69091458	37.25801552	70.35974865	68.68800326	37.25951304
[2,5-^{2}H,2-^{13}C]-	70.32028068	68.00169859	37.07526758	69.35149593	67.39310939	36.65941479	69.36705211	67.38889892	36.66133280
[2,5-^{2}H,4-^{13}C]-	69.16194526	68.78361619	36.98832043	68.18583488	68.18675516	36.57253948	68.20471844	68.17833883	36.57420210
[2,5-^{2}H,5-^{13}C]-	70.86091278	67.37981136	37.02394535	69.91791891	66.74973312	36.60710514	69.92758213	66.74646336	36.60806582
[2,5-^{2}H,3-^{15}N]-	70.44757601	67.58391748	36.96643278	69.52903578	66.92894942	36.54619068	69.53282108	66.93149343	36.54754048
[2,5-^{2}H,1-^{18}O]-	68.31827443	68.25745706	36.59356832	67.39687695	67.63513298	36.17984297	67.40317541	67.63628835	36.18142055
[2,4-^{2}H]-	77.13456445	64.36880515	37.64652134	76.02296627	63.89524076	37.22821986	76.03844864	63.88077676	37.22712541
[4,5-^{2}H]-[a]	70.51997671	69.25254890	37.45700159	67.94200913	70.23608087	37.02855003	67.98307993	70.21672677	37.03410826
[2,4,5-^{2}H]-	66.15799092	65.22110324	35.13783889	65.16191627	64.69731131	34.73698073	65.18617134	64.68355428	34.73856119
[5-^{2}H,2-^{13}C]-	75.81128787	71.73160364	39.58242933	74.85344183	71.03383426	39.13771287	74.87064804	71.04192292	39.14397546
[5-^{2}H,4-^{13}C]-	75.80317235	71.43980770	39.48990647	74.87210674	70.72112495	39.04468546	74.88434498	70.73161525	39.05053117
[5-^{2}H,5-^{13}C]-	77.05856216	70.49588323	39.52660187	76.16904262	69.73520751	39.08017199	76.17968033	69.74386128	39.08517792
[5-^{2}H,3-^{15}N]-	78.38258161	69.11218693	39.42534634	77.56886950	68.27415837	38.97507062	77.57224096	68.29018873	38.98051203
[2,4,5-^{2}H,2-^{13}C]-	65.54924274	63.71951769	34.57737065	64.56645727	63.20735105	34.18533016	64.59068895	63.19586670	34.18733508
[2,4,5-^{2}H,4-^{13}C]-	66.03768279	63.07922751	34.50357610	65.01267789	62.61157020	34.11113237	65.04195395	62.59434065	34.11284091
[2,4,5-^{2}H,5-^{13}C]-	64.62993404	64.47879028	34.56089182	63.68403729	63.93402685	34.16810284	63.70362365	63.92246884	34.16918550

[a] The isotopologue with large residuals is highlighted in light blue.

Table S36. Rotational constant residuals from the r_e^{SE} structure of oxazole before and after application of r_e^{SE}-based vibration-rotation interaction corrections [a,b]

Isotopologue	B_e^{xrefit} (initial) − B_e^{SE}			B_e^{xrefit} (final) − B_e^{SE}			B_e^{xrefit} (2nd iteration) − B_e^{SE}		
	ΔA (MHz)	ΔB (MHz)	ΔC (MHz)	ΔA (MHz)	ΔB (MHz)	ΔC (MHz)	ΔA (MHz)	ΔB (MHz)	ΔC (MHz)
Normal	−0.0235	−0.0206	0.0233	−0.1068	−0.0305	0.0299	−0.0867	−0.0324	0.0346
[2-^{13}C]-	−0.2184	0.2510	0.0414	−0.1648	0.0154	0.0256	−0.1567	0.0228	0.0297
[4-^{13}C]-	−0.1033	0.0198	0.0170	−0.1201	−0.0636	0.0181	−0.1316	−0.0497	0.0194
[5-^{13}C]-	−0.2193	0.0934	−0.0010	−0.1509	−0.0274	0.0186	−0.1116	−0.0319	0.0270
[3-^{15}N]-	−0.0259	−0.0341	0.0173	−0.1128	−0.0715	0.0167	−0.0849	−0.0950	0.0172
[1-^{18}O]-	−0.0956	−0.0538	−0.0054	−0.1228	−0.0823	0.0062	−0.1124	−0.0495	0.0179
[2-^{2}H]-	−0.0082	−0.0360	0.0157	−0.0950	−0.0260	0.0269	−0.0838	−0.0450	0.0253
[4-^{2}H]-	−0.1315	0.0849	0.0174	−0.0836	−0.0588	0.0240	−0.1621	0.0513	0.0361
[5-^{2}H]-	−0.0829	0.0383	0.0129	−0.0735	−0.0491	0.0277	0.0387	−0.1351	0.0292
[2-^{2}H,2-^{13}C]-	−0.0345	0.0431	0.0295	−0.1140	−0.0280	0.0206	−0.1030	−0.0465	0.0189
[2-^{2}H,4-^{13}C]-	−0.0208	−0.0336	0.0071	−0.1021	−0.0577	0.0144	−0.1180	−0.0634	0.0098
[2-^{2}H,5-^{13}C]-	−0.1194	−0.0216	−0.0058	−0.1034	−0.0566	0.0164	−0.0738	−0.0777	0.0184
[2-^{2}H,3-^{15}N]-	−0.1928	0.1064	0.0098	−0.1845	0.0108	0.0153	−0.1578	−0.0368	0.0102
[2-^{2}H,1-^{18}O]-	−0.4004	0.3213	−0.0015	−0.1455	−0.0221	0.0092	−0.1999	0.0472	0.0142
[2,5-^{2}H]-	−0.1027	0.0343	0.0057	−0.0629	−0.0508	0.0234	−0.0787	−0.0516	0.0199
[2,5-^{2}H,2-^{13}C]-	0.0273	−0.0291	0.0180	−0.0701	−0.0634	0.0180	−0.1110	−0.0440	0.0148
[2,5-^{2}H,4-^{13}C]-	0.0475	−0.1097	0.0008	−0.0558	−0.0922	0.0127	−0.1108	−0.0718	0.0061
[2,5-^{2}H,5-^{13}C]-	−0.1409	−0.0069	−0.0137	−0.0920	−0.0618	0.0135	−0.1010	−0.0573	0.0131
[2,5-^{2}H,3-^{15}N]-	−0.2497	0.1362	0.0007	−0.0933	−0.0647	0.0126	−0.0758	−0.1049	0.0055
[2,5-^{2}H,1-^{18}O]-	−0.4385	0.2828	−0.0111	−0.2017	0.0279	0.0084	−0.1566	0.0007	0.0118
[2,4-^{2}H]-	−0.0567	0.0315	0.0087	−0.0728	−0.0523	0.0201	−0.0662	−0.0395	0.0257
[4,5-^{2}H]-[a]	−2.2462	2.1803	0.0088	−0.4199	0.2853	0.0213	−0.3335	0.2339	0.0297
[2,4,5-^{2}H]-	−0.0282	−0.0387	0.0009	−0.0663	−0.0577	0.0167	−0.0863	−0.0306	0.0197
[5-^{2}H,2-^{13}C]-	0.1132	−0.0498	0.0257	−0.0630	−0.0543	0.0208	0.0010	−0.1011	0.0222
[5-^{2}H,4-^{13}C]-	−0.1260	0.0620	0.0086	−0.0735	−0.0904	0.0160	0.0155	−0.1739	0.0141
[5-^{2}H,5-^{13}C]-	0.0598	−0.1359	−0.0118	−0.0251	−0.1047	0.0144	0.0717	−0.1637	0.0191
[5-^{2}H,3-^{15}N]-	−0.4732	0.2745	0.0103	−0.4563	0.1631	0.0179	−0.3242	0.0503	0.0152
[2,4,5-^{2}H,2-^{13}C]-	−0.0528	0.0471	0.0120	−0.0904	−0.0346	0.0101	−0.1077	−0.0109	0.013
[2,4,5-^{2}H,4-^{13}C]-	0.0572	−0.1218	−0.0035	−0.0651	−0.0930	0.0071	−0.1158	−0.0511	0.0072
[2,4,5-^{2}H,5-^{13}C]-	−0.1441	−0.0208	−0.0180	−0.0877	−0.0787	0.0074	−0.0768	−0.0668	0.0134

[a] r_e^{SE} structures from 57 independent moments of inertia.
[b] The isotopologue with large residuals is highlighted in light blue.

Table S37. Fully corrected inertial defects ($\Delta_{i\,e}$) of oxazole isotopologues from various r_e^{SE} structures.

Isotopologue	r_e^{SE}(initial) $\Delta_{i\,e}$ (uÅ^2) [a]	r_e^{SE}(final) $\Delta_{i\,e}$ (uÅ^2) [b]	r_e^{SE}(2nd iteration) $\Delta_{i\,e}$ (uÅ^2) [c]
C_3H_3NO	0.00070	0.00130	0.00131
[2-^{13}C]	0.00065	0.00130	0.00131
[4-^{13}C]	0.00077	0.00134	0.00135
[5-^{13}C]	0.00057	0.00130	0.00131
[3-^{15}N]	0.00069	0.00131	0.00132
[1-^{18}O]	0.00067	0.00122	0.00123
[2-^{2}H]	0.00061	0.00126	0.00128
[2-^{2}H,2-^{13}C]	0.00060	0.00125	0.00127
[2-^{2}H,4-^{13}C]	0.00048	0.00124	0.00125
[2-^{2}H,5-^{13}C]	0.00064	0.00128	0.00130
[2-^{2}H,3-^{15}N]	0.00067	0.00132	0.00133
[2-^{2}H,1-^{18}O]	0.00033	0.00118	0.00119
[4-^{2}H]	0.00055	0.00134	0.00134
[5-^{2}H]	0.00047	0.00130	0.00130
[5-^{2}H,2-^{13}C]	0.00032	0.00115	0.00115
[5-^{2}H,4-^{13}C]	0.00049	0.00132	0.00132
[5-^{2}H,5-^{13}C]	0.00030	0.00113	0.00113
[5-^{2}H,3-^{15}N] [d]	0.00088	0.00169	0.00168
[2,4-^{2}H]	0.00031	0.00125	0.00127
[2,5-^{2}H]	0.00047	0.00127	0.00127
[2,5-^{2}H,2-^{13}C]	0.00051	0.00129	0.00129
[2,5-^{2}H,4-^{13}C]	0.00050	0.00127	0.00128
[2,5-^{2}H,5-^{13}C]	0.00048	0.00128	0.00129
[2,5-^{2}H,3-^{15}N]	0.00046	0.00130	0.00130
[2,5-^{2}H,1-^{18}O] [d]	0.00040	0.00123	0.00123
[4,5-^{2}H] [d]	0.00048	0.00133	0.00133
[2,4,5-^{2}H]	0.00048	0.00128	0.00128
[2,4,5-^{2}H,2-^{13}C]	0.00030	0.00110	0.00110
[2,4,5-^{2}H,4-^{13}C]	0.00045	0.00130	0.00131
[2,4,5-^{2}H,5-^{13}C]	0.00056	0.00134	0.00135
**Average ($\bar{x}$)**	**0.00053**	**0.00128**	**0.00129**
**Std. Dev. (s)**	**0.00014**	**0.00010**	**0.00010**

[a] Vibration-rotation interaction and electron-mass corrections at the CCSD(T)/cc-pCVTZ level.

[b] Vibration-rotation interaction corrections calculated from the preliminary r_e^{SE} geometry and electron-mass corrections at the CCSD(T)/cc-pCVTZ level.

[c] Vibration-rotation interaction corrections calculated from the r_e^{SE} structure determined using the r_e^{SE}-based geometry and electron-mass corrections at the CCSD(T)/cc-pCVTZ level.

[d] Isotopologue with A_0'' and B_0'' constants excluded from r_e^{SE} structure determination.

Table S38. Experimental and computational structural parameters of oxazole.

	r_e^{SE}(initial) CCSD(T)/cc-pCVTZ	r_e^{SE}(final) CCSD(T)/cc-pCVTZ **(recommended)**	r_e^{SE}(2nd Iteration) CCSD(T)/cc-pCVTZ	CCSD(T) BTE	CCSD(T)/ cc-pCV5Z
$R_{O–C2}$ (Å)	1.3512 (3)	1.3512 (2)	1.3512 (3)	1.3518	1.3502
$R_{O–C5}$ (Å)	1.3668 (2)	1.3667 (2)	1.3666 (2)	1.3672	1.3661
$R_{C2–N}$ (Å)	1.2879 (3)	1.2885 (2)	1.2885 (3)	1.2887	1.2884
$R_{C4–C5}$ (Å)	1.3491 (3)	1.3491 (2)	1.3491 (3)	1.3493	1.3491
$R_{C2–H}$ (Å)	1.0750 (2)	1.0746 (1)	1.0747 (2)	1.0748	1.0748
$R_{C4–H}$ (Å)	1.0740 (2)	1.0740 (1)	1.0739 (2)	1.0741	1.0740
$R_{C5–H}$ (Å)	1.0721 (2)	1.0724 (2)	1.0725 (2)	1.0726	1.0725
$\theta_{C5–O–C2}$ (°)	103.946 (17)	103.955 (13)	103.957 (16)	103.935	103.998
$\theta_{N–C2–O}$ (°)	115.085 (20)	115.067 (15)	115.067 (19)	115.065	115.062
$\theta_{C4–C5–O}$ (°)	108.076 (18)	108.088 (14)	108.091 (18)	108.097	108.065
$\theta_{O–C2–H}$ (°)	116.654 (30)	116.758 (23)	116.764 (28)	116.752	116.786
$\theta_{O–C5–H}$ (°)	116.933 (32)	116.864 (25)	116.845 (30)	116.850	116.902
$\theta_{C5–C4–H}$ (°)	129.105 (32)	129.045 (25)	129.035 (32)	129.086	129.020
$N_{isotopologues}$	30	30	30		

Table S39 through **Table S43** contain the semi-experimental substitution structure *a* and *b* coordinates and corresponding uncertainties for oxazoles using different parent isotopologue reference systems calculated using Kraitchman's equations.[15-16] This method is unable to calculate coordinates that are very close to an axis, and these situations are denoted by "Near-Axis". The "Not Available" notation that either there were no data available for the isotopologue necessary to calculate the given coordinates, or the isotopologue was one of the three excluded from the substitution structure due to poor determination of the A_0'' and B_0'' constants ([2,5-^{2}H, 1-^{18}O]-, [5-^{2}H,3-^{15}N]-, and [4,5-^{2}H]-oxazole).

Table S39. Coordinates and uncertainties of semi-experimental substitution (r_s^{SE}) structure using the normal isotopologue of oxazole reference system

Atom	C2	C4	C5	N	O	H2	H4	H5
a=	−0.1468161	−0.6218032	0.7235498	−1.1728643	1.0493886	−0.1292456	−1.2341503	1.5369355
Δa=	0.0000010	0.0000148	0.0000003	0.0000019	0.0000050	0.0000780	0.0000069	0.0000003
b=	1.06753591	−0.98962581	−0.88862493	0.28887106	0.43827358	2.14202015	−1.87203035	−1.58764292
Δb=	0.00000014	0.00000930	0.00000021	0.00000773	0.00001196	0.00000470	0.00000454	0.00000027

Table S40. Coordinates and uncertainties of semi-experimental substitution (r_s^{SE}) structure using [2-^{2}H]-oxazole reference system

Atom	C2	C4	C5	N	O	H2	H4	H5
a=	1.0465031	−0.9452498	−0.9944159	0.3869558	0.2884617	2.1121771	−1.7541609	−1.7792495
Δa=	0.0000028	0.0000072	0.0000043	0.0000376	0.0000369	−0.0000046	0.0000014	0.0000004
b=	−0.02573160	−0.72946851	0.61856182	−1.13496319	1.08993231	0.11077051	−1.43604440	1.34957036
Δb=	0.00052194	0.00002376	0.00001008	0.00000347	0.00001466	−0.00000381	0.00000905	0.00000728

Table S41. Coordinates and uncertainties of semi-experimental substitution (r_s^{SE}) structure using [5-^{2}H]-oxazole reference system

Atom	C2	C4	C5	N	O	H2	H4	H5
a=	0.7475757	Near-Axis	−1.0647996	Not Available	Not Available	1.3309320	Not Available	−2.1298841
Δa=	0.0000515	Near-Axis	0.0000394	Not Available	Not Available	0.0000074	Not Available	−0.0000002
b=	−0.81153387	1.16167265	0.32859094	Not Available	Not Available	−1.71403627	Not Available	0.45503256
Δb=	0.00004742	0.00000383	0.00012760	Not Available	Not Available	0.00000573	Not Available	−0.00000093

Table S42. Coordinates and uncertainties of semi-experimental substitution (r_s^{SE}) structure using [2,5-^{2}H]-oxazole reference system

Atom	C2	C4	C5	N	O	H2	H4	H5
a=	0.9859581	−0.5034074	−1.1334470	0.8723164	Not Available	1.8860854	Near-Axis	−2.1578149
Δa=	0.0000051	0.0000048	0.0000046	0.0000089	Not Available	−0.0000051	Near-Axis	−0.0000003
b=	0.42253848	−1.07397531	0.11824635	−0.86073134	Not Available	1.00970410	Near-Axis	0.43658887
Δb=	0.00001182	0.00000225	0.00004444	0.00000900	Not Available	−0.00000946	Near-Axis	−0.00000152

Table S43. Coordinates and uncertainties of semi-experimental substitution (r_s^{SE}) structure using [2,4,5-^{2}H]-oxazole reference system

Atom	C2	C4	C5	N	O	H2	H4	H5
a=	1.0949394	0.9134200	0.9295619	Not Available	Not Available	Not Available	1.7394927	1.6962782
Δa=	0.0000415	0.0000065	0.0000182	Not Available	Not Available	Not Available	−0.0000015	−0.0000034
b=	−0.05235637	−0.70612525	0.64272285	Not Available	Not Available	Not Available	−1.39256372	1.39274086
Δb=	0.00086754	0.00000842	0.00002631	Not Available	Not Available	Not Available	−0.00000190	−0.00000412

Table S44. Semi-experimental substitution (r_s^{SE}) structure parameters that could be determined from various isotopologue reference systems

Parameter	C_3H_3NO	[2-^{2}H]-	[5-^{2}H]-	[2,5-^{2}H]-	[2,4,5-^{2}H]-	Average	Std Dev
$r_{O\text{-}C2}$	1.3516	1.3488				1.3502	0.0020
$r_{O\text{-}C5}$	1.3663	1.3667				1.3665	0.0003
$r_{C2\text{-}N}$	1.2881	1.2905		1.2883		1.2890	0.0013
$r_{C4\text{-}C5}$	1.3491	1.3489		1.3485	1.3489	1.3489	0.0003
$r_{N\text{-}C4}$	1.3922	1.3926		1.3922		1.3923	0.0002
$r_{C2\text{-}H}$	1.0746	1.0744	1.0746	1.0747		1.0746	0.0001
$r_{C4\text{-}H}$	1.0741	1.0741			1.0741	1.0741	0.0000
$r_{C5\text{-}H}$	1.0725	1.0725	1.0726	1.0727	1.0726	1.0726	0.0001
$\theta_{C5\text{-}O\text{-}C2}$	103.950	104.019				103.985	0.049
$\theta_{N\text{-}C2\text{-}O}$	115.059	115.070				115.064	0.008
$\theta_{C2\text{-}N\text{-}C4}$	103.878	103.807		103.872		103.852	0.039
$\theta_{N\text{-}C4\text{-}C5}$	109.024	109.018		109.044		109.028	0.013
$\theta_{C4\text{-}C5\text{-}O}$	108.090	108.086				108.088	0.003
$\theta_{O\text{-}C2\text{-}H}$	116.810	116.895				116.852	0.060
$\theta_{N\text{-}C2\text{-}H}$	128.132	128.035		128.178		128.115	0.073
$\theta_{N\text{-}C4\text{-}H}$	121.924	121.934				121.929	0.007
$\theta_{H\text{-}C4\text{-}C5}$	129.052	129.048			129.040	129.047	0.006
$\theta_{C4\text{-}C5\text{-}H}$	135.031	135.055		135.118	135.055	135.065	0.037
$\theta_{O\text{-}C5\text{-}H}$	116.879	116.859				116.869	0.014

(a)

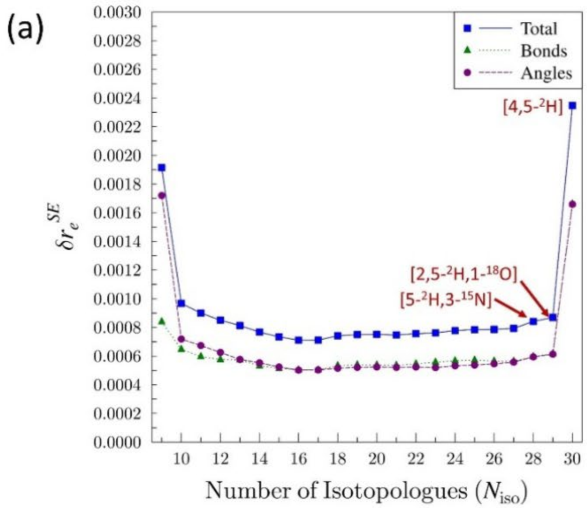

N_{iso}	Isotopologue Added	N_{iso}	Isotopologue Added
9	Core set	22	[2-^{2}H, 5-^{13}C]
10	[2-^{2}H, 1-^{18}O]	23	[2,4,5-^{2}H, 5-^{13}C]
11	[5-^{2}H, 4-^{13}C]	24	[2,5-^{2}H]
12	[2,4-^{2}H]	25	[2,5-^{2}H, 5-^{13}C]
13	[2,5-^{2}H, 2-^{13}C]	26	[2-^{2}H, 3-^{15}N]
14	[2,5-^{2}H, 4-^{13}C]	27	[2,5-^{2}H, 3-^{15}N]
15	[2-^{2}H, 4-^{13}C]	28	[5-^{2}H, 3-^{15}N]
16	[2,4,5-^{2}H, 4-^{13}C]	29	[2,5-^{2}H, 1-^{18}O]
17	[2-^{2}H, 2-^{13}C]	30	[4,5-^{2}H]
18	[2,4,5-^{2}H]		
19	[2,4,5-^{2}H, 2-^{13}C]		
20	[5-^{2}H, 2-^{13}C]		
21	[5-^{2}H, 5-^{13}C]		

(b)

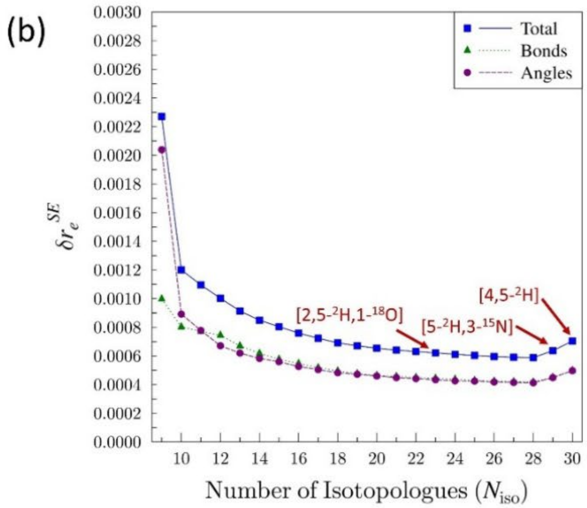

N_{iso}	Isotopologue Added	N_{iso}	Isotopologue Added
9	Core set	22	[2-^{2}H, 2-^{13}C]
10	[2-^{2}H, 1-^{18}O]	23	[2,5-^{2}H, 1-^{18}O]
11	[2,5-^{2}H, 2-^{13}C]	24	[5-^{2}H, 5-^{13}C]
12	[2,4,5-^{2}H, 5-^{13}C]	25	[2-^{2}H, 3-^{15}N]
13	[2,5-^{2}H, 4-^{13}C]	26	[2,4-^{2}H]
14	[2,4,5-^{2}H, 2-^{13}C]	27	[2,4,5-^{2}H]
15	[2,5-^{2}H, 3-^{15}N]	28	[2,5-^{2}H]
16	[2-^{2}H, 5-^{13}C]	29	[5-^{2}H, 3-^{15}N]
17	[2-^{2}H, 4-^{13}C]	30	[4,5-^{2}H]
18	[5-^{2}H, 2-^{13}C]		
19	[5-^{2}H, 4-^{13}C]		
20	[2,5-^{2}H, 5-^{13}C]		
21	[2,4,5-^{2}H, 4-^{13}C]		

(c)

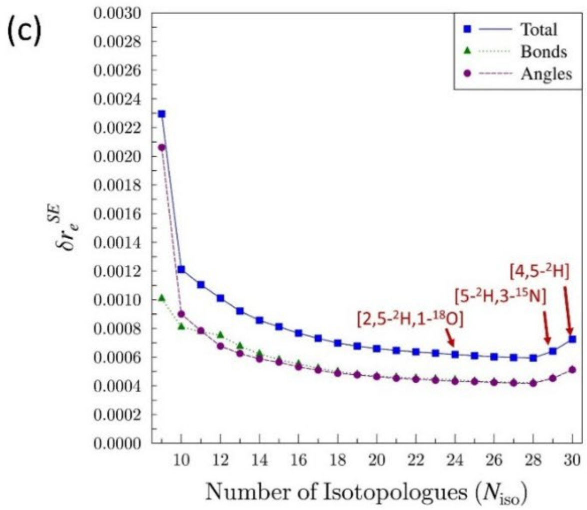

N_{iso}	Isotopologue Added	N_{iso}	Isotopologue Added
9	Core set	22	[2-^{2}H, 2-^{13}C]
10	[2-^{2}H, 1-^{18}O]	23	[5-^{2}H, 5-^{13}C]
11	[2,5-^{2}H, 2-^{13}C]	24	[2,5-^{2}H, 1-^{18}O]
12	[2,4,5-^{2}H, 5-^{13}C]	25	[2-^{2}H, 3-^{15}N]
13	[2,5-^{2}H, 4-^{13}C]	26	[2,4-^{2}H]
14	[2,4,5-^{2}H, 2-^{13}C]	27	[2,4,5-^{2}H]
15	[2,5-^{2}H, 3-^{15}N]	28	[2,5-^{2}H]
16	[2-^{2}H, 5-^{13}C]	29	[5-^{2}H, 3-^{15}N]
17	[2-^{2}H, 4-^{13}C]	30	[4,5-^{2}H]
18	[5-^{2}H, 2-^{13}C]		
19	[5-^{2}H, 4-^{13}C]		
20	[2,5-^{2}H, 5-^{13}C]		
21	[2,4,5-^{2}H, 4-^{13}C]		

Figure S32. Plot of δr_e^{SE} values as a function of the number of isotopologues (N_{iso}) incorporated in the structure determination. The total uncertainty (δr_e^{SE} Total, blue squares), the bond-distance uncertainty (δr_e^{SE} Bonds, green triangles), and the uncertainty in the angles (δr_e^{SE} Angle, purple circles) are presented. The isotopologue ordering along the *x*-axis is provided in the table. (a) Initial, using the original r_e-based vibration-rotation interaction corrections. (b) Final, using first iteration of r_e^{SE}-based vibration-rotation interaction corrections. (c) Using second iteration of r_e^{SE}-based vibration-rotation interaction corrections (using r_e^{SE} parameter values from (b) as input).

(a)

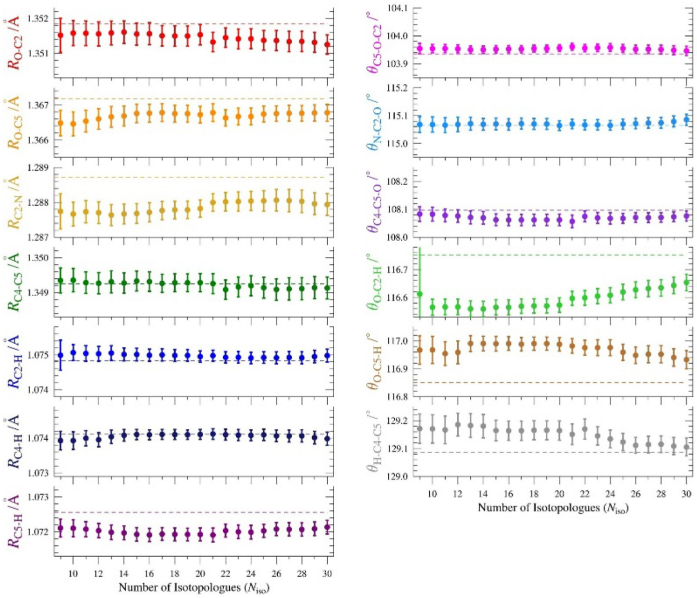

N_{iso}	Isotopologue Added
9	Core set
10	[2-^{2}H, 1-^{18}O]
11	[5-^{2}H, 4-^{13}C]
12	[2,4-^{2}H]
13	[2,5-^{2}H, 2-^{13}C]
14	[2,5-^{2}H, 4-^{13}C]
15	[2-^{2}H, 4-^{13}C]
16	[2,4,5-^{2}H, 4-^{13}C]
17	[2-^{2}H, 2-^{13}C]
18	[2,4,5-^{2}H]
19	[2,4,5-^{2}H, 2-^{13}C]
20	[5-^{2}H, 2-^{13}C]
21	[5-^{2}H, 5-^{13}C]
22	[2-^{2}H, 5-^{13}C]
23	[2,4,5-^{2}H, 5-^{13}C]
24	[2,5-^{2}H]
25	[2,5-^{2}H, 5-^{13}C]
26	[2-^{2}H, 3-^{15}N]
27	[2,5-^{2}H, 3-^{15}N]
28	[5-^{2}H, 3-^{15}N]
29	[2,5-^{2}H, 1-^{18}O]
30	[4,5-^{2}H]

(b)

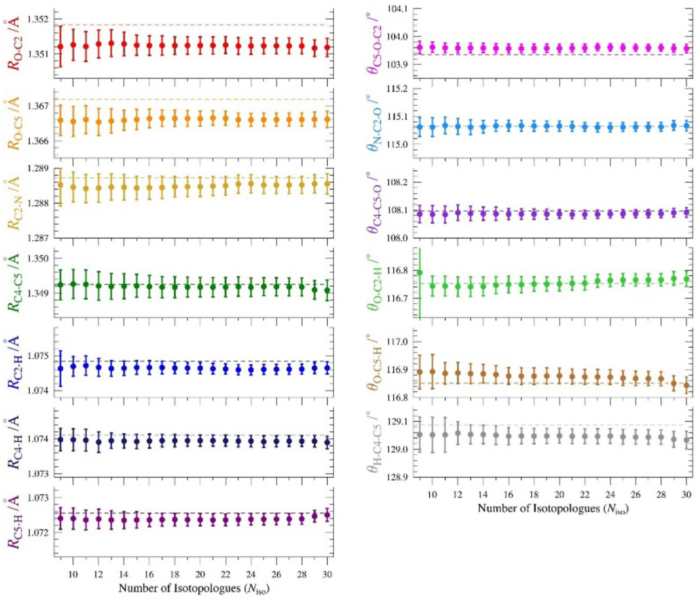

N_{iso}	Isotopologue Added
9	Core set
10	[2-^{2}H, 1-^{18}O]
11	[2,5-^{2}H, 2-^{13}C]
12	[2,4,5-^{2}H, 5-^{13}C]
13	[2,5-^{2}H, 4-^{13}C]
14	[2,4,5-^{2}H, 2-^{13}C]
15	[2,5-^{2}H, 3-^{15}N]
16	[2-^{2}H, 5-^{13}C]
17	[2-^{2}H, 4-^{13}C]
18	[5-^{2}H, 2-^{13}C]
19	[5-^{2}H, 4-^{13}C]
20	[2,5-^{2}H, 5-^{13}C]
21	[2,4,5-^{2}H, 4-^{13}C]
22	[2-^{2}H, 2-^{13}C]
23	[2,5-^{2}H, 1-^{18}O]
24	[5-^{2}H, 5-^{13}C]
25	[2-^{2}H, 3-^{15}N]
26	[2,4-^{2}H]
27	[2,4,5-^{2}H]
28	[2,5-^{2}H]
29	[5-^{2}H, 3-^{15}N]
30	[4,5-^{2}H]

(c)

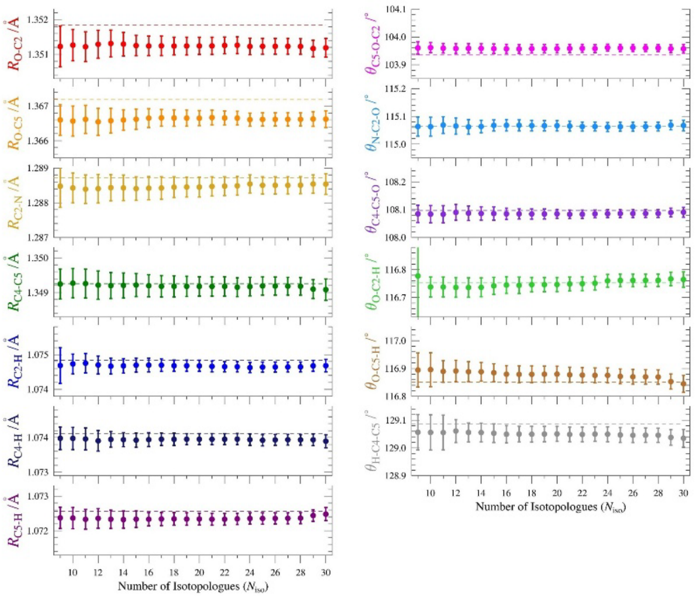

N_{iso}	Isotopologue Added
9	Core set
10	[2-^{2}H, 1-^{18}O]
11	[2,5-^{2}H, 2-^{13}C]
12	[2,4,5-^{2}H, 5-^{13}C]
13	[2,5-^{2}H, 4-^{13}C]
14	[2,4,5-^{2}H, 2-^{13}C]
15	[2,5-^{2}H, 3-^{15}N]
16	[2-^{2}H, 5-^{13}C]
17	[2-^{2}H, 4-^{13}C]
18	[5-^{2}H, 2-^{13}C]
19	[5-^{2}H, 4-^{13}C]
20	[2,5-^{2}H, 5-^{13}C]
21	[2,4,5-^{2}H, 4-^{13}C]
22	[2-^{2}H, 2-^{13}C]
23	[5-^{2}H, 5-^{13}C]
24	[2,5-^{2}H, 1-^{18}O]
25	[2-^{2}H, 3-^{15}N]
26	[2,4-^{2}H]
27	[2,4,5-^{2}H]
28	[2,5-^{2}H]
29	[5-^{2}H, 3-^{15}N]
30	[4,5-^{2}H]

Figure S33. Plots of the structural parameters as a function of the number of isotopologues (N_{iso}) and their 2σ uncertainties with consistent scales for each distance (0.0020 Å) and each angle (0.25°). The dashed line in each plot is the BTE value calculated for that parameter. The isotopologue included in the structural least-squares fit corresponding to the total number along the x-axis is provided in the table to the right.

(a) Initial, using the original r_e-based vibration-rotation interaction corrections.

(b) Final, using first iteration of r_e^{SE}-based vibration-rotation interaction corrections.

(c) Using second iteration of r_e^{SE}-based vibration-rotation interaction corrections (using r_e^{SE} parameter values from (b) as input).

Experimental Procedures for Oxazole Isotopologues

Method 1: H/D exchange by weak base at room temperature (Scheme S1).

In a beaker, 4.56 g Na_2CO_3 (43 mmol, 1.2 eq.) was dissolved in 25 mL D_2O. This solution was added to a 50-mL round-bottom flask containing a magnetic stir bar and 2.50 g oxazole (36.2 mmol, 1 eq.). The solution was loosely capped and stirred at room temperature for 9 days. Reaction progress was monitored by taking aliquots for analysis by mass spectrometry and both ^{1}H-NMR and ^{2}H-NMR spectroscopy. Once it appeared the majority of oxazole had been deuteriated based on the mass spectrum of an aliquot of the reaction mixture, the oxazole was isolated *via* short-path distillation (bp = 69 °C) out of the D_2O solution without prior neutralization. The distillation yielded 1.54 g (~ 61% yield) of colorless liquid.

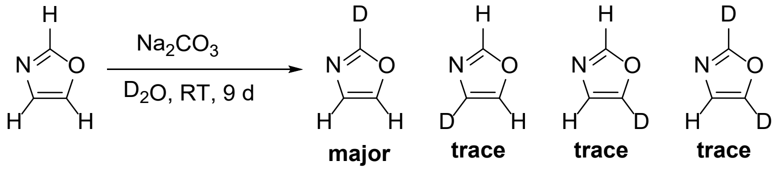

Scheme S1. Deuteriation of oxazole by carbonate-mediated exchange with D_2O at room temperature.

Method 2: H/D exchange by weak base in reflux (Scheme S2).

In a beaker, 5.19 g Na_2CO_3 (49 mmol, 1.3 eq.) were dissolved in 25 mL D_2O. This solution was added to a 50-mL round-bottom flask containing a magnetic stir bar and 2.51 g oxazole (36.4 mmol, 1 eq.). The round-bottom flask was fitted with a water-cooled reflux condenser. The solution was stirred at reflux for 44 hours. Within 19 hours, the solution had turned a deep brownish-orange. After cooling to room temperature, oxazole was isolated *via* short-path distillation (bp = 69 °C) out of the D_2O solution without prior neutralization. The distillation yielded 1.64 g (~ 65% yield) of colorless liquid.

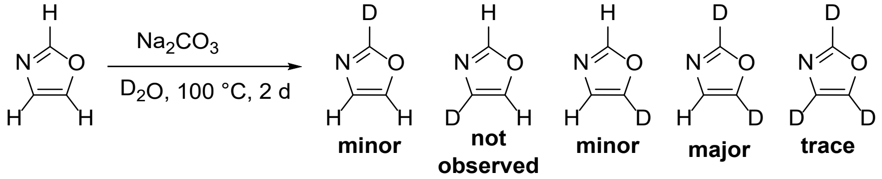

Scheme S2. Deuteriation of oxazole by carbonate-mediated exchange with D_2O at reflux.

Method 3: H/D exchange by weak base under pressurized conditions (Scheme S3)

To a steel vessel (~150 mL, Swagelok DOT-3E 1800 304L-HDF-150, Figure S34) were added a magnetic stir bar, 2 g of oxazole (1 eq.), 1.40 g K_2CO_3 (0.3 eq.), and 7.06 g D_2O (12.2 eq). The steel vessel was sealed tightly using Teflon tape and brass fittings, then placed in an oil bath at 140 °C for approximately five hours behind a blast shield. Due to a coincidental situation, the vessel was allowed to sit at ambient temperature for one week. The material recovered from the steel vessel contained a dark liquid, as well as pearlescent black solid. Oxazole was distilled from this mixture using a Vigreux column at temperatures less than 90 °C. The clear, colorless distillate (~ 1.4 g) was transferred back into the steel vessel along with a fresh 1.38 g K_2CO_3 (~0.5 eq, assuming some deuterium incorporation) and 6.95 g D_2O (~17 eq., assuming some deuterium incorporation). The vessel was resealed and placed in the oil bath at 140 °C for approximately 4.5 hours behind a blast shield. After the vessel had cooled, oxazole was distilled anew at temperatures less than 90 °C using a Vigreux column. The final clear, colorless distillate contained predominantly [2,5-^{2}H]-oxazole with [2,4,5-^{2}H]-oxazole the next-most abundant oxazole specie (determined by rotational spectroscopy). A small amount of the normal isotopologue of oxazole was also observed.

Single-cycle variations of this reaction were also carried out with a larger amount of D_2O and for longer heating duration (approximately three days), but the reaction yielded less distillate and did not result in more complete deuterium incorporation. Based on the observed distributions of deuterio-isotopologues, the 2- and 5- positions exchange rapidly, while the 4-position is quite slow.

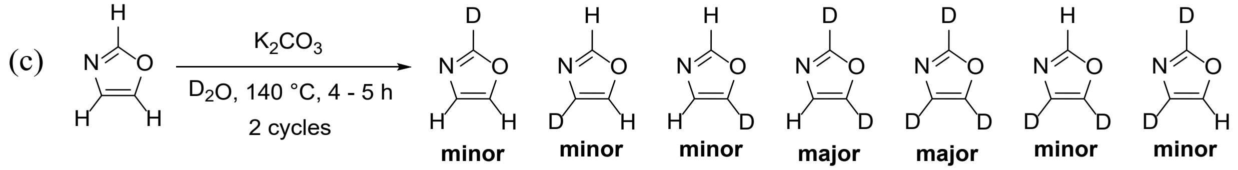

Scheme S3. Deuteriation of oxazole by carbonate-mediated exchange with D_2O under pressurized conditions.

General Note. Acid-catalyzed H/D exchange in 1,3-oxazole was not viable. In contrast to a similar reaction of thiazole, minimal exchange was observed when oxazole was stirred in D_2O with D_2SO_4 for two weeks at room temperature. Heating of this acidic solution led to rearrangement of oxazole into pyrazine, as previously reported.[17-18]

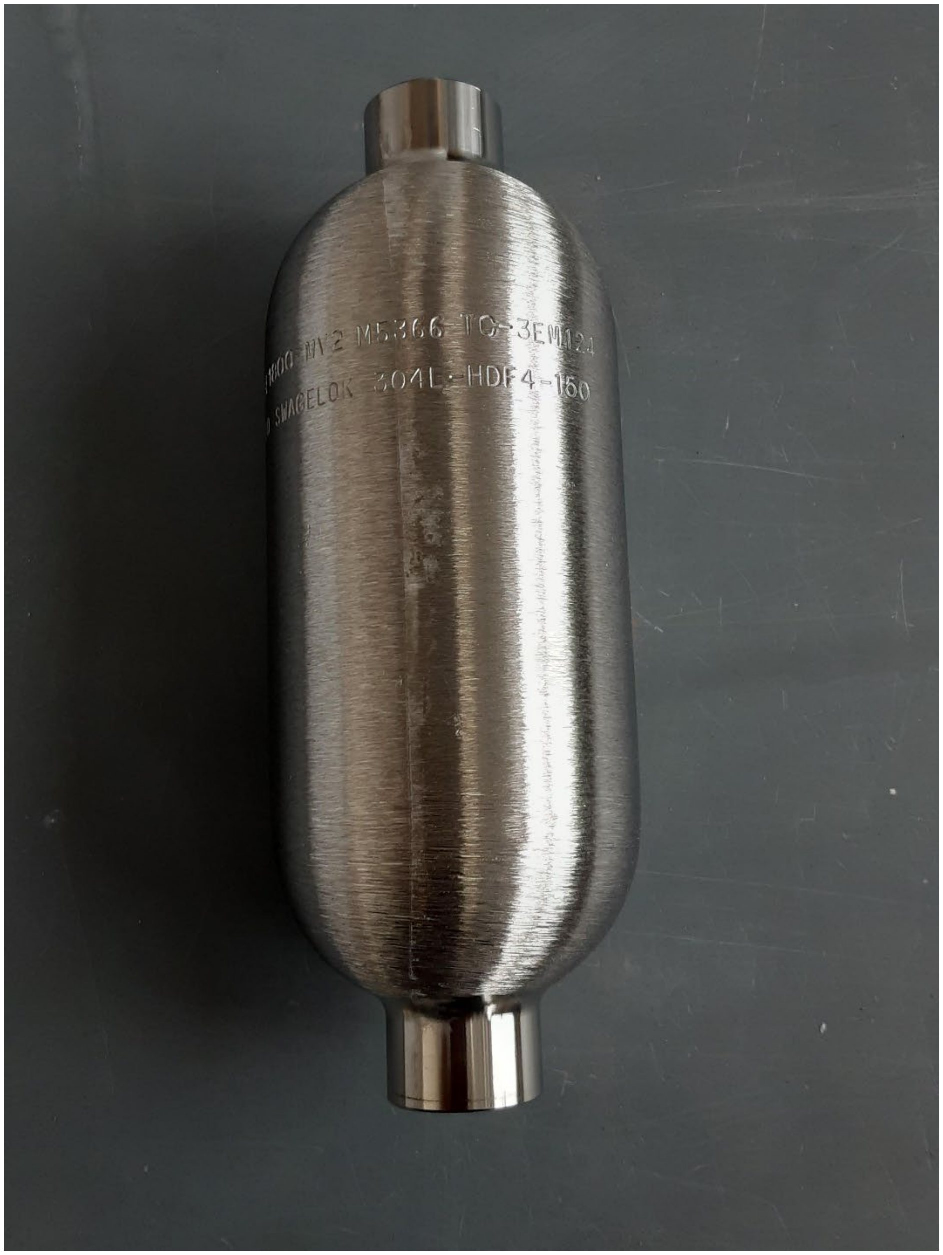

Figure S34. Photograph of the steel vessel (excluding fittings) used for reaction.

Mass Spectral data of product from Method 1

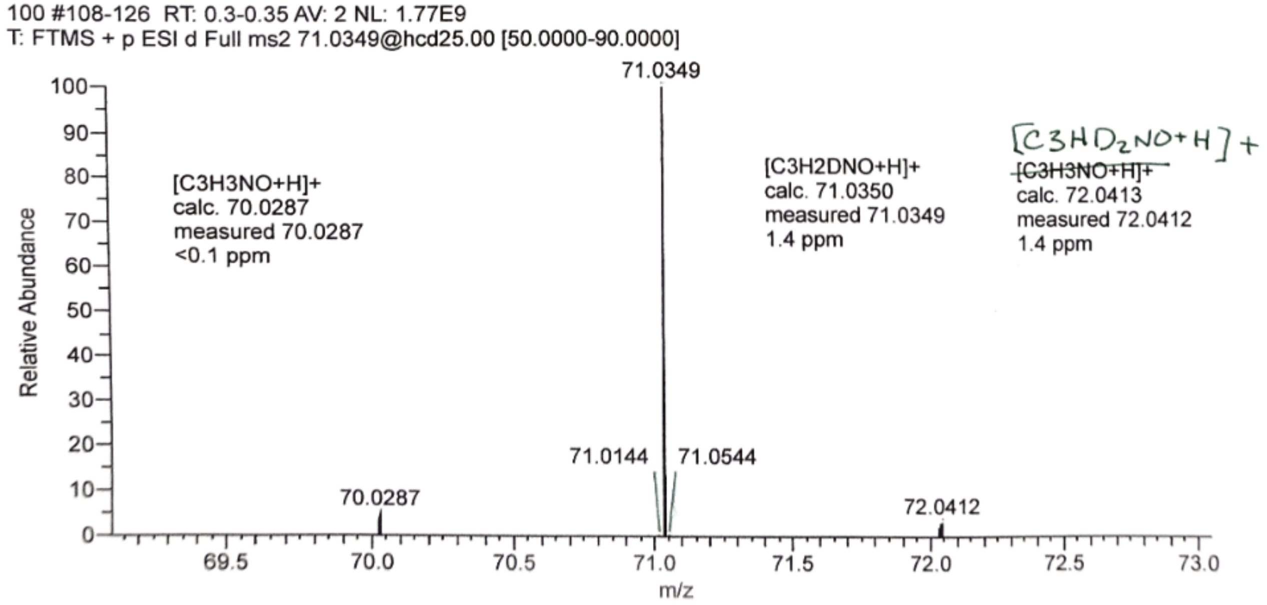

Mass Spectral data of product from Method 2

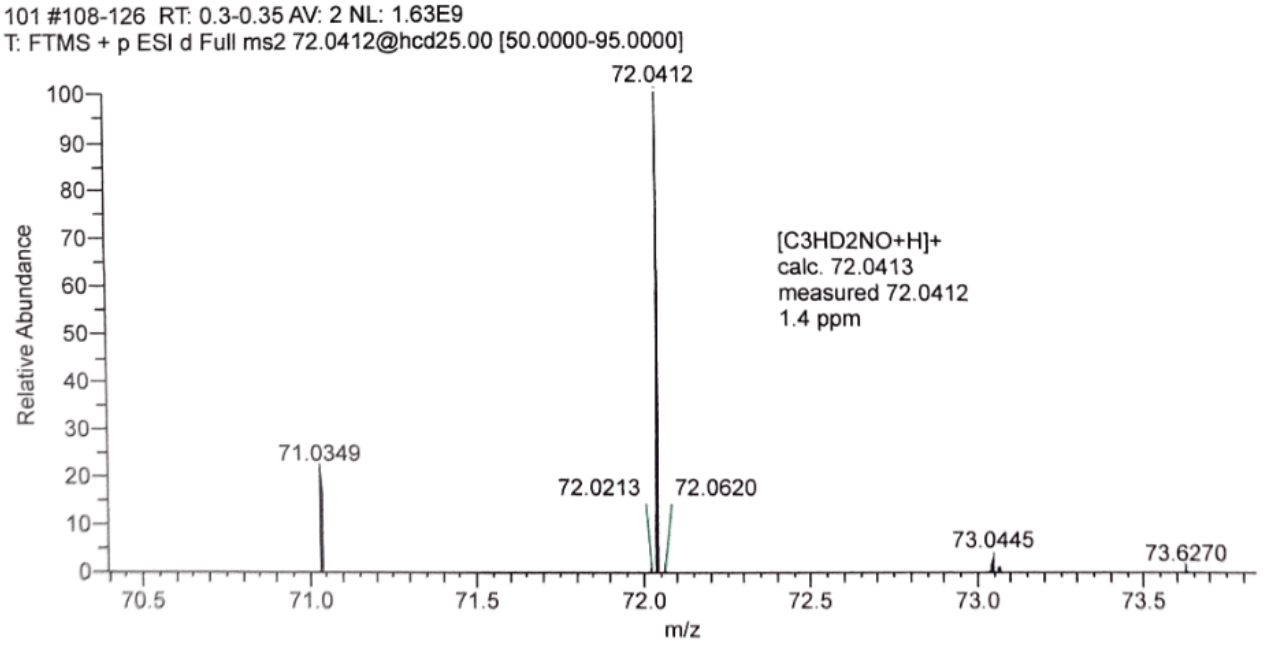

^{1}H-NMR of product from Method 1

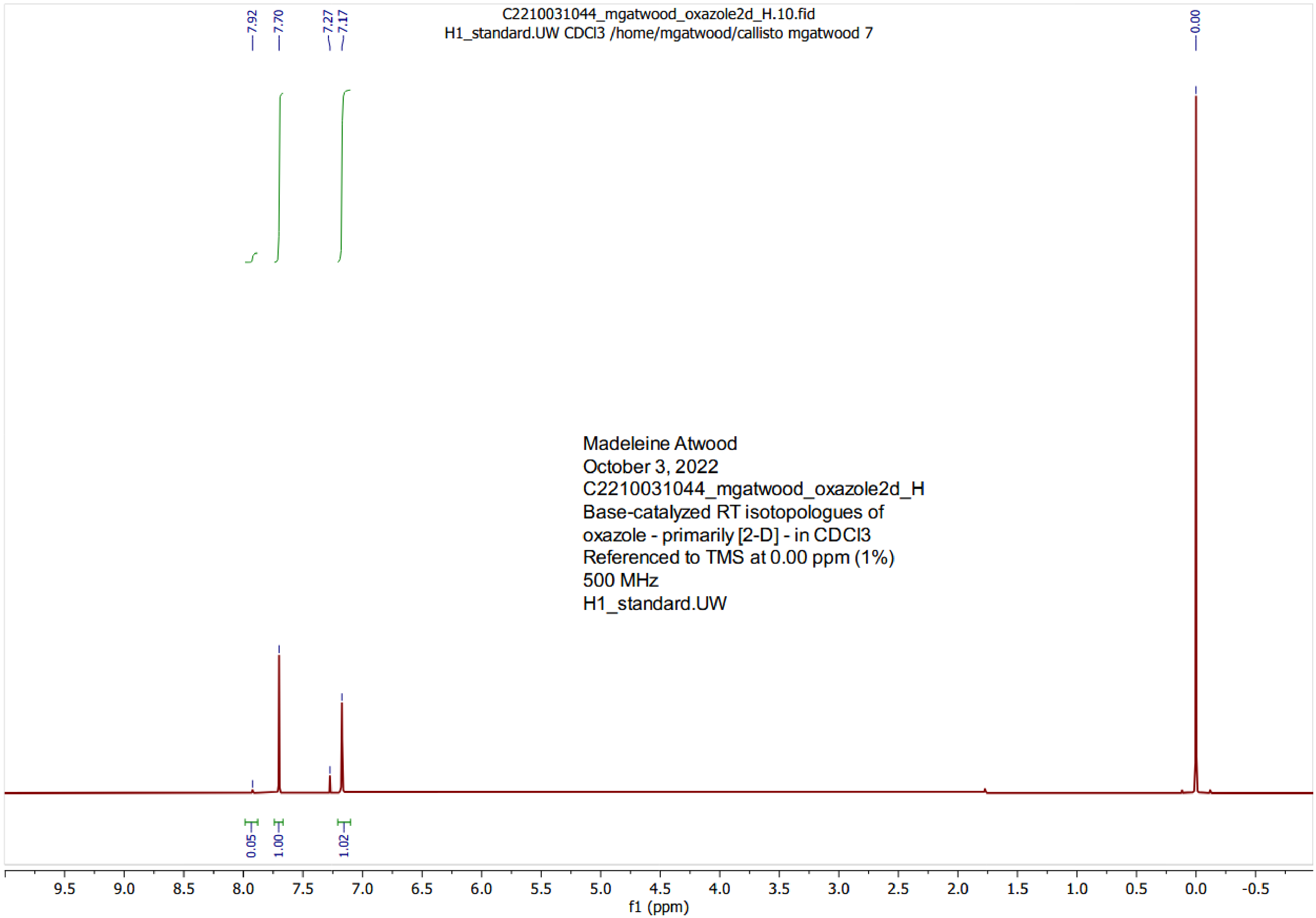

^{2}H-NMR of product from Method 1

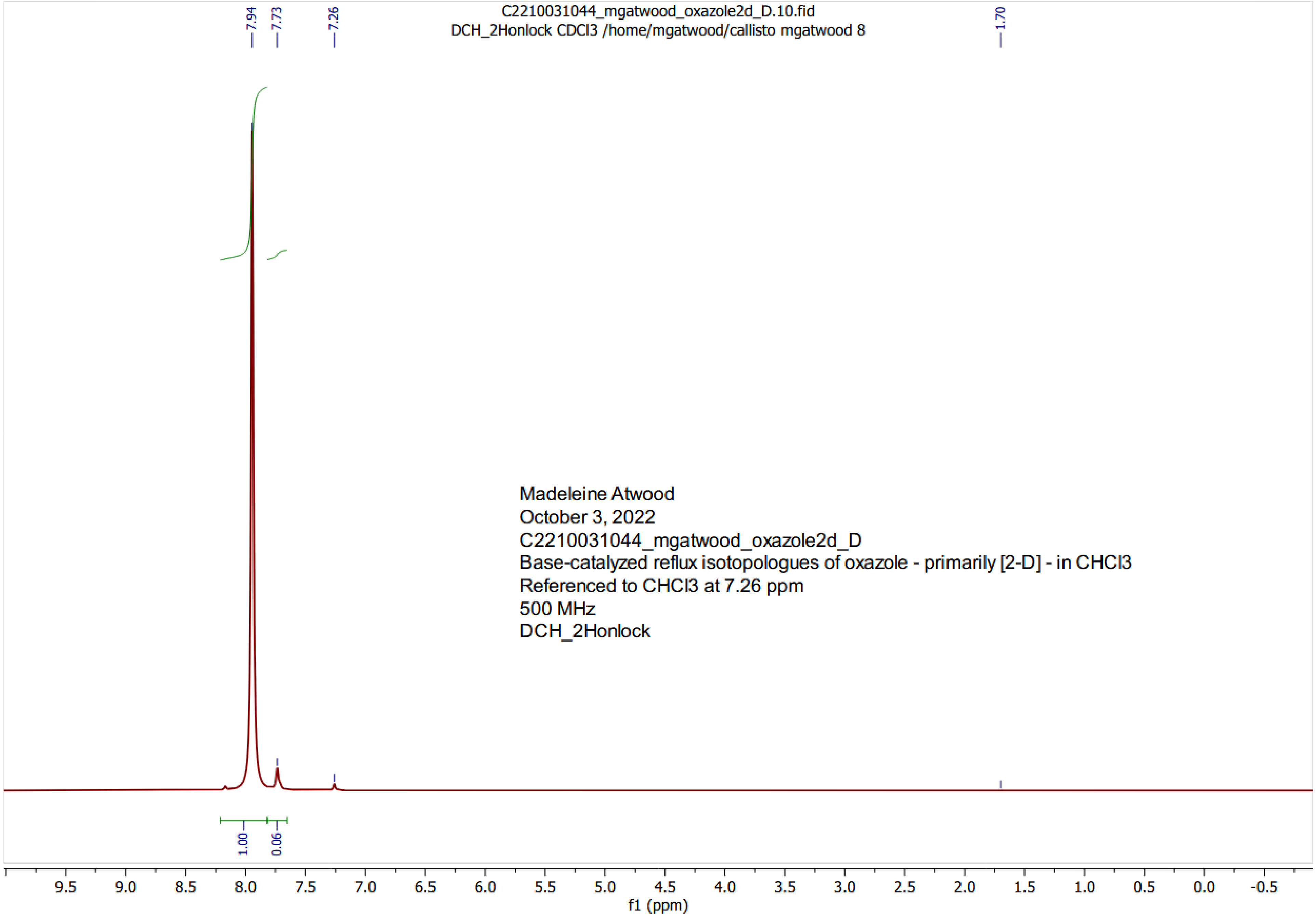

^{1}H-NMR of product from Method 2

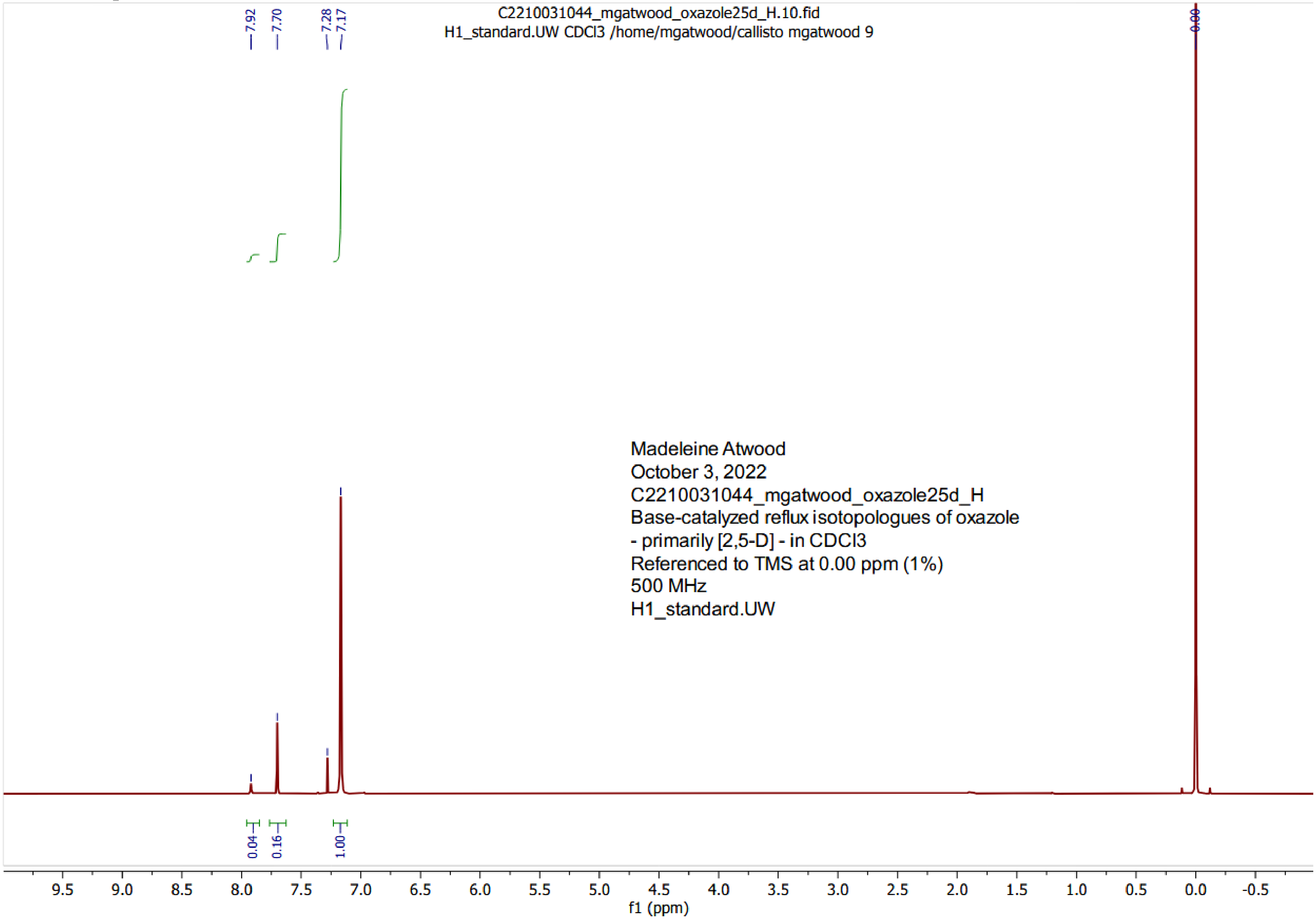

^{2}H-NMR of product from Method 2

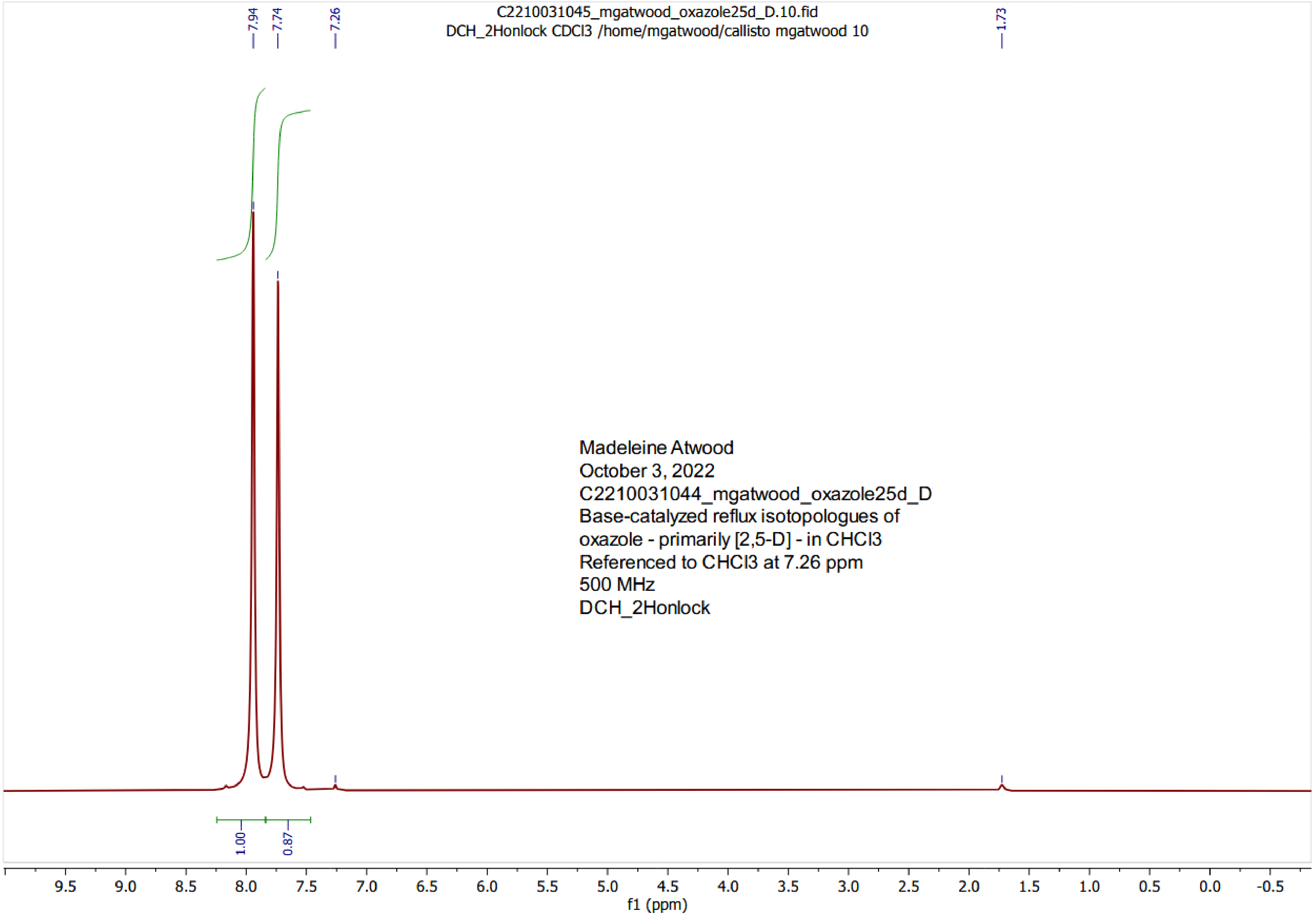

References

(1) Gordy, W.; Cook, R. L., *Microwave Molecular Spectra*. 3rd ed.; Wiley Interscience: New York, 1984.

(2) Morgan, W. J.; Matthews, D. A.; Ringholm, M.; Agarwal, J.; Gong, J. Z.; Ruud, K.; Allen, W. D.; Stanton, J. F.; Schaefer, H. F., III, Geometric Energy Derivatives at the Complete Basis Set Limit: Application to the Equilibrium Structure and Molecular Force Field of Formaldehyde *J. Chem. Theory Comput.* **2018**, *14*, 1333−1350.

(3) Puzzarini, C.; Bloino, J.; Tasinato, N.; Barone, V., Accuracy and Interpretability: The Devil and the Holy Grail. New Routes across Old Boundaries in Computational Spectroscopy. *Chem. Rev.* **2019**, *119*, 8131-8191.

(4) Feller, D., The use of systematic sequences of wave functions for estimating the complete basis set, full configuration interaction limit in water *J. Chem. Phys.* **1993**, *98*, 7059-7071.

(5) Bomble, Y. J.; Stanton, J. F.; Kállay, M.; Gauss, J., Coupled-cluster methods including noniterative corrections for quadruple excitations. *J. Chem. Phys.* **2005**, *123*, 054101.

(6) Cheng, L.; Gauss, J., Analytic energy gradients for the spin-free exact two-component theory using an exact block diagonalization for the one-electron Dirac Hamiltonian. *J. Chem. Phys.* **2011**, *135*, 084114.

(7) Dyall, K. G., Interfacing relativistic and nonrelativistic methods. II. Investigation of a low-order approximation. *J. Chem. Phys.* **1998**, *109*, 4201-4208.

(8) Liu, W.; Peng, D., Exact two-component Hamiltonians revisited. *J. Chem. Phys.* **2009**, *131*, 031104.

(9) Handy, N. C.; Yamaguchi, Y.; Schaefer, H. F., III, The Diagonal Correction to the Born-Oppenheimer Approximation: Its Effect on the Singlet-Triplet Splitting of CH_2 and Other Molecular Effects. *J. Chem. Phys.* **1986**, *84*, 4481-4484.

(10) Born, M.; Huang, K., *Dynamical Theory of Crystal Lattices*. Oxford Univ. Press: 1954; p 430 pp.

(11) Hegelund, F.; Larsen, R. W.; Palmer, M. H., High-resolution infrared and theoretical study of gaseous oxazole in the 600–1400 cm^{-1} region. *J. Mol. Spectrosc.* **2007**, *241*, 26-44.

(12) Demaison, J.; Császár, A. G.; Margulès, L. D.; Rudolph, H. D., Equilibrium Structures of Heterocyclic Molecules with Large Principal Axis Rotations upon Isotopic Substitution. *J. Phys. Chem. A* **2011**, *115*, 14078-14091.

(13) Townes, C. H.; Schawlow, A. L., *Microwave Spectroscopy*. Dover Publications, Inc.: New York, 1975; p. 11.

(14) Puzzarini, C.; Stanton, J. F.; Gauss, J., Quantum-chemical calculation of spectroscopic parameters for rotational spectroscopy. *Int. Rev. Phys. Chem.* **2010**, *29*, 273-367; Eqn 143 and accompanying text.

(15) Kraitchman, J., Determination of Molecular Structure from Microwave Spectroscopic Data. *Am. J. Phys.* **1953**, *21*, 17-24.

(16) Rudolph, H. D., Extending Kraitchman's equations. *J. Mol. Spectrosc.* **1981**, *89*, 430-439.

(17) Venkatasubramanian, R.; Krishnamachari, S. L. N. G., Synthesis of (2-D)-Thiazole and (2-D)-Oxazole and Formation of Pyrazine from Reaction of Oxazole. *Indian J. Chem., Sect B* **1990**, *29B*, 562-563.

(18) Esselman, B. J.; Zdanovskaia, M. A.; Owen, A. N.; Stanton, J. F.; Woods, R. C.; McMahon, R. J., Precise equilibrium structure of thiazole (*c*-C_3H_3NS) from twenty-four isotopologues. *J. Chem. Phys.* **2021**, *155*, 054302.
